# Meta-analysis of the effects of exercise interventions on dialysis patients with cardiac function disorders

**DOI:** 10.3389/fmed.2025.1573498

**Published:** 2025-05-13

**Authors:** Huizhen Liu, Ming Zhang, Minqi Chen, Liusi Chen, Tingrong Huang, Zhao Ye

**Affiliations:** ^1^Hubei University of Traditional Chinese Medicine, Wuhan, China; ^2^Huangshi Hospital of Traditional Chinese Medicine, Huangshi, China

**Keywords:** dialysis, exercise, cardiac function, LVEF, meta-analysis

## Abstract

**Background:**

Prolonged dialysis can lead patients to multiple complications, with heart failure being the most dangerous and the leading cause of death among dialysis patients. Concurrently, exercise has been shown to improve several indicators of heart function.

**Methods:**

A comprehensive search was conducted across seven databases. The search was limited to studies published between January 2010 and July 2024.

**Results:**

(1) Compared to conventional care, exercise significantly increased the left ventricular ejection fraction (LVEF) in dialysis patients (MD = 2.00, 95% CI: 1.15–2.84, *p* < 0.001). Subgroup analysis revealed that combined aerobic and resistance exercise led to a more substantial increase in LVEF compared to aerobic exercise (MD = 2.78, 95% CI: 1.17–4.38, *p* < 0.001). Each workout lasting more than 30 min was associated with a significant increase in LVEF compared to sessions lasting 30 min or less (MD = 2.5, 95% CI:0.57–4.43, *p* = 0.001). An exercise intervention cycle of 10 to 12 weeks resulted in a significant increase in LVEF compared to cycles longer than 12 weeks (MD = 3.36, 95% CI: 2.04–4.68, *p* < 0.001). A weekly exercise frequency of more than three times per week significantly improved LVEF compared to three times per week or less (MD = 2.73, 95% CI: 1.21–4.25, *p* < 0.001). (2) In comparison to conventional care, exercise effectively reduced the left ventricular mass index (LVMI) of dialysis patients (MD = −7.93, 95% CI: −14.67--1.19, *p* = 0.02). However, exercise interventions did not demonstrate statistically significant improvements in pulse wave velocity (PWV), left ventricular end-systolic volume (LVESV), and left ventricular end-diastolic volume (LVEDV).

**Conclusion:**

Exercise can significantly enhance LVEF and decrease LVMI in dialysis patients. However, no significant improvements were observed in PWV, LVESV, or LVEDV. The subgroup analysis indicated that a combination of aerobic and resistance exercise had a better effect on improving LVEF compared to aerobic exercise, and exercise intervention with each workout lasting for more than 30 min, an exercise intervention cycle of 10 to 12 weeks, and an exercise frequency of more than three times per week were more effective in improving LVEF.

## 1 Introduction

End-stage renal disease (ESRD) represents the final stage of chronic kidney disease (CKD), and hemodialysis (HD) is a crucial and frequently utilized renal replacement therapy for patients with ESRD. Statistics indicate that the prevalence of ESRD and the utilization rate of renal replacement therapy are increasing annually on a global scale (1). In China, the in-hospital mortality rate for patients with CKD is 2.6%, significantly higher than the 0.8% rate for non-CKD patients. Among CKD patients, those with heart failure experience an even greater in-hospital mortality rate, reaching 7.8% (2). Once heart failure occurs, it can lead to substantial morbidity and mortality.

Heart failure is a common complication among patients undergoing HD. Pathological changes in cardiac structure and function are directly associated with the incidence of cardiovascular events in dialysis patients. These changes include left ventricular hypertrophy (LVH), left ventricular dilation, left ventricular systolic or diastolic dysfunction, and aortic sclerosis, among others (3). When the left ventricular ejection fraction (LVEF) is 40% or lower, the risk of heart failure increases, often accompanied by progressive left ventricular dilation and adverse cardiac remodeling (4). The reduction in cardiac output and coronary artery perfusion results in ischemia of myocardial cells and abnormal regional wall motion, a condition known as myocardial stunning. Repeated episodes of myocardial stunning can exacerbate heart failure. Research has demonstrated that exercise can enhance the quality of life and functional capacity of dialysis patients, as well as alleviate hemodialysis-induced myocardial stunning and reduce its incidence (5, 6). LVH is the most prevalent cardiac abnormality observed in patients with end-stage renal disease (ESRD). Factors associated with LVH include left ventricular mass index (LVMI), arterial stiffness, and volume overload (7). Studies have shown a significant correlation between LVMI and residual renal function, indicating that LVMI is lower when urine output exceeds 250 mL per day (8). Aortic pulse wave velocity (PWV) is a significant predictor of cardiovascular disease and mortality in patients with ESRD. Studies have shown that managing circulating volume can lower blood pressure, promote regression of LVH, and improve survival rates among dialysis patients (9).

According to the Kidney Association’s Clinical Practice Guidelines for Hemodialysis, exercise rehabilitation therapy should be provided for dialysis patients who do not have contraindications to exercise (such as no lower limb fractures, bone or joint lesions, or activity disorders within the past six months) (10). As research progresses, it has been discovered that, in addition to traditional aerobic exercise (such as cycling, recumbent pedaling, stair climbing, and tai chi, etc.), resistance exercise (such as tying sandbags, elastic bands, and dumbbells, etc.) and combined exercise, other innovative forms of exercise (such as virtual reality gaming, yoga for dialysis patients, electrical muscle stimulation, and blood flow restriction training, etc.) can significantly enhance the immune system of individuals undergoing dialysis. These exercises can also reduce the risk of joint stiffness, improve muscle strength, enhance heart function, regulate blood pressure, improve quality of life and sleep, and ultimately promote health (11, 12). Tieming et al. discovered that exercise can significantly enhance the cardiac function of patients undergoing dialysis (13). However, Grigoriou et al. found that exercise had no significant effect on improving the cardiac function of these patients (14).

Therefore, it is essential to collect large samples, comprehensively evaluate eligible studies, and analyze the impact of exercise on improving cardiac function in dialysis patients. This study aims to screen all eligible trials and systematically analyze the effects of exercise rehabilitation on LVEF, PWV, LVMI, left ventricular end-systolic volume (LVESV), and left ventricular end-diastolic volume (LVEDV) in dialysis patients.

## 2 Methods

### 2.1 Research registration

The Preferred Reporting Items for Systematic Reviews and Meta-Analysis (PRISMA) statement criteria and the Cochrane Handbook for Systematic Reviews of Interventions were followed in the conduct of this study (15). This systematic review has been registered with PROSPERO, and the registration number is CRD42024574659.

### 2.2 Search strategy

After discussions among all the members of the research team, a literature search strategy was developed. The primary search terms will included “Renal Dialysis “, “Aerobic Exercise,” “Resistance Exercise,” “Physical Activity “, “Stretching,” “Cycling,” “physical fitness,” “exercise training “, and “Randomized Controlled Trial “, combined with free-text keywords for comprehensive searches. This approach aims to identify randomized controlled trials and assess the impact of exercise intervention programs (aerobic exercise, resistance exercise, or combined exercise) on cardiovascular outcome indicators. The search period was from January 2010 to July 2024 for all published articles. Three English databases (PubMed, Web of Science, and Cochrane Library) and four Chinese databases (China National Knowledge Infrastructure, Wanfang, VIP, and China Biomedical Literature Service System) were searched. The search strategy is detailed in Table 1.

**Table 1:** Literature search strategy.

Search strategy in the china national knowledge infrastructure	Search strategy in PubMed
#1 (Subject: Dialysis) OR (Subject: Hemodialysis) OR (Subject: Peritoneal Dialysis)	#1 “Renal Dialysis”[Mesh]
#2 (Subject: Exercise) OR (Subject: Aerobic Exercise) OR (Subject: Resistance Exercise) OR (Subject: Physical Activity) OR (Subject: Stretching) OR (Subject: Cycling) OR (Subject: Tai Chi) OR (Subject: Physical Fitness) OR (Subject: Baduanjin)	#2 (“Renal Dialysis”[Mesh]) OR (((((((((Dialysis, Renal[Title/Abstract]) OR (Renal Dialyses[Title/Abstract])) OR (Dialyses, Renal[Title/Abstract])) OR (Hemodialysis[Title/Abstract])) OR (Hemodialyses[Title/Abstract])) OR (Dialysis, Extracorporeal[Title/Abstract])) OR (Dialyses, Extracorporeal[Title/Abstract])) OR (Extracorporeal Dialyses[Title/Abstract])) OR (Extracorporeal Dialysis[Title/Abstract]))
#3 #1 AND #2	#3 #1 OR #2
#4 (Abstract: Randomized Controlled Trial) OR (Abstract: Randomized) OR (Abstract: RCT)	#4 ((((((((Exercise[Title/Abstract]) OR (Aerobic Exercise[Title/Abstract])) OR (Resistance Exercise[Title/Abstract])) OR (Physical Activity[Title/Abstract])) OR (Stretching[Title/Abstract])) OR (Cycling[Title/Abstract])) OR (physical fitness[Title/Abstract])) OR (exercise trainin[Title/Abstract]))
#5 #3 AND #4	#5 #3 AND #4 Filters:Randomized Controlled Trial, from 2010/1/1–2024/7/31

### 2.3 Inclusion criteria

(1) Study subjects: patients undergoing hemodialysis or peritoneal dialysis for a minimum of 3 months, exhibiting abnormal cardiac function with LVEF>45%, NYHA score<3, aged between ≥18 and ≤70 years. (2) Intervention measures: dialysis patients who did not engage in any exercise, while the intervention group participated in exercise without restrictions on the form of exercise performed. (3) Control measures: dialysis patients who did not perform any exercise. (4) Outcome indicators: the primary outcome indicator was LVEF, while the secondary outcome indicators included PWV, LVMI, LVESV, and LVEDV. The selected literature must include at least one primary or secondary outcome indicator. (5) Study type: randomized controlled trials.

### 2.4 Exclusion criteria

(1) Study subjects who are not dialysis patients and do not exhibit abnormal cardiac function. (2) Patients with severe cardiac, vascular, or respiratory diseases, such as unstable angina, valvular heart disease, acute myocardial ischemia, cardiovascular surgery (including valve replacement, coronary artery bypass grafting, and angioplasty), cancer, chronic obstructive pulmonary disease, and other conditions. (3) Intervention measures that do not involve exercise. (4) Literature published before 2010, literature from which valid outcome data cannot be extracted, duplicate publications, or literature with incomplete data. (5) Non-randomized controlled trials.

### 2.5 Literature screening and data extraction

**Figure 1:**
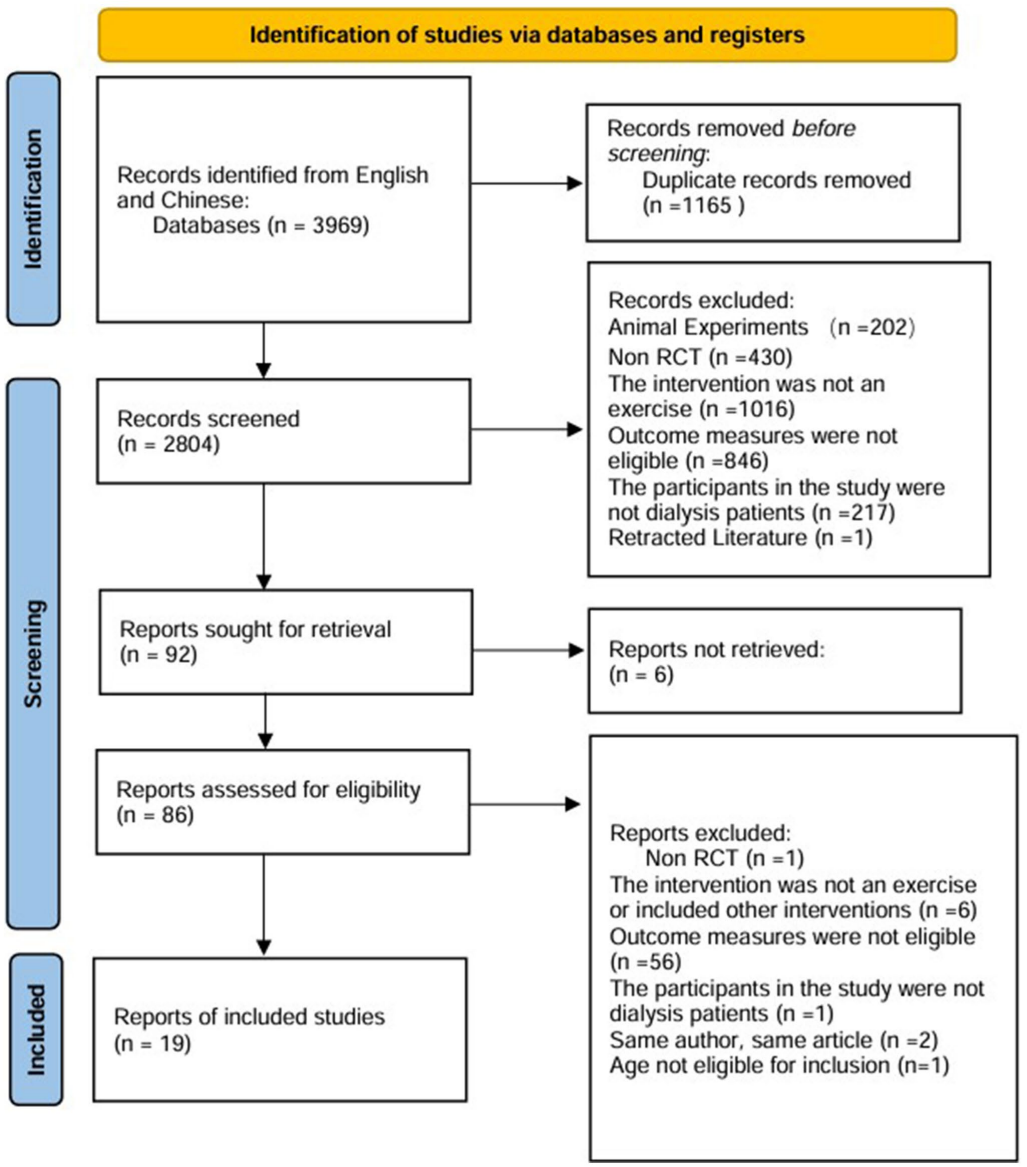
Schematic diagram of the literature inclusion process.

Two independent researchers (H.Z.L. and M.Q.C.) downloaded the results of the literature search and imported them into the literature management software note express. Using this software, duplicate papers were removed, and the titles and abstracts of all eligible papers were screened. For the papers that passed the initial screening, the full texts were obtained, while those for which the full text could not be retrieved were excluded. A comprehensive assessment was conducted based on the inclusion criteria, and studies that did not meet these criteria were excluded. Any disagreements were resolved by a third researcher (M.Z.). The entire process of selecting research data is presented in the form of a flowchart (Figure 1). The extraction of fundamental data from the included literature was conducted independently by two researchers (H.Z.L. and M.Q.C.) using Excel. Any disagreements were resolved by a third researcher (M.Z.). The extracted data primarily included the first author. For the papers that option, country of study, sample size, patient age, duration of dialysis, and intervention measures, which encompassed types of exercise, duration of exercise, frequency of exercise, intervention period, and exercise intensity.

The primary outcome measured in this study was LVEF, while the secondary outcomes included PWV, LVMI, LVESV, and LVEDV. All outcomes were assessed using echocardiography.

### 2.6 Literature quality assessment

In this study, two independent researchers (H.Z.L. and M.Q.C.) utilized the risk of bias tool developed by the Cochrane Collaboration to conduct a quality assessment. The assessment criteria included random allocation methods, allocation concealment, blinding of assessors, integrity of outcome data, selective reporting, and other potential biases.

In this study, the GRADE system was employed to assess the quality of evidence. The evaluation was conducted across five dimensions: reference to the risk of bias, inconsistency, indirectness, imprecision, and publication bias. The overall strength of the evidence was classified into four categories: high, moderate, low, and very low.

### 2.7 Statistical analysis

This article utilized RevMan Manager 5.4 to conduct a meta-analysis of more than two randomized controlled trials with identical outcome indicators. The mean difference (MD) was used as the effect measure, and its 95% confidence interval (CI) was calculated simultaneously. The *p*-value for the combined statistics was determined using the Z test, with P domized controlled trials with identical outcome indigeneity of the research data was assessed using the I^2^ statistic. If *p* > 0.1 and I^2^ < 50%, it indicated no heterogeneity or low heterogeneity, and the fixed-effect model was employed to calculate the combined effect size. Conversely, if *p* ≤ 0.1 and I^2^ ≥ 50%, it indicated high heterogeneity, prompting the use of the random-effects model to compute the combined effect size. In instances of significant heterogeneity, subgroup analyses were conducted. A sensitivity analysis was performed on the included literature by systematically removing one study at a time, comparing the changes in I^2^ values before and after each removal, and assessing the stability of the results. Publication bias was evaluated using a funnel plot. The quality of evidence was assessed using the Grading of Recommendations Assessment, Development, and Evaluation (GRADE) framework for pairwise meta-analysis.

## 3 Results

### 3.1 Literature retrieval process

We searched seven databases and retrieved a total of 3,969 relevant articles in both Chinese and English. We searched English databases: 212 from PubMed, 429 from Web of Science, 478 from the Cochrane Library. Additionally, we searched Chinese databases: 387 from CNKI, 1,568 from Wanfang, 306 from VIP, and 589 from SinoMed. After excluding duplicate articles, reviews, comments, and animal studies, 92 articles remained. Following the exclusion of those that did not meet the inclusion criteria and those for which the full text could not be obtained, a total of 19 articles were included.

### 3.2 Methodological quality

Quality evaluation using the Cochrane Risk of Bias tool revealed that only one literature met all evaluation criteria. The remaining literature failed to achieve allocation concealment. Only three literatures explicitly mentioned the implementation of blinding. In exercise trials, it is challenging to meet high-quality evaluation standards, and most studies find it difficult to implement double-blinding. Consequently, the literature is at some risk of bias. The specific literature retrieval process is illustrated in Figure 1.

### 3.3 Basic characteristics and quality evaluation of the included literature

**Figure 2:**
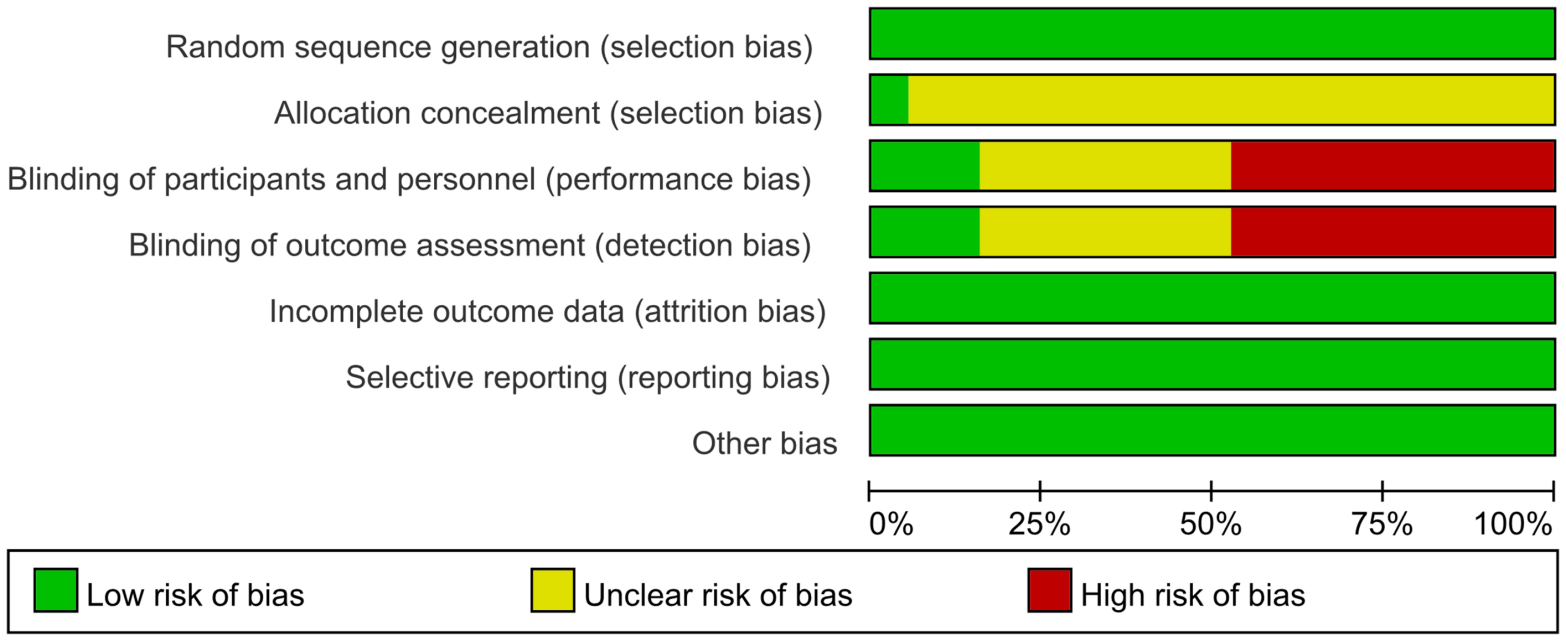
Risk of bias graph.

**Figure 3:**
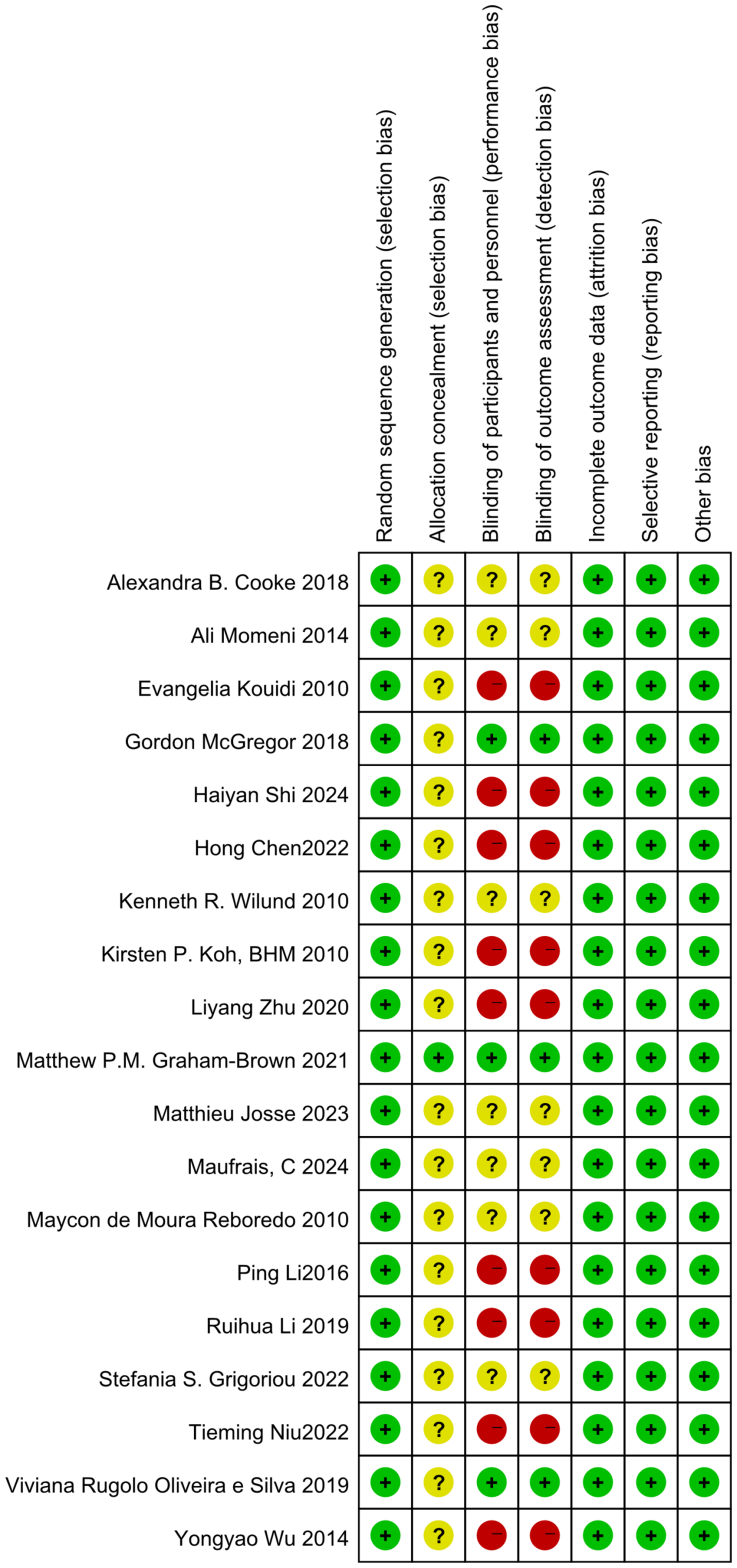
Risk of bias summary.

A total of 19 studies were included in this article, consisting of 16 randomized controlled trials, 3 randomized crossover trials, and 12 publications in English (14, 16–26). Additionally, there were 7 publications in Chinese (13, 27–32). The overall sample size comprised 1,088 individuals, with 615 participants in the intervention group and 610 in the control group. Three studies examined the effects of aerobic exercise combined with resistance training, one study focused solely on resistance training, and the remaining 15 studies investigated aerobic exercise alone. Additionally, one study specifically targeted patients undergoing peritoneal dialysis. Basic information regarding the included literature is presented in Table 2, while detailed quality evaluation tables for the literature are displayed in Figures 2, 3.

**Table 2:** Basic information of the included literature.

Authors and year	Country	Sample size (IG/CG)	Patient Age (years)	Dialysis duration (months)	CG	IG				
						IG	Duration of each exercise (minutes)	Exercise frequency (times per week)	Exercise intervention (weeks)	Exercise intensity (borg rating of perceived exertion scale)
Maufrais, C. 2024 (16)	France	56 (56/56)	63 ± 13	66 ± 68.4	HD	AE	30	3	NR	11–14
Matthieu Josse 2023 (17)	France	60 (60/60)	63 ± 14	68.4 ± 72	HD	AE	30	3	NR	11–14
Stefania S. Grigoriou 2022 (14)	Greece	21 (21/21)	56 ± 19	40 ± 44	HD	AE + RE	45	3	NR	14–16
Matthew P. M. Graham-Brown 2021 (18)	Britain	101 (51/50)	IG:55.5 ± 15.5 CG:58.9 ± 14.9	IG:1.2 ± 2.37 CG:1.3 ± 2.074	HD	AE	30	3	24	12–14
Viviana Rugolo Oliveira e Silva 2019 (19)	Brazil	30 (15/15)	IG:50 ± 17.2 CG:58 ± 15.0	IG:26.0 ± 14.58 CG:21.0 ± 27.1	HD	AE	30	3	16	13
Alexandra B. Cooke 2018 (20)	Canada	20 (10/10)	IG:58 ± 17 CG:53 ± 15	NR	HD	AE	42.6	3	16	12–16
Gordon McGregor 2018 (21)	Britain	34 (16/18)	IG:54.3 ± 12.22 CG:52.1 ± 11.63	IG:48.1 ± 32.44 CG:49.3 ± 29.19	HD	AE	60	3	10	12–14
Ali Momeni 2014 (22)	Iran	40 (20/20)	43.1 ± 10.5	NR	HD	AE	30	3	12	NR
Evangelia Kouidi 2010 (23)	Greece	44 (24/20)	IG:46.3 ± 11.2 CG:45.8 ± 10.8	IG:73.2 ± 55.2 CG:75.6 ± 58.8	HD	AE	60–90	3	48	11–13
Maycon de Moura Reboredo 2010 (24)	Brazil	22 (11/11)	IG:49.6 ± 10.6 CG:43.5 ± 12.8	IG:41.9 ± 42.4 CG:60.1 ± 54.4	HD	AE	60	3	12	4–6
Kirsten P. Koh, BHM 2010 (25)	Australia	49 (27/22)	IG:52.3 ± 10.9 CG:51.3 ± 14.4	30.5 ± 26.6	HD	AE	15–45	3	24	12–13
Kenneth R. Wilund 2010 (26)	America	17 (8/9)	IG:60.8 ± 3.2 CG:59.0 ± 4.9	IG:63.3 ± 8.7 CG:44.6 ± 12.2	HD	AE	45	3	24	12–14
Ping Li 2016 (31)	China	84 (41/43)	IG:49.8 ± 13.5 CG:53.4 ± 14.5	IG:37.8 ± 29.4 CG:35.7 ± 30.4	HD	AE	20–30	3–5	24	NR
Yongyao Wu 2014 (32)	China	69 (34/35)	IG:45.92 ± 8.64 CG:45.28 ± 8.16	IG:55.5 ± 37.3 CG:39.8 ± 29.7	HD	AE	10–15	3	12	12–16
Haiyan Shi 2024 (27)	China	80 (40/40)	IG;58.28 ± 9.72 CG:59.58 ± 9.12	IG:131.57 ± 63.77 CG:121.83 ± 40.56	HD	AE	30	3	24	NR
Liyang Zhu 2020 (29)	China	106 (53/53)	IG:46.8 ± 13.72 CG:47.4 ± 12.57	IG:13.41 ± 5.66 CG:14.87 ± 4.89	HD	AE + RE	10–20	4	12	11–12
Ruihua Li 2019 (30)	China	50 (25/25)	IG:48.8 ± 12.41 CG:49.1 ± 12.36	IG:34.02 ± 15.75 CG:33.79 ± 15.81	HD	AE	30	3	12	11–12
Tieming Niu 2022 (13)	China	60 (30/30)	IG:57.1 ± 8.7 CG:56.9 ± 9.1	IG:27.6 ± 25.2 CG:25.2 ± 26.4	PD	AE + RE	30–60	3	24	11–13
Hong Chen 2022 (28)	China	125 (63/62)	IG:41.96 ± 5.08 CG:42.75 ± 5.21	IG:25.11 ± 4.12 CG:24.33 ± 4.08	HD	RE	40	3	24	11–13

IG: Intervention Group; CG: Control Group; HD: Hemodialysis; PD: Peritonel Dialysis; AE: Aerobic Exercise; RE: Resistance Exercise; NR: Not Reported.

### 3.4 Primary outcomes

#### 3.4.1 LVEF

**Figure 4:**
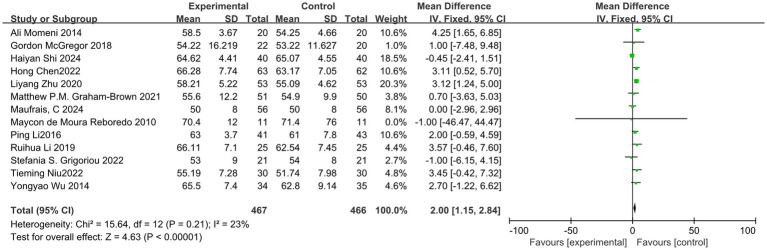
Forest plot of LVEF.

Among the 19 studies included in the analysis, 13 reported the outcome indicator of LVEF. Following a heterogeneity test, the I^2^ value was found to be 23%, which is less than 50%, and the Q test yielded a *p*-value of 0.21, indicating low heterogeneity among the studies. A fixed-effect model was employed to combine the effect sizes. Sensitivity analysis was conducted using the one-by-one exclusion method, and the results changed only slightly, suggesting that this study demonstrates good stability. The meta-analysis results indicated that exercise significantly improves LVEF in dialysis patients (MD = 2.00, 95%CI: 1.15–2.84, *p* < 0.001), as illustrated in Figure 4.

**Figure 5:**
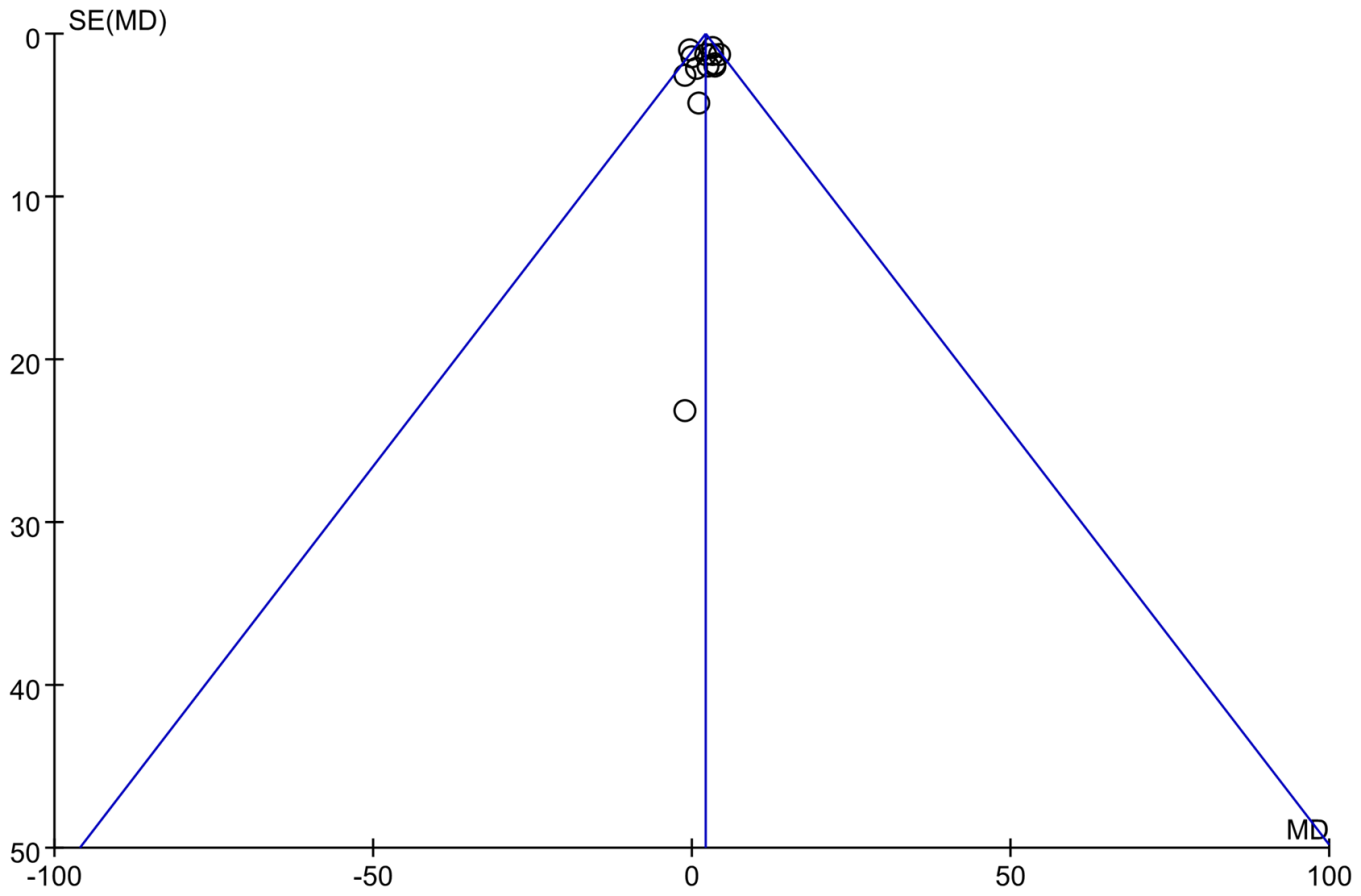
Funnel plot of LVEF.

The publication bias of this study was evaluated using a funnel plot. The results indicated that the distribution of points in the funnel plot was largely symmetrical, suggesting a low likelihood of publication bias that would not affect the analysis results, as shown in Figure 5. The overall certainty of this evidence was assessed to be low, as detailed in Table 3.

**Table 3:** GRADE assessment.

Outcomes	Study design	Risk of bias	Inconsistency	Indirectness	Imprecision	Other considerations	No. of participants		Absolute effect (95% CI)	Quality
							Experimental	Control		
LVEF	RCTs	Serious	Serious	No	No	No	467	466	MD 2.00 (1.15 to 2.84)	Low
LVMI	RCTs	Serious	Serious	No	Serious	No	104	105	MD -7.93 (−14.67 to −1.19)	Very low
PWV	RCTs	Serious	Serious	No	Serious	No	74	67	MD -0.48(−1.34 to 0.37)	Very low
LVEDV	RCTs	Serious	Serious	No	No	No	158	157	MD -0.51 (−2.41 to 1.40)	Low
LVESV	RCTs	Serious	Serious	No	Serious	No	51	51	MD -0.96 (−2.94 to 1.02)	Very low

RCTs, randomized controlled trials. MD, mean difference.

#### 3.4.2 LVMI

**Figure 6:**
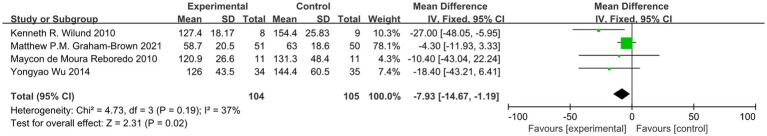
Forest plot of LVMI.

Among the included studies, four reported outcome indicators related to LVMI. After conducting a heterogeneity test, we found I^2^ = 37%, which is less than 50%, and *p* = 0.19, which is greater than 0.1, indicating low heterogeneity. Therefore, we employed a fixed-effect model to combine the effect sizes. The results of the sensitivity analysis indicated minimal variation, suggesting that this study possesses good stability. The meta-analysis results demonstrated that exercise significantly improves LVMI in dialysis patients (MD = −7.93, 95%CI: −14.67-1.19, *p* = 0.02), as illustrated in Figure 6.

**Figure 7:**
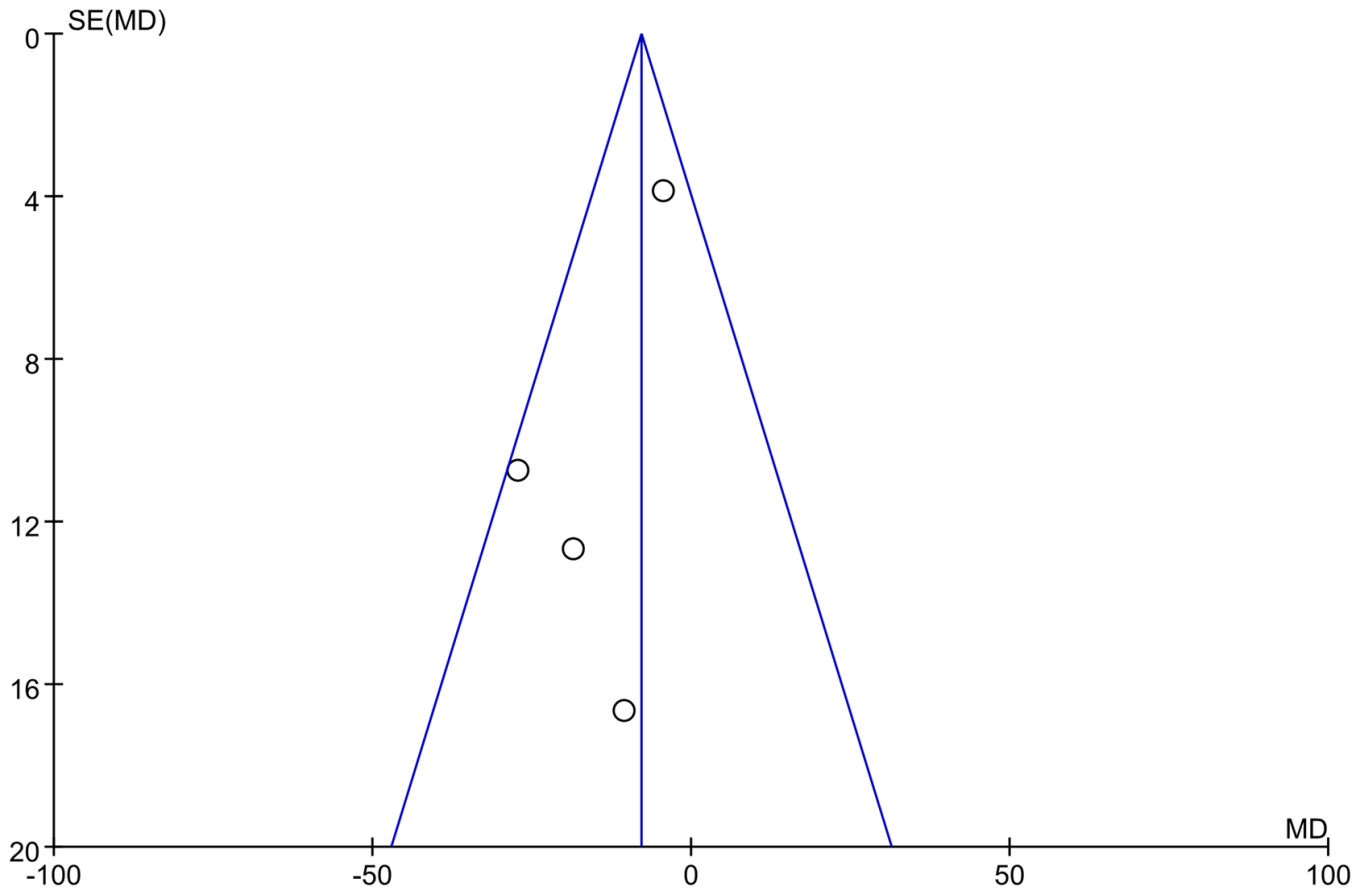
Funnel plot of LVMI.

To evaluate potential publication bias in this study, we constructed a funnel plot. The results demonstrated an asymmetric distribution of points within the funnel plot, indicating a possibility of publication bias, which may influence the findings of this study, as illustrated in Figure 7. The overall certainty of this evidence was assessed to be very low, as illustrated in Table 3.

#### 3.4.3 PWV, LVEDV, and LVESV

**Figure 8:**
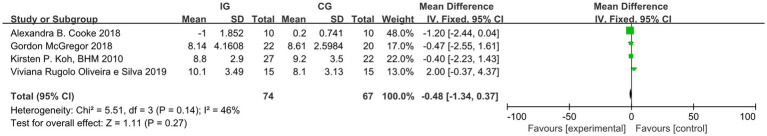
Forest plot of PWV.

**Figure 9:**
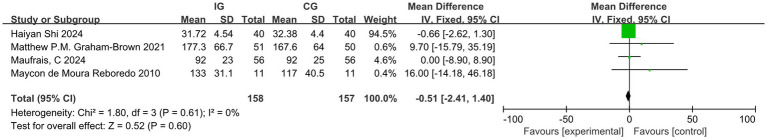
Forest plot of LVEDV.

**Figure 10:**
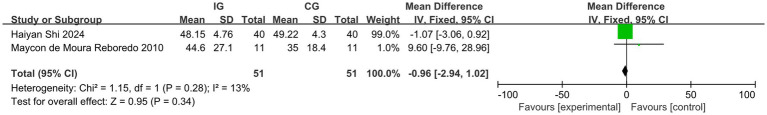
Forest plot of LVESV.

Among the studies included in the analysis, four reported the outcome indicator of pulse wave velocity (PWV). After conducting a heterogeneity test, we found I^2^ = 46%, which is less than 50%, and *p* = 0.14, which is greater than 0.1, indicating low heterogeneity. Therefore, we employed a fixed-effect model to combine the effect sizes. The results of the meta-analysis indicated a MD of −0.48 (95% CI, −1.34-0.37, *p* = 0.27), which was not statistically significant. Four studies reported the outcome indicator of LVEDV. After conducting a heterogeneity test, we found I^2^ = 0%, which is less than 50%, and *p* = 0.61, which is greater than 0.1, indicating low heterogeneity. Therefore, we employed a fixed-effect model to combine the effect sizes. The meta-analysis results showed an MD of −0.51 (95% CI, −2.41–1.40, *p* = 0.60), and this difference was not statistically significant. Two studies reported the outcome indicator of LVESV. After conducting a heterogeneity test, we found I^2^ = 13%, which is less than 50%, and *p* = 0.28, which is greater than 0.1, indicating low heterogeneity. Therefore, we employed a fixed-effect model to combine the effect sizes. The meta-analysis results indicated an MD of −0.96 (95% CI, −2.94–1.02, *p* = 0.34), with no statistically significant difference. This study cannot demonstrate that exercise improves the outcome indicators of PWV, LVEDV, and LVESV in dialysis patients, as illustrated in Figures 8–10. The overall quality of the evidence was judged as very low, low, and very low, respectively, as illustrated in Table 3.

#### 3.4.4 Subgroup analysis of LVEF

Among the included studies, nine utilized pure aerobic exercise, three combined aerobic exercise with resistance training, and only one employed pure resistance exercise (subgroup analysis could not be performed). The results indicated that both aerobic exercise and the combination of aerobic and resistance exercise significantly increased the LVEF in dialysis patients compared to the control group. The groups with workout lasts more than 30 min and less than 30 min, the intervention included groups with cycles longer than 12 weeks and those with cycles of 10 to 12 weeks, as well as groups with a weekly exercise frequency of more than three times per week and those with a frequency of three times per week or less, all demonstrated significant improvements in LVEF among dialysis patients. The differences observed were statistically significant. Please refer to Table 4 for further details.

**Table 4:** Subgroup analysis of the effect of exercise intervention on LVEF in dialysis patients.

Subgroup	Number of studies	Research subjects (cases)	Pooled effect size			Effect model	Heterogeneity test	
			MD [95%CI]	Z-value	*p*-value		I^2^-value	*p*-value
Exercise methods								
AE	9	536	1.14 [0.07,2.22]	2.08	0.04	fix	28%	0.19
AE + RE	3	187	2.78 [1.17,4.38]	3.39	<0.001	fix	13%	0.31
Duration of continuous exercise								
>30 min	5	262	2.50 [0.57,4.43]	2.53	0.01	fix	0%	0.67
≤30 min	8	586	1.88 [0.94,2.82]	3.92	<0.001	fix	46%	0.07
Exercise Intervention period								
>12 weeks	5	450	1.35 [0.13,2.56]	2.17	0.03	fix	38%	0.17
10–12 weeks	6	321	3.36 [2.04,4.68]	4.98	<0.001	fix	0%	0.97
Exercise frequency per week								
>3	2	190	2.73 [1.21,4.25]	3.53	<0.001	fix	0%	0.49
≤3	11	658	1.67 [0.56,2.68]	3.21	0.001	fix	28%	0.18

## 4 Discussion

The results of this meta-analysis on the effects of exercise interventions on cardiovascular outcomes in dialysis patients indicate that exercise training can significantly enhance several indicators of cardiovascular function, including LVEF and LVMI. However, it does not show statistical significance in improving PWV, LVESV, and LVEDV. The GRADE assessment of the quality of evidence rated the levels of evidence for LVMI, PWV, and LVESV as very low due to the limited quality of the included literature. It is essential to analyze the results of these studies with caution. However, the quality of evidence may be progressively improved in the future through rigorous methodological design, standardized interventions, technological innovations, and multidisciplinary collaboration.

A lifestyle devoid of physical activity is closely linked to the mortality rate among dialysis patients. Studies indicate that nearly 35% of these patients develop cardiovascular disease due to lack of engagement in physical activity (33). Although there are various methods available to treat cardiovascular events in dialysis patients, including pharmacological therapy and surgical interventions, significant financial resources are required, and the outcomes have not substantially improved the survival rates of these patients. On the other hand, exercise is a low-cost intervention that is highly applicable and generally well-accepted by patients. Recent research indicates that exercise has a significant positive impact on cardiovascular health (34, 35).

### 4.1 LVEF

LVEF is a crucial indicator of left ventricular systolic function, reflecting the heart’s pumping ability. It serves as an important measure for assessing the cardiovascular health of patients undergoing dialysis. Numerous studies have demonstrated that exercise during dialysis can significantly enhance LVEF in these patients (16, 17). The results of this study are consistent and robust. This research conducted subgroup analyses for various types of exercise. The findings indicated that both aerobic plus resistance exercise and simple aerobic exercise can improve the LVEF in dialysis patients, with aerobic plus resistance exercise demonstrating a superior effect compared to simple aerobic exercise. However, a limitation of this study is that the types of exercises examined were relatively limited, and the effects of simple resistance exercise were not compared. Nevertheless, many current studies indicate that personalized exercise regimens can be tailored for dialysis patients in various situations, resulting in different improvement effects across various indicators. For example, AE, respiratory muscle training (PMT), and RE + AE significantly enhance performance on the six-minute walk test (6MWT). AE and RE + AE are associated with a higher maximal oxygen consumption (VO2max) (36). Mind–body training (MBT) and RE + AE significantly improve blood pressure. Moreover, the benefits of MBT in reducing arterial blood pressure are unparalleled compared to other forms of exercise (37). It appears that varying durations of continuous exercise also influence the LVEF in dialysis patients. The study assessed the duration of individual exercise sessions, the length of the exercise intervention period, and the frequency of weekly exercise sessions. It can be concluded that for dialysis patients, the duration of each exercise sessions lasting longer than 30 min, an exercise intervention period of 10 to 12 weeks, and a frequency of more than three sessions per week are associated with greater improvements in LVEF. Due to the limited range of exercise intensities included in this trial, most of the literature employs a rating of perceived exertion between 11 and 16 (medium to hard) on the 6–20 Borg scale. Additionally, a small portion of the literature primarily utilizes 60 to 70% of the maximum heart rate (calculated as 220 minus age) during exercise, a comparison of dialysis intensity was not conducted. One piece of literature involving a study population of patients with Parkinson’s disease was included in this trial but could not be compared statistically. This literature was included in the LVEF study index. We performed a sensitivity analysis of this literature using an exclusion-by-exclusion approach, which resulted in only minor differences, suggesting that this study had a limited impact on the final results. However, due to the different mechanisms of action of HD and peritoneal dialysis, there are advantages and disadvantages to each method. Hemodialysis removes excess water and toxins from the body efficiently and quickly, but may adversely affect the heart, with the most common risk being hypotension. In contrast, peritoneal dialysis enhances water and sodium retention and helps prevent the development of heart failure associated with vascular access. However, patients undergoing peritoneal dialysis are also at increased risk of developing peritoneal dialysis-associated peritonitis. As a result, the choice of dialysis modality varies in its effectiveness in improving heart failure. Comparisons of different dialysis modalities can be made in the future.

### 4.2 LVMI

Left ventricular hypertrophy (LVH) can result from various factors, including hypertension, diabetes, anemia, hyperparathyroidism, and inflammatory conditions. LVMI is a critical criterion for diagnosing LVH. A study involving 153 participants, with a follow-up period exceeding 54 months, demonstrated that improvements in LVH outcome indicators positively influenced the survival rate of dialysis patients with cardiovascular disease (CVD). Specifically, for every 1 gram reduction in left ventricular mass, the cardiovascular risk decreased by 1% (38). A prospective study revealed that two echocardiographic examinations conducted 18 months apart showed a graded relationship between changes in LVMI and all-cause mortality, which was significantly associated with cardiovascular events (39). This study demonstrates that exercise can significantly improve the LVMI in dialysis patients, thereby reducing the incidence of cardiovascular events and enhancing their survival rates. Interestingly, among the four studies included, two indicated that exercise had no significant effect on improving LVMI, while the other two reported notable improvements. These discrepancies may be attributed to variations in exercise intensity and duration. Due to the limited number of studies included, a subgroup analysis could not be performed.

The outcome indicators of PWV, LVEDV, and LVESV did not show statistical significance. Consequently, it cannot be concluded that exercise improves PWV, LVEDV, and LVESV in dialysis patients. This lack of significance may be attributed to the limited literature included in this study. For LVESV, both studies analyzed indicated that exercise interventions were ineffective, which aligns with the findings of this study. Among the literature concerning PWV and LVEDV, one study showed no significant improvement in PWV, while two studies reported similar results for LVEDV. However, studies have confirmed that for patients with renal failure, every 1 m/s increase in PWV is associated with a 14% increase in the total cardiovascular mortality rate (40). PWV is closely related to hypertension, and an increasing number of studies have established that arteriosclerosis is an independent predictor of hypertension (20). Exercise can improve PWV by dilating blood vessels and enhancing endothelial function. Therefore, regarding the outcomes for PWV, LVEDV, and LVESV, a larger sample size is necessary, and this study should not be considered as evidence-based. In the literature reviewed, both the control and experimental groups reported related adverse events. The incidence of hypotension was relatively high, followed by nausea and vomiting, which may be linked to individual physical conditions or exercise intensity. Although no systematic statistical analysis was conducted in this study, however, future research should include additional statistical analyses regarding the incidence of adverse effects.

The safety of exercise during dialysis remains a significant concern. For example, it is unclear whether exercise may increase the workload on the heart, disrupt cardiac hemodynamics, and consequently elevate the risk of complications in dialysis patients. Additionally, there are questions about whether exercise in patients undergoing peritoneal dialysis could lead to catheter detachment, increased intra-abdominal pressure, or infection. Furthermore, it is important to consider whether medical staff are adequately trained in structured exercise protocols and can provide appropriate exercise recommendations. Although some studies have confirmed that a single session of moderate-intensity aerobic exercise performed during dialysis does not significantly alter hemodynamic parameters, such as blood pressure and cardiac output, further research is needed to fully understand the implications of exercise in this population (16). This confirms that this type of exercise is safe for dialysis patients. However, developing individualized exercise programs requires collaboration among a multidisciplinary team. Several studies have demonstrated that exercise during dialysis, under these conditions, is both safe and effective for patients (41, 42).

## 5 Conclusion

Our research findings indicate that exercise can significantly enhance LVEF and LVMI in patients undergoing dialysis who experience cardiac function issues. Subgroup analysis suggests that individualized exercise regimens can be tailored for these patients, incorporating both aerobic and resistance training. Effective regimens typically involve exercise sessions lasting more than 30 min, an intervention period of 10 to 12 weeks, and a frequency of more than three times per week. It is important to note that no statistically significant effects were observed for PWV, LVEDV, and LVESV. To enhance the robustness of the research, it may be necessary to increase the sample size and improve the quality of the included literature.

## 6 Study limitations and future prospects

This study has several significant limitations. Firstly, the quality of the included literature was subpar. Secondly, the types of exercise examined were relatively monotonous, as most studies focused solely on traditional aerobic exercises. The assessment of publication bias was conducted solely through funnel plots, and the limited number of included studies imposed certain constraints on the results. Future research should prioritize higher-quality articles and explore a broader range of exercise modalities, such as virtual reality exercise, yoga during dialysis, electrical muscle stimulation, and more. The outcome measures of the study could be expanded to include inflammatory factors, changes in blood pressure, and other relevant metrics. This would provide dialysis patients with more options to develop appropriate exercise programs. Additionally, the effects of exercise interventions on patients undergoing home peritoneal dialysis should also be considered.

## Data Availability

The datasets presented in this study can be found in online repositories. The names of the repository/repositories and accession number(s) can be found in the article/Supplementary material.
