# Supplementary material for: Meta-analysis of the effects of exercise interventions on dialysis patients with cardiac function disorders
Source: Front Med (Lausanne). 2025 May 13;12:1573498. doi: 10.3389/fmed.2025.1573498 (PMC12106465; doi:10.3389/fmed.2025.1573498)
Supplement: Supplementary file 3 [file Table_3.docx]

1. Fifty-six outcomes were considered ineligible for inclusion in the outcome measures^[1-36, 49-51, 53-54, 57-64, 66, 68-73]^.

2. In addition to exercise, the interventions also included other interventions, such as diet, in a total of five articles^[37-41]^.

3. The subjects of the study were non-renal dialysis patients, and 1 was published^[52]^.

4. For the same study conducted by the same author, literature published in 2021 was selected for inclusion, while two studies with the same title were excluded^[55, 56]^.

5. One article in the literature focused on dialysis as the intervention rather than exercise^[65]^.

6. One piece of literature did not meet the inclusion criteria^[67]^.

7. One article was not included in the randomized controlled clinical trial^[74].^

8. Six articles were not found^[75-80].^

9. Finally, seven Chinese articles were included^[42-48]^ and twelve articles were included in English ^[81-92]^

[1] Li Jianying, Huang Yanlin, Teng Yanjuan, et al. Effect of Exercise Training on symptomatic hypotension in uremic hemodialysis patients[J]. Primary Medicine Forum, 2012, 16(24): 3139-40.

[2] LIU Lixiu. Effect of exercise on quality of life of hemodialysis patients [J]. Chinese Journal of Primary Medicine, 2015, (17): 2657-9,60.

[3] Lv Liqiao. Effect of exercise during dialysis on symptomatic hypotension and dialysis adequacy [J]. Zhejiang Clinical Medicine, 2017, 19(05): 923-4.

[4] Wang Hui. Effect of low-intensity aerobic training combined with health education on morbidity and quality of life in patients with maintenance hemodialysis [J]. Nursing Practice and Research, 2017, 14(19): 31-3.

[5] Li Qianling, Wu Bijing, Li Guanmei, et al. Preventive effect of Reasonable Exercise Nursing on Hypotension in Patients Undergoing Maintenance Hemodialysis. Practice and Research, 2018, 15(07): 39-40.

[6] Li Xiaoqing, Guo Min, Lin Ying, et al. Effect of exercise intervention on symptomatic hypotension in dialysis patients [J]. Electronic Journal of Practical Clinical Nursing, 2018, 3(17): 32-5.

[7] Xiao Yu, Liu Jun, Xie Lizhen, et al. Effect of exercise therapy on hypotension in hemodialysis patients [J]. Chinese Journal of Contemporary Medicine, 2019, 26(11): 48-50.

[8] Yang Xue. Effect of exercise during dialysis on symptomatic hypotension and dialysis adequacy [J]. 健|康必read, 2019, (32): 230-1.

[9] Ceng Xiaoling, Li Xiaoqing, Xiao Qing. Nursing effect of exercise intervention on symptomatic hypotension in dialysis patients [J]. Chinese and Foreign Medical Research, 2020, 18(10): 59-61.

[10] PU Wei. Exercise therapy for the nursing intervention of hypotension in patients on maintenance dialysis [J]. Health Care Guide, 2020, (34): 139.

[11] Wang Yan. Effect of Combination of Dietary Care and Aerobic Exercise in Peritoneal Dialysis Patients [J]. China Health Nutrition, 2020, 30(23): 198-9.

[12] Xiong Min. Effect of individualized exercise regimen on maintaining prognosis and social regression in hemodialysis patients [J]. Contemporary Nurses, 2020, 27(12): 75-7.

[13] Cai Yanju, Chenxiao. Effect of resistance exercise intervention on fatigue and physical activity level in patients undergoing maintenance hemodialysis [J]. International Journal of Nursing, 2021, 40(20): 3734-8.

[14] LI Yucui. Effect of exercise intervention in dialysis based on the knowledge-belief-action model on physical function in hemodialysis patients [D]; University of South China, N1 - Cheng Peiyu, 2021.

[15] Song Ya. Effect of reasonable exercise nursing on the prevention of hypotension in patients on maintenance hemodialysis [J]. Pharmacy Weekly, 2021, 30(37): 113-4.

[16] Wang Chunrong, Liang Caihong, Chang Shengtao, et al. Effect of planned aerobic exercise-resistance exercise intervention on oxidative stress in patients on maintenance hemodialysis [J]. Smart Health, 2021, 7(31): 157-9.

[17] Wu Menghan, Wang Yan, Ai Shuanglan, et al. Effect of exercise on improving cardiopulmonary function and psychological state in patients undergoing hemodialysis [J]. Chinese Journal of Medical Frontiers, 2021, 13(8): 67-70.

[18] Yao Lan. Preventive effect of reasonable exercise care on hypotension in patients on maintenance hemodialysis [J]. Oriental Medicinal Diet, 2021, (15): 185.

[19] Yao Li. Effect of exercise nursing intervention on cardiopulmonary function and psychological state in patients undergoing maintenance hemodialysis [J]. Integrative Medicine Nursing, 2021, 7(8): 151-3.

[20] Yuan Xiazhi, Hua Ying, Fang Jinqiong, et al. Application of recumbent bicycle exercise in preventing hypotension in hemodialysis patients [J]. Nursing and Rehabilitation, 2021, 20(2): 1-4.

[21] Huang Dejian, Dong Dabao, Tan Dandan, et al. Effect of CPET-guided aerobic exercise intervention on cardiopulmonary function in peritoneal dialysis patients with CKD [J]. Chinese Journal of Border Health and Quarantine, 2022, 45(S01): 101-2+8.

[22] Li Bai, Jiao Tianjie. Efficacy of exercise therapy on dialysis hypotension and restless legs syndrome in uremia patients and its effect on dialysis adequacy [J]. International Journal of Transplantation and Blood Purification, 2022, 20(4): 6-9.

[23] Li Jiaming, Zhang Yixin, Xing Lingli, et al. Application of individualized exercise program in improving sleep quality, quality of life, and blood pressure control in patients undergoing maintenance hemodialysis [J]. Chinese Science and Technology Journals Database (Full Text Edition) Medicine and Health, 2022, (8): 0033-6.

[24] Liu Fang, Wu Han, Zhang Yingying, et al. Application of Incremental Resistance Exercise Training Combined with WeChat Health Education Platform in the Care of Patients Undergoing Maintenance Hemodialysis with Chronic Renal Failure [J]. China Herald of Medicine, 2022, 19(3): 162-5,81.

[25] Liu Lijie. Effect of moderate aerobic exercise in patients with uremia maintenance hemodialysis [J]. Sports Illustrated, 2022, (8): 118-9,21.

[26] Su Xiaolian, Ai Lingyan, Duan Yinfeng, et al. Effects of aerobic exercise on resting energy metabolism, PEW status and cardiorespiratory endurance in maintenance hemodialysis patients[J]. Dialysis and Artificial Organs, 2022, 33(02): 96-101.

[27] Chenghui Tao, Yang Liu, Jialu Ran, et al. Application value of cardiac rehabilitation exercise in patients with uremia maintenance hemodialysis [J]. A must-read for health, 2022, (34): 27-8.

[28] Zhang Wei, Wang Hui, Huan Shelley, et al. Study on the occurrence of IDH in hemodialysis patients by aerobic exercise exercise during dialysis [J]. Electronic Journal of Practical Clinical Nursing, 2022, 7(38): 60-2,59.

[29] Zhang Yayi, Li Chunxi, Huang Xiaoling, et al. Effect of exercise intervention during hemodialysis in the prevention of symptomatic hypotension [J]. Primary Medicine Forum, 2022, 26(21): 1-3.

[30] He Yanping. Effect of intermittent exercise care during dialysis on the incidence of hypotension and quality of life in patients undergoing maintenance hemodialysis [J]. Science Consulting (Science & Technology Management), 2023, (04): 142-4.

[31] Lou Li, Chen Chen, Jiang Chunlei, et al. Efficacy of exercise therapy on dialysis-associated hypotension in patients [J]. Chinese Science and Technology Journals Database (Full Text Edition) Medicine and Health, 2023, (2): 0086-8.

[32] Tan Yixiang, Azatijaynasi, Wang Xiaoqin, et al. Effect of multimodal exercise training on volume load and nutritional status in patients on continuous ambulatory peritoneal dialysis [J]. Chinese Science and Technology Journals Database (Full Text Edition) Medicine and Health, 2023, (11): 0027-32.

[33] Li Yanyang, Quan Minying. Effect of cycling exercise combined with nutritional intervention on complications in patients on maintenance hemodialysis [J]. Heilongjiang Medicine, 2024, 37(03): 682-4.

[34] Su Xiaoming, Yang Hongfei, Yu Meihua, et al. Application of low-intensity exercise of yoga ball in patients with maintenance hemodialysis [J]. Chinese Science and Technology Journals Database (Full Text Edition) Medicine and Health, 2024, (1): 0040-3.

[35] Wu Fen. Effect of Personalized Rehabilitation Exercise Training Based on Cardiopulmonary Exercise Test in Maintenance Hemodialysis Patients [J]. Primary Medicine Forum, 2024, 28(9): 70-2.

[36] Xu Xiuxiu, Qin Wenting, Su Xiaoxuan, et al. Application of high-intensity interval exercise in patients on maintenance hemodialysis [J]. Hebei Medicine, 2024, 46(9): 1315-9.

[37] Wang Li, Zhou Meimei, Wang Jing, et al. Effect of dietary management combined with aerobic exercise on volume load and cardiac function in patients undergoing maintenance peritoneal dialysis [J]. Chinese Journal of Practical Nursing, 2021, 37(36): 2813-8.

[38] Chen Huiping, Kong Xiaoli, Chen Ning, et al. Effect of Baduanjin Exercise Combined with Traditional Chinese Medicine Diet Intervention on Volume Load, Nutrition and Fatigue in Patients on Maintenance Peritoneal Dialysis [J]. Qilu Journal of Nursing, 2023, 29(17): 57-60.

[39] Zhuang Peixia. Effect of continuous health guidance on blood pressure and quality of life in patients on maintenance hemodialysis and hypertension [J]. Knowledge of Prevention and Treatment of Cardiovascular Diseases, 2023, 13(19): 35-7.

[40] Hu Yaqian. Effect of levocarnitine combined with resistance exercise training on hypotension in hemodialysis patients [J]. Medical Theory & Practice, 2023, 36(24): 4308-9,18.

[41] Chen Xin. Effect of levocarnitine combined with intermittent exercise therapy on dialysis with hypotension in uremia patients [J]. Medical Theory & Practice, 2024, 37(11): 1964-5+70.

[42]  Niu Tieming, Luan Xunfei, Dong Qingze, et al. Effects of aerobic exercise combined with resistance training on motor function and factors related to cardiovascular events in peritoneal dialysis patients [J]. Chinese Journal of Physical Medicine and Rehabilitation. 2022, 44(6): 540-542.

[43]  Shi Haiyan, Zang Xiujuan, Chen Peng, et al. Effects of quantitative aerobic exercise under the guidance of cardiopulmonary exercise test on cardiopulmonary function and quality of life in hemodialysis patients [J]. Chinese Journal of Integrated Traditional and Western Nephrology. 2024, 25(02): 142-145.

[44]  Chen Hong, Zhou Wenlei, Zhang Hong. Effects of incremental resistance exercise training during dialysis on dialysis quality and psychological status of middle-aged maintenance hemodialysis patients [J]. International Journal of Transplantation and Hemopurification. 2022, 20(2): 46-48.

[45]  Zhu Liyang, Lu Meisu, Wang Honglin, et al. Effects of planned aerobic-resistance exercise during dialysis intervals on nutritional status and dialysis hypotension of patients [J]. Chinese Journal of Modern Nursing. 2020, 26(14): 1894-1898.

[46]  Li Ruihua, Chen Huiling, Wu Xueyu. Effects of cycling training on laboratory objective indicators of uremic hemodialysis patients [J]. Journal of Guangzhou Medical University. 2019, 47(02): 116-118.

[47]  Li Ping, Wang Donghong. Effects of aerobic exercise on 6-minute walking distance and omentin-1 in hemodialysis patients [J]. Tianjin Medical Journal. 2016, 44(08): 1014-1017.

[48]  Wu Yongyao, Xia Min, Cao Shengsheng, et al. Effects and safety of individualized exercise therapy during hemodialysis treatment on cardiac function and exercise capacity of uremic patients [J]. Chinese Journal of Blood Purification. 2014, 13(08): 580-584.

[49] CHEN P Y, HUANG Y C, KAO Y H, et al. Effects of an Exercise Program on Blood Biochemical Values and Exercise Stage of Chronic Kidney Disease Patients [J]. JOURNAL OF NURSING RESEARCH, 2010, 18(2): 98-107.

[50] ORCY R B, DIAS P S, SEUS T L, et al. Combined resistance and aerobic exercise is better than resistance training alone to improve functional performance of haemodialysis patients--results of a randomized controlled trial [J]. Physiotherapy research international, 2012, 17(4): 235‐43.

[51] DE LIMA M C, CICOTOSTE CDE L, CARDOSO KDA S, et al. Effect of exercise performed during hemodialysis: strength versus aerobic [J]. Renal failure, 2013, 35(5): 697‐704.

[52] GREENWOOD S A, KOUFAKI P, MERCER T H, et al. Effect of exercise training on estimated GFR, vascular health, and cardiorespiratory fitness in patients with CKD: a pilot randomized controlled trial [J]. American journal of kidney diseases, 2015, 65(3): 425‐34.

[53] MITSIOU M, KOUIDI E J, LIAKOPOULOS V, et al. Effects of Music and Exercise during Hemodialysis on the Cardiac Autonomic Nervous System Activity [J]. Journal of the American Society of Nephrology : JASN, 2015, 26: 303A.

[54] XINGJUAN T, KA YEE CHOW S, KAM YUET WONG F. A nurse-led case management program on home exercise training for hemodialysis patients: a randomized controlled trial [J]. International journal of nursing studies, 2015, 52(6): 1029‐41.

[55] GRAHAM-BROWN M P, MARCH D S, CHURCHWARD D R, et al. Design and methods of CYCLE-HD: improving cardiovascular health in patients with end stage renal disease using a structured programme of exercise: a randomised control trial [J]. BMC Nephrol, 2016, 17(1): 69.

[56] GRAHAM-BROWN M P, MARCH D S, HULL K L, et al. The effects of a 6-month structured programme of intradialytic cycling on cardiovascular remodelling, myocardial fibrosis, and aortic stiffness: results from the cycle-HD study [J]. Journal of the American Society of Nephrology : JASN, 2019, 30: 96.

[57] KUKI A, TANAKA K, KUSHIYAMA A, et al. Association of gait speed and grip strength with risk of cardiovascular events in patients on haemodialysis: a prospective study [J]. BMC NEPHROLOGY, 2019, 20.

[58] CLARKSON M J, FRASER S F, BENNETT P N, et al. Efficacy of blood flow restriction exercise during dialysis for end stage kidney disease patients: protocol of a randomised controlled trial [J]. BMC nephrology, 2017, 18(1): 294.

[59] HEADLEY S, GERMAIN M, WOOD R, et al. Blood pressure response to acute and chronic exercise in chronic kidney disease [J]. Nephrology (Carlton, Vic), 2017, 22(1): 72‐8.

[60] TSAI Y C, CHEN H M, HSIAO S M, et al. Association of physical activity with cardiovascular and renal outcomes and quality of life in chronic kidney disease [J]. PLOS ONE, 2017, 12(8).

[61] ZHANG L, LUO H M, KANG G P, et al. The association between physical activity and mortality among patients undergoing maintenance hemodialysis [J]. INTERNATIONAL JOURNAL OF NURSING PRACTICE, 2017, 23(1).

[62] HALL R K, MCADAMS-DEMARCO M A. Breaking the cycle of functional decline in older dialysis patients [J]. SEMINARS IN DIALYSIS, 2018, 31(5): 462-7.

[63] JAYASEELAN G, WANG W, BENNETT P N, et al. Exercise Benefits and Barriers: The Perceptions of People Receiving Hemodialysis [J]. NEPHROLOGY NURSING JOURNAL, 2018, 45(2): 185-+.

[64] SUZUKI T, IKEDA M, MINAMI M, et al. Beneficial Effect of Intradialytic Electrical Muscle Stimulation in Hemodialysis Patients: a Randomized Controlled Trial [J]. Artificial organs, 2018, 42(9): 899‐910.

[65] PECOITS-FILHO R, LARKIN J W, POLI-DE-FIGUEIREDO C E, et al. Design and methodology of the impact of HemoDiaFIlTration on physical activity and self-reported outcomes: a randomized controlled trial (HDFIT trial) in Brazil [J]. BMC Nephrol, 2019, 20(1): 98.

[66] ROSSUM K F, THOMPSON S E, HANCOCK E K, et al. Timing of intradialytic exercise and its impact on intradialytic hypotension: a randomized crossover study [J]. Journal of the American Society of Nephrology : JASN, 2019, 30: 96.

[67] SHROFF R, KARABAY BAYAZIT A, STEFANIDIS C J, et al. SAT-040 HAEMODIAFILTRATION IMPROVES THE CARDIOVASCULAR RISK PROFILE AND PATIENT RELATED OUTCOME MEASURES COMPARED TO CONVENTIONAL HAEMODIALYSIS IN CHILDREN – THE HDF, HEART AND HEIGHT (3H) STUDY [J]. Kidney international reports, 2019, 4(7): S20‐S1.

[68] CHENG Y J, ZHAO X J, ZENG W, et al. Effect of Intradialytic Exercise on Physical Performance and Cardiovascular Risk Factors in Patients Receiving Maintenance Hemodialysis: a Pilot and Feasibility Study [J]. Blood purification, 2020, 49(4): 409‐18.

[69] CORREA H L, NEVES R V P, DEUS L A, et al. Could sestrins 2 be the secret of resistance exercise benefiting dialytic patients? [J]. Nephrology, dialysis, transplantation, 2020, 35(12): 2198‐9.

[70] MALLAMACI F, ANTONIO PANUCCIO V, VERSACE M C, et al. Efficacy of physical exercise in peritoneal dialysis patients: a secondary analysis of the excite trial [J]. Nephrology dialysis transplantation, 2021, 36(SUPPL 1): i469.

[71] ANDRADE F P, NOLASCO T, KNORST M M, et al. Aerobic Exercise Increases Vascular Diameter of Arteriovenous Fistula in Hemodialysis Patients [J]. Blood purification, 2022, 51(9): 732‐8.

[72] TASKAYA C, BUYUKTURAN B, BUYUKTURAN O, et al. Comparison of the efficacy of intradialytic core stabilization and aerobic exercises for hemodialysis patients: randomized controlled single-blind study [J]. Disability and rehabilitation, 2024: 1‐10.

[73] WILSCHUT E D, DE WINTER E P, BOS E J, et al. Supervised Pre-Operative Forearm Exercise to Increase Blood Vessel Diameter in Haemodialysis Patients: The PINCH Trial [J]. Eur J Vasc Endovasc Surg, 2024, 67(5): 852-3.

[74] ARROYO E, UMUKORO P E, BURNEY H N, et al. Initiation of Dialysis Is Associated With Impaired Cardiovascular Functional Capacity [J]. J Am Heart Assoc, 2022, 11(14): e025656.

[75] MORA G J A, GARCIA-GARCIA G, BRAMBILA D M, et al. Effect of physical training on echocardiographic parameters during hemodialysis: a randomized clinical trial [J]. Journal of the American Society of Nephrology : JASN, 2017, 28: 895.

[76] TOMLINSON C, CHURCHWARD D, GRANTHAM C, et al. A six month programme of intradialytic exercise improves resting heart rate in haemodialysis patients [J]. Nephrology dialysis transplantation, 2017, 32: iii658‐iii9.

[77] CHO J H, KIM J C. Effect of intradialytic exercise on physical performance and echocardiographic findings in maintenance hemodialysis patients [J]. Journal of the American Society of Nephrology : JASN, 2018, 29: 99.

[78] KIM J C, KIM S H, KANG S H, et al. The effect of intradialytic exercise on physical performance and echocardiographic findings in maintenance hemodialysis patients [J]. Journal of cachexia, sarcopenia and muscle, 2018, 9(6): 1131.

[79] CHANG H H, LAI Y H, HSU B G. Intradialytic Cycling Exercise Improves Arterial Stiffness in Hemodialysis Patients [J]. Journal of the American Society of Nephrology : JASN, 2023, 34: 82.

[80] HUANG M, AILI L V, HONGHONG L V, et al. THE EFFECT OF INTRADIALYTIC EXERCISE ON CARDIAC OUTCOMES IN HEMODIALYSIS PATIENTS [J]. Nephrology dialysis transplantation, 2023, 38: i755.

[81]  Grigoriou S S, Giannaki C D, George K, et al. A single bout of hybrid intradialytic exercise did not affect left-ventricular function in exercise-naïve dialysis patients: a randomized, cross-over trial[J]. Int Urol Nephrol. 2022, 54(1): 201-208.

[82]  Maufrais C, Josse M, Patrier L, et al. Cardioprotective effect of intradialytic exercise on left atrial mechanics[J]. Am J Physiol Renal Physiol. 2024, 326(5): F694-F703.

[83]  Josse M, Patrier L, Isnard M, et al. Cardioprotective Effect of Acute Intradialytic Exercise: A Comprehensive Speckle-Tracking Echocardiography Analysis[J]. JOURNAL OF THE AMERICAN SOCIETY OF NEPHROLOGY. 2023, 34(8): 1445-1455.

[84]  Graham-Brown M, March D S, Young R, et al. A randomized controlled trial to investigate the effects of intra-dialytic cycling on left ventricular mass[J]. KIDNEY INTERNATIONAL. 2021, 99(6): 1478-1486.

[85]  Silva V, Belik F S, Hueb J C, et al. Aerobic Exercise Training and Nontraditional Cardiovascular Risk Factors in Hemodialysis Patients: Results from a Prospective Randomized Trial[J]. CARDIORENAL MEDICINE. 2019, 9(6): 391-399.

[86]  Cooke A B, Ta V, Iqbal S, et al. The Impact of Intradialytic Pedaling Exercise on Arterial Stiffness: A Pilot Randomized Controlled Trial in a Hemodialysis Population[J]. AMERICAN JOURNAL OF HYPERTENSION. 2018, 31(4): 458-466.

[87] Mcgregor G, Ennis S, Powell R, et al. Feasibility and effects of intra-dialytic low-frequency electrical muscle stimulation and cycle training: A pilot randomized controlled trial[J]. PLOS ONE. 2018, 13(7).

[88]  Momeni A, Nematolahi A, Nasr M. Effect of intradialytic exercise on echocardiographic findings in hemodialysis patients[J]. Iran J Kidney Dis. 2014, 8(3): 207-211.

[89]  Kouidi E, Karagiannis V, Grekas D, et al. Depression, heart rate variability, and exercise training in dialysis patients[J]. Eur J Cardiovasc Prev Rehabil. 2010, 17(2): 160-167.

[90]  Reboredo M M, Pinheiro B V, Neder J A, et al. Effects of aerobic training during hemodialysis on heart rate variability and left ventricular function in end-stage renal disease patients[J]. J Bras Nefrol. 2010, 32(4): 367-373.

[91] Koh K P, Fassett R G, Sharman J E, et al. Effect of intradialytic versus home-based aerobic exercise training on physical function and vascular parameters in hemodialysis patients: a randomized pilot study[J]. Am J Kidney Dis. 2010, 55(1): 88-99.

[92]  Wilund K R, Tomayko E J, Wu P T, et al. Intradialytic exercise training reduces oxidative stress and epicardial fat: a pilot study[J]. 2010, 25(8): 2695-2701.
