# Supplementary material for: Meta-analysis of the effects of exercise interventions on dialysis patients with cardiac function disorders
Source: Front Med (Lausanne). 2025 May 13;12:1573498. doi: 10.3389/fmed.2025.1573498 (PMC12106465; doi:10.3389/fmed.2025.1573498)
Supplement: Supplementary file 4 [file Table_4.docx]

**Studies identified in the literature search**

**Total articles screened = 3969**

1 Fang Jianqiao, Liang Yi, Wang Cunxin, et al. Effects of transcutaneous acupoint electrical stimulation on plasma amino acids and extracellular fluid 5-Ht content in the dorsal nucleus of the middle suture of rats running on a platform [J]. Chinese Journal of Sports Medicine, 2010, 29(3): 309-12+20.

2 Tang Aidang, Lin Hongchu, Jiang Jie. Effectiveness of aerobic exercise combined with dietary intervention in controlling volume load in peritoneal dialysis patients [J]. Clinical Nursing Journal, 2010, 9(4): 15-7.

3 Tang Dongxing, Zeng Gaofeng. Effects of exercise intervention on the quality of survival of maintenance hemodialysis patients [J]. Journal of Xiangnan College (Medical Edition), 2010, 12(3): 18-20.

4 Yu Zu-Mei, Wen Xiu-Fang, Zhu Yong-Mei, et al. Application of exercise relearning method in exercise training for hemodialysis patients [J]. Journal of Nursing (Comprehensive Edition), 2010, 25(10): 79-81.

5 Liang Yanping, Xiao Cuiyan, Hou Yongmei. Effects of exercise therapy on sleep quality of maintenance hemodialysis patients [J]. Journal of Guangdong Medical College, 2011, 29(6): 618-20.

6 Huang Ailing, Zhang Suzhen, Song Fen. Effects of aerobic exercise on fatigue in peritoneal dialysis patients [J]. Jilin Medical Science, 2012, 33(6): 1149-50.

7 Li Jianying, Huang Yanlin, Teng Yanjuan. Effects of exercise training on symptomatic hypotension in uremic hemodialysis patients [J]. Primary Medical Forum, 2012, 16(24): 3139-40.

8 Huang Yanlin, Li Jianying, Teng Yanjuan, et al. Effect of exercise therapy on dialysis adequacy in uremic hemodialysis patients [J]. Journal of Nurse Advancement, 2013, 28(7): 587-8.

9 Li Jianying, Huang Yanlin, Teng Yanjuan, et al. Effects of exercise training on fatigue in uremic hemodialysis patients [J]. Journal of Guangxi Medical University, 2013, 30(6): 981-2.

10 Li Jianying, Huang Yanlin, Teng Yanjuan, et al. Effects of exercise training on lower limb motor function in uremic hemodialysis patients [J]. Chinese Journal of Modern Nursing, 2013, 19(15): 1749-50.

11 Qiu Shoutao, Zhang Yunkun. Changes of Glu and GABA contents in striatal microdialysis fluid of rats during the recovery period of re-exercise [J]. Sports Research, 2013, 34(3): 41-5.

12 Rong Xiangjiang, Zhou Jun, Zhang Yuqin, et al. Effects of preexercise on prefrontal cortex ascorbic acid level and recovery of learning memory ability in cerebral ischemic rats [J]. Chinese Journal of Physical Medicine and Rehabilitation, 2013, 35(7): 519-22.

13 Zhang Nianyun, Zhang Yunkun. Effects of thermal environment on monoamine neurotransmitters in the extracellular fluid of hypothalamus of rats recovering from exhaustive swimming [J]. Sports Research, 2013, 34(3): 50-4.

14 Zhang Xufang, Cheng Shubi, Han Xiaowei, et al. Application of home aerobic exercise in hemodialysis patients [J]. General Practice Nursing, 2013, 11(10): 903-5.

15 Hu Leibin, Weng Yaping, Liu Chunbo, et al. Study on the effect of aerobic exercise on calcium and phosphorus metabolism levels in hemodialysis patients [J]. Modern Practical Medicine, 2014, 26(12): 1545-6.

16 Huang Yanlin, Li Jianying, Teng Yanjuan, et al. Effect of exercise training on sleep quality of uremic hemodialysis patients [J]. Chinese Journal of Modern Nursing, 2014, 20(15): 1753-5.

17 Lu Yao. Evaluation of the application effect of exercise therapy in maintenance hemodialysis patients [J]. Nursing Practice and Research, 2014, 11(5): 43-5.

18 Wang Juan, Huang Yanlin, Wu Zhuomei, et al. Effects of exercise training on anxiety and depression in uremic hemodialysis patients [J]. Guangxi Medicine, 2014, 36(9): 1225-7.

19 Wu Yongyao, Xia Min, Cao Shengsheng, et al. Observations on the effects and safety of individualized exercise therapy on cardiac function and exercise capacity of uremic patients during hemodialysis treatment [J]. China Blood Purification, 2014, 13(8): 580-4.

20 Dai Ling. Effect of exercise therapy on sleep quality of maintenance hemodialysis patients [J]. Medical Theory and Practice, 2015, 28(23): 3288-90.

21 Ding Xiang. Effects of home aerobic exercise on fatigue and sleep quality in maintenance hemodialysis patients [J]. Contemporary Nurses (Lower Decade), 2015, 22(3): 126-8.

22 Fan Lingling. Intervention effect of aerobic exercise on fatigue status of maintenance hemodialysis patients [J]. Chinese and Foreign Medical Research, 2015, 13(15): 105-6.

23 He Hangying, Jin Zhiping, Ma Shuanglian, et al. Exercise training improves physical and mental status of maintenance hemodialysis patients [J]. PLA Nursing Journal, 2015, 32(8): 1-4.

24 Huang Liu, Huang Yanlin, Li Jianying, et al. Effects of aerobic exercise on lower limb motor function and fatigue in maintenance hemodialysis patients [J]. Guangxi Medicine, 2015, 37(4): 476-8+84.

25 Liu Lixiu. The effect of exercise on the quality of life of hemodialysis patients [J]. Chinese Primary Medicine, 2015, 22(17): 2657-60.

26 Lu Ailing, Lu Baoquan, Wang Jing, et al. Psychological care combined with exercise therapy in maintenance dialysis patients with malnutrition [J]. China Medical Innovation, 2015, 12(6): 72-4.

27 Rong Shuguang. Analyzing the value orientation of popular sports from the cultural consumption of sports shoes [J]. Sports and Science, 2015, 36(3): 79-86.

28 Zhang Hongmei, Fan Ruyan, Chang Liyang, et al. Effects of Tai Chi exercise on physical fitness and fatigue of hemodialysis patients [J]. Chinese Journal of Integrative Nephrology, 2015, 16(9): 807-10.

29 Fan Ruyan, Zhang Hongmei, Chang Liyang. Effects of taijiquan exercise on anxiety and depression status and sleep quality of hemodialysis patients [J]. China Blood Purification, 2016, 15(4): 241-3.

30 Guo Jiaquan, Wang Jinhong. Effect of aerobic training on cellular immune function after hemodialysis in ckd stage 5 patients [J]. Chinese Experimental Diagnostics, 2016, 20(12): 2047-9.

31 Huang Ruo, Lin Xiaomin, Lin Xiaoxia, et al. Effects of low-moderate aerobic exercise on fatigue and sleep quality in maintenance hemodialysis patients [J]. Chinese Primary Medicine, 2016, 23(23): 3569-72.

32 Jiao Yaxing. Intervention effect of aerobic exercise on fatigue status of maintenance hemodialysis patients [J]. Nursing Practice and Research, 2016, 13(2): 46-7.

33 Li P, Wang DH. Effects of aerobic exercise on 6-minute walking distance and hemopexin-1 in hemodialysis patients [J]. Tianjin Medicine, 2016, 44(8): 1014-7.

34 Wang Yichun. Effects of aerobic exercise workout on mood, sleep and muscle strength in uremic hemodialysis patients [J]. General Practice Nursing, 2016, 14(33): 3526-7.

35 Yan Erping, Qiu Moyan, Ren Jianwei, et al. The effect of evidence-based moxibustion regimen on musculomotor rehabilitation in maintenance hemodialysis patients [J]. Journal of Rehabilitation, 2016, 26(4): 6-10+6.

36 Yin Shi, Wan Fang. Effect of exercise during dialysis on microinflammatory status of maintenance hemodialysis patients [J]. Modern Clinical Nursing, 2016, 15(8): 54-8.

37 Zhou Ruiling, Zhou Ping, Lai Qi, et al. Study on the effect of nursing intervention combined with exercise therapy on the quality of life of hemodialysis patients [J]. Guangxi Medicine, 2016, 38(1): 134-6+9.

38 Zhu LH, Zhu JY. Clinical observation of moxibustion combined with aerobic exercise to improve depressive symptoms in hemodialysis patients treated with fluoxetine [J]. Hunan Journal of Traditional Chinese Medicine, 2016, 32(2): 95-7.

39 Guo Shuzhen, Zhang Yang. Clinical study on peripheral neuropathy in uremic patients by combination of leucovorin and hemodialysis [J]. Heilongjiang Medicine, 2017, 30(4): 817-9.

40 Lv Liqiao. Effect of exercise on symptomatic hypotension and dialysis adequacy during dialysis [J]. Zhejiang Clinical Medicine, 2017, 19(5): 923-4.

41 Ren Kejun, Wang Xiaoqin. Meta-analysis of the effectiveness of exercise therapy in the treatment of restless leg syndrome on hemodialysis [J]. Anhui Medicine, 2017, 21(1): 82-7.

42 Sun Danqin. Effects of rehabilitation exercise on cardiac function and quality of life of maintenance hemodialysis patients [J]. Biped and Health Care, 2017, 26(10): 53-+5.

43 Wang Hui. Effect of low-intensity aerobic training combined with health education on complication rate and quality of life of maintenance hemodialysis patients [J]. Nursing Practice and Research, 2017, 14(19): 31-3.

44 Wang Y, Gan LY, Ma YC, et al. A multicenter current investigation on the acceptance of rehabilitation exercise in dialysis among maintenance hemodialysis patients [J]. China Blood Purification, 2017, 16(12): 798-802.

45 Yin Lixia, Hu Xiaoyan, Zhang Hailin, et al. Effects of exercise in dialysis on dialysis adequacy and sleep quality of maintenance hemodialysis patients [J]. China Nursing Management, 2017, 17(11): 1478-81.

46 Zhang Lan, Cheng Yanjiao, Zhao Xinju, et al. Study on the effects of recumbent gymnastics on toxin clearance and inflammatory status in maintenance hemodialysis patients [J]. China Blood Purification, 2017, 16(4): 247-50.

47 Zou, Yumei. Study on the effect of resistance training combined with aerobic exercise on sleep quality and dialysis-related fatigue in maintenance hemodialysis patients [J]. China Convalescent Medicine, 2017, 26(1): 13-6.

48 Cai Shulan, Jia Hongguang, Zhang Suying, et al. Effects of aerobic exercise on oxidative stress and quality of survival in hemodialysis patients with diabetic nephropathy [J]. Journal of North China University of Science and Technology (Medical Edition), 2018, 20(1): 21-4.

49 Cui Huifang, Qiang Yanjuan, Xu Zhongxiu, et al. Analysis of the role of aerobic exercise workout on sleep quality and adverse emotions in uremic hemodialysis patients [J]. World Journal of Sleep Medicine, 2018, 5(12): 1473-5.

50 Li Qianling, Wu Bijing, Li Guanmei. Analysis of the preventive effect of rational exercise care on hypotension in maintenance hemodialysis patients [J]. Nursing Practice and Research, 2018, 15(7): 39-40.

51 Li Yuanyuan, Zhu Haodong, Zhang Li, et al. Effect of exercise team on improving quality of life sleep quality and dialysis adequacy in hemodialysis patients [J]. Journal of Xinjiang Medical University, 2018, 41(10): 1231-4+8.

52 Liu Xiaochen, Wang Xiuli, Li Na, et al. Effects of motivational interviewing on exercise compliance in continuous ambulatory peritoneal dialysis patients [J]. Qilu Nursing Journal, 2018, 24(23): 59-62.

53 Luo Q. Analysis of the impact of targeted nursing interventions on the quality of uremic hemodialysis patients [J]. Diet Science, 2018, (11X): 266-.

54 Qi L, Chen Y, Chen L, et al. Observation on the efficacy of cardiac rehabilitation exercise on patients with cardiac insufficiency combined with maintenance hemodialysis [J]. China Rehabilitation, 2018, 33(4): 297-300.

55 Qin Yanshi. Effects of exercise intervention on maintenance hemodialysis patients [J]. Nursing Practice and Research, 2018, 15(8): 45-7.

56 Wang X, Zhou Lijuan, Bian Yueqiu. Analysis of the effect of cerebral impedance training combined with aerobic exercise on fatigue status and sleep quality of maintenance hemodialysis patients [J]. Journal of Taizhou Institute of Vocational Technology, 2018, 18(5): 61-3.

57 Wang Wenting, Wu Chao, Luo Yongqin, et al. Meta-analysis of the effects of aerobic exercise on somatic function and quality of life of hemodialysis patients [J]. Chinese Journal of Modern Nursing, 2018, 24(23): 2797-803.

58 Wang YAN. Effect of acupoint massage on fatigue state and lower limb motor function of hemodialysis patients with diabetic nephropathy [J]. Biped and Health Care, 2018, 27(5): 149-+51.

59 Wang Y, Gan LY, Liang JQ, et al. A multicenter investigation on the withdrawal rate of exercise rehabilitation in maintenance hemodialysis patients on dialysis [J]. China Blood Purification, 2018, 17(12): 810-3.

60 Wei S.Y.. Effect of anti-resistance exercise intervention on dialysis adequacy in maintenance hemodialysis patients [J]. Nursing Practice and Research, 2018, 15(10): 31-2.

61 Xu niobium, Zheng guiqiong, Li xiaomei, et al. Mobile phone application software for home exercise guidance for hemodialysis patients [J]. Journal of Nursing, 2018, 33(11): 97-100.

62 Xu Yueping, Hu Qian, Zhu Xiaozhen, et al. Effects of resistance training combined with aerobic exercise on sleep quality and fatigue in maintenance hemodialysis patients [J]. Nursing Research, 2018, 32(2): 317-9.

63 Yang T, Chen M, Tang S, et al. Effects of Pilates exercise on fatigue status and sleep quality of maintenance hemodialysis patients [J]. China Blood Purification, 2018, 17(7): 456-60.

64 Yi Li-Hua. Effect of exercise therapy with nursing intervention on nutritional status and quality of life of maintenance hemodialysis patients [J]. Medical Information, 2018, 31(16): 175-7.

65 Zheng Suping, Ning Qian, Yang Huili. Effect of individualized exercise combined with targeted nursing intervention on nutritional status and quality of life of uremic hemodialysis patients [J]. Henan Medical Research, 2018, 27(17): 3252-3.

66 Zou JH, Bian BH, Hu DK. Effect of individualized exercise intervention on improving quality of life of elderly maintenance hemodialysis patients [J]. Practical Geriatrics, 2018, 32(7): 649-52.

67 A Xian Uzhina, Zhang Li, Li Yufang, et al. Study on the effects of tai chi exercise on blood pressure, heart rate and physical changes in hemodialysis patients [J]. Chinese Journal of Integrative Nephrology, 2019, 20(9): 776-80.

68 Chen YF, Dong YZ, Zhou ML, et al. Effects of exercise-music therapy on general well-being and coping styles of maintenance hemodialysis patients [J]. Zhejiang Medical Education, 2019, 18(5): 32-4+8.

69 Ding SJ. Nursing+ Analysis of the application of exercise therapy in hemodialysis [J]. China Continuing Medical Education, 2019, 11(18): 141-3.

70 Gao Siyao, Lu Wanguang. Meta-analysis of the Effectiveness of Aerobic Exercise in Improving the Quality of Survival of Maintenance Hemodialysis Patients [J]. Chinese Journal of Rehabilitation Medicine, 2019, 34(4): 453-9.

71 Guan Junrong, Lai Shaoyan. Effects of rehabilitation exercise training on sleep quality and fatigue level of uremic hemodialysis patients [J]. Chinese Journal of Practical Nursing, 2019, 35(26): 2012-6.

72 Han Mei. Evaluation of the effect of supportive psychotherapy combined with aerobic exercise applied to maintenance hemodialysis patients [J]. Medical Clinical Research, 2019, 36(11): 2193-5.

73 Hao D. Yun. Application of resistance training combined with aerobic exercise in hemodialysis patients [J]. Nursing Practice and Research, 2019, 16(21): 66-7.

74 HU Xiaoyan, YIN Lixia, ZHANG Hailin, et al. Effect of multidisciplinary cooperative exercise therapy on quality of life and microinflammatory status of maintenance hemodialysis patients [J]. China Nursing Management, 2019, 19(10): 1467-71.

75 Huang Chunxia, Lin Qiuhua, Wu Jinquan. Effects of aerobic exercise in dialysis on sleep quality and quality of life of hemodialysis patients [J]. Nursing Practice and Research, 2019, 16(3): 35-6.

76 Huang Lijuan, Duan Peibei, Zhang Jing, et al. Effects of exercise guidance on quality of life of hemodialysis patients based on trans-theoretical modeling [J]. China Blood Purification, 2019, 18(3): 181-4.

77 Jiao Lijuan, Pazetanmu Tohti, Li Yufang. Analysis of the Effects of Aerobic Exercise Combined with Levocanidin on Inflammatory State, Oxidative Stress and Nutritional Status of Maintenance Hemodialysis Patients [J]. Chinese Journal of Frontiers of Medicine (Electronic Edition), 2019, 11(11): 90-3.

78 Liang Yanping, Zheng Juanlian, Zhang Yingjun. Effects of aerobic-resistance exercise intervention on somatic function and oxidative stress in maintenance hemodialysis patients [J]. General Practice Nursing, 2019, 17(27): 3358-61.

79 LIU Dongmei. the effect of rehabilitation training on fatigue status of maintenance hemodialysis (mhd) patients [J]. Modern Digestive and Interventional Diagnosis and Treatment, 2019, 24(A01): 0408-9.

80 Liu J. Effects of aerobic exercise workout on sleep quality and adverse emotions in uremic hemodialysis patients [J]. World Journal of Sleep Medicine, 2019, 6(8): 1039-40.

81 Luo Jicong, Liang Li. Design and implementation of peritoneal dialysis patient training model based on vark learning style [J]. China Blood Purification, 2019, 18(2): 137-41.

82 Wang Gaying, Chen Xuefang. Effect of low-intensity aerobic exercise on dialysis effect and fatigue condition of hemodialysis patients [J]. Big Doctor, 2019, 4(13): 1-2+7.

83 Wang L, Wang H. Research on the application effect of exercise management pyramid picture in hemodialysis patients [J]. Chinese Medical Science, 2019, 9(15): 199-201.

84 Wang L. Effects of targeted nursing intervention combined with individualized exercise on nutritional status and quality of life of uremic hemodialysis patients [J]. Capital Food and Medicine, 2019, 26(20): 91-.

85 Wang X, Zhou Lijuan. Effectiveness of planned exercise intervention for hemodialysis patients [J]. Contemporary Nurses (in Chinese), 2019, 26(6): 103-5.

86 Wang Wenting, Wu Chao, Shen Meifen, et al. Meta-analysis of the effects of aerobic exercise on dialysis adequacy, microinflammatory status and albumin level in patients on dialysis [J]. Zhejiang Medicine, 2019, 41(3): 255-9+85.

87 Wang Xiaojing, Zhang Kun, Ge Yan, et al. Effects of exercise in hemodialysis on inflammation, oxidative stress and endothelial function in maintenance hemodialysis patients [J]. China Blood Purification, 2019, 18(6): 390-3.

88 Wang Yanping, Cai Yuwei, Mao Weijun, et al. Research on the application effect of exercise management pyramid picture in hemodialysis patients [J]. Chinese Journal of Preventive Medicine, 2019, 20(8): 763-5.

89 Xiao Y, Liu Jun, Xie Lizhen. Effect of exercise therapy on the occurrence of hypotension in hemodialysis patients [J]. Chinese Contemporary Medicine, 2019, 26(11): 48-50.

90 Xie Caiyun, Yan Jing, Zou De'e, et al. Study on the effect of aerobic exercise on toxin clearance in maintenance hemodialysis patients [J]. China Modern Drug Application, 2019, 13(23): 51-2.

91 Xu Jianjing, Zhang Tianda. Effect of collaborative care model on sleep, exercise and nursing satisfaction of uremic hemodialysis patients [J]. Massage and Rehabilitation Medicine, 2019, 10(2): 71-2.

92 Xu WEN N, Weng JJ, Jiang LY. Improvement effect of self-management education on self-management behavior of maintenance hemodialysis patients [J]. Chinese Primary Medicine, 2019, 26(12): 1506-9.

93 Xu Pengge, Shi Xiaona. Evaluation of the effect of acupoint massage on lower limb motor function and nursing effect in hemodialysis patients with diabetic nephropathy [J]. Biped and Health Care, 2019, 28(17): 37-8.

94 Yan Bi-yan, Mai Cui-fang, Yang Wei-hong. Effect of target-intensity aerobic exercise workout on nutritional status of peritoneal dialysis patients [J]. China Clinical Nursing, 2019, 11(5): 411-5.

95 Yi Chenchen. Research on the Application Effect of Aerobic Exercise on Weakness of Maintenance Hemodialysis Patients [J]. Contemporary Nurses (Lower Decade), 2019, 26(9): 120-2.

96 Yu XW, Zhang W, Chen QY, et al. Correlation between rehabilitation exercise and blood pressure regulation in peritoneal dialysis patients [J]. Health Research, 2019, 39(5): 510-3.

97 Zhang Xiaoyan. Effects of individualized exercise combined with targeted nursing intervention on nutritional status and quality of life of uremic hemodialysis patients [J]. Chinese and Foreign Women's Health Research, 2019, 0(10): 19-20.

98 Zhong Lijuan. Effects of regular aerobic exercise on body mass control and quality of life of maintenance hemodialysis patients [J]. Nursing Practice and Research, 2019, 16(23): 75-7.

99 Chang Pingping, He Kai, Li Heng. Effects of resistance training combined with aerobic exercise on fatigue and quality of life of maintenance hemodialysis patients [J]. Medical Theory and Practice, 2020, 33(9): 1530-2.

100 Shen F, Wang WT, Shen MF, et al. Meta-analysis of the effects of resistance exercise on exercise capacity respiratory function and quality of life in maintenance hemodialysis patients [J]. Journal of Nurse Advancement, 2020, 35(1): 23-8+33.

101 Chen S-L, Li S-qing. Nursing research on the effect of exercise intervention on symptomatic hypotension in dialysis patients [J]. Chinese and Foreign Medicine Research, 2020, 18(10): 59-61.

102 Dai Shanshan, Yu Haiyan, Lai Shuang, et al. Effects of incremental resistance exercise on hemoglobin and iron metabolism in maintenance hemodialysis patients on dialysis [J]. West China Medicine, 2020, 35(7): 781-7.

103 Lian Fen, Wu Jing, Shen Huajuan, et al. Repeated-measures ANOVA of low-intensity recumbent gymnastics on dialysis adequacy in maintenance hemodialysis patients [J]. Nursing and Rehabilitation, 2020, 19(2): 55-8.

104 Liang Zhazha, Zhou Huahong, Liang Yanjuan, et al. Effects of multidisciplinary cooperative exercise therapy on maintenance hemodialysis patients [J]. Qilu Nursing Journal, 2020, 26(17): 69-71.

105 Liu Ji-Mei. Effects of rehabilitation exercise training on sleep quality and fatigue level of uremic hemodialysis patients [J]. Systemic Medicine, 2020, 5(15): 165-8.

106 Miao A-Feng, Zhou Li-Juan. Effects of exercise training therapy on dialysis adequacy in uremic hemodialysis patients [J]. International Nursing Medicine, 2020, 2(1): 42-.

107 Na Wei. A study on the effect of collaborative care model on sleep, exercise and nursing satisfaction of uremic hemodialysis patients [J]. World Journal of Sleep Medicine, 2020, 7(12): 2160-2.

108 Niu Tieming, Han Yi, Luan Xunfei, et al. Effects of aerobic exercise combined with resistance exercise on microinflammation and t-cell subsets in peritoneal dialysis patients [J]. Chinese Journal of Practical Internal Medicine, 2020, 40(6): 493-6+501.

109 Shou Dan, Liang Xin, Jin Dan, et al. Meta-analysis of the Effect of Resistance Exercise on Blood Pressure Control and Exercise Tolerance Recovery in Hemodialysis Patients [J]. Journal of Nursing, 2020, 27(15): 44-9.

110 Wang Tao, Liu Yongmei. Effects of aerobic exercise on mineral metabolism, sleep quality and fatigue in maintenance hemodialysis patients [J]. Medical Information, 2020, 33(23): 77-9+95.

111 WANG Wenjing, LI Yanbin, ZHANG Zewei. Effects of exercise therapy combined with nursing intervention on nutritional status and quality of life in maintenance hemodialysis [J]. Clinical Medicine Practice, 2020, 29(2): 155-8.

112 Wang Wuxiao, Li Jialing, Yu Tao, et al. Observation on the prevention and treatment effect of bicycle exercise in dialysis on restless leg syndrome in uremic patients [J]. China Blood Purification, 2020, 19(4): 230-3.

113 Wang Xiaoyan, Qin Minghua. Effects of Time exercise rehabilitation education on somatic function and survival quality of maintenance hemodialysis patients [J]. Contemporary Nurses (上旬刊), 2020, 27(9): 136-8.

114 Wu Jiao-hua, Liang Yan-ping, Lai Xiaobing. Effects of a precise and quantitative walking exercise intervention on maintenance hemodialysis patients [J]. General Practice Nursing, 2020, 18(4): 441-3.

115 Xiong M. Effects of individualized exercise program on prognosis and social regression of maintenance hemodialysis patients [J]. Contemporary Nurses (Zhongdian), 2020, 27(12): 75-7.

116 Ye Xiaoshang, Jiang Luyue, Jun Chenxia, et al. Effects of aerobic-resistance exercise on physiological function and quality of life of maintenance hemodialysis patients [J]. Chinese Science and Technology Journal Database (Full Text Edition) Medicine and Health, 2020, (11): 0031-+33.

117 Yuan Mei. Analysis of the effect of multidisciplinary cooperative exercise therapy applied to maintenance hemodialysis patients [J]. Medical Food Therapy and Health, 2020, 18(11): 7-+13.

118 Zhang Cai-Ling, Chen Feng-Yu, Chen Lian-Di. Effects of aerobic exercise combined with resistance training on somatic function and quality of life of hemodialysis patients [J]. Integrative Nursing in Chinese and Western Medicine (in Chinese and English), 2020, 6(11): 84-7.

119 Zhang L, Li A M, Liu Y J, et al. Study on the Intervention Effect of Aerobic Exercise in Dialysis on Restless Legs Syndrome and Psychological Condition of Maintenance Hemodialysis Patients [J]. Journal of Clinical Nephrology, 2020, 20(7): 586-90.

120 Zhang LH, Zhou WP, Hong MQ, et al. Effects of resistance exercise on blood pressure and quality of life of maintenance hemodialysis patients [J]. Medical Equipment, 2020, 33(15): 136-8.

121 Zhang Xiaohong. Effects of rehabilitation exercise nursing on psychological status of elderly hemodialysis patients [J]. Journal of Electrocardiography (Electronic Edition), 2020, 9(2): 174-5.

122 Zhang Yan, Yue Sign, Hu Meiyan. Effect of exercise therapy in hemodialysis patients [J]. Nursing Practice and Research, 2020, 17(3): 90-2.

123 Zhao P, Huang YL, He L, et al. Meta-analysis of the effects of bicycle exercise on exercise capacity and circulatory status in maintenance hemodialysis patients [J]. Chinese Family Medicine, 2020, 23(14): 1769-77.

124 Zheng F, Huang XL. Effects of cardiac rehabilitation exercise on cardiac function indexes in patients with chronic renal failure combined with cardiac insufficiency undergoing maintenance hemodialysis [J]. Chinese and Western Medicine Nursing, 2020, 6(12): 45-8.

125 Zhu Li-Yang, Lu Mei-Su, Wang Hong-Lin, et al. Effects of planned aerobic-resistance exercise during the dialysis interval on patients' nutritional status and dialysis hypotension [J]. Chinese Journal of Modern Nursing, 2020, 26(14): 1894-8.

126 Cai Yanju, Chen Xiao. Effects of resistance exercise intervention on fatigue state and physical activity level of maintenance hemodialysis patients [J]. International Journal of Nursing, 2021, 40(20): 3734-8.

127 Jianhua Cen. Effects of moxibustion treatment combined with low-intensity aerobic exercise on sleep quality and fatigue in maintenance hemodialysis patients [J]. World Journal of Sleep Medicine, 2021, 8(8): 1351-2.

128 Chen Na. Effects of exercise-music therapy on motor function, negative emotions and fatigue in maintenance hemodialysis patients [J]. Dialysis and Artificial Organs, 2021, 32(4): 69-70.

129 Cheng J, Tao LL. Effect of active limb movement on symptomatic hypotension in hemodialysis patients [J]. Practical Clinical Medicine (Jiangxi), 2021, 22(1): 60-1+97.

130 CHENG Xinjie, TANG Liqun, ZHANG Zhen, et al. Effect of recumbent bicycle exercise on dialysis adequacy in maintenance hemodialysis patients [J]. Minimally Invasive Medicine, 2021, 16(5): 724-7.

131 Cui Chengji, Liu Chunyan, Zhang Hongbao, et al. Effects of Chinese medicine nursing intervention on dialysis quality of elderly hemodialysis patients [J]. China Traditional Chinese Medicine Modern Distance Education, 2021, 19(15): 147-9.

132 Dai Shanshan, Ma Yingchun. Effects of incremental resistance exercise in dialysis on nutritional status and body fat composition of maintenance hemodialysis patients [J]. Chinese Journal of Nephrology, 2021, 37(5): 434-7.

133 Fan Jia Yun. Observations on the effects of collaborative care model on sleep and exercise of uremic hemodialysis patients [J]. China Science and Technology Periodicals Journal Database Medicine, 2021, (7): 0070-1.

134 Feng Lei, Li Yunshu, Yang J, et al. Effects of virtual reality-based somatosensory game integrated into home dialysis exercise management on fall risk and quality of life of dialysis patients [J]. China Blood Purification, 2021, 20(5): 302-5+32.

135 Feng Q, Buheliqi Maimeti, Zhang Lei. Application of dumbbell weighted swinging arm exercise in maturation of arteriovenous endovascular fistula in hemodialysis patients [J]. Xinjiang Medicine, 2021, 51(8): 866-8.

136 Fu Jun-Xiang, Wang Ai-Min, Zhou Yun-Ping, et al. Effects of eight-duanjin on sleep quality and negative emotions in maintenance hemodialysis patients [J]. Journal of Nursing Management, 2021, 21(4): 285-90.

137 Guo Suping, Shi Bin, Ji Xiaojing, et al. Impact of home walking exercise with the application of pedometer software+ weibo on the quality of life of maintenance hemodialysis patients [J]. Contemporary Nurses (上旬刊), 2021, 28(6): 135-8.

138 Heng Jia. Aerobic Exercise Helps Resolve Symptoms Associated with Dialysis [J]. Health and Beauty, 2021, (7): 24-.

139 Hua Qiong. Effects of Ba Duan Jin on microinflammatory status and exercise capacity of elderly peritoneal dialysis patients [J]. Chinese Convalescent Medicine, 2021, 30(7): 700-3.

140 Jiang Y, Cai WQ, Yan GL. Aerobic exercise combined with progressive muscle relaxation training in peritoneal dialysis patients [J]. Chinese Journal of Modern Nursing, 2021, 27(34): 4721-6.

141 Li HH. Evaluation of the effect of rehabilitation exercise care on the psychological state of hemodialysis elderly [J]. Yishoubaidian, 2021, (7): 0124-5.

142 Li H, Li BF. Effects of resistance exercise intervention in hemodialysis on the quality of life of maintenance hemodialysis patients [J]. Chinese Science and Technology Journal Database (Full Text Edition) Medicine and Health, 2021, (9): 0137-9.

143 Liang Yilan, He Dejiao, Yang Dingping, et al. Effects of aerobic combined stretching exercise on restless legs syndrome and quality of life in hemodialysis patients [J]. Chinese Journal of Practical Nursing, 2021, 37(29): 2280-7.

144 Liu Sha, Tang Xueqin. Effects of cognitive behavioral therapy combined with aerobic exercise on nutritional status and quality of life of hemodialysis patients [J]. Reflexology and Rehabilitation Medicine, 2021, 2(9): 164-6.

145 Lv Yanhui, Chen Jianhua, Ge Jiali, et al. Effect of exercise therapy on microinflammatory state and hyperhomocysteinemia in maintenance hemodialysis patients [J]. China Blood Purification, 2021, 20(3): 166-70.

146 Luo Yan, Wu Su-Min, Zhang Shao-Hua, et al. Effects of rehabilitation exercise intervention on sleep quality and quality of life of uremic hemodialysis patients [J]. World Journal of Sleep Medicine, 2021, 8(2): 196-8.

147 Miao Jiayi, Zhang Yiwen, Zhang Liyuan. Effects of aerobic exercise combined with resistance exercise on lipid metabolism, osteoporosis and blood pressure in maintenance hemodialysis patients [J]. Chinese Journal of Nephrology, 2021, 22(10): 911-3.

148 Pazetanmu Tohti, Li Yufang. Study on the effect of aerobic exercise on protein-energy consumption of maintenance hemodialysis patients on patients' nutrient status [J]. Chinese Science and Technology Journal Database (Full Text Edition) Medicine and Health, 2021, (11): 0007-9.

149 Qian M. Effects of resistance training combined with aerobic exercise on patients' quality of life and fatigue in maintenance hemodialysis care [J]. Chinese Science and Technology Journal Database (Full Text Edition) Medicine and Health, 2021, (10): 0225-6.

150 Wang Li-Hong, Ma Yuan. Study on the efficacy of diet and exercise guidance combined with rhubarb umbilical cord patch in hemodialysis patients with constipation [J]. Chinese Science and Technology Journal Database (Full Text Edition) Medicine and Health, 2021, (2): 0245-6.

151 Wang Chunrong, Liang Caihong, Chang Shengtao. Effects of planned aerobic exercise-resistance exercise intervention on oxidative stress in maintenance hemodialysis patients [J]. Wisdom Health, 2021, 7(31): 157-9.

152 Wang L, Zhou M, Wang J, et al. Effect of dietary management combined with aerobic exercise on volume load and cardiac function in maintenance peritoneal dialysis patients [J]. Chinese Journal of Practical Nursing, 2021, 37(36): 2813-8.

153 Wang Na, Li Yi. Effects of rehabilitation exercise training on sleep quality and fatigue level of uremic hemodialysis patients [J]. Chinese Pharmaceutical Industry, 2021, 30(S02): 276-.

154 Wang Shaohua, Li Xiaojuan, Luan Jie, et al. Clinical study on improving motor function of hemodialysis patients with meridian acupuncture [J]. Chinese Journal of Nephrology, 2021, 22(3): 218-21.

155 Wang Susu, Li Juanjuan, Hu Huagang. Meta analysis of the effect of exercise in dialysis on improving the quality of survival of maintenance hemodialysis patients [J]. Nursing Research, 2021, 35(6): 987-95.

156 Wang Wenjuan, Jiang Xia. Effects of different exercise modes on bone mineral density in maintenance hemodialysis patients [J]. Chinese Journal of Osteoporosis, 2021, 27(8): 1183-6+200.

157 WU Menghan, WANG Yan, AI Shuanglan, et al. Improvement effects of exercise on cardiopulmonary function and psychological status of maintenance hemodialysis patients [J]. Chinese Journal of Frontiers of Medicine (Electronic Edition), 2021, 13(8): 67-70.

158 XIA Jinghua, SONG Dan, ZHU Wenbo, et al. Effectiveness of exercise intervention during dialysis to improve the quality of life and sleep quality of elderly maintenance hemodialysis patients under multidisciplinary collaborative team model [J]. Practical Geriatrics, 2021, 35(12): 1254-7.

159 Xu Qinjuan, Hu Yanfei, Hu Huagang. Net Meta-analysis of Different Exercise Modes on Improving Walking Ability of Maintenance Hemodialysis Patients [J]. PLA Nursing Journal, 2021, 38(7): 1-5.

160 YAN Xing, HUANG Xue-Fang, HE Min-Jing, et al. Effects of metabolic-equivalent-based moderate-intensity aerobic exercise on sleep quality and fatigue in maintenance hemodialysis patients [J]. General Practice Nursing, 2021, 19(29): 4130-3.

161 Yang J, Feng L, Fu L, et al. Observations on the improvement of home exercise compliance and post-dialysis fatigue in dialysis patients by the "web-mediated+ exercise" program [J]. Journal of Clinical Nephrology, 2021, 21(7): 583-8.

162 Yang Xiaoling. Effects of exercise therapy on nutritional status and quality of life of maintenance hemodialysis patients [J]. China Health Standard Management, 2021, 12(14): 141-4.

163 Yao L. Effects of exercise nursing intervention on cardiopulmonary function and psychological status of patients undergoing maintenance hemodialysis [J]. Chinese and Western Medicine Nursing (in English), 2021, 7(8): 151-3.

164 Yu XT, Cao SM, Ji SJ. Meta-analysis of the effects of exercise on exercise capacity and dialysis efficiency in maintenance hemodialysis patients [J]. China Blood Purification, 2021, 20(8): 526-31.

165 Yu XT, Cao SM, Ji SJ, et al. Effects of exercise intervention in dialysis on maintenance hemodialysis patients [J]. Journal of Nursing, 2021, 36(17): 5-8.

166 Yuan Xiazhi, Hua Ying, Fang Jinqiong, et al. Application of horizontal bicycle exercise in preventing hypotension in patients undergoing hemodialysis [J]. Nursing and Rehabilitation, 2021, 20(2): 1-4.

167 Zhang Chengxiu. Effects of low-intensity aerobic rehabilitation exercise on sleep quality and fatigue in maintenance hemodialysis patients [J]. Reflexology and Rehabilitation Medicine, 2021, 2(21): 171-3.

168 Zhang Chengxiu. Effects of rehabilitation exercise on sleep quality and fatigue level of hemodialysis patients with chronic renal failure [J]. Reflexology and Rehabilitation Medicine, 2021, (14): 172-4.

169 Zhang LH, Zhou WP, Hong MQ, et al. Optimization of Resistance Exercise on Hemodialysis-Related Hypotension [J]. Cardiovascular Disease Prevention and Control Knowledge (Academic Edition), 2021, 11(7): 51-3+6.

170 Zheng Y, Zhang Y, Li R. The effect of multidisciplinary cooperative exercise therapy on maintenance hemodialysis patients [J]. Effectiveness of multidisciplinary cooperative exercise therapy for maintenance hemodialysis patients [J]. International Journal of Nursing, 2021, 40(14): 2610-4.

171 Zheng Yuxiu, Wang Fujun. Effects of exercise therapy on fatigue, psychological status and sleep quality of hemodialysis patients [J]. Reflexology and Rehabilitation Medicine, 2021, (13): 156-8.

172 Cai Guomei, Hu Jingwen, Wang Quanrui, et al. Effects of exercise rehabilitation on nutritional status and fatigue syndrome in maintenance hemodialysis patients [J]. Chinese Contemporary Medicine, 2022, 29(16): 140-3+7.

173 Chen Guanjie, Zhang Hailin, Yin Lixia, et al. Construction and application of an exercise intervention program for patients with maintenance hemodialysis combined with sarcopenia [J]. Chinese Journal of Nursing, 2022, 57(7): 798-806.

174 Chen Guanjie, Zhang Hailin, Yin Lixia, et al. Effects of exercise in dialysis on cognitively debilitated patients on maintenance hemodialysis [J]. Journal of Nursing, 2022, 37(20): 33-7.

175 Chen J, Liu YX, Luo XJ, et al. Meta-analysis of the effects of exercise intervention on somatic function in maintenance hemodialysis patients [J]. China Blood Purification, 2022, 21(11): 850-7.

176 Chen S.H., Mai L.Y., Feng J.X., et al. Effects of aerobic exercise on psychological status, physiological function and quality of life of peritoneal dialysis patients [J]. Internal Medicine, 2022, 17(3): 348-50.

177 Chen Yanni. Application effect of individualized exercise care in maintenance hemodialysis patients [J]. Contemporary Medicine, 2022, 28(26): 178-81.

178 Chen YW, Wei YB, Du HH. Effects of aerobic exercise based on safe heart rate control on exercise tolerance, cardiopulmonary function and fatigue in uremic maintenance hemodialysis patients [J]. General Practice Nursing, 2022, 20(24): 3386-8.

179 Fan Xiaobo, Jiang Yanping, Zhang Xiaoyan, et al. Application of upper limb rehabilitation exercises in maintenance hemodialysis patients [J]. Chinese Journal of Nursing, 2022, 57(21): 2572-8.

180 He Qi-en, Ying Guanghui, Chen Zhaogui, et al. Meta-analysis of the interventional effects of exercise therapy on hemodialysis restless legs syndrome [J]. Chinese Journal of Nephrology, 2022, 23(10): 876-81.

181 Hou Y. Observations on the Application Effect of Endurance Exercise in Elderly Maintenance Hemodialysis Patients [J]. Chinese and Western Medicine Nursing, 2022, 8(7): 7-11.

182 Huang Dejian, Dong Dabao, Tan Dandan, et al. Effect of cpet-guided aerobic exercise intervention on cardiopulmonary function in ckd peritoneal dialysis patients [J]. Chinese Journal of Border Health and Quarantine, 2022, 45(S01): 101-2+8.

183 Huo Hongyan. Effects of rehabilitation exercise care on anxiety and depression in elderly hemodialysis patients [J]. Yishoubaidian, 2022, (12): 0119-21.

184 Li J. M., Zhang Y. X., Hsing L. L., et al. Study on the use of personalized exercise program in improving sleep quality, quality of life, and blood pressure control in maintenance hemodialysis patients [J]. Chinese Science and Technology Journal Database (Full Text Edition) Medicine and Health, 2022, (8): 0033-6.

185 Li Xumei, Li Hao, Guo Lanying, et al. Effects of exercise therapy on exercise capacity and quality of life of maintenance hemodialysis patients [J]. Chinese Journal of Practical Nursing, 2022, 38(28): 2178-83.

186 Li Yi-Nan, Ma Tao, Zhang Lei. Application of aerobic-resistance exercise combined with stratified intervention in maintenance hemodialysis patients [J]. China Medical Journal, 2022, 19(12): 168-72.

187 Liang Yan, Zhang Xiaoqin. Application effect of bicycle exercise in patients with uremia complicated with restless leg syndrome on maintenance hemodialysis [J]. China Clinical Nursing, 2022, 14(6): 331-3.

188 Liu F, Wu Han, Zhang Yingying, et al. Application of incremental resistance exercise training combined with WeChat health education platform in the care of patients with chronic renal failure on maintenance hemodialysis [J]. China Medical Journal, 2022, 19(3): 162-5+81.

189 Liu Lanying. Effects of individualized exercise instruction on sleep quality and quality of life of hemodialysis patients [J]. World Journal of Sleep Medicine, 2022, 9(4): 754-6.

190 Liu Yinyan, Xu Fengling. Application effect of cycling exercise combined with respiratory training in elderly maintenance hemodialysis patients [J]. Clinical Medicine Research and Practice, 2022, 7(18): 176-8.

191 Luan Xuemei. The effect of nursing intervention combined with exercise therapy on the quality of life of hemodialysis patients [J]. Chinese Science and Technology Journal Database (Citation Edition) Medicine and Health, 2022, (12): 0176-8.

192 Luo Xiju, Deng Siyan, Chen Jing, et al. Net Meta-analysis of the Effect of 9 Exercise Modalities on Improving Dialysis Adequacy in Hemodialysis Patients [J]. Chinese Journal of Nursing, 2022, 57(23): 2921-9.

193 Ma Yue. A clinical study on the effects of positive meditation and breathing combined with Baduanjin on negative emotions and motor functions of hemodialysis patients [J]. Chinese Science and Technology Journal Database (Full Text Edition) Medicine and Health, 2022, (12): 0059-62.

194 Niu Tieming, Luan Xunfei, Dong Qingze, et al. Effects of aerobic exercise combined with resistance training on motor function and factors related to cardiovascular events in peritoneal dialysis patients [J]. Chinese Journal of Physical Medicine and Rehabilitation, 2022, 44(6): 540-2.

195 Pan Qin, Hao Yan, Liu Ping, et al. Effects of multimodal exercise on symptom clusters of fatigue-negative mood-sleep disorder in maintenance hemodialysis patients [J]. Hebei Medicine, 2022, 44(13): 1970-3+7.

196 Shi Xuan. Nursing Intervention of Exercise Prescription to Improve Thirst in Maintenance Hemodialysis Patients [J]. Chinese Science and Technology Journal Database (Citation Edition) Medicine and Health, 2022, (9): 0195-8.

197 Shi Xuan. Effects of arteriovenous endovascular fistula exercise on upper limb motor function and endovascular blood flow rate in dialysis patients [J]. Chinese Science and Technology Journal Database (Citation Edition) Medicine and Health, 2022, (8): 0079-82.

198 Su JT, Zhao YC. Effect of safe heart rate-controlled aerobic exercise in the care of uremic maintenance hemodialysis patients [J]. Medical Theory and Practice, 2022, 35(1): 165-7.

199 Su Xiaolian, Ai Lingyan, Duan Yinfeng, et al. Effects of aerobic exercise on resting energy metabolism, Pew state and cardiopulmonary endurance in maintenance hemodialysis patients [J]. Dialysis and Artificial Organs, 2022, 33(2): 96-101.

200 Tang Yi-Pin. Application effect observation of rehabilitation exercise training combined with dietary care in uremic hemodialysis patients [J]. Chinese Science and Technology Journal Database (Digest Edition) Medicine and Health, 2022, (10): 0244-6.

201 Tian Chaoyang, Wei Xuquan, Kong Lingxin, et al. Observations on the therapeutic effects of Kidney Protecting and Blood Activating Patch on exercise endurance and Chinese medicine symptoms in maintenance hemodialysis patients with spleen and kidney yang deficiency and blood stasis [J]. Sichuan Traditional Chinese Medicine, 2022, 40(1): 128-31.

202 Tong L, Li Yucui, Li Yang, et al. The effect of exercise education on hypotension in dialysis patients based on the Knowing, Believing and Doing model [J]. Hebei Medicine, 2022, 44(4): 532-5.

203 Wan Niu, Tong Hui, Wang Lisheng. Summary of the best evidence for exercise management programs for maintenance hemodialysis patients [J]. Evidence-Based Nursing, 2022, 8(13): 1719-24.

204 Wang Tingting, Chen Xiangjiao, Liang Yanjuan. Effects of low-intensity aerobic rehabilitation exercise on psychological status and lower limb motor function of uremic hemodialysis patients [J]. Jilin Medical Science, 2022, 43(2): 565-6.

205 WANG Weiping, JIANG Lu, ZHANG Qiang, et al. Effects of exercise rehabilitation under the guidance of cardiopulmonary exercise test on immune function and fatigue level of hemodialysis patients [J]. Modern Journal of Integrative Medicine, 2022, 31(10): 1337-41+427.

206 Wang Xianfang, Liu Junhui. Effectiveness of Ba Duan Jin applied to patients on maintenance hemodialysis [J]. Chinese and Western Medicine Nursing, 2022, 8(12): 41-4.

207 Wu Juntao, Qiu Juanjuan, Lin Cuiyun. Effects of tai chi exercise combined with acupressure on fatigue and sleep quality of hemodialysis patients [J]. Contemporary Nurses (Zhongdian), 2022, 29(8): 72-4.

208 Wu Hao, Xu Fangfang, Tao Ling. Effects of aerobic stretching exercise on hemodialysis patients with restless legs syndrome [J]. China Medical Innovation, 2022, 19(27): 108-12.

209 Xia Dan, Tong Hui, Wang Lisheng. Summary of the best evidence on exercise management programs for peritoneal dialysis patients [J]. China Clinical Nursing, 2022, 14(11): 700-6.

210 Xia JH, Zhu WB, Song D, et al. Application effect of three-party empowerment combined with Zhixinxing health education model in exercise intervention for maintenance hemodialysis patients [J]. Guangxi Medical Science, 2022, 44(17): 2065-8.

211 Xia M, Chen FX. Application value of bicycle exercise therapy and gabapentin in treating hemodialysis patients with restless leg syndrome [J]. Chinese Family Medicine, 2022, 20(5): 793-5+807.

212 Xie Lijuan, Shi Suhua, You Gengji. Effect of aerobic exercise care in maintenance hemodialysis patients [J]. Zhongguo Nankang Med, 2022, 34(13): 190-2.

213 Xiong Changqing, Wang Weiping, Jia Xiaojun. Effects of aerobic exercise on oxidative stress and quality of life in maintenance hemodialysis patients [J]. Contemporary Medicine, 2022, 20(23): 36-8.

214 Xu Lixuan, Liu Jianjing, Xu Yingyin, et al. Meta-analysis of the effectiveness of Baduanjin exercise on improving sleep quality of maintenance hemodialysis patients [J]. Primary Chinese Medicine, 2022, 1(1): 57-63.

215 Xue WJ, Wu Han, Liu F, et al. Application of rehabilitation nursing based on haccp principles combined with exercise therapy in maintenance hemodialysis patients [J]. China Medical Journal, 2022, 19(33): 151-4.

216 Yan Xing, Zhao Qiaohong, Peng Yaoli. Effects of progressive resistance exercise on exercise capacity, nutritional indicators and sleep quality of maintenance hemodialysis patients [J]. Evidence-Based Nursing, 2022, 8(9): 1215-9.

217 YE Dan, ZHENG Na, ZHAO Lingyu, et al. The effects of continuity of care with aerobic exercise on sleep quality and symptoms in maintenance hemodialysis patients [J]. Journal of Naval Medicine, 2022, 43(6): 607-10.

218 Ye Xia. Application effect of individualized exercise combined with targeted nursing care in hemodialysis patients [J]. Chinese Science and Technology Journal Database (Full Text Edition) Medicine and Health, 2022, (7): 0071-3.

219 Ye Xiaoshang, Jiang Luyue, Jun Chenxia, et al. Effects of aerobic-resistance exercise on physiological function and quality of life of elderly hemodialysis patients [J]. Chinese Journal of Gerontology, 2022, 42(6): 1399-402.

220 Zhang Chenjie, Hao Jingyu, Zhang Hongmei, et al. Analysis of the effects of incremental resistance exercise on body fat composition and nutritional status of mhd patients on dialysis [J]. Chongqing Medicine, 2022, 51(S01): 217-8.

221 Zhang Fan, Liao Jing, Zhang Weihong, et al. Evaluation of the application effect of eight-duanjin exercise in peritoneal dialysis patients [J]. Shanghai Nursing, 2022, 22(8): 34-7.

222 Zhang Yayi, Li Chunxi, Huang Xiaoling. Effectiveness of exercise intervention in preventing symptomatic hypotension during hemodialysis [J]. Primary Medical Forum, 2022, 26(21): 1-3.

223 Zhang Yuanli, Liu Yinglian, Lin Zicheng, et al. Effects of exercise management guided by traditional Chinese medicine exercise theory on dialysis adequacy and microinflammatory status in mhd patients [J]. Chinese Journal of Gerontology, 2022, 42(17): 4210-4.

224 Zhao Chen, Yan Li. Analysis of the application value of exercise intervention therapy in hemodialysis patients based on multidisciplinary collaboration [J]. Xinjiang Medicine, 2022, 52(6): 711-4.

225 Zhu D, Li LJ. Application effect of functional exercise training in the treatment of chronic kidney disease patients [J]. Chinese Medical Science, 2022, 12(17): 9-13.

226 Zhu Li-Fang. Effect of incremental resistance exercise in hemodialysis applied to maintenance hemodialysis patients [J]. Dialysis and Artificial Organs, 2022, 33(3): 85-8.

227 Zuo Liyan. Effectiveness of collaborative exercise therapy management based on multidisciplinary diagnosis and treatment in maintenance hemodialysis patients [J]. Dialysis and Artificial Organs, 2022, 33(3): 81-4.

228 Zeng Fei, Wang Xiaosheng. Effect of self-management based on WeChat punch card supervision on the effect of exercise intervention in maintenance hemodialysis patients [J]. International Journal of Nursing, 2023, 42(24): 4596-600.

229 Chen Feifei. Effects of cpet-based rehabilitation exercise on immune function and fatigue in mhd patients [J]. Guangzhou Medicine, 2023, 54(10): 101-5.

230 Chen Huiping, Kong Xiaoli, Chen Ning. Effects of eight-duanjin exercise combined with traditional Chinese medicine dietary therapy on volume load, nutrition and fatigue in patients undergoing maintenance peritoneal dialysis [J]. Qilu Nursing Journal, 2023, 29(17): 57-60.

231 Chen Jinrong, Chen Yayan, He Liping, et al. Analysis of the effects of individualized exercise therapy combined with nursing intervention on sleep quality and quality of life of hemodialysis patients [J]. World Journal of Sleep Medicine, 2023, 10(4): 923-5+9.

232 Chen Shuangru, Cui Wenfang, Lv Huali. Effects of individualized exercise therapy on negative emotions and sleep quality in maintenance hemodialysis patients [J]. World Journal of Sleep Medicine, 2023, 10(10): 2482-4.

233 Zhai Wanchun, Wu Shumin, Mo Yangping, et al. Effect of nutritional support combined with exercise training on the debilitating condition of hemodialysis patients [J]. China Health Standard Management, 2023, 14(15): 187-90.

234 Fan Huanxin. Impact of nutritional management combined with aerobic exercise on the nursing outcomes of elderly patients on maintenance hemodialysis [J]. Evidence-Based Nursing, 2023, 9(9): 1701-4.

235 Fan Yunjing, Xu Chen. Evaluation of the effect of individualized exercise combined with targeted nursing care on uremic hemodialysis patients [J]. Chinese Science and Technology Journal Database (Full Text Edition) Medicine and Health, 2023, (9): 0156-9.

236 FANG Meng, DAI Min, YANG Wenjuan, et al. Effects of aerobic combined with resistance exercise on elderly maintenance hemodialysis patients with obese sarcopenia [J]. Journal of Nursing, 2023, 38(5): 95-100.

237 Guo Yueyue, Bo Xiangmin, Liu Shengfeng, et al. Application of Fitness Qigong Five-Animal Play in Exercise Rehabilitation of Dialysis Patients with Myasthenia Gravis [J]. Journal of Nurse Advancement, 2023, 38(20): 1865-9.

238 He Yanping. Effects of intermittent exercise care in dialysis on the incidence of hypotension and quality of life of maintenance hemodialysis patients [J]. Scientific Counseling, 2023, (7): 142-4.

239 Hu Yachan. Effect of leucovorin combined with resistance exercise training on hypotension in hemodialysis patients [J]. Medical Theory and Practice, 2023, 36(24): 4308-9+18.

240 Hu Yanshuo, Gao Yanjun, Chen Fang, et al. Observation on the comprehensive effect of home exercise management program on hemodialysis patients [J]. Chinese Journal of Nephrology, 2023, 24(6): 529-32.

241 Li Bo, Xie Wenjun, Zhang Li, et al. Effect of upper limb local exercise on the incidence of avf complications in maintenance hemodialysis patients [J]. Chinese Science and Technology Journal Database (Full Text Edition) Medicine and Health, 2023, (9): 0048-52.

242 Li Hongli, Wang Yan, Niu Xiuru, et al. Intervention Study of Individualized Exercise on Survival Quality of Elderly Maintenance Hemodialysis Patients [J]. Primary Chinese Medicine, 2023, 2(8): 58-63.

243 Li J, Liao J-So, Liu X-Y, et al. Effects of personalized exercise combined with targeted nursing intervention on uremic hemodialysis patients [J]. Chinese Community Physician, 2023, 39(6): 126-8+31.

244 Li Pingping, Liu Yunqi. Effects of aerobic exercise on anxiety and depression in patients with end-stage renal disease on maintenance hemodialysis [J]. Frontiers of Medicine, 2023, 13(32): 15-7.

245 Li Qiaofen. Application of Ba Duan Jin exercise in maintenance hemodialysis patients [J]. Chinese Science and Technology Journal Database (Citation Edition) Medicine and Health, 2023, (3): 0009-13.

246 Li Wanlin, Yun Jie, He Ling, et al. Bayesian net meta-analysis of the effect of different exercise therapies on fatigue intervention in maintenance hemodialysis patients [J]. Modern Medicine and Health, 2023, 39(24): 4215-24+31.

247 Li YY, Li T, Zhao XH, et al. Net Meta-analysis of the Effect of Three Exercise Therapies on the Intervention of Non-Dialysis Chronic Kidney Disease [J]. Chinese Journal of Evidence-Based Medicine, 2023, 23(1): 34-40.

248 Liang Yumei, Shen Yan. Effect of continuity of care of exercise in dialysis on mhd patients [J]. Corps Medicine, 2023, (2): 75-7.

249 LIN Haixue, SHENG Qilin, ZHU Beixia, et al. Summary of the best evidence on home exercise rehabilitation for peritoneal dialysis patients [J]. Chinese Journal of Modern Nursing, 2023, 29(9): 1176-81.

250 Liu J, Zhu XL, Chen XX, et al. Effects of exercise rehabilitation on peripheral nerve function and fatigue status in maintenance hemodialysis patients [J]. Journal of Modern Electrophysiology, 2023, 30(2): 81-4.

251 Liu Yibing, Ding Meimei, Wang Xiaoyu, et al. Exercise intervention based on adopt model in hemodialysis patients [J]. Journal of Nursing, 2023, 38(13): 78-82+8.

252 Lou L, Chen Chen, Jiang Chunlei. Effectiveness of exercise therapy on dialysis-associated hypotension in patients [J]. Chinese Science and Technology Journal Database (Full Text Edition) Medicine and Health, 2023, (2): 0086-8.

253 Luo Xiju, Liu Yixiu, Deng Siyan, et al. Meta-analysis of the effect of exercise in dialysis on improving dialysis adequacy and solute clearance in hemodialysis patients [J]. Journal of Nurse Advancement, 2023, 38(20): 1890-6.

254 Qiqige, Zhao Jianrong. Effects of aerobic and impedance exercise on dialysis adequacy and quality of life of hemodialysis patients [J]. China Blood Purification, 2023, 22(7): 498-502.

255 Tan Yixiang, Azati Jainasi, Wang Xiaoqin, et al. Effects of multimodal exercise training on volume loading and nutritional status of continuous ambulatory peritoneal dialysis patients [J]. Chinese Science and Technology Journal Database (Full Text Edition) Medicine and Health, 2023, (11): 0027-32.

256 Tang, Hongzhen. Finger-fist-arm movement exercise combined with video feedback education in the maturation of arteriovenous endovascular fistula in hemodialysis patients [J]. China Clinical Nursing, 2023, 15(9): 557-9.

257 Tian W. Effect of individualized exercise therapy care for maintenance hemodialysis patients [J]. Chinese and Foreign Medicine Research, 2023, 2(14): 112-4.

258 TIAN Zhijuan, XU Jun, DAI Huanhuan, et al. Effects of progressive rehabilitation exercise on respiratory function, motor function and iron metabolism in elderly hemodialysis patients [J]. Qilu Nursing Journal, 2023, 29(21): 13-6.

259 Wang Manli, Weng Yingying, Zhang Ping, et al. Analysis of the effect of target-intensity guided aerobic exercise workout method on the nutritional status of abdominal dialysis patients with chronic renal failure [J]. Chinese Contemporary Medicine, 2023, 30(16): 96-9+103.

260 Weng Yaping, Fei Jing, Zhu Yafei. Effects of nutritional ratios combined with individualized exercise program on postmenopausal women on hemodialysis [J]. China Maternal and Child Health, 2023, 38(22): 4340-3.

261 Wu QF, Li Z, Chang LY, et al. Meta-analysis of the effects of exercise therapy and nutritional intervention on hemodialysis patients with sarcopenia [J]. Nursing and Rehabilitation, 2023, 22(5): 24-30.

262 Xie Caiyun, Chen Qiongmei, Zou De'e, et al. Effects of rehabilitation exercises on quality of life and sleep quality of maintenance hemodialysis patients [J]. Chinese Geriatric Medicine, 2023, 21(6): 148-51.

263 Xu Q. T. Effects of exercise intervention on maintenance hemodialysis patients [J]. Chinese Science and Technology Journal Database (Full Text Edition) Medicine and Health, 2023, (3): 0127-30.

264 Zhang Fan, Dong Yan, Zeng Zhen, et al. Effects of prone bicycle exercise in dialysis on exercise capacity of hemodialysis patients [J]. Evidence-Based Nursing, 2023, 9(11): 2037-42.

265 Zhang J. Effectiveness of exercise guidance combined with intensive skin management on maintenance hemodialysis patients [J]. Qingdao Medicine and Health, 2023, 55(6): 440-3.

266 Zhang L, Hou KL, Nie Zhaolan. Nursing effect of aerobic exercise based on safe heart rate in uremic maintenance hemodialysis patients [J]. Heilongjiang Traditional Chinese Medicine, 2023, 52(3): 331-3.

267 Zhang Xuan, Guo Yueyue, Zhou Kouxiang, et al. Evidence-based practice of exercise rehabilitation in maintenance hemodialysis patients [J]. China Blood Purification, 2023, 22(6): 472-6.

268 Zhou Xiaorui, Leng Yingjie, Fu Xiaorong, et al. Effects of appropriate exercise training during dialysis on dialysis adequacy and fatigue in hemodialysis patients [J]. China Medical Innovation, 2023, 20(20): 102-6.

269 Zhou YY. Analysis of the effect of exercise rehabilitation exercise on sleep quality and fatigue level of uremic hemodialysis patients [J]. Health Home, 2023, (4): 96-8.

270 Zhu Chan. Effects of Focused Empowerment Model Exercise Combined with Self-Efficacy Training on Exercise Self-Efficacy and Motor Function in Peritoneal Dialysis Patients [J]. Reflexology and Rehabilitation Medicine, 2023, 4(13): 83-6.

271 Zhu Guo-Ying. Application of exercise program in hemodialysis care for patients with end-stage renal disease [J]. Chinese Rural Medicine, 2023, 30(24): 65-6.

272 Zou, Chunbo, Su, Tingting, Zhang, Shuyan, et al. Exercise management combined with paricalcitol upregulates serum Klotho protein and improves secondary hyperparathyroidism [J]. China Blood Purification, 2023, 22(2): 114-8.

273 Chen Chaoyan, Chen Fang, Li Kaizhi. Analysis of the effects of acupressure combined with bed bicycle in the prevention and treatment of hypotension in maintenance hemodialysis patients [J]. China Science and Technology Journal Database Medicine, 2024, (6): 0177-80.

274 Chen P, Wu J, Liu LF, et al. Effects of Tai Chi Softball Exercise on Muscle Strength and Fatigue Status of Maintenance Hemodialysis Patients with Chronic Kidney Disease [J]. Contemporary Medicine, 2024, 30(3): 110-3.

275 Chen X. Analysis of the effect of leucovorin combined with intermittent exercise therapy on dialysis with hypotension in uremic patients [J]. Medical Theory and Practice, 2024, 37(11): 1964-5+70.

276 Ding Yunxi, Zhang Yu, He Xinying. An analysis of the effect of applying the health education model of Zhixinxing on maintenance hemodialysis (Mhd) patients [J]. Chinese Science and Technology Journal Database (Digest Edition) Medicine and Health, 2024, (3): 0062-5.

277 HAN Miaomiao, TIAN Mingjie, ZHAO Haihong, et al. Analysis of the effect of incremental resistance exercise combined with nutritional therapy in patients with end-stage renal disease receiving maintenance hemodialysis [J]. Journal of Clinical and Experimental Medicine, 2024, 23(3): 265-70.

278 Huang HY, Zhao HL, Cao XK, et al. Effects of moderate exercise on the quality of survival of maintenance hemodialysis patients [J]. China Science and Technology Journal Database Medicine, 2024, (5): 0065-8.

279 Huang QF. Effect of exercise therapy combined with paricalcitol in the treatment of MHD patients with secondary hyperparathyroidism and its effect on their FGF23 and Klotho protein levels [J]. Reflexology and Rehabilitation Medicine, 2024, 5(3): 61-4.

280 Jiang, Guanghui, Xu, Mingyue, Dai, Wenli, et al. Power orientation intervention combined with quantitative exercise guidance in elderly uremic maintenance hemodialysis patients [J]. Qilu Nursing Journal, 2024, 30(5): 30-4.

281 Lai Yanyang, Quan Minying. Effects of bicycle exercise and nutritional intervention on complications in maintenance hemodialysis patients [J]. Heilongjiang Medicine, 2024, 37(3): 682-4.

282 Li A-M, Zhou L-H. Exercise-cognitive dual-task exercise in hemodialysis patients with cognitive decline [J]. Nursing Research, 2024, 38(6): 1064-7.

283 Li Chunyan, Cao Tingting. Effects of multimodal exercise therapy on fatigue status and exercise capacity of maintenance hemodialysis patients [J]. Contemporary Nurses (Upper Ten Journal), 2024, 31(4): 21-4.

284 Li Shuyue, Sun Wanting, Gong Xiang, et al. Effects of cycling training and respiratory training on exercise tolerance, lung function and quality of survival in maintenance hemodialysis patients [J]. Chinese Journal of Rehabilitation Medicine, 2024, 39(5): 687-92.

285 Li WM, Tan JQ. Effects of exercise guidance on the quality of life of hemodialysis patients [J]. China Urban and Rural Enterprise Health, 2024, 39(1): 104-6.

286 Li, Yucui. Effects of an empowerment model-based exercise intervention on frailty in elderly hemodialysis patients [J]. Evidence-Based Nursing, 2024, 10(10): 1819-24.

287 Li Yucui, Tong Li, Li Yang, et al. The effect of physical exercise on patients' negative emotions in dialysis based on the Knowing, Believing and Doing model [J]. Journal of Nurse Advancement, 2024, 39(1): 71-6.

288 Liu Cong, Yang Jiju, Li Hongdian, et al. Net Meta-analysis of Different Exercise Modalities for the Treatment of Sleep Disorders in Hemodialysis Patients [J]. Tianjin Traditional Chinese Medicine, 2024, 41(5): 600-11.

289 Liu Feng, Qiu Mingtao, Liao Wen. Application of gabapentin combined with exercise training in patients with maintenance hemodialysis combined with restless legs syndrome [J]. China Medical Innovation, 2024, 21(10): 150-4.

290 Liu Yixiu, Deng Siyan, Luo Xiju, et al. Meta-analysis of the intervention effect of aerobic combined resistance exercise on hemodialysis patients [J]. Chongqing Medicine, 2024, 53(12): 1856-63.

291 Luo Lianhua, Cheng Lin. Effects of group acceptance and commitment therapy combined with aerobic exercise on hemodialysis patients [J]. Chinese and Foreign Medical Research, 2024, 22(10): 144-7.

292 Shi Haiyan, Zang Xiujuan, Chen Peng, et al. The effect of quantitative aerobic exercise under the guidance of cardiopulmonary exercise test on cardiopulmonary function and quality of life of hemodialysis patients [J]. Chinese Journal of Nephrology, 2024, 25(2): 142-5.

293 Song Q. Effects of cognitive-behavioral interventions on compliance behavior, exercise capacity, and risk events in hospitalized peritoneal dialysis patients [J]. Journal of Qiannan National Medical College, 2024, 37(1): 107-9.

294 Song Yanyun. Effects of dynamic resistance exercise training on renal function, microinflammatory status and quality of life in non-dialysis chronic kidney disease patients [J]. China Practical Medicine, 2024, 19(2): 158-61.

295 Su, H., Yang, H. F., Yu, M. H., et al. Application of Yoga Ball Low Intensity Exercise in Maintenance Hemodialysis Patients [J]. Chinese Science and Technology Journal Database (Full Text Edition) Medicine and Health, 2024, (1): 0040-3.

296 Wang G. Y. Effect of low-intensity aerobic training+ dietary management in uremic high-flux hemodialysis patients [J]. Clinical Medicine Research and Practice, 2024, 9(14): 165-8.

297 Wang Li-Qin, Li Kai, Cao Cheng-Cheng, et al. Effects of exercise education combined with aerobic pedaling exercise on negative emotions and sleep disorders in maintenance hemodialysis patients [J]. Journal of Weifang Medical College, 2024, 46(1): 23-6.

298 Wang Ruyan. Effects of aerobic exercise on maintenance hemodialysis patients [J]. Chinese Science and Technology Journal Database (Citation Edition) Medicine and Health, 2024, (2): 0063-7.

299 Wei Minghong. Effects of fine quality nursing care combined with incremental resistance exercise training on adverse emotions and fatigue in maintenance hemodialysis patients [J]. Reflexology and Rehabilitation Medicine, 2024, 5(2): 77-80.

300 Wu F. Analysis of the effect of individualized rehabilitation exercise training based on cardiopulmonary exercise test in maintenance hemodialysis patients [J]. Primary Medical Forum, 2024, 28(9): 70-2.

301 Wu Xiaomei, Wang Qiujing. Effects of low-intensity aerobic rehabilitation exercise on endovascular fistula maintenance care and nursing satisfaction in dialysis patients with diabetic nephropathy [J]. Chinese Health Care, 2024, 42(5): 129-31.

302 Xu Xuxiu, Qin Wenting, Su Xiaoxuan. Application of high-intensity interval exercise in maintenance hemodialysis patients [J]. Hebei Medicine, 2024, 46(9): 1315-9.

303 Zhang Guanglu, Jiao Xia, Wang Yannan. Effects of rehabilitation exercise training on renal function and urinary tgf-β1 and Iv-C levels in hemodialysis patients [J]. Clinical Medicine Progress, 2024, 14(6): 1105-10.

304 Zhang Xiaojiao. Effects of rehabilitation exercise training combined with quality nursing care on sleep quality of uremic hemodialysis patients [J]. China Science and Technology Journal Database Medicine, 2024, (3): 0140-3.

305 Zhao HF. Effects of arteriovenous endovascular fistula exercise on upper limb motor function and endovascular blood flow rate in dialysis patients [J]. China Medical Guide, 2024, 22(13): 96-9.

306 ZHAO Liang, YIN Wenyan, LING Shuxun, et al. Analysis of the effect of horizontal pedaling exercise in dialysis to assist blood purification treatment for maintenance hemodialysis patients [J]. Modern Biomedical Progress, 2024, 24(6): 1114-8.

307 Cao J. Synthesis of long-circulating SPIO liposomes and comparative study with SPIO in MRI lymphatic imaging [D]; Southern Medical University, Imaging Medicine and Nuclear Medicine, 2010.

308 Chang S.-L. Study on the effects of taurine on brain metabolism and mitochondria in rats with severe traumatic brain injury [D]; Tianjin Medical University, Biology ; Biochemistry and Molecular Biology, 2010.

309 Chen Xuebo, Hu Yuren, Wu Guangyu, et al. Effect of Astragalus injection combined with recombinant human erythropoietin on the immune function of erythrocytes in hemodialysis patients [J]. Chinese Journal of Continuing Medical Education, 2010, 33(22): 24-6.

310 CHENG Jianping, LIU Tao, JING Jinzhi, et al. Application of airtight pre-flush dialyzer in hemodialysis [J]. International Journal of Medicine and Health, 2010, 16(18): 2218-20.

311 FANG Jianqiao, LIANG Yi, WANG Cunxin, et al. Effects of transcutaneous electrical stimulation of acupoints on plasma amino acids and extracellular fluid 5-HT content in the dorsal nucleus of the middle suture of rats exercising in table running [J]. Chinese Journal of Sports Medicine, 2010, 29(3): 309-12,20.

312 He Li. Efficient preparation of recombinant human sCR1 functional domains SCR15-18 and their protective effects on cerebral ischemia/reperfusion injury [D]; Third Military Medical University, Microbiology, 2010.

313 Hu Feng. Effects of blockade-reducing agents on hemodynamics of abdominal aorta and hindlimb arteries and skeletal muscle perfusion in rats [D]; Southern Medical University, Internal Medicine (Cardiovascular Disease), 2010.

314 Huang, H. L., Chang, S. L., Zhang, X. B., et al. Effects of taurine on the metabolism and respiratory function of brain tissues in rats with traumatic brain injury; proceedings of the Second National Symposium on Infection in Trauma and Critical Illness of the Chinese Medical Association, Zhenjiang, Jiangsu, F, 2010 [C].

315 Jin Lingwei, Zheng Yu, Ye Hanyang, et al. Effects of lycopene on microinflammation and oxidative stress in elderly maintenance hemodialysis patients [J]. Pharmacology, 2010, 22(1): 82-4.

316 Li L, Liu ML, Li Y, et al. Effects of reduced glutathione combined with hemodialysis filtration on the microinflammatory status of maintenance hemodialysis patients [J]. Journal of Clinical Internal Medicine, 2010, 27(11): 757-9.

317 Li MX. Plasma dialysis filtration in sepsis [D]; Fudan University, Internal Medicine (Nephrology), 2010.

318 Li Q. Dynamic changes of DA, 5-HT and their metabolites in striatal microdialysis fluid of rats during recovery from exhaustive exercise [D]; Nanjing Institute of Physical Education, Exercise Human Science, 2010.

319 Lin, B.-Q. Synthesis of long-circulating SPIO-PBCA-PEG nanoparticles and their study in MR imaging of rat liver tumors [D]; Southern Medical University, Imaging Medicine and Nuclear Medicine, 2010.

320 Liu DD. Pharmacokinetics of levofloxacin hydrochloride in rat pancreas by microdialysis [D]; Second Military Medical University, Surgery (General), 2010.

321 Liu Yuhong, Zou Yukun, Zhang Yi, et al. Comparison of the efficacy and safety of linezolid and vancomycin against methicillin-resistant Staphylococcus aureus bloodstream infection [J]. Comparison of the efficacy and safety of linezolid and vancomycin in methicillin-resistant Staphylococcus aureus bloodstream infections [J].

322 Ma Zhichao. An exploratory study on the application of exercise-loaded nanoparticle-targeted drug delivery system for tumor therapy [D]; Yangzhou University, Sports Human Science, 2010.

323 Nan J. Intervention study of hemopexin injection on oxidative stress and microinflammatory status in maintenance hemodialysis patients [D]; Zhengzhou University, Epidemiology and Health Statistics, 2010.

324 Qi Min. Experimental study of adenovirus-mediated canstatin gene inhibition in hepatocellular carcinoma [D]; Zhengzhou University, Clinical Medicine (Internal Medicine and Gastroenterology), 2010.

325 Qi Leilei. Clinical study on the treatment of heart failure with cardiac yang deficiency by ginseng and yi qi strong heart soup combined with blood ultrafiltration [D]; Beijing University of Traditional Chinese Medicine, Traditional Chinese Medicine, 2010.

326 QIN Jianhua, LI Ying, YING L. Observation on the efficacy of sodium deoxyribonucleotide injection in the treatment of acute kidney injury [J]. Western Medicine, 2010, 22(8): 1482-3.

327 Song Suhua, Wang Zhongfu, Meng Shuhong, et al. Irbesartan combined with traditional Chinese medicine in the treatment of diabetic nephropathy [J]. Journal of Gansu College of Traditional Chinese Medicine, 2010, 27(6): 37-40.

328 Su Jingwen. Experimental study on the alteration of urinary metabolites in rats with early diabetic nephropathy (stage II) and the interventional effect of self-proposed Sanqi Tang [D]; Hebei Medical University, Fundamentals of Integrative Medicine, 2010.

329 Tang Aidang, Lin Hongchu, Jiang Jie, et al. Observation on the effect of aerobic exercise combined with dietary intervention on controlling volume load in peritoneal dialysis patients [J]. Clinical Nursing Journal, 2010, 9(4): 15-7.

330 TANG DONGXING, ZENG GAOFENG, GAOFENG Z. Effects of exercise intervention on the quality of survival of maintenance hemodialysis patients [J]. Xiang South College Journal (Medical Edition), 2010, 12(3): 18-20.

331 Tian Ru, Sun Yuming, Guo Yidan, et al. Clinical analysis of parathyroid hormone levels in patients on maintenance hemodialysis [J]. International Journal of Transplantation and Blood Purification, 2010, 8(3): 33-7.

332 Wang R. The therapeutic efficacy of flufenacet against interstitial fibrosis in UUO rats [D]; Zhongnan University, Internal Medicine (Nephrology), 2010.

333 Wei, F. N.. Mechanistic study on the treatment of chronic renal failure with qi and blood-activating traditional Chinese medicine [D]; Guangzhou University of Traditional Chinese Medicine, Department of Nephrology, Chinese Medicine, 2010.

334 Wu Lei, Li Daxian, Zhang Xiaomin, et al. A comparative study of the efficacy of intravenous iron sucrose and oral iron in the treatment of renal anemia [J]. China Medical Guide, 2010, 8(31): 73-4.

335 Xie H-Ch. Effect of vitamin C dialysate on secondary hyperparathyroidism [D]; Zhengzhou University, Blood Purification, 2010.

336 Xu B, Jin B, Ji DX, et al. Effect of bicarbonate citrate dialysis solution on hypertension in hemodialysis patients [J]. Journal of Nephrology and Dialysis Kidney Transplantation, 2010, 19(5): 424-9.

337 Xu Hualei. Study on the protective mechanism of DFP on the reproductive system of aluminum-contaminated rats [D]; Shandong University, Labor Hygiene and Environmental Hygiene, 2010.

338 Xu JY. Protective effect of Astragalus injection on angiotensin II-induced peritoneal fibrosis in rats [D]; Zhengzhou University, Clinical Medicine (Internal Nephrology), 2010.

339 Xu M L, Hu Y W, Ding D L, et al. Effects of antioxidant nutrients on the microinflammatory state of uremic patients [J]. Chinese Family Medicine, 2010, 13(26): 2945-7.

340 Yin Li. Effects of different dosing regimens of intravenous iron on anemia and oxidative stress in maintenance hemodialysis patients [D]; South Central University, Nephrology, 2010.

341 Yu Zu-Mei, Wen Xiu-Fang, Zhu Yong-Mei, et al. Application of exercise relearning method in exercise training for hemodialysis patients [J]. Journal of Nursing, 2010, 25(19): 79-81.

342 Yuan Jianghong, Wang Chengkai, Yan Wei, et al. Effect of intravenous iron supplementation combined with vitamin E on the efficacy of anemia and oxidative stress in hemodialysis patients [J]. Modern Journal of Laboratory Medicine, 2010, 25(5): 114-7.

343 Zhang Jianjun, Lv Weiquan, Huang Huichuan, et al. The efficacy of urethane granules combined with traditional Chinese medicine retention enema in the treatment of chronic renal failure [J]. Journal of Practical Medicine, 2010, 26(23): 4425-7.

344 ZHANG Ori, GUO Zhiyong, ZHI-YONG G U O. Relationship between nitric oxide and peritoneal vascular proliferation in peritoneal dialysis [J]. Journal of Clinical Nephrology, 2010, 10(5): 229-31,seal 2.

345 Zhang, L., Wu, B., Tang, Q., et al. Effects of trimetazidine on oxidative stress in maintenance hemodialysis patients [J]. China Clinics, 2010, 26(3): 242-5.

346 Zhang Miao, Tao Nana, Jiang Chunming, et al. Effects of tanshinone IIA on peritoneal histology and oxidative stress changes induced by peritoneal dialysis fluid in rats [J]. Pharmaceutical Biotechnology, 2010, 17(5): 412-6.

347 Zhang YJ. Effect of mannitol on MDA, AOPP and NO in hypertensive patients on maintenance hemodialysis [D]; Zunyi Medical College, Internal Medicine (Nephrology), 2010.

348 Zhang Z R, Gong Y H, Yang H, et al. Effect of hemoperfusion combined with hemodialysis on inflammatory cytokines in patients with severe organophosphorus poisoning [J]. Chinese Ethnic and Folk Medicine, 2010, 19(6): 21-2.

349 Zhao Li, Ruan Hualing, Chen Huaxi, et al. Comparison of the efficacy of different blood purification modalities in the treatment of multiple organ dysfunction syndrome [J]. China Blood Purification, 2010, 9(10): 542-5.

350 ZHAO PINGGUE, LIU XIAO, XIAO L. Extraction and purification of digitonin polysaccharide and its effect on blood glucose in diabetic mice [J]. Straits Pharmacology, 2010, 22(9): 29-32.

351 KERSTIN D R O H, MENGISTU A, BOLDT J, et al. Comparison of renal integrity when sevoflurane is administered via an anesthesia maintenance device versus propofol given intravenously for sedation in the intensive care unit [J]. Anesthesia and Analgesia, 2010, 06(4): 46-52.

352 Buheliqi Maimeti. Effect of continuous hemodialysis filtration on monocyte apoptosis in dogs with multiple organ dysfunction [D]; Xinjiang Medical University, Internal Medicine (Nephrology), 2011.

353 Chen JH, Yu C, Liu J, et al. Effects of continuous veno-venous hemodialysis filtration on the clearance of different solutes in dogs with multiple organ dysfunction syndrome [J]. Chinese Journal of Nephrology, 2011, 27(2): 124-7.

354 Chen Jian'e, Xu Jinhua, Wu Sijun, et al. Effect of intravenous iron on anemia, inflammatory response and oxidative stress in maintenance hemodialysis patients [J]. China Modern Physician, 2011, 49(16): 55-7.

355 Cheng Fafeng. Therapeutic effects of purified Qingkailing injection on cerebral ischemia and the regulatory mechanism of excitotoxic cascade injury [D]; Beijing University of Traditional Chinese Medicine, Clinical Basis of Traditional Chinese Medicine, 2011.

356 Cui K. In situ study of the protective effect of block-reducing agents on canine ischemic myocardium [D]; Southern Medical University, Internal Medicine (Cardiovascular Disease), 2011.

357 Gao Jiayuan. A metabolomic study on the efficacy of small molecule solute clearance in high and low flux hemodialysis patients based on GC-TOF-MS platform [D]; Shanghai Jiao Tong University, Internal Medicine (Nephrology), 2011.

358 Gao Liqun. Effects of different doses of folic acid on homocysteine in hemodialysis patients; proceedings of the 2011 Chinese Medical Association Nephrology Section Blood Purification Forum and Heilongjiang Provincial Medical Association Nephrology Annual Meeting, Harbin, F, 2011 [C].

359 Gao Min, Liu Hong, Liu Bicheng, et al. Evaluation of surgical efficacy of carpal tunnel syndrome in patients on maintenance hemodialysis [J]. Modern Medicine, 2011, 39(6): 635-7.

360 Gong L B. Clinical observation of iron sucrose combined with erythropoietin in the treatment of renal anemia in hemodialysis patients [J]. China Medical Guide, 2011, 9(20): 49-50.

361 Gong JH, Xu Q, Yuan H, et al. Therapeutic efficacy of continuous blood purification tandem hemoperfusion in the treatment of severe fish bile poisoning [J]. Journal of Hebei Medical University, 2011, 32(3): 321-3.

362 Gu Lijuan. Effect of sodium metasilicate on reactive oxygen species production in peripheral blood leukocytes of hyperlipidemic rabbits [D]; Hebei Medical University, Pathology and Pathophysiology, 2011.

363 Guo HW. The efficacy of combining low-dose growth hormone and insulin-like growth factor in the treatment of malnutrition in hemodialysis patients [D]; Hebei Medical University, Internal Medicine, 2011.

364 HAN Zhiwu, YAN Lizhong, LI Jing, et al. Compound α-keto acids combined with antioxidants for the treatment of chronic dialysis hypotensive state [J]. Chinese Journal of Hypertension, 2011, 19(2): 120-4.

365 He Ji-Qing. Effect of reduced glutathione on the expression of 8-OHdG, a marker of peritoneal oxidative damage caused by high glucose in rats [D]; Guiyang Medical College, Internal Medicine (Nephrology), 2011.

366 Hou, Xiaorui. Preparation and preliminary application of monoclonal antibodies to late oxidized protein products [D]; Southern Medical University, Immunology, 2011.

367 JIA Meng, SUN Weiming, YU Ming S. Therapeutic effect of online hemodialysis filtration combined with hemodialysis on renal anemia [J]. Shandong Medicine, 2011, 51(13): 91-2.

368 Jian GD. Construction and evaluation of a novel human cell hybrid bioartificial liver [D]; Southern Medical University, General Surgery (Hepatobiliary Surgery), 2011.

369 Ke YW. Effects of Methylprednisolone Pretreatment on Lung Permeability and Alveolar Surface Active Substances in Rabbit Recurrent Pulmonary Edema [D]; Southern Medical University, Anesthesiology, 2011.

370 Li GH. Effect of low molecular heparin calcium on secondary hyperparathyroidism in maintenance hemodialysis patients [D]; Zhengzhou University, Urology, 2011.

371 Li GH, Zhao XG, Zhang HT, et al. Intervention effect of vitamin C dialysis solution on arterial calcification in maintenance hemodialysis patients [J]. Chinese Family Medicine, 2011, 14(17): 1911-3.

372 LI Guohui, ZHAO Xianguo, XIAN Guo Z. Effect of low molecular heparin calcium on secondary hyperparathyroidism in maintenance hemodialysis patients [J]. Journal of Practical Medicine, 2011, 27(12): 2241-3.

373 Li Huipeng. Effects of bone marrow mesenchymal stem cells on CD4+CD25+ regulatory T cells in rats with severe acute pancreatitis and the combination of Chinese and Western medicine [D]; Dalian Medical University, Clinical Integrative Medicine, 2011.

374 Li J. Preparation of chitosan nanoparticles of loratrex hydrochloride and detection of their antitumor activity [D]; Southern Medical University, Oncology, 2011.

375 Li Q, Yu T-S, Liu L-Q, et al. The therapeutic roles of medication, psychology, sleep and other multifactors in uremic peritoneal dialysis with refractory hypertension; proceedings of the 2011 Chinese Medical Association Nephrology Section Blood Purification Forum and the Annual Meeting of the Heilongjiang Provincial Medical Association Nephrology, Harbin, F, 2011 [C].

376 Li YF, Gai BY, Chen JH, et al. Continuous hemodialysis filtration management and effect observation on the application of multiorgan dysfunction syndrome in test dogs [J]. Journal of Xinjiang Medical University, 2011, 34(5): 477-81.

377 Li Yuxiu. Effect of nursing intervention on the quality of life of maintenance hemodialysis patients [J]. Jilin Medical Science, 2011, 32(21): 4498-9.

378 Li Yuan. Study on factors related to nutrition and mineral metabolism in patients with chronic renal failure [D]; Qingdao University, Internal Medicine (Nephrology), 2011.

379 Liang, H. L.. Preparation and in vitro characterization of tumor-targeting hyaluronic acid-coupled chitosan nanoparticles [D]; Southern Medical University, Cardiothoracic Surgery, 2011.

380 Liang Yanping, Xiao Cuiyan, Hou Yongmei, et al. Effects of exercise therapy on sleep quality of maintenance hemodialysis patients [J]. Journal of Guangdong Medical College, 2011, 29(6): 618-20.

381 Liang Yanping, Xiao Cuiyan, Wu Jiaohua, et al. Effects of home care on quality of life of hemodialysis patients [J]. International Journal of Nursing, 2011, 30(11): 1633-5.

382 Liu Na. Intraocular pharmacokinetics and in vitro antibacterial activity of ticlopidine against MRSA [D]; Zhengzhou University, Pharmacology, 2011.

383 LIU Peng, HE Yuezhong, ZHANG Xigang, et al. Study on the efficacy of hemoperfusion combined with continuous veno-venous hemofiltration in acute paraquat poisoning [J]. Chinese Journal of Labor Health and Occupational Diseases, 2011, 29(4): 266-9.

384 Liu Yixian. Role of GLT-la, a splice variant of GLT-1, in the induction of cerebral ischemic tolerance [D]; Hebei Medical University, Pathology and Pathophysiology, 2011.

385 Lu Rui. Preventive effect of small doses of furosemide on contrast nephropathy based on adequate hydration [D]; Hebei Medical University, Internal Medicine, 2011.

386 Lu, Chuan-Dong. Protective effect of astragaloside on contrast nephropathy in diabetic rats and its mechanism [D]; Hebei Medical University, Pharmacology, 2011.

387 Luo Xingjun. Experimental study on the therapeutic effect of intraperitoneal resuscitation on rats with infectious shock [D]; Three Gorges University, Surgery, 2011.

388 Ma J, Gu PY, Yu CH, et al. Clinical study on the effects of ustekin on intra-abdominal hypertension and intestinal mucosal function in elderly patients with severe pancreatitis [J]. Chinese Clinical Pharmacology and Therapeutics, 2011, 16(1): 93-7.

389 Ma T. Effects of acute ischemic kidney injury on lungs in rats [D]; China Medical University, Emergency Medicine, 2011.

390 Peng HG. The effect of Tong Wei Oral Liquid on the function of endovascular fistula after autologous arteriovenous endovascular fistula surgery [D]; Guangzhou University of Traditional Chinese Medicine, Chinese Medicine, 2011.

391 PENG Wei, ZHANG Conghua, YONG-HUA Z. Impact of Clinical Pathway Health Education on Hemodialysis Patients' Compliance Behavior [J]. Nursing Practice and Research, 2011, 8(8): 100-2.

392 Pu Chao, Chen Yongzhong, Zou Deping, et al. Interventional effects of Salvia divinorum polyphenolates on oxidative stress in maintenance hemodialysis patients [J]. Western Medicine, 2011, 23(2): 278-9,82.

393 Pu Chao, Chen Yongzhong, Zou Deping, et al. Intervention effects of Salvia divinorum polyphenolates on oxidative stress in maintenance hemodialysis patients [J]. Medical Information (Upper Ten Journal), 2011, 24(3): 1352-3.

394 Qiu Shoutao. Changes of amino acid neurotransmitters in striatal microdialysis fluid of rats during recovery from exhaustive exercise [D]; Nanjing Institute of Physical Education, Exercise Human Science, 2011.

395 Shen Wanqin. Expression and significance of HIF-1α and CTGF in peritoneal tissue of peritoneal dialysis rats [D]; Zhengzhou University, Internal Medicine (Nephrology), 2011.

396 Shen Wanqin, Wu Ge, Zhao Zhanzheng, et al. Expression and significance of HIF-1α and CTGF in peritoneal tissues of peritoneal dialysis rats; proceedings of the 2011 Chinese Medical Association Nephrology Branch Blood Purification Forum and Heilongjiang Medical Association Nephrology Annual Meeting, Harbin, F, 2011 [C].

397 Sun Jingshu. Cordyceps sinensis inhibits renal tubulointerstitial fibrosis by inducing heme oxygenase-1 expression [D]; Shanghai Jiao Tong University, Internal Medicine (Nephrology), 2011.

398 Sun Xiaofang. Effects of Shouwu formula on blood-striatal levodopa pharmacokinetics and neurotransmitters in rats with Parkinson's disease [D]; Beijing University of Traditional Chinese Medicine, Fundamentals of Integrative Medicine, 2011.

399 Tang, Dongxing. Effects of rehabilitation exercise on cardiac function and quality of life of maintenance hemodialysis patients [D]; Nanhua University, Internal Medicine, 2011.

400 Wang Baoxing. Effects of Megsin on ERK and ECM in renal tissues of diabetic mice [D]; Hebei Medical University, Internal Medicine, 2011.

401 Wang, Bingyue. Effects of different dialysis modalities on advanced oxidized protein products and analysis of related factors [D]; Tianjin Medical University, Clinical Medicine; Internal Medicine Nephrology, 2011.

402 Wang BX. Isolation and preliminary characterization of goat placental peptides and their nutritional, immunological and antioxidant effects [D]; Shandong Agricultural University, Clinical Veterinary Medicine, 2011.

403 Wang Ge, Tang Feng, Zhang Ping, et al. Observations on the efficacy of tanshinone IIA on diabetic maintenance hemodialysis with ischemic heart disease patients [J]. Southwest National Defense Medicine, 2011, 21(3): 256-8.

404 Wang Li-Hua. Effects of high-throughput dialysis on vasoactive substances and blood pressure in dialysis patients [D]; Hebei Medical University, Internal Medicine, 2011.

405 Wang WJ. Study on the efficacy of different blood purification modalities in acute paraquat poisoning [D]; Hebei Medical University, Emergency Medicine, 2011.

406 XI Xiaoli. Experimental study of polyethylene glycol for fluid resuscitation in endotoxemic rats [D]; Southern Medical University, Anesthesiology, 2011.

407 XIANG Jing, MA Zhifang, ZHI Fang M. Lower limb strength training on reducing the incidence of hypotension in maintenance hemodialysis patients; proceedings of the 2011 Chinese Medical Association Nephrology Branch Blood Purification Forum and the Annual Meeting of Heilongjiang Medical Association Nephrology, Harbin, F, 2011 [C].

408 XU Chuan-Wen, XU Yan-Mei, YAN-MEI X U. Effects of hemoperfusion on homocysteine and oxidative stress status in uremic patients [J]. Chinese Family Medicine, 2011, 14(30): 3518-9.

409 Xu ZZ, Pu CH, Chen YZ, et al. Intervention effects of leucovorin and iron sucrose on oxidative stress in renal anemia in hemodialysis patients [J]. Western Medicine, 2011, 23(2): 262-4.

410 Xu ZZ, Pu CH, Chen YZ, et al. Interventional effects of leucovorin and iron sucrose on renal anemia and oxidative stress in hemodialysis patients; proceedings of the 2011 Chinese Medical Association Nephrology Branch Blood Purification Forum and Annual Meeting of Heilongjiang Medical Association Nephrology, Harbin, F, 2011 [C].

411 Yao L, Wei DD, Ge DM, et al. Timosartan improves microinflammation and oxidative stress in maintenance hemodialysis patients [J]. China Blood Purification, 2011, 10(8): 426-8.

412 Yao L, Yang Shuang, Du Yinke, et al. Clinical study of dextrose hydroxide iron in the treatment of renal anemia [J]. Practical Medicine and Clinic, 2011, 14(2): 126-8.

413 Yu T. Preliminary Preparation and Evaluation of Biotinylated Liposomal SPIO Nanoparticles, a Novel Active Targeted MR Contrast Agent [D]; Southern Medical University, Imaging Medicine and Nuclear Medicine, 2011.

414 Zhang D, Wang Y, Shi Z, et al. Effects of moxibustion on peritoneal tissue morphology and VEGF, NO in peritoneal dialysis rats; proceedings of the 2011 Chinese Acupuncture and Moxibustion Society Annual Meeting, Beijing, F, 2011 [C].

415 Zhang D, Wang Y, Shi Z, et al. Effects of moxibustion on peritoneal tissue morphology and VEGF, NO in peritoneal dialysis rats; proceedings of the 2011 Symposium on the Fundamentals of Moxibustion Actions and the Laws of Application, Shiyan, Hubei, F, 2011 [C].

416 Zhang D, Wang Y, Shi Z, et al. Effects of moxibustion on peritoneal tissue morphology and VEGF and NO in peritoneal dialysis rats [J]. Shanghai Journal of Acupuncture and Moxibustion, 2011, 30(4): 268-71.

417 Zhang, Dongliang. Role of Apelin-APJ system in the development of diabetic nephropathy [D]; Shanghai Jiao Tong University, Internal Medicine (Nephrology), 2011.

418 Zhang F. Mechanisms of pre-ischemic exercise training to attenuate cerebral ischemia-reperfusion injury in rats via glutamate system and ERK1/2 [D]; Fudan University, Rehabilitation Medicine and Physical Therapy, 2011.

419 Zhang JY. Nursing care and health education of elderly hemodialysis patients during dialysis [J]. China Medical Guide, 2011, 9(20): 167-9.

420 Zhang L, Tian Y, Yu XK, et al. Effects of adding tanshinone to peritoneal dialysis fluid on oxidative stress in rats [J]. Western Medicine, 2011, 23(1): 12-6.

421 Zheng DZ. Effects of blockade-reducing agents on skeletal muscle perfusion in rats with chronic ischemic hindlimb [D]; Southern Medical University, Internal Medicine (Cardiovascular Disease), 2011.

422 Zhu H-T. Cranial multimodal monitoring of traumatic cerebral hemorrhage in minipigs under simulated rapid entry plateau environment [D]; Third Military Medical University, Surgery (Neurology), 2011.

423 Zou Chuan, Wu Xiuqing, Lin Qizhan, et al. The effect of comprehensive therapy of promoting internal organs and draining turbidity on functional constipation in hemodialysis patients [J]. Chinese Journal of Traditional Chinese Medicine, 2011, 26(4): 875-8.

424 Zou, Guimian. A series of studies on community and clinical hyperuricemia and its related diseases [D]; Southern Medical University, Nephrology, 2011.

425 NUSSBAUM A, GARCIA R. Physical therapy effects on exercise capacity in hemodialysis patients: preliminary results of the Promoting Kidney Disease Rehabilitation Program study; proceedings of the 6th Beijing International Rehabilitation Forum, Beijing, F, 2011 [C].

426 Bu Xunya. Treatment of chronic renal insufficiency in 36 cases with renal failure [J]. China Traditional Chinese Medicine Modern Distance Education, 2012, 10(12): 29-.

427 Chang Jing, Song Xiuqin, Qiu Xiaojun, et al. Effects of hyperbaric oxygen on cognitive function recovery in patients with uremia complicating stroke on maintenance hemodialysis [J]. Chinese Journal of Family Medicine, 2012, 11(6): 459-61.

428 CHEN Sijie, GONG Baowen, CHEN Jiansheng, et al. Clinical Observation on Chinese Medicine Diagnosis and Nutrition with Health Education in the Treatment of Non-Dialysis Patients with Chronic Renal Failure [J]. New Chinese Medicine, 2012, 44(7): 47-9.

429 Chen, Xue-Ling. Effect of nursing intervention on the psychology of uremic hemodialysis patients [J]. Journal of Nursing Advancement, 2012, 27(9): 837-9.

430 Chen, Yue-Mei. Clinical and basic research on the relationship between hydrogen sulfide and refractory hypertension combined with maintenance hemodialysis [D]; Fudan University, Internal Medicine (Nephrology), 2012.

431 Cheng L. Clinical observation on the treatment of peritoneal dialysis gastrointestinal dysfunction with spaced ginger moxibustion [D]; Hubei University of Traditional Chinese Medicine, Integrative Medicine Clinic, 2012.

432 Cui X. Aluminum exposure interferes with learning memory and effects on Ras/Raf/ERK and CREB in rat hippocampus [D]; China Medical University, Basic Medicine; Biochemistry and Molecular Biology, 2012.

433 Shan Y. Construction of Plasmodium falciparum transmission-blocking vaccine candidate antigen P48 and study of its effect mechanism [D]; China Medical University, Basic Medicine; Immunology, 2012.

434 Dong Chunxia, Liu Bing, Niu Kai, et al. Effect of vitamin E combined with high flux dialysis on oxidative stress in MHD; proceedings of the Chinese Academy of Engineering, Department of Medicine and Health, Frontier Forum of Nephrology, the 13th Annual Meeting of North China and Beijing Nephrology, Beijing, F, 2012 [C].

435 Dong Shuying. Observations on the effects of different doses of simvastatin in patients with EPO resistance [D]; Harbin Medical University, Internal Medicine (Nephrology), 2012.

436 FAN X P, HUA J, JIAN H. Study related to the effect of intravenous iron on oxidative stress in maintenance hemodialysis patients [J]. Chinese and Foreign Medicine, 2012, 31(5): 43-4.

437 Fang Qin. Effect of ultrasound monitoring of polyethylene oxide on hepatic and renal hemodynamics in endotoxin-shocked rabbits [D]; Southern Medical University, Anesthesiology, 2012.

438 Feng Liping, Li Juanjuan, Liu Tongqiang, et al. Reversal of left ventricular hypertrophy by cloxartan in relation to inflammation and oxidative stress in maintenance hemodialysis patients [J]. China Blood Purification, 2012, 11(8): 421-4.

439 Yung SJ. Role of GLT-1 in intermittent low-pressure hypoxic preconditioning and cerebral ischemic preconditioning [D]; Hebei Medical University, Pathology and Pathophysiology, 2012.

440 GUAN Li, ZHU Mingxia, ZHAO Jinyuan, et al. Characteristics of changes in dopamine-like transmitters in the hippocampal region of the brain of rats with acute CO poisoning [J]. Chinese Journal of Industrial Medicine, 2012, 25(2): 83-6,seal 3.

441 Hu, Eddie. Exploration of factors related to the occurrence of adverse reactions in bee-acupuncture therapy [D]; Guangzhou University of Traditional Chinese Medicine, Acupuncture and Massage, 2012.

442 Hu ZH. Experimental study on the effect of polyethylene oxide on the microcirculation of spinal oblique muscle in rats with septicemia shock [D]; Southern Medical University, Anesthesiology, 2012.

443 Huang Ailing, Zhang Suozhen, Song Fen, et al. Effect of aerobic exercise on fatigue status of peritoneal dialysis patients [J]. Jilin Medical Science, 2012, 33(6): 1149-50.

444 Chun Jiang. Discussion on the application of PBL teaching mode in the health education of dialysis patients [J]. Journal of Qiqihar Medical College, 2012, 33(15): 2109-10.

445 Jiang Yafen. Alteration of indolephenol sulfate blood concentration in renal failure and its degradation by lactobacillus genetically engineered bacteria [D]; Zhongnan University, Clinical Medicine (Internal Medicine), 2012.

446 Leng Chengxiang. Study on the intervention effect of aerobic exercise on maintenance hemodialysis patients [D]; Tianjin Medical University, Clinical Medicine; Nursing, 2012.

447 Li, B. Pigeon. Preparation of L-selectin ligand-targeted USPIO-PBCA nanoparticles and their MRI application in lymph nodes [D]; Southern Medical University, Imaging Medicine and Nuclear Medicine, 2012.

448 Li B. Relationship between serum matrix metalloproteinase 10 and atherosclerosis in patients with end-stage renal disease [D]; Tianjin Medical University, Clinical Medicine; Surgery (Urology), 2012.

449 Li Jianying, Huang Yanlin, Teng Yanjuan, et al. Effect of exercise training on symptomatic hypotension in uremic hemodialysis patients [J]. Primary Medical Forum, 2012, 16(24): 3139-40.

450 Li, Dongli. Preparation of molecular probes targeting tumor mesenchymal fibronectin and experimental study of magnetic resonance imaging [D]; Southern Medical University, Imaging Medicine and Nuclear Medicine, 2012.

451 Liu Yuhong, Zou Rikun, Zhang Yi, et al. Comparison of the efficacy and safety of linezolid and vancomycin against methicillin-resistant Staphylococcus aureus bloodstream infections; proceedings of the 2012 Army Critical Care Medicine Forum, Beijing, F, 2012 [C].

452 Lu Guan Xie. Clinical research on the treatment of sepsis with blood purification without the use of modality [J]. Modern Diagnosis and Treatment, 2012, 23(9): 1467-8.

453 Luo WF. Application of real-time 3D echocardiography to evaluate left ventricular function and mechanical synchronization in uremic myocardial damage [D]; Hebei Medical University, Imaging Medicine and Nuclear Medicine, 2012.

454 Ma Wenlu, Xu Mifeng, Miao Xiaomei, et al. Clinical study on the effect of Fxclass high flux hemodialyzer on oxidative stress in uremic dialysis patients [J]. International Journal of Transplantation and Blood Purification, 2012, 10(1): 36-41.

455 Mao Guiyuan. Experimental study on lipid-lowering and hepatoprotective effects of red snow tea and its polysaccharides [D]; Guangxi Medical University, Pharmacology, 2012.

456 Min M, Zuo F, Wang L, et al. Effects of physical exercise on the quality of life of renal transplant recipients [J]. Chinese Tissue Engineering Research, 2012, 16(31): 5733-6.

457 Pan Xiaohong, Piduo Bo, He Aijun, et al. Efficacy of methylcobalamin combined with erythropoietin in the treatment of patients with combined peripheral neuropathy on maintenance hemodialysis [J]. Chinese Medical Journal, 2012, 14(8): 1136-8.

458 Qiu Shoutao, Zhang Yunkun, Jiang Xiaoling, et al. Neurotransmitter changes and central fatigue in striatal microdialysis fluid of exercising rats; proceedings of the Ninth Universiade of the People's Republic of China and scientific paper presentation, Tianjin, F, 2012 [C].

459 Shen Haiyan. Effects of home aerobic exercise on physical and mental health of hemodialysis patients [D]; Nanhua University, Nursing, 2012.

460 SUN YUAN, GUO RUI MIN, RUI MIN G. Clinical observation on the antioxidant effect of dialysis membrane [J]. Journal of Hebei Union University (Medical Edition), 2012, 14(4): 548-9.

461 Tang F. Effects of different doses of branched-chain amino acids on hypothalamic 5-hydroxytryptamine and its precursors in exercising rats [D]; Nanjing Institute of Physical Education, Exercise Human Science, 2012.

462 Teng Xuemei, Zhang Guangcai, Wang Huixin, et al. Study on the effect of leucovorin on microinflammatory status of maintenance hemodialysis patients [J]. Journal of Xuzhou Medical College, 2012, 32(6): 382-4.

463 Wang Chunzhang. Interventional effects of curcumin pretreatment on renal injury after extracorporeal circulation in rats [D]; Second Military Medical University, Thoracic Surgery, 2012.

464 Wang, Tao. Effects of valsartan on microinflammation and lung function in maintenance hemodialysis patients [D]; Southern Medical University, Respiratory Pathology, 2012.

465 Wang Genbao. Effects of pre-administration of bumetanide on focal cerebral ischemia-reperfusion injury in rats [D]; Guangzhou Medical University; Guangzhou Medical College, Anesthesiology, 2012.

466 Wang H, Zhao ZZ, Shen WQ, et al. Expression of HIF-1α and CTGF in peritoneal tissue of peritoneal dialysis rats [J]. Journal of Zhengzhou University (Medical Edition), 2012, 47(2): 205-8.

467 Wang Lei. Clinical analysis of continuous blood purification for the treatment of diabetic nephropathy ketoacidosis combined with acute renal failure [J]. Chinese and Foreign Medicine, 2012, 31(30): 38-9.

468 Wang MJ. A series of studies on phosphorus metabolism disorders in maintenance hemodialysis patients [D]; Fudan University, Internal Medicine (Nephrology), 2012.

469 Wu L-You, Li Shun-Min, Sun Hui-Li, et al. Effect of renal failure formula on microinflammatory status in patients with malnutrition with moist heat evidence on maintenance hemodialysis [J]. New Chinese Medicine, 2012, 44(1): 48-50.

470 Wu, L. Y.. Pharmacological study and safety evaluation of trauma coating agent [D]; Hebei Medical University, Pharmacology, 2012.

471 Wu Shengtian. Experimental study on the mechanism of neuroprotective effect of electrical stimulation of the thalamic floor nucleus in animals with Parkinson's disease [D]; Capital Medical University, Surgery (Neurosurgery), 2012.

472 Xia Xuan. Effects of reduced glutathione on the expression of 8-hydroxydeoxyguanosine (8-OHdG) and its repair enzyme 8-hydroxyguanine-DNA glycosylase (rOGG1) in high glucose-induced rat peritoneal mesothelial cells [D]; Guiyang Medical College, Internal Medicine (Nephrology), 2012.

473 Jing Xu, Jie Tian, Jihong Zhao, et al. Evaluation of the efficacy of low molecular heparin versus normal heparin in patients undergoing maintenance hemodialysis; proceedings of the Chinese Academy of Engineering, Department of Medicine and Health, Frontier Forum of Nephrology, the Thirteenth Annual Meeting of Nephrology in North China and Beijing, Beijing, F, 2012 [C].

474 Yang L, Wang M, Pan J S, et al. A randomized and multicenter controlled clinical study of intravenous dextrose iron hydroxide injection for the treatment of renal anemia in hemodialysis patients; proceedings of the 2013 Chinese Expert Consensus Workshop on the Treatment of Renal Anemia, Jinan, F, 2012 [C].

475 Yuan Ying. Study on the effects of Cordyceps sinensis polysaccharide extract to lower uric acid and colchicine on the yield of cordycepin [D]; South China Normal University, Microbiology and Biochemical Pharmacology, 2012.

476 Zhang HW. Effect of CVVH and CVVHDF on the prognosis of AKI patients with sepsis at the same flow rate of waste fluid [D]; Hebei Medical University, Emergency Medicine, 2012.

477 Zhang Jisheng, Yang Fengyan, Chen Qijun, et al. Effects of ascorbic acid dialysate on the expression of hs-CRP, TNF-α, IL-6, MDA, and GSH-px factors in patients with intravenous iron supplementation on maintenance hemodialysis [J]. Chinese Journal of Nephrology, 2012, 13(12): 1071-4.

478 Zhang Junli. Study of Ambroxol Hydrochloride Injection on Lung Surface Active Substance Protein-D in Rats with Acute Lung Injury Caused by Paraquat Poisoning [D]; Hebei Medical University, Emergency Medicine, 2012.

479 Zhang M. Clinical experience in emergency rescue of paraquat poisoning [J]. China Medical Guide, 2012, 10(11): 290-1.

480 Zhang Nanyun. Effects of thermal environment on monoamine neurotransmitters in the extracellular fluid of hypothalamus of rats recovering from exhaustive swimming [D]; Nanjing Institute of Physical Education, Exercise and Human Science, 2012.

481 ZHANG Nian-Yun, ZHANG Yun-Kun, YUN Kun Z. Effects of thermal environment on monoamine neurotransmitters in the extracellular fluid of hypothalamus of rats recovering from exhaustive swimming exercise; proceedings of the 2012 China Exercise Physiology and Biochemistry Academic Conference, Rizhao, Shandong, F, 2012 [C].

482 ZHANG Qing-De, QU Zhong-Sen, ZHONG-SEN Q U. Effects of peritoneal dialysis fluid-added urokinase on oxidative stress and endothelial function in patients with peritoneal dialysis [J]. Journal of Clinical Nephrology, 2012, 12(6): 252-5.

483 ZHANG Qing-De, QU Zhong-Sen, ZHONG-SENG Q U. Protective effect and mechanism of peritoneal dialysis fluid-added urokinase on patients with uremia complicated with cerebral infarction [J]. Chinese Journal of Cerebrovascular Disease (Electronic Edition), 2012, 6(5): 256-60.

484 Zhang Tianjie, Song Kingland, Jin Donghua, et al. Effect of citrate dialysis with application of blood conservation fluid (ACD-A fluid) on refractory hypertension in dialysis [J]. Chinese Journal of Hemorheology, 2012, 22(2): 250-2.

485 Zhang Xianggui, Zhang Yongjun, Xu Lijun, et al. Effects of mannitol on oxidative stress-related indexes in hypertensive patients on maintenance hemodialysis [J]. China Pharmacy, 2012, 23(16): 1477-9.

486 Zhang Xianggui, Zhang Yongjun, Yang Dan, et al. The effect of mannitol on blood pressure of hypertensive patients on maintenance hemodialysis and its mechanism [J]. Shandong Medicine, 2012, 52(40): 54-6.

487 Zhang Xuefeng, Ma Siqing, Wu Shizheng, et al. Establishment of intensive treatment protocol for acute severe plateau disease [J]. China Critical Care Emergency Medicine, 2012, 24(7): 415-8.

488 ZHANG YUNKUN, TANG FANG, FANG T. Effects of branched-chain amino acids on 5-hydroxytryptamine and its precursors in the extracellular fluid of hypothalamus of exercising rats; proceedings of the 2012 4th International Forum on Sports Nutrition Foods, Beijing, F, 2012 [C].

489 Zhang ZY, Li MX, Yu YW, et al. Long-term clinical efficacy of ambulatory peritoneal dialysis versus continuous ambulatory peritoneal dialysis [J]. China Blood Purification, 2012, 11(3): 131-5.

490 Zhao Haiyan. Cloning, Expression, Polyclonal Antibody Preparation, and Preliminary Study of Recombinant Human Thioredoxin 1 cDNA and its Protective Effect on Neonatal Rats with Endotoxemia [D]; Southern Medical University, Pediatrics, 2012.

491 Zhao Xiyun, Hao Li, Fang Mei, et al. The effect of nursing intervention on the increase of body mass during the interdialysis period in maintenance hemodialysis patients [J]. Anhui Medicine, 2012, 33(7): 910-1.

492 Zhao T. Preparation of long-acting human serum albumin and its biological function and pharmacokinetics [D]; Shandong University, Microbiology and Biochemical Pharmacy, 2012.

493 ZHAO Yu, ZHOU Xiaoyan, JIANG Yinhua, et al. Pulsed Tissue Doppler Evaluation of Right Ventricular Function and Load Status in Patients with Uremia [J]. Journal of Clinical Ultrasound Medicine, 2012, 14(5): 317-9.

494 Zhu Qing, Ji Wei, Zhao Xiyue, et al. Superficial cervical plexus nerve block combined with remifentanil intravenous pumping in uremic patients undergoing parathyroid surgery [J]. Journal of Medical Research, 2012, 41(2): 137-40.

495 Zeng Qiao, Li Qi, Zhong Qingrong, et al. Efficacy of iron sucrose combined with erythropoietin in the treatment of renal anemia on maintenance hemodialysis [J]. Hainan Medicine, 2013, 24(23): 3457-9.

496 Chen YL. Effects and mechanisms of adipose tissue RAS activation on adipose tissue macrophage phenotypic changes in rats with chronic renal failure [D]; Southern Medical University, Internal Medicine (Nephrology), 2013.

497 Chen Chunzhi. Clinical study of immunosorbent therapy for acute progressive glomerulonephritis [J]. Healthy People (Late Edition), 2013, (11): 106-.

498 CHENG Shiping, CHA Yan, YUAN Jing, et al. Effects of Renkang injection on peritoneal dialysis function and oxidative stress in rats [J]. Journal of Guiyang Medical College, 2013, 38(2): 138-40.

499 Cui Ling, Tang Min, Jiao Yali, et al. Clinical Observation of Prostaglandin in the Treatment of Pulmonary Arterial Hypertension in Maintenance Hemodialysis Patients [J]. Journal of Clinical Rational Use of Drugs, 2013, 6(20): 59-60.

500 Deng Yuanyan. Effects of blockade-reducing agents on exercise endurance in rats with acute hindlimb ischemia and their possible mechanisms of action [D]; Southern Medical University, Internal Medicine (Cardiovascular Disease), 2013.

501 Deng Yuanyan, Zhang Quan, Zhang Ye, et al. Effects of polyethylene oxide on the hemodynamics of abdominal aorta in rats with acute hindlimb ischemia; proceedings of the 15th National Congress of Cardiovascular Disease of the Chinese Medical Association, Tianjin, F, 2013 [C].

502 Dong Jianying, Li Lei, Shang Mingxia, et al. Survey on the current status of neonatal nurse specialists in military hospitals [J]. Chinese Journal of Modern Nursing, 2013, 19(21): 2506-9.

503 Fu Y, Xiao Xiangyun, Tang Hong, et al. Analysis of the efficacy of different blood purification modalities for chronic renal failure [J]. International Journal of Urology, 2013, 33(6): 758-60.

504 Fujiyama. Pharmacokinetic studies of quinzetol in pigs, chickens, fish, rats and humans [D]; Huazhong Agricultural University, Basic Veterinary Medicine, 2013.

505 Ge XY. Extraction and functional activity of polysaccharides from Cordyceps chrysanthemi fruiting bodies [D]; Fujian Agriculture and Forestry University, Food Science and Engineering; Food Science, 2013.

506 GONG Rong, PI Jingjing, QUAN Dayong, et al. Application study of daytime small-dose incremental peritoneal dialysis in patients with end-stage renal disease [J]. Chinese Journal of Clinical Physicians (Electronic Edition), 2013, (24): 11185-8.

507 Guo Yisha. Preparation of Capsaicin Nanostructured Lipid Carriers and Investigation of Their Characterization [D]; Hebei Medical University, Pharmacy, 2013.

508 Huang Guoquan, Chen Rui Ju, Li Zhanpeng, et al. Continuous static-venous hemodialysis filtration in the treatment of multiple organ dysfunction syndrome [J]. Chinese Medicine, 2013, 8(1): 112-4.

509 Huang Lin, Li Yanlin, Pang Jie, et al. Effects of urethane Kang Combination on peritoneal dialysis fluid-induced histologic changes and TGF-β1 expression in rat peritoneum [J]. Chinese Medicine Clinical Research, 2013, (11): 10-2.

510 Huang Yanlin, Li Jianying, Teng Yanjuan, et al. Effect of exercise therapy on dialysis adequacy in uremic hemodialysis patients [J]. Journal of Nurse Advancement, 2013, 28(7): 587-8.

511 Huang Ying. Biochemical and Immunological Characterization of Hemagglutinin in Snake Pitvipers [D]; Southern Medical University, Pharmacology, 2013.

512 ZHANG Xiuqin. Evaluation of longitudinal strain and torsional motion of left ventricular myocardium in uremic patients with different PTH levels by two-dimensional speckle tracking technique [D]; Wenzhou Medical University; Wenzhou Medical College, Imaging Medicine and Nuclear Medicine, 2013.

513 Kong Juan. Clinical efficacy of nalmefene, naloxone and hemodialysis in the treatment of acute severe alcoholism [D]; Zhengzhou University, Emergency Medicine, 2013.

514 Li Jianying, Huang Yanlin, Teng Yanjuan, et al. Effects of exercise training on fatigue in uremic hemodialysis patients [J]. Journal of Guangxi Medical University, 2013, 30(6): 981-2.

515 Li Jianying, Huang Yanlin, Teng Yanjuan, et al. Effects of exercise training on lower limb motor function in uremic hemodialysis patients [J]. Chinese Journal of Modern Nursing, 2013, 19(15): 1749-50.

516 Li Rong. Mechanism of platelet-mediated tumor vascularization and stability [D]; Southwest Medical University; Luzhou Medical College, Pharmacology, 2013.

517 Li WK. Effects of Andrographis paniculata on neurobehavior, hippocampal intersynaptic Glu levels and its receptor NMDA levels in rats [D]; China Medical University, Public Health and Preventive Medicine; Health Toxicology, 2013.

518 Li W. Cardioprotective effects of scopolamine combined with statin in direct PCI after AMI [D]; Hebei Medical University, Internal Medicine, 2013.

519 Li Chui, Zhang Mou, Zhao Yu, et al. Observations on the effects of hyperbaric oxygen on the blood flow of arteriovenous endovascular fistula for dialysis [J]. Sichuan Animal, 2013, 32(1): 112-5.

520 LIAN JUN, LI JING JING, JING JING L. Clinical application value of blood-water pretreatment dialyzer after self hemodialysis for preventing dialyzer reaction in maintenance hemodialysis patients [J]. China Modern Drug Application, 2013, 7(20): 79-80.

521 Lin Shan Pong. Prolyl 4 hydroxylase inhibition for the treatment of CKD anemia-a new means of a very promising CKD anemia drug; proceedings of the 2013 Annual Meeting of the Chinese Medical Association Nephrology Section, Fuzhou, F, 2013 [C].

522 Liu Chunqiu, Liu Lei, Li Liangzhi, et al. Study on the effect of osteotriol on microinflammation and oxidative stress in maintenance hemodialysis patients [J]. Chinese Family Medicine, 2013, 16(5): 516-9.

523 Liu DD. Experimental study on the treatment of chronic osteomyelitis with targeted ozone infusion [D]; Second Military Medical University, Surgery (Bone Surgery), 2013.

524 Liu G. Experimental study of transferrin-modified adriamycin-carrying graphene oxide nanoparticles against glioma [D]; Suzhou University, Neurosurgery, 2013.

525 Liu WJ. Salt loading test combined with hydration improvement program to prevent contrast nephropathy nursing study [D]; Xuzhou Medical University; Xuzhou Medical College, Nursing, 2013.

526 Liu W. Effects of immunized OBR extracellular proximal transmembrane region proteins on glucose and lipid metabolism in SD rats [D]; Southern Medical University, Zoology, 2013.

527 Ma Li, Lin Zhilong, Luo Hongmin, et al. Protective effect of pyruvate peritoneal dialysis solution on peritoneal organs after intravenous fluid resuscitation in hemorrhagic shock in rats [J]. Infection, Inflammation, Repair, 2013, 14(2): 84-7.

528 Pan M. Nursing analysis of high arteriovenous endovascular fistula in 30 cases of long-term hemodialysis patients [J]. Chinese Ethnic and Folk Medicine, 2013, 22(19): 83.

529 PENG Hongwei. Clinical observation on 22 cases of severe organophosphorus poisoning rescued by hemodialysis [J]. Medical Aesthetics and Cosmetology (Zhongdian Journal), 2013, (12): 181-,2.

530 PENG Xionghui, WU Qiufen, YAN Jiexing, et al. Effects of pre-exercise training on the changes of striatal monoamine neurotransmitters and their metabolites in rats with focal ischemia [J]. Journal of Physical Education and Sport, 2013, (6): 132-7.

531 Qiu Ju. Discussion on the application of health education on the effect of improving the home care ability of caregivers of elderly hemodialysis patients [J]. Chinese and Foreign Medicine, 2013, 32(9): 137,9.

532 QIU Shoutao, ZHANG Yunkun, YUN-KUN Z. Changes of Glu and GABA contents in striatal microdialysis fluid of rats during the recovery period of re-exhaustion exercise [J]. Sports Research, 2013, 34(3): 41-5.

533 Qu Songben, Liu Xinhua, Wang Furong, et al. Effect of urethane particles on oxidative stress in maintenance hemodialysis patients [J]. World Journal of Integrative Medicine, 2013, 8(12): 1232-4.

534 Ren Yi. Study on the protective effect of estrogen on acute lung injury caused by paraquat poisoning [D]; Nanjing University, Clinical Medicine, 2013.

535 Ren Yueheng. The therapeutic effect of stable hypoxia-inducible factor on anemia in chronic kidney disease and its regulation of target gene mechanism [D]; Fudan University, Clinical Medicine, 2013.

536 RONG Xiangjiang, ZHOU Jun, ZHANG Yuqin, et al. Effects of preexercise on prefrontal cortex ascorbic acid level and recovery of learning memory ability in cerebral ischemic rats [J]. Chinese Journal of Physical Medicine and Rehabilitation, 2013, 35(7): 519-22.

537 Shang WJ. Renal injury and protection in brain-dead rats and clinical practice of organ donation from brain-cardiac double-dead donors [D]; Zhengzhou University, Surgery (Urology), 2013.

538 She Liping, Yang Jinfang, Dong Shuya, et al. The effect of self-management education on quality of survival in maintenance hemodialysis patients [J]. Jilin Medical Science, 2013, 34(23): 4802-3.

539 Sun N. Effects and mechanisms of late protein oxidation products on proliferation and osteogenic differentiation of rat bone marrow mesenchymal stem cells and osteoblasts [D]; Southern Medical University, Clinical Medicine, 2013.

540 Sun Yang. Changes of MUC1 mucin during colitis carcinomatosis and the effect of apple polysaccharides on MUC1 [D]; Shaanxi University of Traditional Chinese Medicine; Shaanxi College of Traditional Chinese Medicine, Traditional Chinese Medicine, 2013.

541 Sun Youwen. Experimental study on the effect of silencing Survivin gene by magnetic siRNA-CCMN on the proliferation and apoptosis of individual bladder cancer cells [D]; Shandong University, Clinical Medicine (Surgery-Urology), 2013.

542 Sun ZK. Meta-analysis of a randomized controlled trial of intravenous N-acetylcysteine for the prevention of contrast nephropathy [D]; Southern Medical University, Cardiovascular Medicine, 2013.

543 Tu, Xinrui. Risk factors for Enterococcus spp. infection and drug resistance in hospitalized patients [D]; China Medical University, Clinical Medicine; Infectious Diseases, 2013.

544 WANG Chengkai, SONG Changpeng, CAI Xioman, et al. Effects of simvastatin tablets on oxidative stress status and efficacy in dialysis-combined stroke patients [J]. Chinese Journal of Emergency Resuscitation and Disaster Medicine, 2013, 8(6): 535-7.

545 Wang D. Observation on the efficacy of lamotrigine in the treatment of trigeminal neuralgia [D]; Southern Medical University, Stomatology, 2013.

546 Wang H. Observations on the effects of nitrendipine on blood uric acid, nitric oxide and blood pressure in hypertensive patients on hemodialysis [J]. China Medical Guide, 2013, (17): 150-.

547 Wang Q. Effects of different blood purification methods on serum SOD and MDA in patients with paraquat poisoning [D]; Hebei Medical University, Emergency Medicine, 2013.

548 Wang Xiaolei. Preparation and preliminary pharmacokinetic study of artemether lipid nanoparticles [D]; Shanxi Medical University, Pharmacy, 2013.

549 Wei Xing, Zhang Zejin, Liu Luojin, et al. Effects of compound danshen tablets on oxidative stress and microinflammatory status in patients with diabetic nephropathy [J]. Medical Information (Lower Decade), 2013, 26(10): 99-100.

550 Wu K. Observation on the efficacy of hemodialysis perfusion combined with hyperbaric oxygen in treating patients with severe hypnotic sedative drug intoxication [J]. Contemporary Nurses (Specialty Edition), 2013, (6): 112-3.

551 WU XIAOYING, WU CHANGXIU, ZHAO YULAN, et al. Observation on the efficacy and nursing care of hemoperfusion combined with hemodialysis in the treatment of patients with acute poisonous rat strength poisoning [J]. International Journal of Nursing, 2013, 32(4): 680-2.

552 Xiao Baili, Sui Xiaoni, Zhang Yunjing, et al. Effects of different dialysis membranes on bone mineral metabolism and microinflammatory status in maintenance hemodialysis patients [J]. China Blood Purification, 2013, 12(7): 379-83.

553 Xie A. Pharmacokinetics of cycloserine in the lung and brain of healthy rats by microdialysis [D]; Suzhou University, Internal Medicine (Respiratory Diseases), 2013.

554 Xu, Suiyi. Protective effects of intermittent multiple hypothermia on glucose-oxygen deprived neurons [D]; Southern Medical University, Neurology, 2013.

555 Yan WJ. Screening and identification of Streptococcus pneumoniae ClpE interacting proteins [D]; Chongqing Medical University, Clinical Laboratory Diagnostics, 2013.

556 Yang, Wei-Ning. Effect of health education on improving home care ability of hemodialysis patients and caregivers [J]. Chinese and Foreign Health Digest, 2013, (33): 180-,1.

557 YAO QIAO QIAOLING, QIU SHULI, SHU-LI Q I U. Clinical nursing pathway applied to peritoneal dialysis treatment of chronic renal failure patients [J]. International Journal of Medicine and Health, 2013, 19(20): 3222-5.

558 Zhang Honghao, Lin Chunhuan, Wang Hongjie, et al. Clinical efficacy of methylcobalamin capsule combined with hemoperfusion in the treatment of uremic peripheral neuropathy [J]. Chinese Journal of Continuing Medical Education, 2013, 36(1): 49-50.

559 Jingjing Zhang, Yanlin Wang, Zhaojun Qin, et al. Effects of intraperitoneal resuscitation with pyruvate on intestinal injury in rats with hemorrhagic shock [J]. Chinese Journal of Anesthesiology, 2013, 33(11): 1393-6.

560 ZHANG Nian-Yun, ZHANG Yun-Kun, YUN-KUN Z. Effects of thermal environment on monoamine neurotransmitters in the extracellular fluid of hypothalamus of rats during the recovery period of exhaustive swimming exercise [J]. Sports Research, 2013, 34(3): 50-4.

561 ZHANG Qing-De, QU Zhong-Sen, ZHONG-SEN Q U. Study on the therapeutic mechanism of peritoneal dialysis fluid-added urokinase in patients with uremia combined with acute cerebral infarction [J]. Journal of Brain and Neurological Diseases, 2013, 21(1): 10-3.

562 Zhang X. Preparation of lipid microbubble-dual ligand paclitaxel nanoparticle complexes and evaluation of their in vitro biological properties [D]; Southern Medical University, Imaging Medicine and Nuclear Medicine (Diagnostic Ultrasound), 2013.

563 Zhang X. Construction of a localized drug delivery system in the inner ear using PLGA nanoparticles as carriers and study of drug delivery mechanism [D]; Guangdong Pharmaceutical University, Pharmacy, 2013.

564 Zhang Xufang, Cheng Shubi, Han Xiaowei, et al. Home aerobic exercise in hemodialysis patients [J]. General Practice Nursing, 2013, 11(10): 903-5.

565 Zhang Yanfei. Clinical analysis of continuous blood purification for the treatment of diabetic nephropathy ketoacidosis combined with acute renal failure [J]. China Medical Guide, 2013, (29): 409-,10.

566 Zhong Honglin, Wang Bing, Ou Weining, et al. Application of continuous blood purification therapy in infectious shock patients with renal dysfunction [J]. Guangxi Medicine, 2013, (10): 1348-50.

567 Zhou Lili, Ji Tianrong, Liu Feng, et al. The effect of nursing interventions carried out by applying the Focused Solution Model on the self-management ability of maintenance hemodialysis patients [J]. Chinese Journal of Modern Nursing, 2013, 48(34): 4201-4.

568 Chen J. Mechanism of Klotho gene hypermethylation in indolephenol sulfate-induced vascular calcification [D]; Fudan University, Internal Medicine, 2014.

569 Chen Yu. Analysis of diagnosis and treatment of severe acute pancreatitis complicated with multiple organ dysfunction syndrome [J]. Heilongjiang Medicine, 2014, 38(3): 263-5.

570 Chen Zejun, Gong Rong, Pi Jingjing, et al. A preliminary study on the practice effect of joint management of homebound peritoneal dialysis patients between tertiary hospitals and community hospitals [J]. West China Medicine, 2014, 29(5): 831-4.

571 Chen LQ, Chen T, TAO C. Experiences of nursing interventions for patients on maintenance dialysis; proceedings of the 2014 Academic Annual Meeting of the Nephrology Branch of the Chinese Medical Association, Xi'an, F, 2014 [C].

572 Ding W L, Wang J X, Yan S M, et al. Clinical observation of colon dialysis with Chinese medicine retention enema in the treatment of middle and advanced chronic renal failure [J]. Modern Journal of Integrative Medicine, 2014, (17): 1867-8.

573 Feng T. Role of CCK system in conditioned fear memory and its mechanism in rats with post-traumatic stress disorder [D]; Hebei Medical University, Pathology and Pathophysiology, 2014.

574 Gao Jinzhi. Effect of collaborative care model on self-care ability and quality of life of hemodialysis patients [J]. Qilu Nursing Journal, 2014, (1): 33-5.

575 Hou Fanfan. Uremic toxins associated with cardiovascular complications; proceedings of the Chinese Medical Association Nephrology Section 2014 Blood Purification Forum, Wuhan, F, 2014 [C].

576 HU Gaoxiao, PENG Yizhi, WANG Fan, et al. Role of blood purification in burn sepsis patients [J]. Chinese Journal of Burns, 2014, 30(3): 213-8.

577 HU Leibin, WENG Yaping, LIU Chunbo, et al. Study on the effect of aerobic exercise on calcium and phosphorus metabolism levels in hemodialysis patients [J]. Modern Practical Medicine, 2014, 26(12): 1545-6.

578 Huang, H., Han, X. T., Sha, Q., et al. Nursing interventions in uremic patients on dialysis; proceedings of the Chinese Medical Association Nephrology Branch 2014 Academic Annual Meeting, Xi'an, F, 2014 [C].

579 Huang L, Gao Sterilization, Liang M L, et al. Prostaglandin injection after arteriovenous endovascular fistuloplasty in uremic patients [J]. Journal of Clinical Nephrology, 2014, 14(7): 429-32.

580 Huang M. Preparation of Enterobacter cloacae Z0206 polysaccharide and its role in alleviating lipid metabolism disorders in high-fat-fed mice and its immunological mechanism [D]; Zhejiang University, Animal Nutrition and Feed Science, 2014.

581 Huang Yanlin, Li Jianying, Teng Yanjuan, et al. Effect of exercise training on sleep quality of uremic hemodialysis patients [J]. Chinese Journal of Modern Nursing, 2014, 20(15): 1753-4,5.

582 Jiang, Meiyun. Impact of systematic health promotion on dialysis quality [J]. Medical Information, 2014, (19): 358-.

583 Li Caihong, Feng Xiangfeng, Xue Song, et al. Observation on the efficacy of recombinant human erythropoietin combined with iron preparation in the treatment of patients on maintenance hemodialysis [J]. Journal of Difficult Diseases, 2014, (8): 810-3.

584 Liang Yanping. Effect of targeted nursing intervention on blood pressure in MHD patients [J]. Frontiers of Medicine, 2014, (9): 338-9.

585 LIU F, LIU XIHAN, YIN WEI, et al. Effect of α-lipoic acid on oxidized protein products in patients with end-stage renal disease in advanced stages [J]. Medical Clinical Research, 2014, (2): 349-51.

586 Liu L. Protective effect of quercetin on atherosclerosis and its mechanism [D]; Huazhong University of Science and Technology, Nutrition and Food Hygiene, 2014.

587 Liu Mei. Observation on the effect of comprehensive nursing care applied in early dialysis for diabetic nephropathy [J]. Journal of Clinical Rational Drug Use, 2014, 7(4): 83-4.

588 Liu Xinhua, Wu Qiong, Wang Furong, et al. Effect of urethane particles on HO-1 levels in maintenance hemodialysis patients [J]. World Journal of Integrative Medicine, 2014, 9(12): 1321-3.

589 Liu Xueyi, Chai Hong, Du Zancai, et al. Effects of tea polyphenols on oxidative stress and microinflammatory status in maintenance hemodialysis patients [J]. Chinese Journal of Biochemical Drugs, 2014, (7): 87-9.

590 Liu ZG, Ren LY, Ren Shan'er, et al. Study on the treatment of renal bone disease with low-calcium dialysis, sevelamer hydrochloride, and osteotriol shock therapy [J]. China Practical Medicine, 2014, (25): 30-1.

591 Lu Z. Establishment and observation of an animal model of central venous catheter for hemodialysis [D]; Tianjin Medical University, Clinical Medicine; Surgery, 2014.

592 Lu Q. H., Ding G. H., Shi M., et al. Effects of leucovorin on oxidative stress status in hemodialysis patients [J]. Practical Clinical Medicine, 2014, (2): 32-4.

593 Lu Enfeng, He Jianjing, Li Song, et al. Effects of high flux dialysis on oxidative stress and microinflammatory status in maintenance hemodialysis patients [J]. Journal of Guangxi Medical University, 2014, 31(4): 636-8.

594 L. Lu. Clinical experience of applying combined artificial kidney combined with hemopexin in the treatment of myocarditis after paraquat poisoning [J]. Medicine and Health Care, 2014, 22(1): 24.

595 Deer, W. T.. Improvement effect of taurine on aluminum-induced learning and memory impairment in rats and the corresponding mechanism [D]; Shandong University, Health Laboratory Science, 2014.

596 Lu Yao. Evaluation of the application effect of exercise therapy in maintenance hemodialysis patients [J]. Nursing Practice and Research, 2014, (5): 43-4,5.

597 Luo Yiqun. Analyzing the clinical effect of early application of blood purification in the treatment of acute poisoning [J]. Health Road, 2014, (7): 121-.

598 Mai Miaojin, Pang Xiaoyu, Chen Zhentuo, et al. Effect of nursing intervention on quality of life and satisfaction of hemodialysis patients with renal bone disease [J]. China Disability Medicine, 2014, (17): 165-7.

599 Mu R. Observation on the efficacy of intravenous iron supplementation and oral iron supplementation in the treatment of renal anemia in patients on maintenance hemodialysis [J]. China Pharmacoeconomics, 2014, (1): 227-8.

600 Nie Zhenyu. Effect of rosiglitazone on AQP-1, VEGF-A and COX-2 in uremic peritoneal dialysis rats [D]; Ningbo University, Internal Medicine, 2014.

601 Qiu Jing, Zhang Qingxia, Wang Liying, et al. Observations on the effect of different nursing methods on catheter infection in 90 peritoneal dialysis patients [J]. Yanbian Medicine, 2014, (19): 52-4.

602 Ren Y. Probucol in peritoneal fibrosis and its relationship with SRF [D]; Guiyang Medical College, Nephrology, 2014.

603 SHI JUN H, WANG DONG H, DONG HONG W. Effects of different blood purification methods on serum antioxidant low-density lipoprotein antibodies in patients with end-stage renal disease [J]. Chinese Journal of Gerontology, 2014, (8): 2107-8.

604 SI L L, LI L, LI L. Observation on the efficacy of applying glycerol fructose to prevent dialysis imbalance syndrome [J]. Chinese Journal of Physician Advancement, 2014, 37(1): 58-9.

605 Sun Shuqing, Chen Jin, Wang Yan, et al. Double venous puncture as temporary vascular access in hemodialysis [J]. Chinese Journal of Physician Advancement, 2014, 37(7): 57-9.

606 Wang Chunhua, Gao Leiping, Zhou Yufeng, et al. Observation on the efficacy of oral phosphorus binding agent in dialysis patients [J]. Zhongguo Nguo Kang Medicine, 2014, (21): 11-3.

607 Wang D. F. Observation on the clinical effect of blood purification-assisted treatment of acute poisoning [J]. Journal of Clinical Rational Use of Drugs, 2014, (34): 165-.

608 Wang Jianzhi. Chinese medicine nursing experience of uremia [J]. China Modern Distance Education of Traditional Chinese Medicine, 2014, 12(24): 137-8,46.

609 Wang JZ. Rehabilitation exercise improves physical fitness and quality of life in C KD patients [J]. Chinese Electronic Journal of Nephrology, 2014, (5): 279-.

610 Wang Juan, Huang Yanlin, Wu Zhuomei, et al. Effects of exercise training on anxiety and depression in uremic hemodialysis patients [J]. Guangxi Medicine, 2014, (9): 1225-7.

611 WANG Weijun, LI Qian, QIAN L. Study on the effect of Renkang injection on oxidative stress and microinflammatory state in maintenance renal dialysis patients [J]. Chinese Medicine Herald, 2014, (11): 61-2,3.

612 WANG Xinmei, ZHU Xiaoling, XIAO LING Z. Effects of aerobic exercise on fatigue status of maintenance hemodialysis patients; proceedings of the 2014 Annual Academic Conference of the Nephrology Branch of the Chinese Medical Association, Xi'an, F, 2014 [C].

613 Wang Yan. Oxygen intervention on oxidative stress and energy metabolism in whole brain ischemia/reperfusion in long-pawed gerbils [D]; China Medical University, Clinical Medicine; Emergency Medicine, 2014.

614 WANG Yueli, JIN Juanjuan, WANG Chengkai, et al. Differential effects of intravenous iron supplementation combined with simvastatin versus oral iron supplementation alone on clinical efficacy and oxidative stress status in patients with renal anemia [J]. Chinese Journal of Emergency Resuscitation and Disaster Medicine, 2014, (7): 609-12,23.

615 Wang Zhe, Wei Fang, Chen Haiyan, et al. Analysis of prognostic factors in patients with acute paraquat poisoning [J]. Journal of Tianjin Medical University, 2014, 20(5): 392-5.

616 Wu, T. Tim. Effect of hippocampal 5-HT pathway on POFS and intervention study of leucine [D]; Wenzhou Medical University, Gastrointestinal Surgery, 2014.

617 Wu, Xiaohui. Study related to cerebral glucose metabolism and neurospecific markers after craniocerebral injury [D]; Chongqing Medical University, Neurosurgery, 2014.

618 WU Yongyao, XIA Min, CAO Shengsheng, et al. Observations on the effects and safety of individualized exercise therapy on cardiac function and exercise capacity of uremic patients during hemodialysis treatment [J]. China Blood Purification, 2014, 13(8): 580-4.

619 Xiao Yang, Guan Wei, Cong Yunfeng, et al. Clinical efficacy of continuous blood purification for acute respiratory distress syndrome with hypoxemia [J]. China School Medicine, 2014, 28(12): 955-7.

620 Xie Xiangli, Ma Liqing, Ye Shuqin, et al. The correlation between exercise training and health promotion behavior in hemodialysis patients; proceedings of the 2nd Shanghai International Nursing Conference, Shanghai, F, 2014 [C].

621 Xu H. Effect of health education on quality of life and compliance behavior of hemodialysis patients [J]. China Health Education, 2014, 30(10): 949-50.

622 Xu JY. Anticipatory care of elderly patients with severe renal failure implementing bedside intermittent hemodialysis [J]. Chinese Journal of Modern Nursing, 2014, 20(16): 1931-3.

623 Xu Yikuang, Yang Zhiqiang, Gong Weiying, et al. The effect of comprehensive rehabilitation on the efficacy of maintenance hemodialysis patients [J]. Massage and Rehabilitation Medicine, 2014, (6): 91-,2.

624 Xue Xuan, Chang Xiaodong, Yang Youjing, et al. Effects of Irbesartan on Oxidative Stress and Microinflammatory State in Maintenance Hemodialysis Patients [J]. Western Medicine, 2014, 26(11): 1453-6.

625 YAN Haihong, CHENG Yongjun, WANG Wenlong, et al. Effects of metoclopramide added to peritoneal dialysis fluid on appetite and nutritional status of peritoneal dialysis patients [J]. Chinese Journal of Integrative Nephrology, 2014, (12): 1069-71.

626 YANG Wei-Wei, ZHANG Xi-Wen, ZHANG Liqin, et al. Evaluation of health education effect of clinical pathway applied to hemodialysis patients [J]. China Health Education, 2014, 30(11): 1005-7.

627 YI Ye, LU Yuanhang, JI Qianqian, et al. Effects of artificial cordyceps preparation on oxidative stress and microinflammation in elderly maintenance hemodialysis patients [J]. Journal of Clinical Nephrology, 2014, 14(9): 543-6.

628 YU Chaoxia, MA Yan, MA Y. Effects of ustekin treatment on intestinal mucosa and intra-abdominal pressure in patients with severe pancreatitis [J]. World Digest of Recent Medical Information (Continuous Electronic Journal), 2014, (25): 86-7.

629 Yu Xiuzhi, Lu Shi, Zhang Jinyuan, et al. Observation on the efficacy of methylcobalamin in the treatment of uremic peripheral neuropathy [J]. Chinese Journal of Integrative Nephrology, 2014, (12): 1056-8.

630 Yu Yaoliang, Xie Hao, Tong Fengqin, et al. Observation on the effect of sodium tanshinone IIA sulfonate on oxidative stress state in peritoneal dialysis rats [J]. Asia-Pacific Traditional Medicine, 2014, 10(4): 16-8.

631 Zhang, Chao-Nan. Effects of electroacupuncture on renal function and renal tissue β-strand protein expression in rats with chronic renal failure [D]; Chongqing Medical University, Acupuncture and Tuina, 2014.

632 Zhang Zhengyu. Protective effect of fasudil hydrochloride on vascular endothelial function in maintenance hemodialysis patients [J]. Journal of Practical Medicine, 2014, (21): 3499-502.

633 Zhang Fengli. Effect of comprehensive nursing care on health knowledge and related symptoms in patients with diabetic nephropathy [J]. International Journal of Nursing, 2014, (9): 2437-9.

634 Zhang Hongmei, Fan Ruyan, Chang Liyang, et al. Effects of tai chi exercise on physical fitness and fatigue status of hemodialysis patients; proceedings of the 2014 Annual Academic Conference of the Renal Disease Specialized Committee of the Chinese Society of Integrative Medicine, Shenzhen, F, 2014 [C].

635 Zhang, J. Z.. Overview of new drugs approved by FDA in January 2014 [J]. Shanghai Medicine, 2014, (5): 61-.

636 Zhang Q. Facilitation of the neuroprotective effect of ice tablet on the passage of kaempferol across the blood-brain barrier based on simultaneous blood and brain microdialysis [D]; Anhui Medical University, Traditional Chinese Medicine, 2014.

637 Zhang Shu-Qing, Liu Lan-Xia, Li Gui-Lan, et al. A study on the influence of peer education in self-health management of maintenance hemodialysis patients [J]. China Medical Innovation, 2014, (14): 97-9.

638 Zhang, W., Yu, W. J., Wang, D. H., et al. The effects of Jianshang and Stopping the Movement Tang on the anti-twitching effect and the excitatory amino acid transmitters in the brain of rats in the TS model; proceedings of the Fifth National Academic Forum for Doctoral Students of Traditional Chinese Medicine, Changsha, F, 2014 [C].

639 Zhang W, Yu WJ, Wang DH, et al. Effects of fortifying spleen and stopping movement soup on anti-twitching effect and excitatory amino acid transmitters in the brain of TS model rats [J]. Chinese Journal of Traditional Chinese Medicine, 2014, 29(5): 1653-6.

640 Zhang, X. Y., Lu, W., Feng, X., et al. Impact of health education on the quality of survival of hemodialysis patients [J]. China Pharmacoeconomics, 2014, (9): 113-5.

641 Zhang Yimiao, Tian Aihui, Zuo Li, et al. Study on the safety and effectiveness of using acidic oxidation potential water to disinfect hemodialysis concentrate containers [J]. China Blood Purification, 2014, 13(3): 152-5.

642 Zhu Quan. Serum oxidized protein product levels in uremic patients and clearance effects of different dialysis modalities [J]. Journal of Clinical and Experimental Medicine, 2014, (17): 1439-41.

643 SAKATA Y, ITO O, KOHZUKI M. Basic research on renal rehabilitation; proceedings of the 9th Beijing International Rehabilitation Forum, Beijing, F, 2014 [C].

644 Ben H. L., Song L. L., Ren T., et al. Application of behavioral staged change theory in health education for elderly peritoneal dialysis patients with diabetic nephropathy [J]. Journal of Nursing, 2015, (4): 62-5.

645 CAO Cong, MO Zhan Yu, ZHAN YU M. Observation on the efficacy of hemodialysis perfusion for uremic encephalopathy [J]. Modern Journal of Integrative Chinese and Western Medicine, 2015, (28): 3121-3.

646 Zeng Zeying. Analysis of the application value of comfort care model in hemodialysis patients with kidney disease [J]. Chinese and Foreign Medical Research, 2015, (5): 88-9.

647 Chen Jiebin. Comparative study on the effect of Chinese medicine in the treatment of chronic kidney disease stage 5 (non-dialysis) [D]; Southern Medical University, Integrative Medicine Clinic, 2015.

648 Chen Xianghua, Xie Zhuneng, Yang Pu, et al. Effects of methylcobalamin combined with hemodialysis filtration therapy on peripheral nerve conduction velocity and microinflammation level in patients with end-stage renal disease [J]. Guangxi Medicine, 2015, (5): 698-700.

649 Chen Zanqing. Clinical efficacy of early short-term continuous static-venous hemodialysis filtration for severe acute pancreatitis combined with acute lung injury [J]. Journal of Clinical Rational Drug Use, 2015, (36): 93-4.

650 CHENG Yongjun, SHOU Zhangfei, WANG Wenlong, et al. Effects of lipoic acid on oxidative stress, inflammation and nutritional status of peritoneal dialysis patients [J]. Chinese Journal of Immunology, 2015, (4): 541-4.

651 Dai L. Effect of exercise therapy on sleep quality of maintenance hemodialysis patients [J]. Medical Theory and Practice, 2015, 28(23): 3288-90.

652 SAN Jianping, JI Gang, GANG J I. Effects of different blood purification methods on vasoactive substances in uremic patients with chronic renal failure [J]. Chinese Journal of Practical Diagnosis and Therapy, 2015, 29(5): 481-3.

653 Deng Zhe, Zeng Hongke, Liang Shi, et al. Dynamic effects of assisted abdominal resuscitation on early intestinal injury in hemorrhagic shock in rats [J]. Chinese Emergency Medicine for Critical Illness, 2015, 27(1): 59-63.

654 Ding Xiang. Effects of home aerobic exercise on fatigue and sleep quality in maintenance hemodialysis patients [J]. Contemporary Nurses (Lower Decade), 2015, (3): 126-8.

655 Dong YX. A study of self-management education in physical activity of maintenance hemodialysis patients [D]; Zhengzhou University, Nursing, 2015.

656 Duan Hailing, Cui Xiangqin, Wang Zongbao, et al. Clinical application value of vitamin E combined with hemoperfusion in maintenance hemodialysis patients [J]. Clinical Metaphysics, 2015, (11): 1288-91.

657 Fan Lingling. Intervention effect of aerobic exercise on fatigue condition of maintenance hemodialysis patients [J]. Chinese and Foreign Medical Research, 2015, (15): 105-6.

658 Gao Feng, Li Fang, He Xiao, et al. Effects of leucovorin on nutrition and microinflammatory status of peritoneal dialysis patients [J]. Journal of Clinical Military Medicine, 2015, (5): 472-4.

659 Gao Hui. Study on the role and safety of transcutaneous electrical stimulation of the foot-sanli point in the resuscitation of patients with organophosphorus pesticide poisoning [D]; Hebei Medical University, Clinics of Integrative Medicine and Western Medicine, 2015.

660 Gao Lu, Ye Liping, Cheng Wei, et al. Observation on clinical efficacy of hemodialysis filtration in elderly uremic patients [J]. Journal of Clinical Nephrology, 2015, 15(2): 102-5.

661 Gao Xia. Clinical nursing pathway application in peritoneal dialysis for patients with chronic renal failure [J]. Chinese and Foreign Women's Health Research, 2015, (14): 138.

662 Guan Yuanyuan. Effect of EPO generating hormone combined with iron sucrose treatment on serological indexes and microinflammatory status of hemodialysis patients with renal anemia [J]. Journal of Hainan Medical College, 2015, 21(8): 1091-3.

663 Guo J. Etiologic analysis and prognostic risk factors of acute kidney injury [D]; Southern Medical University, Internal Medicine (Nephrology), 2015.

664 PASSING JARRU, WANG YAN-YU, YAN-YU W. The effects of different nursing models on the health knowledge rate and placement time of hemodialysis patients with tube placement [J]. Journal of Bengbu Medical College, 2015, (6): 830-2.

665 He Hangying, Jin Zhiping, Ma Shuanglian, et al. Exercise training improves physical and mental status of maintenance hemodialysis patients [J]. PLA Nursing Journal, 2015, (8): 1-4.

666 He Yun, Yin Yumin, Shi Wei, et al. Blood purification in severe acute pancreatitis combined with acute respiratory distress syndrome [J]. Frontiers of Medicine, 2015, (8): 28-9,30.

667 Hou J, Wen Xiang Q, Liu J, et al. Effects of leucovorin and iron sucrose on renal anemia and oxidative stress in elderly hemodialysis patients [J]. Chinese Journal of Gerontology, 2015, (5): 1184-6.

668 Hu Yi-wen. Hemodialysis combined with hemoperfusion in the treatment of calcium and phosphorus metabolism disorders in maintenance hemodialysis patients [D]; Southern Medical University, Internal Medicine (Nephrology), 2015.

669 Huang Liu, Huang Yanlin, Li Jianying, et al. Effects of aerobic exercise on lower limb motor function and fatigue in maintenance hemodialysis patients [J]. Guangxi Medicine, 2015, (4): 476-8,84.

670 Huang Qiangwei, Jin Ru, Wei Xiaoling, et al. Impact of WeChat Follow-up Education on Treatment Adherence of Hemodialysis Patients [J]. Hospital Management Forum, 2015, (4): 60-2.

671 Huang Xiang, Yu Shanshan, Chen Fang, et al. Observation on the efficacy of hemoperfusion combined with hemodialysis in treating patients with severe organophosphorus poisoning [J]. China Medical Guide, 2015, (26): 55-.

672 Jiao Yue, Liu Yang, Sun Dandan, et al. Interventional effects of Shouwu formula on levodopa-induced anisocoria in rats and its mechanism [J]. Chinese Journal of Biochemical Drugs, 2015, (2): 1-4,9.

673 LAI Fei. Clinical efficacy of laparoscopic gastric bypass for T2DM with different body mass index [D]; Southern Medical University, Surgery (Gastrointestinal), 2015.

674 Li J, Zhang Z, Wei SZ, et al. Effects of ultrapure dialysis on anemia and oxidative stress in maintenance hemodialysis patients; proceedings of the 2015 Clinical Acute and Critical Care Experience Exchange Summit, Beijing, F, 2015 [C].

675 Li Jialian, Quan Lixia, Wei Hong, et al. Effect of early nursing intervention on renal bone disease in young and middle-aged maintenance hemodialysis patients [J]. Medical Information, 2015, 28(51): 133.

676 Li Li-Yang. Application effect of GC extended care model in peritoneal dialysis patients [J]. Laboratory Medicine and Clinics, 2015, (19): 2946-8.

677 LI LING LI, ZHAO GUI YAN, GUI YAN Z. Observation on the effect of traditional Chinese medicine umbilical cord compress plus acupressure in treating constipation in hemodialysis patients [J]. China Rural Medicine, 2015, (7): 41-2.

678 Li Lu. Study on the disruptive effect of picloram on Escherichia coli biofilm formation in vitro [D]; Guangxi Medical University, Nephrology, 2015.

679 Li Suning. Preliminary study on the effect of carboxylated single-walled carbon nanotubes on liver, lung and kidney fibrosis in rats and its molecular mechanism [D]; Guangxi Medical University, Pharmacology, 2015.

680 Li XJ. Inhibition of gap junction protein 43 promotes neurological function recovery after cerebral ischemic injury in neonatal rats [D]; Suzhou University, Neurology, 2015.

681 Li Xiaojing. Study on the protective effect and mechanism of gap junction protein 43 analog (Gap26) on cerebral ischemia-hypoxia injury in neonatal rats [D]; Suzhou University, Neurology, 2015.

682 Li Yan. Experimental study on the effect of health education on improving the home care ability of caregivers of elderly hemodialysis patients [J]. Chinese and Foreign Women's Health Research, 2015, (21): 29-,36.

683 Chestnut, R. M.. Research on the application of artificial simulated enzymes in bioanalysis [D]; University of Chinese Academy of Sciences, Physical Chemistry, 2015.

684 Liu, Guanghui. Target metabolomics of sedative-hypnotic effects of Jiaotaiwan [D]; Guangzhou University of Traditional Chinese Medicine, Pharmacognosy, 2015.

685 Liu JX. Study on the efficacy of low-calcium peritoneal dialysis solution combined with low-protein diet and compound a-keto acid in treating hyperphosphatemia in patients with peritoneal dialysis [J]. International Journal of Urology, 2015, 35(5): 733-6.

686 Liu J, Liu XL, Qiao DC, et al. A study on the regulation of striatal A2AR and D2DR on the striatal-pallidal pathway during forceful exercise in rats; proceedings of the 2015 10th National Sports Science Congress, Hangzhou, F, 2015 [C].

687 Liu Lixiu. The effect of exercise on the quality of life of hemodialysis patients [J]. China Primary Medicine, 2015, (17): 2657-9,60.

688 LIU Shenghui, LU Yongxin, WANG Qingyue, et al. Effect of self-prescribed formula of benefiting qi and consolidating kidney and eliminating stasis on inflammatory response in patients with maintenance dialysis with malnutrition-inflammatory response syndrome [J]. Journal of Clinical Rational Drug Use, 2015, (25): 66-7.

689 LIU Shenghui, LU Yongxin, WANG Qingyue, et al. Effect of self-prescribed formula for strengthening qi and resolving blood stasis on cardiac function in patients on maintenance dialysis with malnutrition-inflammatory response syndrome [J]. Journal of Clinical Rational Drug Use, 2015, (22): 65-6.

690 LIU Xiaoqin, ZHANG Jie, ZHANG Xiaoning, et al. The effect of total seamless care model on compliance behavior adherence and comfort of maintenance hemodialysis patients [J]. Chinese Journal of Modern Nursing, 2015, 21(5): 555-7,8.

691 Lu Ailing, Lu Baoquan, Wang Jing, et al. Psychological care combined with exercise therapy in malnutrition of maintenance dialysis patients [J]. China Medical Innovation, 2015, (6): 72-4.

692 Lv Bo. The effect of colon dialysis with Tongzhi Lung Tang on blood lactate levels in patients with pneumonia combined with sepsis; proceedings of the 2015 Academic Annual Conference on Emergency Medicine and Academic Symposium on Acute and Critical Illnesses in Traditional Chinese Medicine, Zhengzhou, F, 2015 [C].

693 LUO JH, JIANG HUI, HUI J. The value of Sheng Vein Injection in elderly maintenance hemodialysis patients with hypotension and its effect on ET-I and NO expression [J]. Journal of Modern Integrative Chinese and Western Medicine, 2015, (15): 1671-3.

694 Luo X. W. Effects of spleen-enhancing and kidney-discharging methods on metabolic toxins and intestinal flora in chronic kidney disease stage 5 [D]; Guangzhou University of Traditional Chinese Medicine, Clinical Integrative Medicine, 2015.

695 Luo Y. Nursing experience of blood glucose management in hemodialysis patients with diabetic nephropathy [J]. China Practical Medicine, 2015, (26): 216-7.

696 Luo Meiliang. Effect of self-management education on self-management behavior of maintenance hemodialysis patients; proceedings of the Twenty-eighth Academic Exchange Meeting of the Nephrology Branch of the Chinese Society of Traditional Chinese Medicine, Guangzhou, F, 2015 [C].

697 Ma, Xiaofen. Brain changes in end-stage renal disease patients without significant cognitive impairment: a multimodal magnetic resonance imaging study [D]; Southern Medical University, Imaging Medicine and Nuclear Medicine, 2015.

698 Mok C.N., Liu Y.Y., Wang H.H., et al. Clinical study on the application of sequential dialysis combined with BiPAP treatment in end-stage renal disease complicating acute left heart failure? [J]. Chinese Journal of Integrative Nephrology, 2015, (2): 128-30.

699 Nie Qinqi. The effect of extended care on complications and self-care ability of hemodialysis patients [J]. Nursing Practice and Research, 2015, (1): 25-6.

700 Nie Zhenyu, Chen Qiangyue, Yu Xiongwei, et al. Effects of rosiglitazone on aquaporin 1, vascular endothelial growth factor A and cyclooxygenase 2 in uremic peritoneal dialysis rats [J]. Chinese Journal of Nephrology, 2015, 31(2): 126-32.

701 Nurzye Abrikimu, Ma Bing, Hu Lei, et al. Effects of different blood purification treatments on organ function and cytokines in dogs with multiple organ dysfunction [J]. Chinese Journal of Clinical Physicians (Electronic Edition), 2015, (21): 3925-9.

702 PENG Chunhui, WANG Qi, QI W. Maintenance of oxidative stress status in hemodialysis patients and therapeutic implications of nutritional support [J]. Jilin Med, 2015, (17): 3966-7.

703 Qiao Decai, Zhang Jimin, Hou Lijuan, et al. Study on the regulatory role of GABA and Glu in rat pallidum during force exercise [J]. Journal of Shenyang Sports Institute, 2015, 34(2): 82-6.

704 Qin Wenlan. Exploring the effect of hemodialysis on the level of inflammation and oxidative stress in uremic patients [J]. Everybody's Health (Middle Edition), 2015, (4): 45-.

705 Qin XD, Liu XS, Wu YF, et al. Efficacy of exercise therapy on non-dialysis patients with chronic kidney disease; proceedings of the twenty-eighth academic exchange meeting of the Chinese Society of Traditional Chinese Medicine Nephrology Branch, Guangzhou, F, 2015 [C].

706 Quan Xiuquan. Preparation of platinum dichloride-targeted nanomicelles based on PEGylated hyaluronic acid and its effect on a lung cancer model [D]; Yanbian University, Internal Medicine, 2015.

707 Rong Shuguang. Analyzing the value orientation of popular sports from the cultural consumption of sports shoes [J]. Sports and Science, 2015, 36(3): 79-86.

708 Shang Bangjuan. Changes of pulmonary artery pressure and its risk factors in patients with chronic kidney disease [D]; Taishan Medical College, Internal Medicine, 2015.

709 Shao N, Sun ZH, Liu LW, et al. Clinical efficacy of α-lipoic acid on peripheral neuropathy in maintenance hemodialysis patients [J]. International Journal of Transplantation and Blood Purification, 2015, 13(4): 20-2.

710 Sheng KX. Meta-analysis of the effect of exercise on hemodialysis patients during dialysis [D]; Zhejiang University, Nephrology, 2015.

711 Song Lulu, Ben Hongling, Li Yan, et al. Effects of collaborative care model on volume loading and self-care behavior of peritoneal dialysis patients with elderly diabetic nephropathy [J]. Nursing Research, 2015, (4): 469-70,71.

712 Sun Yun. Study on the association between leucovorin and renal anemia [D]; Shandong University, Clinical Medicine (Internal Medicine - Nephrology), 2015.

713 Wang J, Zhu QH, Chen J, et al. Isolation and purification of trypsin inhibitor from purple kidney bean and study of hypoglycemic effect [J]. Journal of Clinical Rational Drug Use, 2015, (9): 126-7.

714 Wang, M. H., Deng, Y., Jiang, L., et al. Dynamic observation of changes in glutamate concentration in the brain of perinatal hypoxic-convulsed rats and neuroprotective effect of lamotrigine [J]. Chinese Family Medicine, 2015, 13(7): 1042-4,162,seal 3.

715 WANG YAN, WANG QIANSHOU, QIANSHOU W. Effects of leucovorin and iron sucrose on renal anemia and oxidative stress in elderly hemodialysis patients [J]. China Continuing Medical Education, 2015, (29): 164-5.

716 WANG Yanqun, SONG Haifeng, YAO Gang, et al. Observation on the efficacy of levocanidin in improving cardiac function in patients with chronic renal failure and hemodialysis heart failure [J]. Modern Drugs and Clinics, 2015, (3): 90-3.

717 WANG Y, GAO Ganke, GAN Ke G. Effect of compound danshen injection combined with hemodialysis on the efficacy and oxidative indexes of patients with end-stage renal disease combined with heart failure [J]. Chinese Medicine Herald, 2015, 21(17): 78-80.

718 WANG Zhen, XU Zhe, ZHE X U. Analysis of factors associated with uremia complicating acute pancreatitis [J]. Journal of Clinical Nephrology, 2015, 15(6): 364-7.

719 Wang ZQ. Observation on the efficacy of blood transfusion during dialysis in patients with uremia [J]. Modern Diagnosis and Treatment, 2015, 26(18): 4221-2.

720 Wenpeng. Low-frequency electrical stimulation of the pontine nucleus of the foot affects gait and neurotransmitter levels in the ventral lateral nucleus of the thalamus in 6-OHDA Parkinson's rats [D]; Medical University of the South, Neurosurgery, 2015.

721 Wen Wen. Clinical efficacy of cefodizime combined with levofloxacin in the treatment of lower respiratory tract infections in hemodialysis patients with chronic renal failure and the effect on immune function [J]. Journal of Clinical Rational Drug Use, 2015, (24): 40-1.

722 Wu F. Clinical and cytologic study of ustekin and continuous veno-venous hemofiltration in the treatment of septic shock [D]; Southern Medical University, Critical Care Medicine, 2015.

723 WU Su-Ying, FANG Xiu-Yun, XIU YUN F. Discussion on the preventive effect of two methods on perianal and perineal eczema in medical ICU patients [J]. Contemporary Nurses (Lower Lunar), 2015, (11): 98-,9.

724 XI Hongge, QI Bailing, BAI Ling Q. Clinical comparison between high flux hemodialysis and conventional hemodialysis [J]. World Digest of Recent Medical Information (Continuous Electronic Journal), 2015, (31): 109-,10.

725 Xia Zaomin, Huang Yun, Yao Jingchun, et al. Effects of different blood purification modalities on quality of life of patients with renal failure [J]. Contemporary Medicine, 2015, (20): 100-,1.

726 Xiang Yingying, Wu Zhongming, Cai Xueqin, et al. A randomized controlled trial of increasing crisis awareness to promote glycemic attainment in patients with type 2 diabetes mellitus; proceedings of the 19th National Academic Conference of the Diabetes Branch of the Chinese Medical Association, Suzhou, F, 2015 [C].

727 Xiao Tailing, Qin Yan, Liu Cuiping, et al. Evaluation of self-management effects in elderly maintenance hemodialysis patients [J]. Chinese Medicine, 2015, 10(4): 598-600.

728 Xiao YQ. Clinical Observation on 30 Cases of Heart Failure Patients on Maintenance Dialysis Treated with Strong Xin Tang [J]. Everybody's Health (Middle Edition), 2015, (1): 127-.

729 Xiao Chang-Chang, Jiang Hui-Fang, Luo Jiou-Yuan, et al. Effects of different dosing regimens of iron sucrose injection on anemia and microinflammation in maintenance hemodialysis patients [J]. Journal of Gannan Medical College, 2015, 35(3): 427-9.

730 Xu Cuiling, Lu Dan, Ding Yanqiong, et al. Effect of medication combined with rehabilitation on osteoporosis in maintenance peritoneal dialysis patients [J]. Chinese Journal of Physical Medicine and Rehabilitation, 2015, 37(8): 621-3.

731 Xu Man, Wu Weiying, Zhou Qiongrong, et al. Effect of nursing intervention on the degree of skeletal muscle atrophy in hemodialysis patients [J]. Guangdong Medicine, 2015, (16): 2605-7.

732 XU WEN-YI, HUANG XUAN-ZHU, XUAN ZHU H. Study on the effect of different dressing change methods in the treatment of peritoneal dialysis catheter outlet infection [J]. Contemporary Medicine, 2015, (4): 87-8.

733 XUE Xiaoxia, XUE Dongxia, DONGXIA X U E. Effects of reduced glutathione on serum microinflammation, oxidative stress and renal function in dialysis patients with hyperbilirubinemia combined with renal failure [J]. China Medicine Herald, 2015, (26): 122-5.

734 Yang Liu. Effect of dexmedetomidine on propofol injection pain and its mechanism of action [D]; Guangdong Medical College, Anesthesiology, 2015.

735 YANG Pu, YANG Jingxin, XIE Zhuneng, et al. Efficacy of levocanidin combined with iron sucrose in the treatment of hemodialysis with renal anemia and its effect on the level of oxidative stress in patients [J]. Hainan Medicine, 2015, 26(24): 3680-2.

736 Ye Van, Yin Jinfeng, Yi Wei, et al. Clinical study on the effect of stroking on the improvement of "restless legs syndrome" in uremic hemodialysis patients [J]. Chinese Journal of Modern Medicine, 2015, 25(36): 97-101.

737 Yi Ye, Lu Yuanhang, Ji Qianqian, et al. Effect of Jinshuibao on oxidative stress and microinflammatory status in hemodialysis patients with diabetic nephropathy [J]. China Medicine Herald, 2015, (7): 93-6.

738 YI YE, LU YUAN HANG, YUAN HANG L. Effects of high-throughput dialysis on oxidative stress and microinflammation induced by intravenous iron supplementation in hemodialysis patients [J]. Guangdong Medicine, 2015, (4): 590-1.

739 Yin, Xiaotong. Preliminary study of IHHNV coat protein-vanabin shrimp gill cell membrane receptor [D]; Nanjing Agricultural University, Preventive Veterinary Medicine, 2015.

740 Yu Zengli. Observation on the efficacy of peritoneal dialysis and hemodialysis in the treatment of diabetes mellitus complicated with nephropathy [J]. Electronic Journal of Clinical Medicine Literature, 2015, 2(15): 2955.

741 Yu Hui. Experimental study on the effect of late oxidized protein products on articular cartilage and synovium in rabbit knee osteoarthritis [D]; Southern Medical University, Surgery (Bone Surgery), 2015.

742 Yu X. Microdialysis method to study the pharmacokinetic effect of ambroxol hydrochloride on moxifloxacin in the lungs of rats with Streptococcus pneumoniae [D]; Second Military Medical University, Internal Medicine (Respiratory Diseases), 2015.

743 Zhang Hongmei, Fan Ruyan, Chang Liyang, et al. Effects of Tai Chi exercise on physical fitness and fatigue of hemodialysis patients [J]. Chinese Journal of Integrative Nephrology, 2015, (9): 807-10.

744 ZHANG Huifang, YANG Jiangcheng, JIANGCHENG Y. Effects of hyperbaric oxygen combined with high-throughput dialysis on serum bone-forming protein-7 expression and bone metabolism in patients with renal bone disease [J]. China Continuing Medical Education, 2015, (32): 38-9.

745 ZHANG Jie, WANG Q. Effect of collaborative care model on self-care ability and quality of life of hemodialysis patients [J]. Chinese Journal of Modern Nursing, 2015, (24): 2885-7,8.

746 Zhang LF, Yan HL, Chen X, et al. Effects of osteotriol on nutritional status, microinflammation and oxidative stress in maintenance hemodialysis patients [J]. Journal of Hainan Medical College, 2015, 21(8): 1062-5.

747 Zhang WB, Dai ZB, Zhou J, et al. Clinical efficacy of leucovorin in the treatment of nondialysis cardiorenal syndrome [J]. Journal of Clinical Rational Drug Use, 2015, (24): 61-2.

748 Zhang XL. Block reducers improve aortic and ventricular remodeling in spontaneously hypertensive rats [D]; Southern Medical University, Internal Medicine (Cardiovascular Disease), 2015.

749 Zhang Y. Study on the inhibitory effect of curcumin-loaded nanomicelles on cervical cancer [D]; Southwest Medical University; Sichuan Medical University, Oncology, 2015.

750 ZHONG J, ZHAO NING-BO, NING-BO Z. Effects of Kidney-boosting and Blood-activating Formula combined with Chlorosartan on Endogenous Hydrogen Sulfide and Inflammatory Cytokines in Maintenance Hemodialysis Patients [J]. Chinese Journal of Experimental Formulas, 2015, 21(2): 196-9.

751 Zhong Lijuan. Basic research on multi-targeted anti-angiogenic nanomedicines applied to ovarian cancer treatment [D]; Southern Medical University, Obstetrics and Gynecology, 2015.

752 Zhou YS. Analysis of the effect of continuous blood purification treatment for patients with acute renal failure after severe burns [J]. Contemporary Medicine Series, 2015, 13(21): 216-7.

753 Zhu Qijin. Effects of melatonin and pirfenidone on peritoneal dialysis-associated peritoneal fibrosis in rats [D]; Anhui Medical University, Internal Medicine (Nephrology), 2015.

754 Zou Lifeng. The effect of implementing synchronized health education for family members of outpatient hemodialysis patients on patient compliance [J]. Chinese and Foreign Medical Treatment, 2015, (24): 63-5.

755 Bao Enhao, Zhu Aiguo, Wang Zhihong, et al. Effect of vitamin C combined with atorvastatin calcium on oxidative stress and microinflammatory status in hemodialysis patients [J]. Journal of Hainan Medical College, 2016, 22(5): 431-4.

756 Cai J. The experience of the effect of exercise therapy on dialysis adequacy in uremic hemodialysis patients [J]. Frontiers of Medicine, 2016, 6(26): 65-6.

757 Cao Hu, Zhu Lingfang, Shi Jianmei, et al. Effects of Renkang injection on oxidative stress and microinflammatory state in maintenance hemodialysis patients [J]. Journal of Changchun University of Traditional Chinese Medicine, 2016, 32(3): 563-5.

758 Cao X. Selection and breeding of lipase-producing bacteria, purification and characterization of enzyme, and the mechanism of enzyme production by two induction methods [D]; Zhejiang University, Food Science, 2016.

759 Zeng Lingling, Qu Suulin, SUI-LIN Q U. Clinical study of hematopoietic tablets combined with erythropoietin and levocanidin in the treatment of renal anemia [J]. Modern Drugs and Clinics, 2016, 31(5): 678-82.

760 Chang Xiaodong, Xue Trace, Yang Youqin, et al. Effects of Cerebral Heart Capsules on Oxidative Stress, Microinflammation, and Carotid Intima-Media Thickness in Maintenance Hemodialysis Patients [J]. Chinese Pharmaceutical Industry, 2016, 25(19): 39-41.

761 Chen Guimei, Zeng Jinliang, Du Jieting, et al. Clinical Observation on Ginger Acupuncture Point Patch with Acupressure for Treatment of Nausea and Vomiting in Hemodialysis [J]. Shenzhen Journal of Integrative Chinese and Western Medicine, 2016, 26(10): 66-7.

762 CHEN Haihui, XU Min, MIN X. Clinical efficacy observation of reduced glutathione combined with recombinant human erythropoietin in the treatment of uremic anemia patients [J]. Zhejiang Medicine, 2016, 38(12): 959-61,1018.

763 Chen J-L, Li W-J, Lu Z, et al. Effectiveness analysis of over-continuous blood purification in the treatment of severe heart failure with renal failure [J]. Huaxia Medicine, 2016, 29(1): 33-5.

764 Chen JX. An overview of the impact of peer education on maintenance hemodialysis patients [J]. Psychologist, 2016, 22(32): 245-6.

765 Chen Sun, Zhang Guojuan, Jiang Liping, et al. Urban-rural differences in bone metabolism indexes of hemodialysis patients in Beijing [J]. China Blood Purification, 2016, 15(2): 121-3.

766 Chen T, Ban Zunpu, Luo Guohong, et al. Analysis of the effect of Bailing capsule on oxidative stress in patients with hemodialysis renal anemia caused by iron sucrose [J]. Practical Chinese and Western Medicine Clinic, 2016, 16(6): 47-8.

767 Chen YM. Influence of exercise therapy on dialysis effect in maintenance hemodialysis patients [J]. Modern Nutrition (Lower Half Monthly Edition), 2016, (10): 123.

768 CHENG Jinxiu, PAN Xing, LIU Cuilan, et al. Effects of telmisartan combined with leucovorin on oxidative stress and microinflammatory status in peritoneal dialysis patients [J]. Journal of Hainan Medical College, 2016, 22(12): 1273-5.

769 Shan DW. Effect of individualized exercise therapy on nutrition and quality of life of uremic hemodialysis patients [J]. China Healthcare Nutrition, 2016, 26(14): 190.

770 Fan Ruyan, Zhang Hongmei, Chang Liyang, et al. Effects of Tai Chi exercise on anxiety and depression status and sleep quality of hemodialysis patients [J]. China Blood Purification, 2016, 15(4): 241-3.

771 Feng Jiali, Liu Cuilan, Liu Hua, et al. Effects of intensive self-empowered nursing care on disease management ability and organism volume status of diabetic nephropathy patients on peritoneal dialysis [J]. Hainan Medicine, 2016, 27(14): 2399-401.

772 Fu Bing. Effect of Tegretol on Coronary Microcirculation after Percutaneous Coronary Intervention in Patients with Acute Coronary Syndrome [D]; Hebei Medical University, Internal Medicine, 2016.

773 Guo Dongmei. Study on the effect of picloram on the expression of Escherichia coli outer membrane protein A [D]; Guangxi Medical University, Nephrology, 2016.

774 GUO Jiaquan, WANG Jin-hong, JIN-HONG W. Effects of aerobic training on cellular immune function after hemodialysis in patients with CKD stage 5 [J]. Chinese Experimental Diagnostics, 2016, 20(12): 2047-9.

775 Guo J. Clinical analysis of 20 cases of acute renal failure complicated by diabetic ketoacidosis [J]. Diabetes New World, 2016, 19(3): 121-3.

776 GUO Zi-Hong, TIAN Jia-Wei, JIA WEI T. A preliminary study of VFM technique combined with PW/TDI synchronization on left ventricular diastolic function in patients with CRF; proceedings of the Chinese Society of Echomedical Engineering 13th National Echocardiography Symposium, Beijing, F, 2016 [C].

777 GUO Zi-Hong, TIAN Jia-Wei, JIA Wei T. A preliminary study of VFM technique combined with PW/TDI synchronization on left ventricular diastolic function in patients with CRF; proceedings of the Chinese Society of Ultrasound Medical Engineering 11th National Abdominal Ultrasound Academic Conference, Dalian, F, 2016 [C].

778 Han Ling. Effects of different dialysis modalities on toxin clearance and inflammatory response [D]; Qingdao University, Internal Medicine (Nephrology), 2016.

779 Han Ling, Hao Lintao, Li Yang, et al. Effects of different dialysis modalities on toxin clearance and inflammatory response [J]. Chinese and Foreign Medicine Research, 2016, 14(13): 1-4.

780 HAN Yan, LI Yong, ZHANG Suli, et al. Efficacy of hemoperfusion tandem with different modes of continuous blood purification on lung injury in paraquat poisoning [J]. Medical Review, 2016, 22(19): 3921-3,4.

781 Hao Qufa. Resveratrol attenuates cisplatin-induced acute kidney injury by inhibiting death receptor apoptosis pathway [D]; Shandong University, Internal Medicine (Nephrology), 2016.

782 Huang Lin, Pang Jie, Li Jinshan, et al. Effects of Bailin capsule combined with urethane Kang Combination on residual renal function and microinflammatory status of peritoneal dialysis patients [J]. Shaanxi Traditional Chinese Medicine, 2016, 37(11): 1477-9.

783 Huang Lingli, Hu Bihen, Cheng Jing, et al. Effect of high flux hemodialysis on vasoactive substances in uremic patients with chronic renal failure [J]. Chinese Rural Medicine, 2016, 23(21): 13-4.

784 Huang Ruo, Lin Xiaomin, Lin Xiaoxia, et al. Effects of low-moderate aerobic exercise on fatigue and sleep quality in maintenance hemodialysis patients [J]. Chinese Primary Medicine, 2016, 23(23): 3569-72.

785 Jiao Yaxing. Intervention effect of aerobic exercise on fatigue status of maintenance hemodialysis patients [J]. Nursing Practice and Research, 2016, 13(2): 46-7.

786 Kong G. Role of IQGAP1 in high glucose-induced podocyte apoptosis and its mechanism exploration [D]; Shandong University, Internal Medicine (Nephrology), 2016.

787 Lan Xi. Preparation of baicalin polylactide hydroxyacetic acid copolymer microspheres and its ex vivo study [D]; Hebei Medical University, Pharmacy, 2016.

788 Li Chunming. Effects of osteotriol shock therapy on microinflammation and oxidative stress status in maintenance hemodialysis patients [J]. Chinese Primary Medicine, 2016, 23(9): 1357-61.

789 Li Gen, Lu Jinlian, Liu Tao, et al. Clinical significance of thyroid function changes before and after hemodialysis in patients with dialysis hypotension [J]. Chinese Journal of Physicians, 2016, 18(5): 717-9.

790 Li Guixia. Clinical Study on the Effect of Solid Kidney Detoxification Solution Colon Dialysis on Renal Function and Microinflammatory State of Patients with Chronic Renal Failure Stage 1~3 [D]; Gansu University of Traditional Chinese Medicine, Clinical of Integrative Medicine and Western Medicine, 2016.

791 Li Guixia, Xu Yun, Su Jianping, et al. Effect of colon dialysis with solid kidney detoxification solution on microinflammatory state of chronic renal failure patients [J]. Chinese Medicine Science and Technology, 2016, 23(5): 512-5.

792 Li J, Ma L, Zhou YH, et al. Observation on the efficacy of antimicrobial drug combination in the treatment of lower respiratory tract infection in hemodialysis patients with chronic renal failure [J]. Chinese Journal of Hospital Infection, 2016, 26(16): 3708-10.

793 LI P, WANG DONGHONG, DONGHONG W. Effects of aerobic exercise on 6-minute walking distance and hemoretin-1 in hemodialysis patients [J]. Tianjin Medicine, 2016, 44(8): 1014-7.

794 Li Xingmei. Effect of nursing intervention on quality of life of maintenance hemodialysis patients [J]. World Digest of Recent Medical Information (continuous type electronic journal), 2016, 16(69): 326-7.

795 Li Yunsheng, Wang Wenlong, Dai Zaiyou, et al. Effects of hyperbaric oxygen on cognitive dysfunction and brain-derived neurotrophic factor in peritoneal dialysis patients [J]. Chinese Journal of Integrative Nephrology, 2016, 17(8): 705-7.

796 LI ZHENG SHENG, LU JING JING, JING JING L. Clinical observation on delaying chronic renal failure by retained enema of Beneficial Kidney Junction and Penetrating Soup combined with packaged aldoxymethane starch; proceedings of the 2016 Academic Annual Meeting of Renal Disease Specialized Committee of the Chinese Society of Integrative Medicine, Shanghai, F, 2016 [C].

797 Liao Q. Research on the clinical application of selective plasma purification technology in non-renal diseases [D]; Southern Medical University, Internal Medicine (Nephrology), 2016.

798 LIN Ju-Ying, LIAO Yu-Juan, DAI Juan, et al. Efficacy and care of hemoperfusion and dialysis filtration for severe hyperlipidemic acute pancreatitis [J]. World Digest of Recent Medical Information (Continuous Electronic Journal), 2016, 16(20): 228-,9.

799 Liu F. Prospective randomized controlled clinical trial of early application of continuous blood purification therapy for severe burns [D]; Third Military Medical University, Surgery (Burns), 2016.

800 LIU Feng, HUANG Zhenggen, PENG Yizhi, et al. Feasibility and efficacy of early continuous blood purification treatment for severe burns in a randomized controlled clinical trial [J]. Chinese Journal of Burns, 2016, 32(3): 133-9.

801 LIU Lichun, SUN Yuyan, ZHANG Yuhui, et al. Analysis of risk factors and quality of life survey of uremic encephalopathy patients in Weifang [J]. Journal of Weifang Medical College, 2016, 38(4): 261-5.

802 Liu Muqin. Clinical nursing analysis of herpes zoster infection of internal fistula limb in elderly maintenance hemodialysis patients [J]. Everybody's Health (Late Edition), 2016, 11(6): 201.

803 LIU YQ, LIAO D, DAN L. Effects of anthocyanin soft capsules on serum PON1 and AOPP and left ventricular hypertrophy in diabetic nephropathy patients on maintenance hemodialysis [J]. China Health Nutrition, 2016, 26(29): 9-10.

804 LIU Linwei, SHAO Ning, SUN Zhihua, et al. Clinical observation of musk cardioprotective pills in the treatment of chronic heart failure in patients on maintenance hemodialysis [J]. International Journal of Urology, 2016, 36(2): 237-9.

805 Lu Lina, Cai Yanju, Liu Mengying, et al. Exploring the effect of nursing intervention on the psychology of uremic hemodialysis patients [J]. China Health Standard Management, 2016, 7(15): 257-8.

806 Meng Xuchu. Role of Rho/ROCK signaling pathway in peritoneal fibrosis in peritoneal dialysis rats and the intervention effect of fasudil [D]; Guizhou Medical University, Internal Medicine (Nephrology), 2016.

807 PENG Hui, WANG Changjiang, CHEN Jie, et al. Effects of evidence-based nursing on psychological and survival quality of hemodialysis patients with heart failure [J]. Zhongguo Nation Health Medicine, 2016, 28(7): 110-2.

808 Qian Xiaofang. Observation on the efficacy of auricular acupressure combined with Yongquan acupressure on insomnia in patients undergoing maintenance hemodialysis [D]; Chengdu University of Traditional Chinese Medicine, Clinics of Integrative Medicine and Western Medicine, 2016.

809 Qiao J. Application effect of strengthening health education on hemodialysis patients [J]. China Modern Drug Application, 2016, 10(2): 239-40.

810 Shang Y, Guan Shuangcheng, Li Yue, et al. Dexmedetomidine preconditioning inhibits glutamate release during cerebral ischemia-reperfusion and its receptor mechanism [J]. Chinese Journal of Modern Medicine, 2016, 26(4): 1-4.

811 Shi Qiuying, Zang Ge, Xu Sweet, et al. The effect of Five Animal Play on somatic function and quality of life of maintenance hemodialysis patients; proceedings of the 3rd Chinese Medicine Science Conference, Huizhou, Guangdong, F, 2016 [C].

812 ZEN Wei-Zhi. Study on the isolation and purification of Dendrobium officinale polysaccharides and the mechanism of regulating glucose metabolism disorders [D]; Huazhong University of Science and Technology, Pharmacy, 2016.

813 Song Junjun. Observation on the efficacy of ustekin combined with hemodialysis in the treatment of diabetic ketoacidosis [J]. Modern Drugs and Clinics, 2016, 31(12): 1950-4.

814 Su Sheng, Cao YF, Wang HJ, et al. Effectiveness of hemoperfusion tandem dialysis in the treatment of acute paraquat poisoning [J]. Journal of Tropical Medicine, 2016, 16(8): 1023-5,36.

815 Sun Jiajia. Effects of exercise intervention during hemodialysis on activity function and health status of MHD patients [D]; Suzhou University, Nursing, 2016.

816 Qin Jolian. Effect of high osmotic colonic dialysis with herbal enema on chronic renal insufficiency CKD stage 3-4 based on the theory of intestinal and renal axis; proceedings of the 2016 Academic Annual Meeting of Renal Disease Specialized Committee of Chinese Society of Integrative Medicine, Shanghai, F, 2016 [C].

817 Tang DD. Mechanism of apoptosis induced by hypochlorite-modified albumin in diabetic rat foot cells and the protective effect of mitochondria-targeted peptides [D]; Southern Medical University, Internal Medicine (Nephrology), 2016.

818 TENG Lanbo, CHANG Ming, LIU Shuxin, et al. Effects of dialysate with different calcium ion concentrations on peritoneal tissue injury and signs of peritoneal inflammation [J]. China Blood Purification, 2016, 15(10): 554-8.

819 Wang HM. Predictive significance of erythrocyte volume distribution width to platelet count ratio on myocardial perfusion level in patients undergoing direct PCI for STEMI [D]; Hebei Medical University, Internal Medicine, 2016.

820 Wang J. Risk factors for inter-dialysis hyperkalemia in maintenance hemodialysis patients and the impact on prognosis [D]; Dalian Medical University, Internal Medicine, 2016.

821 Wang, Y. J., Wang, L., Lv, S. L., et al. Research and clinical application of a new patent for an ankle pump exerciser; proceedings of the 2016 Annual Meeting of the China Hospital Association Blood Purification Center Management Branch and the Eighth China Blood Purification Forum, and the Annual Meeting of the Jiangsu Provincial Hospital Association Blood Purification Center Branch, Nanjing, F, 2016 [C].

822 Wang Yanlei. Interference of male yellow with glutathione synthesis in mouse hippocampal tissue induces neurotoxicity and the protective mechanism of glycyrrhizic acid [D]; China Medical University, Public Health and Preventive Medicine; Health Toxicology, 2016.

823 Wang Yichun. Effects of aerobic exercise workout on mood, sleep and muscle strength in uremic hemodialysis patients [J]. General Practice Nursing, 2016, 14(33): 3526-7.

824 Wang Yu. A Practical Study of Health Promotion and Intervention for Kidney Transplant Recipients in Xinjiang [D]; Xinjiang Medical University, Labor Health and Environmental Hygiene, 2016.

825 Gui Zhengnan, Zhang Qinghong, Wu Dong, et al. Effects of high flux hemodialysis on plasma toxin molecule content and microinflammatory state of the organism in uremic patients [J]. Journal of Hainan Medical College, 2016, 22(6): 558-61.

826 Wei Meidan. Study on the effect of colonic dialysis on chronic renal insufficiency based on the theory of "intestinal-renal axis"; proceedings of the 2016 Academic Annual Meeting of Renal Disease Specialized Committee of Chinese Society of Integrative Medicine, Shanghai, F, 2016 [C].

827 Xin Lin. Observations on the impact of health education on treatment adherence of maintenance hemodialysis patients [J]. Primary Medical Forum, 2016, 20(15): 2105-6.

828 Xiong D, Hu W, WEI H. Exploring the effects of high flux hemodialysis (HFHD) on oxidative stress and microinflammatory status in hemodialysis patients with diabetic nephropathy; proceedings of the 2016 Academic Annual Conference of Chinese Journal of Hospital Pharmacy, Kunming, F, 2016 [C].

829 Xu, Sweetie. An intervention study of aerobic-resistance exercise on somatic function and quality of life in maintenance hemodialysis patients [D]; Zhengzhou University, Nursing, 2016.

830 Yan Erping, Qiu Moyan, Ren Jianwei, et al. The effect of evidence-based moxibustion regimen on musculomotor rehabilitation of maintenance hemodialysis patients [J]. Journal of Rehabilitation, 2016, 26(4): 6-10,6.

831 Yan Ye. Analyzing the composition of kinetic functional drink and its effect on small intestinal metabolism in aerobically active rats using microdialysis [D]; South China Normal University, Exercise Human Science, 2016.

832 YANG Chenglin, ZHENG Zhongai, XIAO Bin, et al. Effects of hemodialysis on blood biochemical indexes and cardiac function in patients with refractory heart failure combined with uremia [J]. Medical Review, 2016, 22(7): 1412-4.

833 YANG Jing, JIANG Wenyong, YU Qian, et al. Effects of high flux hemodialysis on oxidative stress and microinflammatory status in maintenance hemodialysis patients with diabetic nephropathy [J]. Guangdong Medicine, 2016, 37(18): 2784-6.

834 Ye Yuanfang. Application effect of comfort care model in the care of hemodialysis patients [J]. Medical Equipment, 2016, 29(14): 194-,5.

835 YI Ye, LU Yuanhang, JI Qianqian, et al. Comparison of the effects of hematopoietin and iron sucrose on anemia, oxidative stress and microinflammation in hemodialysis patients [J]. China Pharmacy, 2016, 27(5): 660-2.

836 YIN S, WAN F, WAN F. Effect of exercise during dialysis on microinflammatory status in maintenance hemodialysis patients [J]. Modern Clinical Nursing, 2016, 15(8): 54-8.

837 Yu Yanmin. Effect of recombinant human brain natriuretic peptide on cardiac function in patients with ejection fraction preserved heart failure [D]; Hebei Medical University, Internal Medicine, 2016.

838 YUE Wenjing, WANG Lu, WANG L. Effects of spironolactone on patients' cardiovascular function in maintenance hemodialysis [J]. Chongqing Medicine, 2016, 45(36): 5118-20.

839 Zhangjiaoli, Xu Zhaoyi, Ma Xiuning, et al. The relationship between kidney and lung in traditional Chinese medicine from the changes of lung function in uremic patients [J]. Chinese Geriatric Healthcare Medicine, 2016, 14(4): 18-9.

840 Zhang JX. The effect of intensive self-empowered care on the disease management ability of dialysis patients with diabetic nephropathy line [J]. Psychiatrist, 2016, 22(26): 207-8.

841 Zhang J. A study of self-management behavioral intervention for maintenance hemodialysis patients [D]; Nanhua University, Nursing, 2016.

842 Zhang QB. Role of Caveolin-1/NF-κB/HMGB1 pathway in endothelial injury and mechanism of EPC protection by probucol in hyperlipidemia [D]; Shandong University, Internal Medicine (Cardiovascular Disease), 2016.

843 Zhang, X. Q., Li, W. B., Tian, Z. T., et al. Application and care of hemodialysis perfusion in the treatment of severe organophosphorus pesticide poisoning; proceedings of the 10th National Congress of Critical Care Medicine of the Chinese Medical Association, Zhengzhou, F, 2016 [C].

844 Zhang Yuchuang. Observation on the efficacy of recombinant human erythropoietin and iron sucrose combined with levocanidin in the treatment of renal anemia [J]. Practical Chinese and Western Medicine Clinic, 2016, 16(12): 8-9,28.

845 ZHAO Lina, YAN Haibing, NIU Kechao, et al. Protective effect of ustekin combined with continuous blood purification on myocardial injury in patients with severe sepsis [J]. Medical Clinical Research, 2016, 33(4): 644-6,9.

846 ZHOU Ruiling, ZHOU Ping, LAI Qi, et al. Study on the effect of nursing intervention combined with exercise therapy on the quality of life of hemodialysis patients [J]. Guangxi Medicine, 2016, (1): 134-6,9.

847 ZHU L H, ZHU JIU YI, JIU YI Z. Clinical observation of moxibustion combined with aerobic exercise to improve depressive symptoms in hemodialysis patients treated with fluoxetine [J]. Hunan Journal of Traditional Chinese Medicine, 2016, 32(2): 95-7.

848 Ai Ling. Effect of hemoperfusion combined with sequential dialysis on inflammatory mediators and oxidative stress in patients with end-stage diabetic nephropathy [J]. Modern Medicine, 2017, 45(2): 174-9.

849 Zeng YQ. Molecular mechanism of the effects of rhubarb and its active substances by enema on rats in a chronic kidney disease model [D]; Guangzhou University of Traditional Chinese Medicine, Internal Medicine of Traditional Chinese Medicine, 2017.

850 SHEN Yuanli, SHAO Haiying, CHEN Kai, et al. A controlled study of the effect of intermittent and continuous dialysis on hemodynamics in patients with end-stage renal disease combined with cerebral hemorrhage [J]. Chinese Journal of Practical Neurological Diseases, 2017, 20(13): 5-8.

851 Chen SB. Feasibility study of targeting and regulating nuclear receptor FOXO1 to prevent cholesterol stone formation [D]; Jiangsu University, Clinical Laboratory Diagnostics, 2017.

852 CHEN Yongjian, LU Faju, YANG Lei, et al. Effects of Renkang Injection on Oxidative Stress and Microinflammatory State in Elderly Patients on Maintenance Hemodialysis [J]. Shaanxi Traditional Chinese Medicine, 2017, 38(8): 1061-2.

853 CHEN Zhuqing, ZHANG Rongguo, RONG-GUO Z. Effects of recombinant human erythropoietin beta injection on SOD, GSH-PX, MDA and Hcy levels in diabetic peritoneal dialysis patients [J]. Chinese Journal of Biochemical Drugs, 2017, 37(2): 136-8.

854 CHENG Jinxiu, LU Jifang, LI Yuexin, et al. Effects of urethane particles on oxidative stress status and VEGF Hcy in peritoneal dialysis patients with diabetic nephropathy [J]. Hebei Medicine, 2017, 23(6): 1014-6.

855 Cheng Xiaojuan, Huang Haiping, Li Zhengsheng, et al. Clinical observation on delaying chronic renal failure by retained enema of Beneficial Kidney JieTuan Tang combined with packaged aldoxymethane starch; proceedings of the inaugural meeting of the Blood Purification Professional Committee of Guizhou Society of Integrative Medicine and Western Medicine and the exchange of national famous old Chinese medicine practitioners' experience inheritance, Guiyang, F, 2017 [C].

856 Cui Dongfeng. Clinical randomized controlled study of hemodialysis and peritoneal dialysis for diabetic nephropathy [J]. Modern Diagnosis and Treatment, 2017, 28(9): 1674-5.

857 Dai F, Zhang WX, Xiao YM, et al. Efficacy and mechanism of enema-assisted colonic dialysis in the treatment of chronic renal insufficiency by the method of consolidation and drainage [J]. Medical Clinical Research, 2017, 34(8): 1594-6.

858 Deng Kaihui, Guo Xiangqian, Zhang Li, et al. Application and effect of telephone follow-up specialized management in patients discharged from hospital after internal fistula surgery [J]. Contemporary Nurses (Zhongdian Journal), 2017, (12): 136-8.

859 Deng Xufang. Study on the effect of HIF-1α mutants on angiogenesis and cardiac function in ischemic myocardium [D]; Guangzhou Medical University, Internal Medicine, 2017.

860 DONG BOSU, WANG YANPING, CHENG YINGYING, et al. The role of weibo platform education in self-management of young and middle-aged maintenance hemodialysis patients [J]. China Rural Health Care Management, 2017, 37(4): 440-2.

861 Duan Leilei. Clinical study of postoperative respiratory management and circulatory support in congenital heart disease [D]; Peking Union Medical College; Chinese Academy of Medical Sciences; Tsinghua University School of Medicine; Peking Union Medical College Chinese Academy of Medical Sciences, Perioperative Medicine, 2017.

862 Fan H., Ren Z., Li W. R., et al. Pathogenesis of hypertension in chronic renal failure and its treatment analysis [J]. Chinese and Foreign Women's Health Research, 2017, (19): 36,8.

863 Feng Xiaodong, Zhang Shiyang, Xiang Rong, et al. Clinical observation of hemoperfusion combined with hemodialysis filtration for hyperlipidemic acute pancreatitis [J]. Sichuan Medicine, 2017, 38(3): 316-8.

864 Feng Xue. Action research-based home aerobic exercise intervention for maintenance hemodialysis patients [D]; Ningxia Medical University, Nursing, 2017.

865 Fu Qunying, Lei Lei, Wang Ying, et al. Effects of Ginkgo biloba capsules on lipid metabolism disorders, vascular endothelial function, coagulation function, and major cardiovascular and cerebrovascular adverse events in patients with end-stage renal disease on maintenance hemodialysis [J]. Journal of Modern Integrative Chinese and Western Medicine, 2017, 26(29): 3215-8.

866 Gao Q, Wu N, Zhu Y, et al. Effects of recombinant human erythropoietin-β injection on inflammatory factor levels and oxidative stress levels in peritoneal dialysis patients with diabetic nephropathy [J]. World Clinical Drugs, 2017, 38(9): 629-33.

867 Gao Song. Analysis of the efficacy of intermittent hemodialysis plus perfusion combined with atropine in the treatment of organophosphorus poisoning [J]. Everybody's Health (Late Edition), 2017, 11(3): 132-3.

868 Gou Yunyun. The effect of health education pathway in the care of uremic patients [J]. Health Care Guide, 2017, (3): 52.

869 GU Jinhua, ZHAO Ping, WU Yalin, et al. Efficacy and safety analysis of glucosamine hydrochloride tablets in the treatment of uremic knee osteoarthritis [J]. West China Medicine, 2017, 32(8): 1217-20.

870 Guo Qian. Effect of comprehensive nursing care on psychological status and quality of life of hemodialysis patients [J]. Health Frontier, 2017, 26(6): 35-6.

871 GUO Shuzhen, YANG Yang, YANG Z. Clinical study of peripheral neuropathy in uremic patients by combination of levocanidin and hemodialysis [J]. Heilongjiang Medicine, 2017, 30(4): 817-9.

872 GUO Zi-Hong, TIAN Jia-Wei, JIA Wei T. Preliminary study of left ventricular diastolic function in patients with chronic renal insufficiency by blood flow vector imaging combined with PW/TDI synchronization [J]. Chinese Journal of Circulation, 2017, 32(z1): 199.

873 HAN Xiaowen. Effects of high-throughput hemodialysis on oxidative stress and microinflammatory status in maintenance hemodialysis patients with diabetic nephropathy [J]. Diabetes New World, 2017, 20(18): 1-2.

874 Han XT. Analysis of the application of clinical pathway in hemodialysis nursing; proceedings of the Chinese Nursing Society 15th National Blood Purification Nursing Academic Exchange Conference, Changsha, F, 2017 [C].

875 Hong Liping, Zhang Ting, Xiao Aihua, et al. Clinical value analysis of continuous blood purification for the treatment of infectious shock with renal dysfunction [J]. Clinical Research, 2017, 25(7): 56,8.

876 Hou Dandan, Wang Shuling, Shang Sainan, et al. Effects of levocanidin adjuvant therapy on EPO resistance and oxidative stress and inflammatory response in hemodialysis patients [J]. Journal of Hainan Medical College, 2017, 23(15): 2056-9,63.

877 Huang Yuming. Study on the factors influencing the formation of biofilm of Escherichia coli in vitro by Picrasidine [D]; Guangxi Medical University, Internal Medicine, 2017.

878 Jiang Q. Effects of hematopoietin and iron sucrose combined with leucovorin on oxidative stress and erythrocyte parameters in patients with MHD anemia [J]. Medical Clinical Research, 2017, 34(9): 1776-8.

879 Jiang Yang. Studies on the properties of apple and hawthorn pectin and its application in fermented milk [D]; Shandong Agricultural University, Food Science, 2017.

880 Jiang Xuan. Study on the cerebroprotective effect of hibernation-inducing factor on deep hypothermia arrested circulation in rats [D]; China Medical University, Surgery 学(心胸外科), 2017.

881 Kang, W.L.. A cohort study of early hemodialysis patients combined with NTIS pharmacological intervention [D]; Nanchang University; Nanchang University School of Medicine, Internal Medicine (Nephrology), 2017.

882 LI Guozhu, XU Hui, HUI X U. Clinical analysis of dialysis patterns in diabetes mellitus combined with end-stage renal disease [J]. China Continuing Medical Education, 2017, 9(13): 86-7.

883 Li Jian, Li Huanyi, Zhi Weihua, et al. Observation on the efficacy of CRRT in the treatment of chronic renal failure combined with heart failure [J]. China Practical Medicine, 2017, 12(33): 83-4.

884 Li L. Effects of compound alpha keto acids combined with conventional nutritional intervention on inflammatory response and oxidative stress in maintenance hemodialysis patients [J]. Journal of Hainan Medical College, 2017, 23(15): 2060-3.

885 Li Na. Role and mechanism of SIRT3 in renal damage in hypertensive mice [D]; Shandong University, Internal Medicine (Cardiovascular Disease), 2017.

886 Li Qiaoyun. Analysis of nursing measures to improve the quality of life of hemodialysis patients with diabetic nephropathy [J]. Dietary Health Care, 2017, 4(8): 160.

887 Li Yiming, Zhang Ling, Xing Xiaozhen, et al. Effect of vitamin C combined with vitamin E on antioxidant power and inflammatory response status of hemodialysis patients [J]. Journal of Hainan Medical College, 2017, 23(19): 2635-7,41.

888 Li Z-Tao. Genetically modified VEGF vaccine combined with doxorubicin for the treatment of hepatocellular carcinoma in mice [D]; Huazhong Agricultural University, Microbiology, 2017.

889 Lin KW. A non-randomized controlled study of the effect of eight-duanjin on the clinical efficacy of peritoneal dialysis patients [D]; Guangzhou University of Traditional Chinese Medicine, Traditional Chinese Medicine and Health Sciences, 2017.

890 Lin Xiangming, Liu Xiaoli, Shi Kaixuan, et al. Experimental study on the inhibition of the excitotoxic effect of striatal glutamate by exercise through modulating endogenous cannabinoid system in rats with Parkinson's disease model [J]. Journal of Tianjin Sports Institute, 2017, 32(3): 261-8.

891 LIN Ye, CHEN Wen, ZHUANG Yijun, et al. Effects of 20 and 40 mg simvastatin combined with chlorosartan potassium orally on oxidative stress and inflammatory factor levels in peritoneal dialysis patients [J]. Shandong Medicine, 2017, 57(39): 79-81.

892 LIU Cuilan, LIU Hua, CHENG Jinxiu, et al. Effects of osteotriol combined with calcium hydroxybenzenesulfonate on microinflammation, nutritional status and quality of life in patients maintained on peritoneal dialysis for diabetic nephropathy [J]. Journal of Modern Integrative Medicine, 2017, 26(11): 1179-81.

893 Liu, Hongchao. Extraction and isolation, bioactivity and structural characterization of polysaccharides from Lamb's quarters [D]; Shanghai Ocean University, Food Science and Engineering, 2017.

894 LIU Huizhen, JIA Qiang, ZHENG Lin, et al. Effects of different hemodialysis membranes on pulmonary function in uremic patients [J]. Chinese Journal of Practical Diagnosis and Therapy, 2017, 31(11): 1122-5.

895 Liu Junli. OHA-COL II bionic gel composite with autologous concentrated bone marrow nucleated cells for repairing cartilage defects in porcine knee joints [D]; Chinese People's Liberation Army Army Medical University; Third Military Medical University, Surgery (Orthopaedic Surgery), 2017.

896 Liu Shuyan. Ginkgo biloba extract combined with valsartan in the treatment of type 2 diabetic nephropathy and effects on sTWEAK, NLR and IL-8 [D]; Zhengzhou University, Clinical Integrative Medicine, 2017.

897 Liu Y. Intervention study of resistance exercise on dialysis hypotension in hemodialysis patients [D]; Dalian Medical University, Nursing, 2017.

898 Liu Yingying, Wang Chen, Luo Pengfei, et al. Construction of myocardium-targeting nanoparticles and its preliminary study on prevention of myocardial injury in mice [J]. Chinese Journal of Burns, 2017, 33(11): 660-7.

899 Lu Yao, Cai Fenfang, Zhou Huahui, et al. Hospital infection control management in maintenance hemodialysis patients [J]. Nursing Practice and Research, 2017, 14(12): 15-7.

900 Lv Liqiao. Effects of exercise on symptomatic hypotension and dialysis adequacy during dialysis [J]. Zhejiang Clinical Medicine, 2017, 19(5): 923-4.

901 Lv Yanhua, Zhang Yanping, Chen Yingchun, et al. Clinical efficacy and mechanism analysis of hemodialysis combined with traditional Chinese medicine formula granules for diabetic nephropathy [J]. 大家健康（下旬版）, 2017, 11(12): 75.

902 Ma Jingui, Tian Gong, Zhao Xiurong, et al. Study on the effect of high flux hemodialysis (HFHD) on oxidative stress and microinflammatory status in hemodialysis patients with end-stage diabetic [J]. Diabetes New World, 2017, 20(22): 159-60.

903 Ma Lei. Analysis of clinical value of continuous blood purification for treatment of infectious shock with renal dysfunction [J]. China Medical Guide, 2017, 15(13): 25,7.

904 MA Shixing, CHEN Weihong, WEIHONG C. Effects of adjuvant treatment with Benefiting Kidney and Activating Blood Soup on carotid intima-media thickness, oxidative stress indexes, and microinflammatory status in patients undergoing maintenance hemodialysis [J]. Journal of Liaoning University of Traditional Chinese Medicine, 2017, 19(12): 121-3.

905 Ma Yanping. Clinical effects of nursing intervention and dialysis therapy in acute renal failure and multifunctional disorder syndrome [J]. Special Health, 2017, (18): 160-1.

906 Ma Zhi-Hong, Hu Wen-Bo, Jia Shun-Lian, et al. Effects of high-flux versus low-flux hemodialysis on oxidative stress, cellular immune function, and microinflammatory status in dialysis patients with diabetic nephropathy [J]. Advances in Modern Biomedicine, 2017, 17(30): 5963-6.

907 Peng Fumei. Impact of extended care model on peritoneal dialysis patients; proceedings of the Hubei Provincial Medical Association Nephrology Branch 2017 Annual Academic Conference, Wuhan, F, 2017 [C].

908 Qiqig. Effects of different exercise modalities on dialysis adequacy and quality of life in maintenance hemodialysis patients [D]; Inner Mongolia Medical University, Internal Medicine, 2017.

909 Qiao Fengjie. The preventive effect of remote ischemic preconditioning on contrast nephropathy [D]; Taishan Medical College, Internal Medicine, 2017.

910 Qu Chuanna. Effects of leucovorin and iron sucrose on renal anemia and oxidative stress in elderly hemodialysis patients [J]. Medical Information, 2017, 30(4): 136-7.

911 REN Kejun, WANG Xiaoqin, XIAOQIN W. Meta-analysis of the effectiveness of exercise therapy in the treatment of restless legs syndrome on hemodialysis [J]. Anhui Medicine, 2017, 21(1): 82-7.

912 Kamiyuki, Masahiro. Renal rehabilitation: future progress and outlook; proceedings of the 12th Beijing International Rehabilitation Forum, Beijing, F, 2017 [C].

913 Sun Danqin. Effects of rehabilitation exercise on cardiac function and quality of life of maintenance hemodialysis patients [J]. Biped and Health Care, 2017, 26(10): 53,5.

914 TANG Chaoxia, WANG Liping, TANG Yidan, et al. Clinical therapeutic effects of clinical continuity of care for renal failure patients on hemodialysis [J]. Mother and Child World, 2017, (15): 133.

915 TANG Guanying, WANG Hongyu, CHU Xiulin, et al. Effects of high flux dialysis on depressive state in maintenance hemodialysis patients [J]. Neurological Diseases and Mental Health, 2017, 17(10): 713-6.

916 Tang Liang, Ma Luping, Hua Jianwu, et al. Clinical effect of cefazolin sodium pentahydrate combined with levofloxacin in the treatment of central venous catheter infection in hemodialysis patients [J]. Chinese Medicine, 2017, 12(9): 1386-8.

917 Tang Xue. Effect of continuous hemofiltration and hemoperfusion combined therapy on the efficacy and inflammatory factors of long-term hemodialysis patients [J]. Journal of Practical Hospital Clinics, 2017, 14(5): 122-5.

918 Teng Cuiqin. Exploring the application effect of seamless nursing care in patients with first-time establishment of endovascular fistula in maintenance hemodialysis [J]. Systemic Medicine, 2017, 2(24): 130-1,4.

919 TIAN Meiling, WU Lijun, LI JUN W. The value of self-exercise nursing guidance for hemodialysis patients [J]. Medical Information, 2017, 30(7): 166-7.

920 Tian T. Effects and mechanisms of intermittent alkali stimulation on high phosphorus-induced calcification in rat vascular smooth muscle cells [D]; Hebei Medical University, Internal Medicine, 2017.

921 Wan Li-Feng. Impact of home care-based nursing intervention on quality of life and self-care ability of maintenance hemodialysis patients [J]. Clinical Medical Engineering, 2017, 24(7): 1019-20.

922 Wang F. Study on the effect of Ha-PTX-P85-SLN on multidrug resistance in cervical and breast cancer [D]; Shandong University, Gynecology, 2017.

923 Wang F, Liu J, Huang ZJ, et al. Effect of continuous hemodialysis filtration on pulmonary function in patients with sepsis complicating postoperative polytrauma [J]. Journal of Practical Heart, Brain, Lung and Vascular Disease, 2017, 25(6): 96-9.

924 Wang H. Effect of low-intensity aerobic training combined with health education on complication rate and quality of life of maintenance hemodialysis patients [J]. Nursing Practice and Research, 2017, 14(19): 31-3.

925 Wang, L. L.. Application of molecular evolutionary approaches to modify the binding properties of Staphylococcus aureus protein A [D]; Chinese People's Liberation Army Naval Medical University; Second Military Medical University, Microbiology, 2017.

926 WANG PENG, YAN HUI FANG, HUI FANG Y. Clinical observation of applying the concept of knowing-trusting-acting to improve the quality of life of patients with secondary hyperparathyroidism [J]. China Health Nutrition, 2017, 27(2): 107-8.

927 Wang T, Zhang F, Wei M, et al. Effects of adjuvant therapy with reductive glutathione on oxidative stress and inflammatory response in maintenance hemodialysis patients in vivo [J]. Journal of Hainan Medical College, 2017, 23(10): 1337-40.

928 WANG Wenzhe, MAO Huihui, CHEN Wenli, et al. Observation on the therapeutic effect of the addition and subtraction of Strengthening the Spleen and Benefiting the Kidney Formula in the treatment of gastrointestinal dysfunction in peritoneal dialysis patients [J]. Shizhen Guojian Guojian, 2017, 28(5): 1144-5.

929 Wang, S. App. Nursing interventions for renal bone disease in patients treated with long-term maintenance hemodialysis; proceedings of the 17th East China Nephrology Forum and Shandong Province Nephrology Annual Conference, Jinan, F, 2017 [C].

930 Wang Xiaoliang, Sun Yuman, Chen Lin, et al. Effects and mechanism of action of Renal Failure Ning Tablets on the regression of CKD3～ stage 4 patients [J]. Chinese Journal of Experimental Formulary, 2017, 23(3): 159-64.

931 Wang Ying. Therapeutic effect of scAAV9-VEGF-165 on ALS transgenic mice and its mechanism of action [D]; Hebei Medical University, Neurology, 2017.

932 WEI Jianbo, NING Fangfang, LU Qinyan, et al. Effects of osteotriol shock therapy on microinflammatory reaction state and oxidative stress indexes in maintenance hemodialysis patients [J]. Jilin Medicine, 2017, 38(7): 1268-70.

933 Wen Yang. Effect of low-calcium dialysate on coronary artery calcification and safety in maintenance hemodialysis patients [D]; Chongqing Medical University, Internal Medicine, 2017.

934 Xian, Xiaohui. Sulbactam participates in the induction of cerebral ischemic tolerance in rats by mediating GLT-1 upregulation via the p38 MAPK pathway [D]; Hebei Medical University, Pathology and Pathophysiology, 2017.

935 Xie Lijuan. Application of intensive self-care to volume loading and self-care behavior of peritoneal dialysis patients with diabetic nephropathy [J]. China Healthcare Nutrition, 2017, 27(10): 204.

936 XIE MINGBEI, WANG FANG, FANG W. The effect of sodium betaprost on pulmonary arterial hypertension in patients on maintenance hemodialysis [J]. Shenzhen Journal of Integrative Chinese and Western Medicine, 2017, 27(21): 9-10.

937 H. Xu, H. Ping, X. Li, X. Li, L. Sun, et al. Effects of levocanidin on inflammatory factors, oxidative stress indexes, and cardiac function in maintenance hemodialysis patients [J]. China Pharmacy, 2017, 28(11): 1558-61.

938 Xu Liping. Comprehensive nursing care to improve adherence of uremic patients on hemodialysis [J]. China Medical Guide, 2017, 15(28): 233-4.

939 Xu Shuxian. Effect and mechanism of hyperbaric oxygen pre-adaptation on endometriosis in rats [D]; Suzhou University, Obstetrics and Gynecology, 2017.

940 Yang Q. Effects of different dialysis membranes on nutritional status and survival quality of maintenance hemodialysis patients [J]. China Medical Engineering, 2017, 25(7): 79-82.

941 YANG Luyan, LIAO Wensheng, TAN Xiaoyan, et al. Analysis of the efficacy of high flux dialysis combined with bezperidin on pulmonary hypertension complicated by maintenance hemodialysis patients [J]. Heilongjiang Medicine, 2017, 41(2): 113-4.

942 YANG Min, JIANG Hongzhen, HE Zhenkun, et al. Study of enteral nutrition on immune function and oxidative stress status in elderly uremic dialysis patients [J]. Chongqing Medicine, 2017, 46(32): 4566-9.

943 Yin Yaoyao, Wang Junxia, Ma Navy, et al. Effects of lipoic acid on oxidative stress and microinflammatory state in hemodialysis patients [J]. Journal of Medicine Forum, 2017, 38(8): 36-8.

944 YIN Lixia, HU Xiaoyan, ZHANG Hailin, et al. Effects of exercise in dialysis on dialysis adequacy and sleep quality in maintenance hemodialysis patients [J]. China Nursing Management, 2017, 17(11): 1478-81.

945 Yu Pengfei. Clinical effect observation of hydroxyethyl starch for extracorporeal circulation precharge in cardiac surgery [D]; Chinese People's Liberation Army Air Force University of Military Medicine; Fourth Military Medical University, Surgery (Cardiovascular Surgery), 2017.

946 Zhang B, Tang N, Xiong Y, et al. Effect of low concentration of calcium dialysate on secondary hyperparathyroidism and carotid atherosclerosis in maintenance hemodialysis patients [J]. Hebei Medicine, 2017, 23(2): 243-7.

947 Changwei Zhang. Research on the isolation, preparation and bioactivity of ginkgo biloba lipids [D]; China Academy of Forestry Sciences, Forest Chemical Processing Engineering, 2017.

948 Zhang Lan, Cheng Yanjiao, Zhao Xinju, et al. Study on the effects of recumbent gymnastics on toxin clearance and inflammatory status in maintenance hemodialysis patients [J]. China Blood Purification, 2017, 16(4): 247-50.

949 Zhang Li. Chronic inflammatory state and anemia in chronic kidney disease; proceedings of the Ninth China Blood Purification Forum and 2017 Annual Meeting of China Hospital Association Blood Purification Center Management Branch, Beijing, F, 2017 [C].

950 Zhang Lu. Study on the molecular mechanism of astragaloside intervening in the phenotypic transformation of peritoneal mesothelial cells [D]; Nanjing University of Traditional Chinese Medicine, Clinics of Integrative Medicine and Western Medicine, 2017.

951 ZHANG Shengqin, LIAO Qiaozhen, QIAO ZHEN L. Impact of continuity of care model on the nursing effect of continuous peritoneal dialysis [J]. Chinese and Foreign Medical Research, 2017, 15(30): 112-3.

952 Zhang JJ. Meta-analysis of the effect of blood activating and blood stasis removing drugs on the maturation of arteriovenous endovascular fistulae in patients with uremia [D]; Hubei University of Traditional Chinese Medicine, Clinic of Integrative Medicine and Western Medicine, 2017.

953 Zhang Yawen. The impact of integrated care on the production of foot health behaviors in patients with diabetic nephropathy on maintenance hemodialysis [J]. Biped and Health Care, 2017, 26(4): 38-9.

954 Zhao Huiping, Li Shaomei, Xing Changwing, et al. Efficacy and safety study of Jerry's automated peritoneal dialysis machine for the treatment of maintenance peritoneal dialysis patients:a multicenter, randomized, two-phase crossover clinical trial [J]. China Blood Purification, 2017, 16(3): 148-53.

955 ZHAO Jinxiang, LI Yaohua, XIE Ping, et al. Effects of huangkui capsule on microinflammation and oxidative stress status in hemodialysis patients with diabetic nephropathy [J]. China Journal of Traditional Chinese Medicine Information, 2017, 24(2): 21-4.

956 Zhao, L.S.. Supramolecular structure of photosynthetic phycobilisome-vesicle membranes of red algae and their response to nitrogen deficiency [D]; Shandong University, Microbiology, 2017.

957 ZHAO Wanzeng, HAO Yujie, YIN Xiaowen, et al. Clinical study on the treatment of depression in hemodialysis patients with combination of Chinese and Western medicine [J]. Journal of Chinese Medicine, 2017, 32(8): 1523-6.

958 ZHAO YIXIN, LI MINGXU, MINGXU L I. Effect of adjuvant treatment with levocanidin on anemia and organ function damage in maintenance hemodialysis [J]. China Medicine Herald, 2017, 14(3): 156-9.

959 Zheng S. Preparation of Poria cocos polysaccharide polylactic acid nanoparticles and its immune-enhancing effect [D]; Nanjing Agricultural University, Veterinary Medicine; Clinical Veterinary Medicine, 2017.

960 Zheng Xuan. Impact of peer education model on self-management behavior and management effect of hemodialysis patients; proceedings of the Hubei Provincial Medical Association Nephrology Branch 2017 Academic Annual Meeting, Wuhan, F, 2017 [C].

961 Zhou Lulu. T.g HSP70 Induces Acute Liver Injury in Toxoplasma gondii Infected Mice via TLR4/MAPK Signaling Pathway [D]; Yanbian University, Pharmacy, 2017.

962 Zhou, Shoubing. Construction of a controlled-release targeted drug delivery system based on mesoporous silica and therapeutic study on B-cell lymphoma [D]; Southeast University, Clinical Medicine; Oncology, 2017.

963 Zhou T. Separation and analysis of Angelica sinensis polysaccharides, structural characterization and study of biological activities [D]; Huazhong University of Science and Technology, Pharmacology, 2017.

964 Zhou ZF. Study on the effect of tranexamic acid on perioperative coagulation and postoperative cerebral infarction in cardiac surgery [D]; Zhejiang University, Anesthesiology, 2017.

965 Zou D. Construction of GM1 drug-carrying micelles through the blood-brain barrier and its experimental study of antitumor and neuroprosthetic co-administration [D]; Chongqing University, Pharmacy, 2017.

966 Zou, Yumei. Research on the effect of resistance training combined with aerobic exercise on sleep quality and dialysis-related fatigue in maintenance hemodialysis patients [J]. China Convalescent Medicine, 2017, 26(1): 13-5,6.

967 Bie Xin, Zhang Yimin, Ma Zu, et al. Effects of green tea extract EGCG on oxidative stress and nutritional status in maintenance hemodialysis patients [J]. Chinese Journal of Integrative Nephrology, 2018, 19(1): 28-31.

968 Cai Shulan, Jia Hongguang, Zhang Suying, et al. Effects of aerobic exercise on oxidative stress and survival quality of hemodialysis patients with diabetic nephropathy [J]. Journal of North China University of Science and Technology (Medical Edition), 2018, 20(1): 21-4.

969 CAI Yu-Wei, ZHANG Ji-Ling, JI LING Z. Impact of WeChat platform education on self-management and adherence of maintenance hemodialysis patients [J]. China Rural Health Care Management, 2018, 38(3): 347-8.

970 Cao Xiaohui, Jia Liyan, Hu Yanyun, et al. Effect of hemoperfusion combined with sequential dialysis on soluble tumor necrosis factor receptor in patients with diabetic nephropathy [J]. Journal of Hainan Medical College, 2018, 24(1): 23-5,9.

971 Zeng Lijuan, Zhan Shuhui, Li Yan, et al. Effects of SLED combined with HP on inflammatory stress, hemodynamic parameters and renal function in patients with sepsis combined with severe acute kidney injury [J]. Journal of Hainan Medical College, 2018, 24(2): 169-72.

972 Chen F. Exploring the clinical effects of nursing intervention and dialysis therapy in acute renal failure and multiple organ dysfunction syndrome [J]. Mother and Child World, 2018, (24): 238.

973 CHEN LIU ZHEN, LEI NAYAN, NA YAN L. Effect of collaborative nursing intervention on treatment adherence and quality of life of hemodialysis patients with chronic renal failure [J]. Massage and Rehabilitation Medicine, 2018, 9(17): 51-2.

974 Chen Ting. Effect of soluble dietary fiber on the implementation effect of enteral nutrition in patients with severe acute pancreatitis [D]; Army Military Medical University, Nursing, 2018.

975 Chen Wei, Li Juan, Niu Yakai, et al. Aerobic exercise regulates body weight changes in obese mice on high-fat diet via mesolimbic-striatal dopamine plasticity [J]. Sports Science, 2018, 38(12): 53-61.

976 Chen Xiaotong. Analysis of the protective effect of far-infrared irradiation on autologous internal fistula in hemodialysis patients [J]. Practical Gynecological Endocrinology Electronic Journal, 2018, 5(19): 36,40.

977 CHEN YAN, CHEN JINLU, WANG XIAOGUANG, et al. Effects of acupoint injection of astragalus injection on the quality of survival of MHD patients [J]. Medical Clinical Research, 2018, 35(2): 386-8.

978 Cheng Juan. Construction of novel nanosystems and its application in hemostasis and abdominal aortic aneurysm control [D]; Army Medical University, Pharmacy, 2018.

979 Cheng Y, Chen G, Zhang L, et al. Effect of cognitive behavioral intervention on disease knowledge and self-care ability of hemodialysis patients with hyperphosphatemia [J]. General Practice Nursing, 2018, 16(30): 3718-21.

980 Cui Huifang, Qiang Yanjuan, Xu Zhongxiu, et al. Analysis of the role of aerobic exercise workout on sleep quality and adverse emotions in uremic hemodialysis patients [J]. World Journal of Sleep Medicine, 2018, 5(12): 1473-5.

981 Cui Xuebin, Zou Ting, Tang Xiaotie, et al. Effects of high flux dialysis on relevant indicators of maintenance hemodialysis patients with diabetic nephropathy [J]. Chinese and Foreign Medical Research, 2018, 16(3): 41-3.

982 Dee, J. L., Dee, J. H., Luo, J., et al. Analysis of the effect of nursing intervention of family empowerment model on improving negative emotions and compliance behavior of uremic hemodialysis patients [J]. Internal Medicine, 2018, 13(3): 422-4.

983 DONG TAO, WU QIAN, QIAN W. Comparison of efficacy and safety of vancomycin and linezolid in the treatment of peritoneal dialysis-associated peritonitis due to methicillin-resistant Staphylococcus aureus [J]. Chinese Journal of Clinicians, 2018, 46(10): 1187-9.

984 Dong ZJ. Research on the intervention effect of resistance exercise on maintenance hemodialysis patients with sarcopenia [D]; Nanjing Medical University, Nursing, 2018.

985 DU Wei-Ping, DANG Li-Juan, JIA Yi-Min, et al. Study on the protective effect of tanshinone IIA sulfonic acid sodium on damaged tissues of rats with ischemic stroke [J]. China Pharmaceutical, 2018, 27(11): 12-4.

986 Feng Tingting, Sun E, Wu Xiaoxia, et al. Effects of hemoperfusion combined with Astragalus injection on oxidative stress, neuroendocrine hormones and quality of life in patients with maintenance hemodialysis combined with refractory hypertension [J]. Journal of Modern Integrative Chinese and Western Medicine, 2018, 27(17): 1864-7.

987 Gao Wei, Yang Xinlu, Wei Xin, et al. Ultrasound-guided rectus abdominis sheath nerve block in peritoneal dialysis tube implantation [J]. Journal of Clinical Anesthesiology, 2018, 34(3): 217-21.

988 Gong Manqin, Wang Yujing, Wang Qingqiu, et al. Effect of high flux hemodialysis on microinflammatory status in diabetic nephropathy patients [J]. Journal of Clinical Military Medicine, 2018, 46(8): 943-5.

989 Gu Weiwei. Effects of sulbactam on GLT-1 binding properties, glutamate uptake and glutamate concentration in the CAI region of hippocampus of rats with total cerebral ischemia [D]; Hebei Medical University, Pathology and Pathophysiology, 2018.

990 Guo Cuicui. Effect of nursing intervention on psychological status of uremic hemodialysis patients [J]. Mother and Child World, 2018, (13): 229.

991 Guo Yuqing, Zhao Chunli, Wei Seefang, et al. Family Intervention in Rehabilitation Nursing of Hemodialysis Patients [J]. Biped and Health Care, 2018, 27(10): 33-4.

992 Guo, Z. H., Deng, Y., Shen, K. K., et al. Evaluation of the value of left ventricular diastolic function in patients with uremia of different ages using the PW/TDI technique; proceedings of the Chinese Society of Echomedical Engineering 14th National Echocardiography Symposium, Tianjin, F, 2018 [C].

993 GUO Z H, TIAN J W, JIA WEI T. Quantification of left ventricular diastolic function in patients with CRF by blood flow vector imaging; proceedings of the 2018 Cross-Strait Medical and Healthcare Exchange and Cooperation Conference and the 10th Cross-Strait Ultrasound Medicine High-end Forum, Xiamen, F, 2018 [C].

994 He Zhongyun. Application effect of multidisciplinary nursing model in patients with diabetic nephropathy uremic stage combined with cerebral infarction [J]. Nursing Research, 2018, 32(21): 3406-9.

995 Hei, Yao-Zong. Observation on the clinical effect of hemoperfusion combined with hemodialysis in the treatment of emergency severe organophosphorus pesticide poisoning [J]. Snake Zhi, 2018, 30(4): 699-701.

996 Hu Jianxia. Effect of salt intake on residual renal function and cardiovascular events in patients undergoing abdominal dialysis [D]; Southern Medical University, Internal Medicine (Nephrology), 2018.

997 Huang Xiaoping, Liu Xiong, Lai Fengmei, et al. Bedside continuous static-venous hemodialysis filtration in acute renal failure [J]. Shenzhen Journal of Integrated Chinese and Western Medicine, 2018, 28(1): 128-30.

998 Huang YW. Genotypic analysis of norovirus in children in Fuzhou area and preliminary establishment of group GII norovirus capture ELISA [D]; Fujian Medical University, Epidemiology and Health Statistics, 2018.

999 Jiang Yuan. The value of 5E rehabilitation nursing model in improving self-efficacy of maintenance hemodialysis patients [J]. International Journal of Nursing, 2018, 37(12): 1637-40.

1000 Lao Xiu Juan. Impact of family health literacy management on health behaviors and health literacy of hemodialysis patients [J]. Journal of Chinese Medicine Management, 2018, 26(15): 163-5.

1001 Lai Pu, Wu Wei, Sun Chenbo, et al. Role of hemodialysis filtration combined with ustekin on inflammatory factors in patients with diabetic ketoacidosis [J]. Journal of Emergency and Critical Care in Internal Medicine, 2018, 24(3): 217-9.

1002 LI DONG FENG, WANG DONG MEI, DONG MEI W. Efficacy of hemodialysis combined with hemoperfusion in treating patients with end-stage diabetic nephropathy and the effect on their inflammatory mediators [J]. Modern Diagnosis and Treatment, 2018, 29(12): 1895-7.

1003 Li Jing, Pei Qiangqiang, Gao Xiujuan, et al. Application of intensive self-empowered nursing care in diabetic nephropathy patients undergoing peritoneal dialysis [J]. China Primary Health Care, 2018, 32(5): 80-2.

1004 Li Li-Min, Ge Yuchen, Liu Zhe, et al. Value assessment of San Xian Kidney Replenishing Formula combined with western medicine in treating post-dialysis patients with diabetic renal failure [J]. Hebei Medicine, 2018, 40(22): 3414-7.

1005 Li Qianling, Wu Bijing, Li Guanmei, et al. Analysis of the preventive effect of rational exercise nursing on hypotension in maintenance hemodialysis patients [J]. Nursing Practice and Research, 2018, 15(7): 39-40.

1006 LI Wenjing, SHI Cong, SONG Pei, et al. Effects of different flux dialysis membranes on oxidative stress status and serum inflammatory factors in maintenance hemodialysis patients [J]. China Clinical Research, 2018, 31(4): 486-9,93.

1007 Li Xiaoqing, Guo Min, Lin Ying, et al. Study on the effect of exercise intervention on symptomatic hypotension in dialysis patients [J]. Electronic Journal of Practical Clinical Nursing, 2018, 3(17): 32,5.

1008 Li Yuanyuan, Zhu Haodong, Zhang Li, et al. Effect of exercise team on improving quality of life sleep quality and dialysis adequacy of hemodialysis patients [J]. Journal of Xinjiang Medical University, 2018, 41(10): 1231-4,8.

1009 LIU Bing, LI Guihua, ZHANG Jinwen, et al. Exploring the effect of hematopoietic tablets on erythropoietin treatment of renal anemia in young and middle-aged chronic kidney disease patients with renal failure [J]. International Journal of Urology, 2018, 38(5): 811-4.

1010 Liu Duo Duo. Diagnostic value of yellow fever virus nonstructural protein 1 (YFV-NS1) in the early stage of viral infection and preparation of anti-YFV-NS1 monoclonal antibody [D]; Southern Medical University, Clinical Laboratory Diagnostics, 2018.

1011 Liu Shuanglin, Ma Xiaolin, Luo Kaikai, et al. The use of blood purification in the treatment of intractable heart failure [J]. Electronic Journal of Clinical Medicine Literature, 2018, 5(41): 28.

1012 LIU Sinan, CHEN Lin, LIN C. Effect of integrated management model of healthcare in hemodialysis patients with hyperphosphatemia [J]. China Blood Purification, 2018, 17(8): 573-6.

1013 Liu WJ. Study on the correlation between brain tissue oxygen saturation and its prognosis after congenital heart disease surgery [D]; Chongqing Medical University, Emergency Medicine, 2018.

1014 LIU XI, LIU F, MU H, et al. Effects of oral phosphorus binding agent on calcium and phosphorus metabolism and vascular calcification in elderly maintenance hemodialysis patients [J]. Hebei Medicine, 2018, 40(10): 1509-12.

1015 LIU Xiaochen, WANG Xiuli, LI Na, et al. Effects of motivational interviewing on exercise compliance in continuous ambulatory peritoneal dialysis patients [J]. Qilu Nursing Journal, 2018, 24(23): 59-62.

1016 LIU Xiaoli, CHEN Ping, LIN Xiangming, et al. Exercise Intervention Improves Behavioral Functions in Parkinson's Disease Model Rats by Slowing Striatal Dopamine Loss [J]. Chinese Journal of Sports Medicine, 2018, 37(1): 28-35.

1017 Liu Yingying. Effects of extended care on self-management ability and quality of life of hemodialysis patients with chronic renal failure [J]. Zhongguo Nation Health Medicine, 2018, 30(23): 119-21.

1018 Luo Xianglian, Huang Yalian, Fang Yanchun, et al. The effect of group love and laughter yoga on the quality of life of maintenance hemodialysis patients [J]. Journal of Nursing, 2018, 25(22): 1-5.

1019 Ma Jinlan. Effects of high-throughput hemodialysis on oxidative stress and microinflammatory status in hemodialysis patients [J]. Qinghai Medicine Journal, 2018, 48(9): 15-7.

1020 Mao M, Ren S, Hong D, et al. Meta-analysis of the effects of exercise on inflammatory indexes, maximal oxygen uptake and quality of life in patients with chronic kidney disease [J]. Sichuan Medicine, 2018, 39(6): 634-8.

1021 MI Zhijie, GONG Hongchang, XIA Xiaofei, et al. Clinical significance of continuous renal replacement therapy in patients with acute kidney injury due to infectious shock [J]. Marker Immunoassay and Clinics, 2018, 25(7): 1048-51,56.

1022 Peng Fumei. Impact of refined extended care model on peritoneal dialysis patients [J]. Electronic Journal of Practical Clinical Nursing, 2018, 3(8): 39,47.

1023 Pu Jiang. Meta-analysis of the effects and safety of exercise on MHD patients in hemodialysis [D]; Southwest Medical University, Internal Medicine, 2018.

1024 Qi L, Chen Y, Chen L, et al. Observation on the efficacy of cardiac rehabilitation exercise on patients with maintenance hemodialysis combined with cardiac insufficiency [J]. China Rehabilitation, 2018, 33(4): 297-300.

1025 Ren Guangwei, Li Mingming, Yang Hongjuan, et al. Effect of febuxostat on vascular endothelial function and oxidative stress in patients with maintenance hemodialysis with hyperuricemia and safety evaluation [J]. Hebei Medicine, 2018, 40(15): 2251-5.

1026 Ren Song, Hong Daxue, Zhang Yuan, et al. Meta-analysis of the effect of exercise on cardiovascular function in patients with chronic kidney disease [J]. Chinese Journal of Integrative Nephrology, 2018, 19(9): 793-8.

1027 SHAO Yunman, DANG Ying, JIANG Jiaojiao, et al. Effects of zinc supplementation therapy on oxidative stress status in maintenance hemodialysis patients [J]. Anhui Medicine, 2018, 39(7): 823-6.

1028 SHENG X A, ZHANG X F, XIAO FENG Z. Clinical efficacy of ustekin combined with hemodialysis in the treatment of diabetic ketoacidosis [J]. Medical Clinical Research, 2018, 35(9): 1758-60.

1029 Song Yuchen, Lei Shuang, Han Xinmin, et al. Exploring the effects of Anshen Dingzhi Ling on prefrontal NE, DA and their metabolite contents in SHR rats based on microdialysis technique [J]. Chinese Journal of Traditional Chinese Medicine, 2018, 33(10): 4631-6.

1030 Sun Xiuzhen. Effects of low-intensity aerobic exercise combined with dietary guidance on dialysis-related fatigue in dialysis patients with chronic renal insufficiency [J]. Health Care Guide, 2018, (19): 122.

1031 QIN Jolian, XIE Yongxiang, MENG Lifeng, et al. Effects of different osmolality herbal colonic dialysis on microinflammatory-nutritional status of patients with CKD stage 3-4 [J]. Chinese Journal of Integrative Nephrology, 2018, 19(10): 879-82.

1032 WANG Fang, XIE Minyan, ZHANG Dongsheng, et al. Effects of alfacalcitol shock therapy on calcium and phosphorus metabolism, microinflammation, and oxidative stress status in maintenance hemodialysis patients [J]. Practical Drugs and Clinics, 2018, 21(7): 765-8.

1033 WANG Haibo, LIU Wei, HU Quanqing, et al. Clinical study of early hemoperfusion combined with dialysis for acute organophosphorus poisoning [J]. Chinese and Foreign Medicine, 2018, 37(24): 37-9.

1034 Wang Jun. Improvement effect of high flux dialysis on oxidative stress and inflammatory state in dialysis patients with diabetic nephropathy [J]. China Health Engineering, 2018, 17(5): 789-91.

1035 WANG Min, ZHANG Dewei, DEWEI Z. Clinical observation of calcium hydroxybenzenesulfonate causing falsely low blood creatinine results [J]. China Modern Physician, 2018, 56(5): 79-81.

1036 Wang X, Zhou Lijuan, Bian Yueqiu, et al. Analysis of the effects of brain resistance training combined with aerobic exercise on fatigue status and sleep quality of maintenance hemodialysis patients [J]. Journal of Taizhou Institute of Vocational Technology, 2018, 18(5): 61-3.

1037 Wang Wenting, Wu Chao, Luo Yongqin, et al. Meta-analysis of the effects of aerobic exercise on somatic function and quality of life of hemodialysis patients [J]. Chinese Journal of Modern Nursing, 2018, 24(23): 2797-803.

1038 Wang S-L. Impact of Accountability Health Promotion on Compliance Behavior of Maintenance Hemodialysis Patients [J]. Contemporary Nurses (in Chinese), 2018, 25(12): 106-7.

1039 Wang YAN. Effect of acupoint massage on fatigue state and lower limb motor function of hemodialysis patients with diabetic nephropathy [J]. Biped and Health Care, 2018, 27(5): 149,51.

1040 Wang YS. Analysis of the efficacy of hemodialysis combined with ustekin in the treatment of diabetic ketoacidosis [J]. Journal of Clinical Psychosomatic Diseases, 2018, 24(1): 148-50.

1041 Wang Zhenkui. Comparative study on the clinical efficacy of traditional Chinese medicine in treating chronic kidney disease in stage 5 [D]; Southern Medical University, Clinical Integrative Medicine, 2018.

1042 Wei, S.Y.. Effect of anti-resistance exercise intervention on dialysis adequacy in maintenance hemodialysis patients [J]. Nursing Practice and Research, 2018, 15(10): 31-2.

1043 WEI Xing, CHEN Ziren, ZHAO Fang, et al. Effects of xanthoi capsule on oxidative stress and microinflammatory response state in hemodialysis patients [J]. Jilin Medicine, 2018, 39(9): 1665-7.

1044 WEI Zhenzhong, SONG Xuexia, LIAO Hongxia, et al. Effects of hemodialysis plus hemoperfusion on bone mineral density as well as anti-ox-LDL-Ab levels in patients with end-stage diabetic nephropathy [J]. Journal of Difficult Diseases, 2018, 17(9): 900-4.

1045 WEI Leilei, XUE Pengshi, PENGSHI X U E. Targeted nutritional care for patients on maintenance hemodialysis [J]. Journal of Practical Clinical Medicine, 2018, 22(4): 34-7.

1046 WEI LIFANG, HE ZHAO LING, ZHAO LING H. Effects of levocanidin on MDA, SOD and cardiac function in maintenance hemodialysis patients [J]. Medical Theory and Practice, 2018, 31(6): 839-41.

1047 Xin Xin. Study on Chrysanthemum Cordyceps Nephroprotection and Improvement of Constipation [D]; Henan University, Chinese Medicine Testing and Analysis, 2018.

1048 Xiong Wei, Gao Zhi, Liu Xin, et al. Effects of Cicadas Free Decoction Granules on Oxidative Stress and Inflammatory Factors in Maintenance Hemodialysis Patients [J]. Research on Integrative Chinese and Western Medicine, 2018, 10(5): 233-5.

1049 XU Niobium, ZHENG Guiqiong, LI Xiaomei, et al. Mobile phone application software for home exercise guidance for hemodialysis patients [J]. Journal of Nursing, 2018, 33(11): 97-100.

1050 Xu Yueping, Hu Qian, Zhu Xiaozhen, et al. Effects of resistance training combined with aerobic exercise on sleep quality and fatigue in maintenance hemodialysis patients [J]. Nursing Research, 2018, 32(2): 317-9.

1051 Chunju Xue. Clinical application study of high flux multimodal combined dialysis on cardiovascular disease related indexes in maintenance hemodialysis patients; proceedings of the 2018 Academic Annual Meeting of the Nephrology Committee of Guizhou Society of Integrative Medicine, Guiyang, F, 2018 [C].

1052 Yang KU. Effect of high flux hemodialysis combined with tea polyphenols on oxidative stress and nutritional status of maintenance hemodialysis patients with diabetic nephropathy [J]. China Clinical New Medicine, 2018, 11(4): 359-62.

1053 Yang T. Research on the effect of Orem support-education system theory intervention on self-care ability of maintenance hemodialysis patients [D]; Nanchang University of Medicine, Nursing, 2018.

1054 Yang T, Chen M, Tang S, et al. Effects of Pilates Exercise on Fatigue Status and Sleep Quality of Maintenance Hemodialysis Patients [J]. China Blood Purification, 2018, 17(7): 456-60.

1055 YANG S L, HAN W, WEI H A N. Effect of clinical pathway health education applied to hemodialysis patients in induction phase [J]. Chinese and Foreign Medical Research, 2018, 16(34): 168-9.

1056 YANG Y. Analysis of the impact of comprehensive nursing care on motor ability, living ability and prognosis of patients with epilepsy secondary to stroke [J]. Family Medicine, 2018, (7): 244-5.

1057 Ye Fei. Research on the intervention effect of bicycle exercise on hypotension in maintenance hemodialysis patients on dialysis [D]; HUZHOU NORMAL COLLEGE, AGE NURSING, 2018.

1058 Ye Jingyun. Clinical study of umeboshi spray on thirst in maintenance hemodialysis patients [D]; Guangzhou University of Traditional Chinese Medicine, Nursing, 2018.

1059 Yi LH. Effect of exercise therapy with nursing intervention on nutritional status and quality of life of maintenance hemodialysis patients [J]. Medical Information, 2018, 31(16): 175-7.

1060 Yi Waihong. Application of low-salt diet combined with lifestyle intervention in the care of hypertensive patients on peritoneal dialysis [J]. International Journal of Nursing, 2018, 37(12): 1678-80.

1061 Yin Juan. Study on the incidence of hospital infection and risk factors in a general hospital in Xi'an [D]; Air Force Military Medical University, Disease Prevention and Health Promotion, 2018.

1062 YU JIN RONG, MA HUI, HUI M. Effects of Kidney-boosting and Blood-activating Formula combined with Chlorosartan on Vascular Endothelial Function and Inflammatory Cytokines in Maintenance Hemodialysis Patients [J]. Journal of Modern Integrative Chinese and Western Medicine, 2018, 27(19): 2133-6.

1063 YU JIN RONG, MA HUI, HUI M. Effects of kidney-boosting and blood-activating formula combined with chlorthalidomide on vascular endothelial function and inflammatory cytokines in patients on maintenance hemodialysis [J]. Journal of Modern Integrative Chinese and Western Medicine, 2018, 27(16): 1757-9.

1064 Yu Xiaolei, Liu Jing, Mao Qunyan, et al. Effects of ultra-early acupuncture at Shuigou acupoint on amino acid neurotransmitters in striatum of rats with acute cerebral ischemia [J]. Shanghai Journal of Acupuncture and Moxibustion, 2018, 37(8): 951-6.

1065 Zhang. Amassing and preparation of shark-derived single domain antibody to polyphenol oxidase [D]; Ocean University of China, Food Processing and Safety, 2018.

1066 Zhang Baotong, Liu Yunxia, Guo Dongci, et al. Effect of melatonin on oxidative stress and inflammatory response in maintenance hemodialysis patients with chronic renal failure [J]. Journal of Difficult Diseases, 2018, 17(4): 374-7.

1067 ZHANG Chunyan, LIU Ying, YING L I U. Application of PDCA cycle nursing intervention in reducing short-term withdrawal rate of peritoneal dialysis patients [J]. Nursing Practice and Research, 2018, 15(20): 54-5.

1068 Zhang Hazhen, Liu Peng, Wang Junlu, et al. Application effect of leucovorin in the treatment of renal anemia [J]. Practical Clinical Medicine, 2018, 19(12): 1-4.

1069 Zhang Juanjuan, Wang Qijing, Chen Lili, et al. Value analysis of continuous blood purification application in infectious shock patients with renal dysfunction [J]. Medical Theory and Practice, 2018, 31(23): 3530-1.

1070 Zhang Qi-Xiong. Synthesis of reactive oxygen-responsive materials and their drug-carrying systems in the prevention and treatment of inflammatory diseases [D]; Army Military Medical University, Pharmacy, 2018.

1071 ZHANG XUPING, ZHENG JUNLIAN, JUN LIANG Z. Effects of targeted nursing care on self-care behaviors of peritoneal dialysis in elderly patients with diabetic nephropathy [J]. Family Medicine, 2018, (2): 215.

1072 ZHANG Ai-Ping. Evaluation of the application effect of extended care on maintenance hemodialysis patients [J]. Chinese and Foreign Medicine Research, 2018, 16(6): 83-5.

1073 ZHAO Guang-Ying, LIU Hui-Lin, HUI Lin L. Effects of corrective education model on calcium and phosphorus metabolism in hemodialysis patients with end-stage renal disease [J]. General Practice Nursing, 2018, 16(19): 2418-20.

1074 Zhao Hongbin. Analysis of factors affecting osteoporotic fracture in postmenopausal female non-dialysis chronic kidney disease patients [J]. Journal of Changzhi Medical College, 2018, 32(3): 198-200.

1075 Zhao Yanyan. Effects of reduced glutathione on TLRs/MYD88 signaling pathway and microinflammatory status in hemodialysis patients [J]. Chinese Journal of Health Inspection, 2018, 28(11): 1350-2.

1076 ZHAO Ying, YANG Xiaojuan, WANG Chunling, et al. Effects of health education pathway management on disease uncertainty and medical compliance behavior of uremic patients [J]. Journal of Practical Diabetes, 2018, 14(6): 58.

1077 Zheng Lijuan. The role of continuous hemodialysis in the treatment of diabetic nephropathy ketoacidosis combined with acute kidney injury [J]. Dietary Health Care, 2018, 5(12): 65-6.

1078 ZHENG Suping, NING Qian, YANG Huili, et al. Effects of individualized exercise combined with targeted nursing intervention on nutritional status and quality of life of uremic hemodialysis patients [J]. Henan Medical Research, 2018, 27(17): 3252-3.

1079 Zou JH, Bian BH, Hu DK, et al. Effect of individualized exercise intervention on improving quality of life of elderly maintenance hemodialysis patients [J]. Practical Geriatrics, 2018, 32(7): 649-52.

1080 Zou Yufang. Evaluation of the effect of quality nursing care for hypertensive nephropathy patients receiving hemodialysis [J]. Contemporary Medicine Series, 2018, 16(17): 243-5.

1081 A Xian Uzhina. Study related to the effects of tai chi exercise on blood pressure, heart rate, physical changes and IGF-1 in hemodialysis patients [D]; Xinjiang Medical University, Internal Medicine (Nephrology), 2019.

1082 A Xian Uzhina, Zhang Li, Li Yufang, et al. Study on the effects of tai chi exercise on blood pressure, heart rate and physical changes in hemodialysis patients [J]. Chinese Journal of Integrative Nephrology, 2019, 20(9): 776-80.

1083 Bo Ruonan. Study on the immune-enhancing effect of liposomes of Lycium barbarum polysaccharide and its mechanism [D]; Nanjing Agricultural University, Veterinary Medicine; Clinical Veterinary Medicine, 2019.

1084 Chen Cheng. Analysis of the efficacy of leucovorin in improving cardiac function in patients with chronic renal failure and hemodialysis heart failure [J]. Chinese and Foreign Medicine, 2019, 38(14): 109-11.

1085 Chen Xianyu. Study on the intervention effect of dandelion polysaccharide on acute kidney injury in rats [D]; Hebei North College, Internal Medicine, 2019.

1086 Chen YF, Dong YZ, Zhou ML, et al. Effects of exercise-music therapy on general well-being and coping styles of maintenance hemodialysis patients [J]. Zhejiang Medical Education, 2019, 18(5): 32-4,8.

1087 Chen YP. Efficacy of noninvasive ventilation combined with hemodialysis in the treatment of uremia with acute left heart failure [J]. Chinese Medical Science, 2019, 9(17): 208-10.

1088 CUI Lianshun, YU Meiyan, YU M. Effect of levofloxacin combined with heparin on serum PCT and CRP levels in patients with hemodialysis catheter infection [J]. International Medical and Health Herald, 2019, 25(18): 3114-7.

1089 Cui Weihua, Liu Weige, Liu Changrong, et al. Effect of nursing intervention based on human fitness transfer theory in hemodialysis patients [J]. Clinical Medicine Research and Practice, 2019, 4(2): 177-8,95.

1090 Deng Huiwen. Clinical Observation of Renyuan Granules in the Treatment of Chronic Kidney Disease in Stages 3-5 [D]; Hubei University of Traditional Chinese Medicine, Traditional Chinese Medicine and Internal Medicine, 2019.

1091 Deng Jiangtao, Zhang Jingjing, Shen Huiqin, et al. Effects of pyruvate intraperitoneal resuscitation on JAK/STAT signaling pathway in intestinal tissues of rats with hemorrhagic shock [J]. Chinese Journal of Anesthesiology, 2019, 39(7): 866-9.

1092 Ding SJ. Analysis of nursing + exercise therapy in hemodialysis [J]. Chinese Continuing Medical Education, 2019, 11(18): 141-3.

1093 Dong Q. Analysis of clinical efficacy of hemodialysis and small-dose hormone combined treatment of uremia [J]. Healthy Friends, 2019, (9): 54.

1094 Du Liping. Research on the application of aerobic exercise program in peritoneal dialysis patients [D]; Zhengzhou University, Nursing, 2019.

1095 DU Meilian, QU Xiaolu, QI Yinghui, et al. Effects of astragalus injection on oxidative stress in hemodialysis patients after intravenous iron therapy [J]. Chinese Journal of Integrative Nephrology, 2019, 20(7): 590-2.

1096 Fan Binfu. Analysis and research on treatment and clinical prognosis of acute poisonous mushroom poisoning [J]. Chinese and Foreign Medicine, 2019, 38(32): 59-61.

1097 Fu Li-Hua. Clinical application of CRRT in patients with acute kidney injury due to crush syndrome; proceedings of the Chinese Physicians Association Nephrologists Branch 2019 Academic Annual Meeting (CNA2019), Boao, Hainan, F, 2019 [C].

1098 GAO SI YAO, LU WAN GANG, WAN GANG L. Meta-analysis of the effectiveness of aerobic exercise in improving the quality of survival of maintenance hemodialysis patients [J]. Chinese Journal of Rehabilitation Medicine, 2019, 34(4): 453-9.

1099 GUAN JUN RONG, LAI SHAO YAN, LAI S. Effects of rehabilitation exercise training on sleep quality and fatigue level of uremic hemodialysis patients [J]. Chinese Journal of Practical Nursing, 2019, 35(26): 2012-6.

1100 Guo H. Methods and effects of aerobic exercise on maintenance hemodialysis patients [J]. Health Care Guide, 2019, (47): 261.

1101 Han Mei. Evaluation of the effect of supportive psychotherapy combined with aerobic exercise applied to maintenance hemodialysis patients [J]. Medical Clinical Research, 2019, 36(11): 2193-5.

1102 HAN Pengxun. Experimental study of TCM somatic evidence and metabolic characteristics of type 2 diabetic nephropathy and artemether intervention [D]; Guangzhou University of Traditional Chinese Medicine, Clinical of Integrative Medicine and Western Medicine, 2019.

1103 Hao DY. Application of resistance training combined with aerobic exercise in hemodialysis patients [J]. Nursing Practice and Research, 2019, 16(21): 66-7.

1104 HE JIAN-ZHONG, CHEN GUANG-QUAN, GUANG-QUAN C. Effects of high-throughput hemodialysis on toxin clearance and levels of inflammatory factors and oxidative stress factors in patients with type 2 diabetic nephropathy [J]. Marker Immunoassay and Clinics, 2019, 26(4): 571-5.

1105 HE Tonglin, MAI HaoTian, LIU JunHua, et al. Observation of the application effect of exercise rehabilitation therapy on patients with uremic sarcopenia [J]. China Practical Medicine, 2019, 14(9): 188-90.

1106 HU Xiaoyan, YIN Lixia, ZHANG Hailin, et al. Effects of multidisciplinary cooperative exercise therapy on quality of life and microinflammatory status of maintenance hemodialysis patients [J]. China Nursing Management, 2019, 19(10): 1467-71.

1107 HUANG Chunxia, LIN Qiuhua, NG Jinquan, et al. Effects of aerobic exercise in dialysis on sleep quality and quality of life of hemodialysis patients [J]. Nursing Practice and Research, 2019, 16(3): 35-6.

1108 Huang Daoda. Efficacy of iron sucrose combined with recombinant human erythropoietin in the treatment of hemodialysis patients with combined renal anemia [J]. Medical Equipment, 2019, 32(22): 74-5.

1109 Huang Haiyan. Effect of Picrasidine on Lipopolysaccharide-Induced Peritoneal Fibrosis in Uremic Rats via TGF--β1/Samd3 [D]; Guangxi Medical University, Nephrology, 2019.

1110 HUANG Li, WANG Yongjian, YONG JIAN W. Observation on clinical efficacy of different blood purification modalities on sepsis-induced acute kidney injury [J]. Chinese Medical Journal, 2019, 54(7): 798-800.

1111 HUANG Lijuan, DUAN Peibei, ZHANG Jing, et al. Effects of exercise guidance on quality of life of hemodialysis patients based on trans-theoretical modeling [J]. China Blood Purification, 2019, 18(3): 181-4.

1112 Huang Xiuli. Clinical study of abdominal wall nerve block combined with remifentanil for patients undergoing peritoneal dialysis placement for combined cardiovascular diseases [D]; Chinese Academy of Medical Sciences, Peking Union Medical College, Anesthesiology, 2019.

1113 Huang Yanlin. Application of 5E rehabilitation model based on WeChat platform in hemodialysis patients with CKD-MBD; proceedings of the Chinese Physicians Association Nephrologists Branch 2019 Academic Annual Meeting (CNA2019), Boao, Hainan, F, 2019 [C].

1114 Huang Yuanyuan. Endurance exercise in hemodialysis improves patients' hypotensive state; proceedings of the Chinese Physicians Association Nephrology Physicians Branch Annual Academic Conference 2019 (CNA2019), Boao, Hainan, F, 2019 [C].

1115 Jiao Lijuan, Tohuti Pazhendanmu, Li Yufang, et al. Analysis of the Effects of Aerobic Exercise Combined with Levocanidin on Inflammatory State, Oxidative Stress, and Nutritional Status of Maintenance Hemodialysis Patients [J]. Chinese Journal of Frontiers of Medicine (Electronic Edition), 2019, 11(11): 90-3.

1116 Xie Yuhuai. Immunomodulatory effects of alfalfa polysaccharides and their mechanisms [D]; Shandong Agricultural University, Animal Nutrition Physiology, 2019.

1117 Lan Xinxin. Clinical study of quality nursing service to reduce peritonitis in home peritoneal dialysis patients; proceedings of the Chinese Physicians Association Nephrologists Branch 2019 Academic Annual Meeting (CNA2019), Boao, Hainan, F, 2019 [C].

1118 Lan Xinxin. Clinical study of quality nursing service to reduce negative emotions of home peritoneal dialysis patients; proceedings of the Chinese Physicians Association Nephrology Physicians Branch Annual Academic Conference 2019 (CNA2019), Boao, Hainan, F, 2019 [C].

1119 LANG Haiyan H, HE Hanqin H, HAN QIN H. Impact of health education based on the trans-theoretical model on compliance behavior and self-care ability of uremic hemodialysis patients [J]. Medical Clinical Research, 2019, 36(4): 819-21.

1120 Li Bo. A study of individualized exercise combined with nursing intervention on nutritional status and quality of life of uremic hemodialysis patients [J]. Health | Health Must Read, 2019, (34): 151.

1121 Li J. Impact of hemodialysis and peritoneal dialysis on the prognosis of elderly patients with end-stage renal disease and the analysis of their influencing factors; proceedings of the Chinese Medical Doctors Association Nephrologists Branch 2019 Academic Annual Meeting (CNA2019), Boao, Hainan, F, 2019 [C].

1122 Li J. Application of continuous blood purification in infectious shock with renal dysfunction [J]. Journal of Shanxi Staff Medical College, 2019, 29(4): 30-2.

1123 Li Ruihua, Chen Huiling, Wu Xueyu, et al. Effects of pedaling training on laboratory objective indexes in uremic hemodialysis patients [J]. Journal of Guangzhou Medical University, 2019, 47(2): 116-8.

1124 Li Ruixue. Cardioprotective effects of leucovorin in maintenance hemodialysis patients [D]; Shandong First Medical University, Internal Medicine, 2019.

1125 Li Xingmei. Impact of nursing interventions on quality of life of maintenance hemodialysis patients; proceedings of the Chinese Physicians Association Nephrology Physicians Branch 2019 Academic Annual Meeting (CNA2019), Boao, Hainan, F, 2019 [C].

1126 Li Zhemei. Research on the effect of health management on calcium and phosphorus metabolism in maintenance hemodialysis patients based on trans-theoretical modeling [J]. Contemporary Nurses (Upper Ten Journal), 2019, 26(5): 130-2.

1127 Liang Yanping, Zheng Juanlian, Zhang Yingjun, et al. Effects of aerobic-resistance exercise intervention on somatic function and oxidative stress in maintenance hemodialysis patients [J]. General Practice Nursing, 2019, 17(27): 3358-61.

1128 Liang Yu. Analysis of the effect of applying levocarnitine to improve nutritional status in uremic hemodialysis patients [J]. China Practical Medicine, 2019, 14(33): 116-7.

1129 LIAO XIAO ZHU, CHEN TONG, CHEN HAI, et al. Effects of renkang injection on cardiac function in patients undergoing maintenance hemodialysis [J]. Chinese Journal of Gerontology, 2019, 39(16): 4002-5.

1130 LIU J, MIAO X, ZHU ZHAOMING, ET AL. Effects of Senqi Qingtu Capsules on Oxidative Stress, Inflammatory Factors and Residual Renal Function in Hemodialysis Patients [J]. China Hospital Drug Evaluation and Analysis, 2019, 19(9): 1079-82.

1131 Liu Meiting. Effect of group management model on adherence of peritoneal dialysis patients; proceedings of the Chinese Physicians Association Nephrologists Branch 2019 Academic Annual Meeting (CNA2019), Boao, Hainan, F, 2019 [C].

1132 Liu XH. Study on prophylactic antibiotics and anesthetic aspects of peritoneal dialysis placement [D]; Shandong University, Internal Medicine (Nephrology), 2019.

1133 Liu, Dialysis. Effects of aerobic exercise workout on sleep quality and adverse emotions in uremic hemodialysis patients [J]. World Journal of Sleep Medicine, 2019, 6(8): 1039-40.

1134 Liu SJ. A study on the effect of motivational interviewing on the self-management level of maintenance hemodialysis patients [D]; Chengde Medical College, Nursing, 2019.

1135 Lu H-T. Study on the Role and Mechanism of Molecular Hydrogen in Ameliorating Peritoneal Fibrosis Associated with Peritoneal Dialysis via PTEN [D]; Chinese People's Liberation Army, Naval Military Medical University; Naval Military Medical University, Specialty Medicine Naval Aviation Medicine, 2019.

1136 LU Xin, YU Weimin, WEI Min Y. Clinical effect analysis of implementing maintenance hemodialysis treatment for patients with diabetic nephropathy [J]. Modern Medicine and Health Research (Electronic Edition), 2019, 3(18): 87-9.

1137 LUO Ji-Cong, LIANG Li, LI L. Design and implementation of peritoneal dialysis patient training model based on VARK learning style [J]. China Blood Purification, 2019, 18(2): 137-41.

1138 Lo, Torch Hui. Evaluation of the efficacy of continuous blood purification for heart failure combined with acute kidney injury in primary hospitals [J]. Journal of Electrocardiography (Electronic Edition), 2019, 8(3): 57-9.

1139 LUO Yi, LI Ba Min, BA MIN L. Observation on the effect of levocanidin combined with iron sucrose on patients with renal anemia on maintenance hemodialysis [J]. China Modern Drug Application, 2019, 13(13): 109-10.

1140 Ma Hui. Effect of nursing intervention on sleep quality and quality of life of patients with sleep disorders occurring on maintenance hemodialysis; proceedings of the Chinese Physicians Association Nephrologists Branch 2019 Academic Annual Meeting (CNA2019), Boao, Hainan, F, 2019 [C].

1141 Ma Hui. Impact of wechat group health education on self-care ability and quality of life of hemodialysis patients; proceedings of the Chinese Physicians Association Nephrologists Branch 2019 Academic Annual Meeting (CNA2019), Boao, Hainan, F, 2019 [C].

1142 Ma Zhaodi. Effects of motivational interviewing based on trans-theoretical modeling on glycemic control and volume load in dialysis patients with diabetic nephropathy [J]. Practical Chinese and Western Medicine Clinic, 2019, 19(5): 160-1.

1143 Mai, Cui-fang. Study on the effect of calcium and phosphorus metabolism in maintenance hemodialysis patients based on trans-theoretical modeling [J]. General Practice Nursing, 2019, 17(15): 1860-3.

1144 Meilan, Liu Huixi, Zhang Wanjun, et al. Intervention of tea polyphenols on lipid peroxidation damage in end-stage renal disease patients on maintenance hemodialysis [J]. China Medical Guide, 2019, 17(28): 5-6.

1145 MENG Xiangzhong, WEI Qiong, ZHU Yu, et al. Continuous renal replacement therapy for the treatment of multiple organ failure in critically ill patients [J]. China Clinical Research, 2019, 32(6): 759-62.

1146 QIN Yajing, ZHANG Yufeng, PING Jinchao, et al. Effects of leucovorin combined with ultrapure dialysis on serum inflammatory factors, cardiac function, and vascular endothelial function in maintenance hemodialysis patients [J]. Clinical Misdiagnosis and Mistreatment, 2019, 32(6): 18-22.

1147 SUN Tianyu, BAN Yuan, ZHANG Peng, et al. Clinical observation of hemodialyzer combined with small-dose hormone treatment of uremia [J]. China Medical Device Info, 2019, 25(19): 134-5.

1148 Qin Chunmei, Li Gang, Zhao Yan, et al. Efficacy analysis of α-lipoic acid combined with methylcobalamin in the treatment of uremic peripheral neuropathy hemodialysis patients [J]. Laboratory Medicine and Clinics, 2019, 16(10): 1408-10.

1149 Tang Caixia. Research on the influence of intellectual promotion on self-management of maintenance hemodialysis patients [D]; Nanhua University, Nursing, 2019.

1150 Tang Xueying, Ning Yangshan, Tian Gangyan, et al. Observation on the effect of levocanidin in the treatment of chronic renal failure combined with arrhythmia [J]. Contemporary Medicine Series, 2019, 17(21): 136-8.

1151 TANG Xiaotie, XU Jie, WANG Liyuan, et al. Effects of atorvastatin combined with irbesartan on oxidative stress and microinflammatory status in elderly peritoneal dialysis patients [J]. Advances in Modern Biomedicine, 2019, 19(20): 3908-11,19.

1152 Wan Guoyun. Study on the synergistic effect of nano-erythrocyte therapy system combined with photothermal/photodynamic and chemotherapy on breast cancer [D]; Tianjin Medical University, Pharmacy, 2019.

1153 Wang T. Analysis of the impact of video tutorials on hemodialysis patients' adherence; proceedings of the Chinese Physicians Association Nephrologists Branch 2019 Academic Annual Meeting (CNA2019), Boao, Hainan, F, 2019 [C].

1154 Wang Aihui. Research on the application of personalized care in the care of arteriovenous endovascular fistula in maintenance hemodialysis patients [J]. Medical Food Therapy and Health, 2019, (15): 162,4.

1155 WANG Daojun, WU Ji, ZHANG Wen, et al. Effect of CRRT treatment on the effect and survival rate of patients with acute kidney injury due to infectious shock [J]. Journal of Baotou Medical College, 2019, 35(12): 33-5.

1156 Wang Chang. Application of quality nursing service in the care of arteriovenous endovascular fistula in hemodialysis patients; proceedings of the Chinese Physicians Association Nephrologists Branch 2019 Academic Annual Meeting (CNA2019), Boao, Hainan, F, 2019 [C].

1157 Wang, H. Y., Cai, S. Q., Xia, J. M., et al. Effectiveness of problem-oriented nursing model in preventing hypoglycemia in hemodialysis in patients with diabetic nephropathy [J]. Journal of Clinical Nursing, 2019, 18(3): 37-40.

1158 Wang Hongying. Evaluation of the effect of intensive health education on maintenance hemodialysis patients; proceedings of the Chinese Physicians Association Nephrologists Branch 2019 Academic Annual Meeting (CNA2019), Boao, Hainan, F, 2019 [C].

1159 WANG JAYING, CHEN XUEFANG, XUEFANG C. Effects of low-intensity aerobic exercise on dialysis effect and fatigue status of hemodialysis patients [J]. Big Doctor, 2019, 4(13): 1-2,7.

1160 WANG Lin, WANG Hong, HONG W. Research on the application effect of exercise management pyramid picture in hemodialysis patients [J]. Chinese Medical Science, 2019, 9(15): 199-201.

1161 WANG L. Effects of targeted nursing intervention combined with individualized exercise on nutritional status and quality of life of uremic hemodialysis patients [J]. Capital Food & Medicine, 2019, 26(20): 91.

1162 Wang P, Wang XC, Liu J, et al. Clinical observation of applying the concept of “Knowing - Believing - Acting” to improve the quality of life of patients with secondary hyperparathyroidism [J]. Jilin Medical Science, 2019, 40(2): 423-5.

1163 WANG XI, ZHOU Lijuan, LI JUAN Z. Effect of planned exercise intervention on hemodialysis patients [J]. Contemporary Nurses (Zhongdian), 2019, 26(6): 103-5.

1164 WANG Shusen. Study on the correlation between improvement of acute kidney injury and mitochondrial autophagy by chuanxiongzine [D]; Hebei North College, Clinical Pharmacy, 2019.

1165 Wang WT, Wu CH, Shen MF, et al. Meta-analysis of the effects of aerobic exercise on dialysis adequacy, microinflammatory status and albumin level in patients on dialysis [J]. Zhejiang Medicine, 2019, 41(3): 255-9,85.

1166 WANG Xiaojing, ZHANG Kun, GE Yan, et al. Effects of exercise on inflammation, oxidative stress and endothelial function in maintenance hemodialysis patients [J]. China Blood Purification, 2019, 18(6): 390-3.

1167 WANG YAHHUI, WU YANQI, SHEN FENG, et al. Hemoperfusion combined with continuous venous-venous hemofiltration for rescue treatment of ? Meta-analysis of clinical efficacy of hemoperfusion combined with continuous veno-venous hemofiltration in paraquat poisoning [J]. Chinese Emergency Medicine for Critical Illness, 2019, 31(2): 214-20.

1168 Wang YAN. Study on the effect of resistance exercise on fatigue in hemodialysis patients during dialysis [D]; Chinese Academy of Medical Sciences, Peking Union Medical College, Nursing, 2019.

1169 Wang Yanping, Cai Yuwei, Mao Weijun, et al. Study on the Effectiveness of Exercise Management Pyramid Picture in Hemodialysis Patients [J]. Chinese Journal of Preventive Medicine, 2019, 20(8): 763-5.

1170 Wei Chunchen, Wei Jingpeng, Lai Jinqiu, et al. Study on the effect of total saponins of Panax quinquefolium on the level of oxidative stress in a rat model of peritoneal fibrosis [J]. Snake Chi, 2019, 31(2): 193-6.

1171 WEN Ganjun, LIU Hong, CHEN Jian, et al. Effects of warming on pathological morphology and nociceptive inflammatory mediators in rats with myofascial pain trigger point model [J]. China Bone Injury, 2019, 32(3): 260-4.

1172 Wu, Huimin. Effects of intravenous iron sucrose supplementation and oral iron dextrose supplementation on anemia and oxidative stress in maintenance hemodialysis patients [D]; Hebei Medical University, Internal Medicine, 2019.

1173 Wu, Li-Ying. Evaluation of the application effect of high flux hemodialysis in the treatment of patients with chronic renal failure [J]. Diet and Health Care, 2019, 6(29): 21-2.

1174 Wu Shenglu. Screening of novel ferulic acid esterase and its enzymatic properties based on macro gene technology [D]; Nanjing Agricultural University, Food Science and Engineering, 2019.

1175 WU Zheqian, DAI Lihua, LIHUA D A I. Efficacy of blood purification combined with ustekin in the treatment of septic acute lung injury and the effect on matrix metalloproteinase levels [J]. Anhui Medicine, 2019, 23(12): 2382-5.

1176 XI Wen, LUO Junxiu, LIU Li, et al. Effects of short video APP on anxiety and depression in young patients on maintenance hemodialysis [J]. Journal of Qiqihar Medical College, 2019, 40(19): 2506-7.

1177 Xiao Y, Liu J, Xie Lizhen, et al. Effect of exercise therapy on the occurrence of hypotension in hemodialysis patients [J]. Chinese Contemporary Medicine, 2019, 26(11): 48-50.

1178 Xie Caiyun, Yan Jing, Zou De'e, et al. Study on the effect of aerobic exercise on toxin clearance in maintenance hemodialysis patients [J]. China Modern Drug Application, 2019, 13(23): 51-2.

1179 Xie YP. Analysis of the application of clinical pathway in hemodialysis; proceedings of the Chinese Physicians Association Nephrologists Branch 2019 Academic Annual Meeting (CNA2019), Boao, Hainan, F, 2019 [C].

1180 Xiong Tingwang. Effects of total alkaloids of Dendrobium officinale on chronic stress-induced anxiety model rats [D]; Zunyi Medical University, Pharmacology, 2019.

1181 Xu Z. Effects of SIRT1/FOXO1-mediated autophagy on diabetic nephropathy and the mechanism of metformin action [D]; Shandong University, Internal Medicine (Endocrine and Metabolic Diseases), 2019.

1182 XU Shuhua, GAO Qianqian, QIAN QIAN G. Effects of Musk Cardioprotection Pill on Cardiac Function in Patients with Chronic Heart Failure Combined with Maintenance Hemodialysis [J]. Dialysis and Artificial Organs, 2019, 30(4): 85-7.

1183 Xu WENN, WENG JINGJING, JIANG LINGYAN, et al. Improvement effect of self-management education on self-management behavior of maintenance hemodialysis patients [J]. Chinese Primary Medicine, 2019, 26(12): 1506-9.

1184 Xu Xuefang, Wu Jingyue, Cui Lina, et al. Effects of self-efficacy intervention on compliance behavior and quality of life of maintenance hemodialysis patients [J]. Nursing Practice and Research, 2019, 16(19): 58-60.

1185 XU PENGGE, SHI XIAO NA, XIAO NA S. Effect of acupressure on lower limb motor function of hemodialysis patients with diabetic nephropathy and evaluation of nursing effect [J]. Biped and Health Care, 2019, (17): 37-8.

1186 Yan Biyan, Mai CuiFang, Yang Weihong, et al. Effects of target-intensity aerobic exercise workout on nutritional status of peritoneal dialysis patients [J]. China Clinical Nursing, 2019, 11(5): 411-5.

1187 Yang Chunping. Effects of extended care on maintenance hemodialysis patients [J]. China National Health Medicine, 2019, 31(8): 161-3.

1188 Yang N. Effects of self-proposed Astragalus and Epiphyllum formula on treatment effect and quality of life of CRF maintenance hemodialysis patients [J]. Chinese Practical Medicine, 2019, 14(29): 20-2.

1189 Yang Qiuxuan. [6]-Gingerol self-microemulsion drug delivery system and its anti-hyperuricemia research [D]; Jiangsu University, Pharmacy, 2019.

1190 Yang X. Effects of peer education on hemodialysis patients [J]. Health Care Guide, 2019, (1): 81.

1191 YANG Xiaodan, ZHAO Changhai, NIE Mingming, et al. Observation on the near- and long-term effects of high-flux hemodialysis for diabetic nephropathy [J]. Zhongguo Nankang Med, 2019, 31(16): 57-9.

1192 Yang Xue. Effects of exercise on symptomatic hypotension and dialysis adequacy during dialysis [J]. Healthcare, 2019, (32): 230-1.

1193 Yang YQ. Role of organic anion transporters (Oats) in adefovir renal interstitial dynamics and its induced renal injury [D]; Lanzhou University, Pharmacy, 2019.

1194 Yi Chenchen. Research on the application effect of aerobic exercise on the debilitation of maintenance hemodialysis patients [J]. Contemporary Nurses (Lower Decade), 2019, 26(9): 120-2.

1195 Yu Xiongwei, Zhang Wei, Chen Qiangyue, et al. Correlation between rehabilitation exercise and blood pressure regulation in peritoneal dialysis patients [J]. Health Research, 2019, 39(5): 510-3.

1196 Yu Wenqiao. Clinical effects and mechanism of glucocorticoids on renal injury caused by severe acute pancreatitis [D]; Zhejiang University, Surgery (General), 2019.

1197 Yuan Mei. Effects of quantitative physical exercise on quality of life and disease regression in maintenance hemodialysis patients [J]. China Rehabilitation, 2019, 34(6): 307-10.

1198 Zang Hongmei. Protective effect of gibberellin saponin XLIX on acute kidney injury and in vivo pharmacokinetic study [D]; Anhui Medical University, Pharmacy, 2019.

1199 Zang Li, Wang Shaoqing, Mao Nan, et al. Effects of different walking numbers on inflammation and nutrition in CKD patients [J]. Journal of Sichuan University (Medical Edition), 2019, 50(2): 252-5.

1200 Zhang Lijun. Exercise therapy improves eGFR, reduces blood pressure and body mass index in nondialysis chronic kidney disease patients; proceedings of the Chinese Physicians Association Nephrologists Branch 2019 Academic Annual Meeting (CNA2019), Boao, Hainan, F, 2019 [C].

1201 Zhang, Lingling. Research on the application of health promotion model in peritoneal dialysis patients [D]; Chengde Medical College, Nursing, 2019.

1202 Zhang V. Intervention Study of Rehabilitation Exercise on Patients with Laparotomy in Chronic Kidney Disease [D]; Ningbo University, Internal Medicine, 2019.

1203 Zhang Xiaoyan. Effects of individualized exercise combined with targeted nursing intervention on nutritional status and quality of life of uremic hemodialysis patients [J]. Chinese and Foreign Women's Health Research, 2019, (10): 19-20.

1204 Zhang Xiaobo, Xu Yunfeng, Chen Fenglan, et al. Clinical Analysis of Staged Blood Purification Combined with Astragalus in the Treatment of Acute Paraquat Poisoning [J]. Hebei Medicine, 2019, 41(22): 3455-7,61.

1205 Zhang Xue. Analysis of nursing measures to improve the quality of life of hemodialysis patients with diabetic nephropathy [J]. Health Care Guide, 2019, (29): 103.

1206 Zhang Yiping, Han Yarong, Liu Yunxia, et al. Effect of hemodialysis combined with hemoperfusion in the treatment of uremic encephalopathy and its effect on patients' serum brain-derived neurotrophic factor and neuropeptide Y [J]. Shaanxi Medical Journal, 2019, 48(8): 1028-31.

1207 Zhang Yue. The effects of different intensities and frequencies of transmedial recumbent gymnastics on endovascularization and quality of life in maintenance hemodialysis patients; proceedings of the Chinese Physicians Association Nephrologists Branch 2019 Academic Annual Meeting (CNA2019), Boao, Hainan, F, 2019 [C].

1208 Zhao Yindan. Soluble α-Klotho protein protects against adenine-mediated sciatic nerve injury in uremic rats [D]; Chinese People's Liberation Army Naval Medical University; Naval Medical University, Internal Medicine (Nephrology), 2019.

1209 Zhong Lijuan. Effects of regular aerobic exercise on body mass control and quality of life of maintenance hemodialysis patients [J]. Nursing Practice and Research, 2019, 16(23): 75-7.

1210 Zhong Yu. Effect of rational exercise therapy on hemodialysis adequacy; proceedings of the Chinese Physicians Association Nephrologists Branch 2019 Academic Annual Meeting (CNA2019), Boao, Hainan, F, 2019 [C].

1211 Zhou Le. Clinical observation of Chinese medicine integrated therapy to improve sleep quality and quality of life in elderly patients with maintenance hemodialysis; proceedings of the Chinese Physicians Association Nephrologists Branch 2019 Academic Annual Meeting (CNA2019), Boao, Hainan, F, 2019 [C].

1212 Zhou M. Application of individualized care based on evidence-based concepts in hypertensive patients on maintenance hemodialysis [J]. China Medical Innovation, 2019, 16(33): 103-7.

1213 Zhu, Li-Wen. Expression purification of recombinant human Asprosin and its protective effect on myocardial ischemia-reperfusion injury in mice [D]; Shaanxi University of Traditional Chinese Medicine, Internal Medicine, 2019.

1214 Bie Xin, Zhang Yimin, Ma Zu, et al. Effect of anti-oxidative stress treatment with green tea extract on renal anemia in hemodialysis patients [J]. Chinese Journal of Nephrology, 2020, 21(7): 585-8.

1215 Cai, M.Y., Gong, W.J., Li, Xue, et al. Effectiveness of Behavioral Change Theory Model in Improving Clinical Symptoms and Quality of Life of Peritoneal Dialysis Patients [J]. China Blood Purification, 2020, 19(5): 305-9.

1216 Cao Cong, He Wenting, Lan Hongjuan, et al. Therapeutic effect of leucovorin combined with erythropoietin in hemodialysis with renal anemia [J]. China Modern Drug Application, 2020, 14(7): 152-4.

1217 Chang Pingping, He Kai, Li Heng, et al. Effects of resistance training combined with aerobic exercise on fatigue status and quality of life of maintenance hemodialysis patients [J]. Medical Theory and Practice, 2020, 33(9): 1530-2.

1218 Shen F, Wang WT, Shen MF, et al. Meta-analysis of the effects of resistance exercise on exercise capacity respiratory function and quality of life in maintenance hemodialysis patients [J]. Journal of Nurse Advancement, 2020, 35(1): 23-8,33.

1219 Shen Huajuan, Dong Yongze, Hu Xiao, et al. Effects of dietary education of traditional Chinese medicine on the internal environment of patients with end-stage renal disease on maintenance hemodialysis [J]. Journal of Zhejiang University of Traditional Chinese Medicine, 2020, 44(5): 486-9,94.

1220 Shen Lintao. Analysis of the effects of exercise intervention on quality of life and sleep in uremic hemodialysis patients [J]. Self-care, 2020, (20): 39-40.

1221 CHEN Xiaoling, LI Xiaoqing, XIAOQING L I. Nursing research on the effect of exercise intervention on symptomatic hypotension in dialysis patients [J]. Chinese and Foreign Medicine Research, 2020, 18(10): 59-61.

1222 Chen Zhonghan. Observation on the effect of exercise rehabilitation to improve the complications of hemodialysis patients [J]. Frontiers of Medicine, 2020, 10(7): 230-1.

1223 CHENG Jiang, WANG Xulei, XU-LEI W. Efficacy of warm spleen and drain turbid soup combined with hemodialysis in the treatment of CRF and its effect on patients' 24 hUP and serum LPO levels [J]. Hainan Medicine, 2020, 31(17): 2184-7.

1224 Cheng Xiaoli, Yang Shifeng, Shi Liaojiang, et al. Efficacy of Astragalus and Epiphyllum Formula for the Treatment of Maintenance Hemodialysis in Chronic Renal Failure and Its Effects on Oxidative Stress, Microinflammatory Response, and Quality of Life of Patients [J]. Shaanxi Traditional Chinese Medicine, 2020, 41(10): 1436-9.

1225 Cui J, Chuan N S, Zhou Chunxia, et al. Effects of high flux hemodialysis combined with Bailing capsule on oxidative stress, microinflammatory status and quality of survival in hemodialysis patients with diabetic nephropathy [J]. Journal of Difficult Diseases, 2020, 19(8): 764-8.

1226 Dai Shanshan, Yu Haiyan, Lai Shuang, et al. Effects of incremental resistance exercise on hemoglobin and iron metabolism in maintenance hemodialysis patients [J]. West China Medicine, 2020, 35(7): 781-7.

1227 DONG Luyao, FAN Xing, XU Yuwei, et al. Study on the effect of paraformaldehyde starch on blood potassium level in patients with chronic renal failure [J]. China Urban and Rural Enterprise Health, 2020, 35(1): 151-4.

1228 Fan Yajuan. Alterations of human gut microbiome in non-dialysis chronic kidney disease and its non-invasive diagnostic value [D]; Zhengzhou University, Internal Medicine (Nephrology), 2020.

1229 FANG Hui, WANG Hui, RI Qing, et al. Effect of Sheng Vein Capsule combined with levocanidin on oxidative stress in uremic patients on maintenance hemodialysis [J]. Jiangsu Medicine, 2020, 46(6): 611-4.

1230 FANG Jilin, JIANG Hongli, LI Xiangdong, et al. Effect of chlorosartan potassium treatment of idiopathic membranous nephropathy on serum anti-PLA2R antibody [J]. Modern Biomedical Progress, 2020, 20(9): 1729-32.

1231 Fang Lugui, Ren Y, Guo Chuan, et al. Literature characterization of the use of Chinese medicine in peritoneal dialysis [J]. Journal of Traditional Chinese Medicine, 2020, 26(9): 134-6,43.

1232 Gao Haibo, Guo Cuicui, Wang Yuzhen, et al. Comparative observation on the effect of continuous renal replacement therapy and intermittent hemodialysis therapy in the treatment of sepsis [J]. Practical Chinese and Western Medicine Clinic, 2020, 20(6): 23-4.

1233 Gao H. Clinical efficacy of continuous renal replacement therapy versus intermittent hemodialysis in treating patients with severe acute renal failure [J]. China Medical Guide, 2020, 18(27): 84-5,8.

1234 Gao Liang, Han Baojie, Li Lanyuan, et al. Effects of bloodletting by stabbing at twelve well points on hypoxia and energy metabolism disorders in cerebral tissues of rats with cerebral ischemia [J]. Chinese Journal of Gerontology, 2020, 40(11): 2388-91.

1235 Gao Rongwei. Observation on the effect of levocanidin combined with huangkui capsule in the treatment of uremic hemodialysis patients and the effect on cardiac and renal function [J]. Journal of Hunan Normal University (Medical Edition), 2020, 17(6): 181-4.

1236 Gao Xiaofeng. Observation on the effect of sevelamer carbonate in the treatment of hemodialysis combined with hyperphosphatemia [J]. Huaihai Medicine, 2020, 38(4): 338-40.

1237 Gao Ying. Predictive value and correlation of blood cell parameters and retinol-binding protein in early diabetic nephropathy [D]; Zhengzhou University, Internal Medicine, 2020.

1238 He Qianxin. Role and molecular mechanism of miR--15a--5p in peritoneal dialysis--associated peritoneal fibrosis [D]; Zhengzhou University, Internal Medicine (Nephrology), 2020.

1239 Hu Wei-Ying. Evaluation of integrated nursing intervention strategies and effects in hemodialysis treatment for patients with uremia combined with chronic heart failure [J]. Self-care, 2020, (20): 85-6.

1240 Hua Meiting, You Jinfang, Wang Liqun, et al. Effects of different hemodialysis modalities on oxidative stress and microinflammatory status in patients with diabetic nephropathy [J]. Contemporary Nurses (上旬刊), 2020, 27(4): 140-2.

1241 Ji Zhuwa. Extraction of ginseng polysaccharides and their neuroprotective effects on mice with Parkinson's disease [D]; Zhejiang University, Pharmaceutical Engineering, 2020.

1242 Jian Hongchang. Effect of erythropoietin combined with ferrous succinate tablets on anemia-related indexes in patients with renal anemia on hemodialysis [J]. Practical Chinese and Western Medicine Clinic, 2020, 20(9): 62-4.

1243 Jiang, Tingting. A study of the effect of cognitive-exercise intervention on fear of falling in elderly hemodialysis patients [D]; Zhengzhou University, Nursing, 2020.

1244 Li He. Clinical Observation on the Effectiveness of the Teach-Back Method in the Application of Health Education for Hemodialysis Patients [D]; Hei Long Jiang University of Traditional Chinese Medicine, Nursing, 2020.

1245 Li M, Yang Danyu, Sun M, et al. Effects of Huangkui capsule combined with combined artificial kidney on inflammatory factors, oxidative stress and quality of life in maintenance hemodialysis patients with diabetic nephropathy [J]. Modern Biomedical Progress, 2020, 20(19): 3779-83.

1246 Li Qiao. Effectiveness of a staged behavioral care model to prevent dialysis imbalance syndrome in long-term hemodialysis patients [J]. General Practice Nursing, 2020, 18(9): 1098-100.

1247 LI YAN LING, GUO Y, YI G U O. Effectiveness of peer support health education on quality of life of maintenance hemodialysis patients [J]. China Health Education, 2020, 36(1): 82-5.

1248 Lian Fen, Wu Jing, Shen Huajuan, et al. Repeated-measures ANOVA of low-intensity recumbent gymnastics on dialysis adequacy in maintenance hemodialysis patients [J]. Nursing and Rehabilitation, 2020, 19(2): 55-8.

1249 Liang Li-Q. Hypoglycemic effect of Dioscorea polysaccharide and its mechanism [D]; Guangxi University of Traditional Chinese Medicine, Pharmacology, 2020.

1250 Liang Xue. Effectiveness of rehabilitation nursing based on 5E theory for chronic renal failure [J]. China Practical Medicine, 2020, 15(26): 191-2.

1251 Liang Zhazha, Zhou Huahong, Liang Yanjuan, et al. Effects of multidisciplinary cooperative exercise therapy on maintenance hemodialysis patients [J]. Qilu Nursing Journal, 2020, 26(17): 69-71.

1252 Liu Hui, Pei Hualian, Li Li, et al. Survey on training needs of continuous ambulatory peritoneal dialysis patients in Xinjiang and establishment of early warning model [J]. Journal of Continuing Nursing Education, 2020, 35(6): 568-71.

1253 Liu Ji-Mei. Effects of rehabilitation exercise training on sleep quality and fatigue level of uremic hemodialysis patients [J]. Systemic Medicine, 2020, 5(15): 165-8.

1254 Liu, Miaomiao. Effect of extracorporeal counterpulsation on early renal damage in patients with coronary artery disease combined with diabetes mellitus [D]; Zhengzhou University, Internal Medicine, 2020.

1255 Liu Pei. Research and Clinical Observation on the Treatment of Chronic Kidney Disease (Non-Dialysis) by Benefiting Kidney, Strengthening Spleen, Activating Blood and Shrinking Liver [D]; Chengdu University of Traditional Chinese Medicine, Integrative Medicine (Kidney Disease), 2020.

1256 LIU Wenjian, JIANG Chuanwei, WANG Hao, et al. Mechanism of hippocampal glutamatergic neuron mediated electroacupuncture on "Foot Sanli" to regulate gastrointestinal function [J]. Acupuncture Research, 2020, 45(11): 861-7,81.

1257 Liu YD, Gu HY, Liu K, et al. Clinical study of sequential hemodialysis combined with compound alpha-keto acids in the treatment of end-stage renal failure [J]. Journal of Clinical Psychosomatic Diseases, 2020, 26(2): 48-51.

1258 Liu ZW. Protective effect and mechanism of liraglutide on early kidney injury in type 2 diabetes mellitus [D]; Tianjin Medical University, Clinical Medical Internal Medicine, 2020.

1259 Lv Wenxi. Observation of ICU infectious shock patients given continuous renal replacement therapy blood purification treatment [J]. Health| Health Essentials, 2020, (1): 104-5.

1260 Lv Yingqi. Cardiac magnetic resonance evaluation of the effects of liraglutide and dagliflozin on cardiac function and its mechanism of action in patients with type 2 diabetes mellitus [D]; Tianjin Medical University, Clinical Medicine (Internal Medicine Endocrinology and Metabolic Disease), 2020.

1261 Luo Q, Qing Shanlin, Tian Zhong, et al. Clinical efficacy of milrinone combined with digitalis in the treatment of uremic hemodialysis combined with heart failure [J]. Western Medicine, 2020, 32(3): 434-7.

1262 Ma Xiaoling. Effects of disciplinary integrated rehabilitation care on compliance behavior and self-efficacy of maintenance hemodialysis patients [J]. China Medical Guide, 2020, 18(20): 247-8,51.

1263 MENG Jingwen, ZHENG Ziling, YAU Zhaoyuan, et al. Efficacy and safety of exercise therapy for the treatment of restless legs syndrome in hemodialysis patients: a systematic review and meta-analysis [J]. International Journal of Transplantation and Blood Purification, 2020, 18(6): 39-45.

1264 MIAO Hui, ZHAO QIWEN, QIWEN Z. Observations on the efficacy of levofloxacin combined with Bailing capsule on lower respiratory tract infection in hemodialysis patients with chronic renal failure [J]. Journal of Practical Clinical Medicine, 2020, 24(7): 31-3.

1265 Miao Wenyan. Correlation study of the effect of tai chi exercise combined with compound alpha--keto acids on IGF--1/IGFBP3 in maintenance hemodialysis patients [D]; Xinjiang Medical University, Internal Medicine (Nephrology), 2020.

1266 Mok, Ya-Wen. A randomized controlled study of dumbbell weighted swinging arm exercise on arteriovenous endovascular fistula in hemodialysis patients [D]; Southern Medical University, Nursing, 2020.

1267 Na Wei. A study on the effect of collaborative care model on sleep, exercise and nursing satisfaction of uremic hemodialysis patients [J]. World Journal of Sleep Medicine, 2020, 7(12): 2160-2.

1268 Ni Man. Clinical effect of continuous hemodialysis combined with hemoperfusion in treating patients with sepsis combined with acute kidney injury [J]. Zhongguo Nankang Med, 2020, 32(13): 4-6.

1269 Niu Tieming, Han Yi, Luan Xunfei, et al. Effects of aerobic exercise combined with resistance exercise on microinflammation and T-cell subsets in peritoneal dialysis patients [J]. Chinese Journal of Practical Internal Medicine, 2020, 40(6): 493-6,501.

1270 PAN Cuiping, ZHOU Shenxin, SHI Shanfen, et al. Efficacy and prognosis of end-stage diabetic nephropathy patients undergoing hemodialysis and peritoneal dialysis by COX regression analysis [J]. Modern Practical Medicine, 2020, 32(3): 311-3.

1271 Peng X. Y. Observation on clinical efficacy of levocanidin combined with hemodialysis in treating patients with chronic renal failure combined with heart failure [J]. Heilongjiang Medicine, 2020, 33(3): 571-3.

1272 Pu Wei. Nursing intervention of exercise therapy on hypotension in maintenance dialysis patients [J]. Health Care Guide, 2020, (34): 139.

1273 Qi Lu, Liu Ruifang, Wang Congmei, et al. Effects of continuous renal replacement therapy on renal function, hemodynamic indexes and serum inflammatory factor levels in patients with severe acute renal failure [J]. Chinese Journal of Practical Medicine, 2020, 47(7): 29-33.

1274 SANG Yongyan, WANG Haiyan, WANG Yu, et al. Effects of comprehensive rehabilitation nursing care guided by 5E theory on patients with chronic renal failure [J]. International Journal of Nursing, 2020, 39(4): 625-8.

1275 SHAO Shuqin, WANG Xiaomei, LIU Xiaoming, et al. Analysis of the efficacy of Lishukang capsule in treating non-dialysis CKD renal anemia and its effect on oxygen radical metabolism in vivo [J]. China Practical Medicine, 2020, 15(10): 17-9.

1276 Shi Kaixuan, Liu Xiaoli, Qiao Decai, et al. Exercise improves the behavior of a rat model of PD by modulating the functional connectivity plasticity of the cortico-striatal pathway [J]. Sports Science, 2020, 40(6): 49-58.

1277 Shi YAN, Hu ZJ, Zhao P, et al. Efficacy and safety analysis of hypoxia-inducible factor-prolinyl hydroxylase inhibitor for the treatment of renal anemia in hemodialysis patients [J]. Chinese Journal of Practical Internal Medicine, 2020, 40(11): 920-5.

1278 Shou Dan, Liang Xin, Jin Dan, et al. Meta-analysis of the Effect of Resistance Exercise on Blood Pressure Control and Exercise Tolerance Recovery in Hemodialysis Patients [J]. Journal of Nursing, 2020, 27(15): 44-9.

1279 Tian Ru, Luo Yang, Feng Xingzhong, et al. Effects of Ba Duan Jin on cognitive function and daily life ability of hemodialysis patients [J]. Beijing Traditional Chinese Medicine, 2020, 39(1): 11-4.

1280 Wang Juan. Effects of protocol care on compliance behavior and nutrition of hemodialysis patients [J]. Electronic Journal of Practical Clinical Nursing, 2020, 5(13): 45,198.

1281 WANG Yangchang, WEN Jiali, LUO Shujuan, et al. Efficacy of sodium bicarbonate tablets in the treatment of end-stage renal disease patients on maintenance hemodialysis [J]. Shenzhen Journal of Integrative Medicine, 2020, 30(13): 181-2.

1282 WANG Haijun, SHANG Ningning, ZHAO Hua'en, et al. Modulation of dopamine levels and insulin signaling in the ventral striatum of obese rats by treadmill exercise [J]. Chinese Journal of Sports Medicine, 2020, 39(3): 210-8.

1283 WANG Haiyan, CAI Xiaoqin, XIA Jianmei, et al. Application of behavioral staged change theory in the prevention of hyperphosphatemia in maintenance hemodialysis patients [J]. International Journal of Nursing, 2020, 39(3): 506-8.

1284 WANG Hongmei, DING Hong, HONG D. Application value of empowerment education for vascular access management in dialysis patients [J]. China Continuing Medical Education, 2020, 12(26): 186-90.

1285 Wang, Kai-E. Effects of Chinese medicine nursing intervention on purification effect and quality of life of uremic hemodialysis patients [J]. Dietary Health Care, 2020, 7(22): 179-80.

1286 Wang KK, Meng XJ, Han H, et al. Efficacy of hemodialysis versus continuous hemodialysis in the treatment of acute renal failure and the effects on patients' toxin clearance and renal function indexes [J]. Hebei Medicine, 2020, 26(5): 788-92.

1287 WANG Lei, DING Hong, HONG D. Analysis of the effect of continuous bedside hemofiltration therapy on patients with severe arsenic poisoning [J]. Chinese Journal of Endemic Disease Control, 2020, 35(5): 591-3.

1288 WANG L L, DING X Z, DING X. Cardiac rehabilitation exercise in patients with MHD combined with heart failure [J]. Journal of Clinical Cardiology, 2020, 29(5): 369-72.

1289 WANG LILI, DING XUE ZHI, XUE ZHI D. Effects of cardiac rehabilitation exercise on dialysis quality and exercise tolerance in patients with maintenance hemodialysis combined with cardiac insufficiency [J]. International Journal of Transplantation and Blood Purification, 2020, 18(5): 27-30.

1290 Wang Lianfeng, Shao Qing, Sheng Xueying, et al. Cognitive behavioral management in dialysis patients [J]. Journal of Chinese Medicine Management, 2020, 28(20): 158-9.

1291 Wang QX. Comparison of the distribution of nebulized drugs in the lungs between two nebulizers using DSDECT [D]; Tongji University, Emergency Medicine, 2020.

1292 WANG TAO, LIU YONG-MEI, YONG-MEI L I U. Effects of aerobic exercise on mineral metabolism, sleep quality and fatigue in maintenance hemodialysis patients [J]. Medical Information, 2020, 33(23): 77-9,95.

1293 WANG Wenjing, LI Yanbin, ZHANG Zewei, et al. Effects of exercise therapy combined with nursing intervention on nutritional status and quality of life in maintenance hemodialysis [J]. Clinical Medicine Practice, 2020, 29(2): 155-8.

1294 WUXIAO WANG, JIALING LI, YU TAO, et al. Observations on the prevention and treatment effects of bicycle exercise in dialysis on restless legs syndrome in uremic patients [J]. China Blood Purification, 2020, 19(4): 230-3.

1295 WANG SIU-YAN, QIN MING-HUA, MING HUA Q. Effects of TIME exercise rehabilitation education on somatic function and survival quality of maintenance hemodialysis patients [J]. Contemporary Nurses (Upper Ten Journal), 2020, 27(9): 136-8.

1296 Wang Xiaosong. Effects of Huangkui capsule on microinflammation and oxidative stress in hemodialysis patients with diabetic nephropathy [J]. Chinese Medical Science, 2020, 10(8): 56-9.

1297 Wang YH, Men YR, Li Y, et al. Effect of CRRT on the improvement of cardiac function and prognosis of patients with acute and chronic renal failure combined with heart failure [J]. Journal of Hunan Normal University (Medical Edition), 2020, 17(4): 134-7.

1298 Wang Yanmei. Analysis of the causes of anemia in hemodialysis patients and nursing countermeasures [J]. Dietary Health Care, 2020, (46): 202.

1299 Wang Yan. Effect of combining dietary care and aerobic exercise in peritoneal dialysis patients [J]. China Health Nutrition, 2020, 30(23): 198-9.

1300 Wei, Mengmeng. Organic anion transporters (Oats) and chymotrypsin//Ang II in adefovir-induced chronic kidney injury and methotrexate-induced acute kidney injury [D]; Lanzhou University, Pharmacy, 2020.

1301 Wei Qiaomu. Observation on the effect of exercise therapy with nursing intervention on nutritional status and quality of life of maintenance hemodialysis patients [J]. World Digest of Recent Medical Information (Continuous Electronic Journal), 2020, 20(7): 297-8.

1302 WU Jiao-hua, LIANG Yan-ping, LAI Xiaobing, et al. Effects of a precise quantitative intervention of walking exercise on maintenance hemodialysis patients [J]. General Practice Nursing, 2020, 18(4): 441-3.

1303 WU Ruijie, CHEN Zhenzhen, ZHENZHEN C. Application effect of 5E rehabilitation model in uremic peritoneal dialysis patients [J]. Zhongguo Nguo Kang Medicine, 2020, 32(19): 153-6.

1304 Wu, Xinjing. Pharmacodynamic studies on the antidiabetic cardiomyopathy effects of bamboo ginseng saponin IVa and the preparation and evaluation of its nanoformulation [D]; Air Force Military Medical University, Pharmacy; Pharmacy, 2020.

1305 Xia Ji-xiu. Evaluation of the effect of rehabilitation exercise nursing on the psychological state of hemodialysis elderly [J]. Psychology Monthly, 2020, (23): 118-9.

1306 XIAO Daojin, XU Ying, YING X. Application of simple ultrafiltration in adjuvant treatment of intractable heart failure [J]. Contemporary Nurses (Zhongdian), 2020, 27(8): 22-4.

1307 Xiao Dongbin. Preventive effect of coenzyme Q10 combined with atorvastatin on contrast nephropathy after coronary intervention in patients with chronic renal insufficiency [D]; Henan University, Clinical Medicine, 2020.

1308 Xie, Chengxia. The role and mechanism of zinc liposomes in reversing cholestatic liver fibrosis [D]; Sichuan University, Biochemistry and Molecular Biology, 2020.

1309 Xiong Kyung. Systematic evaluation and meta-analysis of complications and mortality in neonatal respiratory failure treated with intravenous-venous extracorporeal membrane lung oxygenation [D]; Chongqing Medical University, Pediatrics, 2020.

1310 Xiong M. Effects of individualized exercise program on prognosis and social regression of maintenance hemodialysis patients [J]. Contemporary Nurses (Zhongdian), 2020, 27(12): 75-7.

1311 Xu J. Effect of exercise prescription on treatment effect and survival quality of maintenance hemodialysis patients [J]. Electronic Journal of Practical Clinical Nursing, 2020, 5(39): 119,29.

1312 Xu WQ. Intervention effect of aerobic exercise on maintenance hemodialysis patients [J]. Longevity, 2020, (3): 89.

1313 Xue Hanyu. Clinical study on the improvement of myasthenia gravis in patients on maintenance hemodialysis by the embroidered ball exercise of Zhuang medicine [D]; Guangxi University of Traditional Chinese Medicine, Central and Western Medicine Clinic, 2020.

1314 YAN Si-Yuan. Role of iron death in a rat model of peritoneal fibrosis [D]; Hunan Normal University, Internal Medicine, 2020.

1315 Yang Jun. Effect of high flux hemodialysis on microinflammatory status in maintenance hemodialysis patients with diabetic nephropathy [J]. Dietary Health Care, 2020, 7(21): 56.

1316 Yang WQ. Clinical study of dialysis-related peripheral neuropathy [D]; Neurosurgery, Peking Union Medical College, Chinese Academy of Medical Sciences, 2020.

1317 Yang Yang. Analysis of the effect of 5E rehabilitation nursing on the prognosis and sleep quality of hemodialysis patients [J]. Reflexology and Rehabilitation Medicine, 2020, 29(9): 1-2,5.

1318 YAO Hai-Wen, YANG Mei-Hua, JIANG Qi-Jiang, et al. Efficacy of Prostadil combined with Renal Failure Tablets on chronic renal failure patients with hemodialysis and its effect on residual renal function, immunoglobulin, lung function serum inflammatory factor and hemodialysis indexes [J]. Chinese Journal of Gerontology, 2020, 40(24): 5232-6.

1319 Yi Qiuyue, Zhou Heping, Ming Ming, et al. Cerebral oxygen monitoring guides intraoperative temperature management in type A sandwich [J]. Journal of Cardiopulmonary Vascular Disease, 2020, 39(1): 49-53.

1320 Yuan Mei. Analysis of the effect of multidisciplinary cooperative exercise therapy applied to maintenance hemodialysis patients [J]. Medical Food Therapy and Health, 2020, 18(11): 7,13.

1321 YUAN ZHONGFEI, LIU XIANYAN, XIANYAN L I U. Effects of early nutritional support on oxidative stress status and quality of life in maintenance hemodialysis patients [J]. China Modern Physician, 2020, 58(6): 97-100.

1322 ZHANG Cai-Ling, CHEN Feng-Yu, CHEN Lian-Di, et al. Effects of aerobic exercise combined with resistance training on somatic function and quality of life of hemodialysis patients [J]. Chinese and Western Medicine Nursing (in Chinese and English), 2020, 6(11): 84-7.

1323 Zhang F. Effects of rehabilitation exercise training on sleep quality and fatigue level of uremic hemodialysis patients [J]. Health Care Guide, 2020, (43): 285.

1324 Zhang L, Li A M, Liu Y J, et al. Study on the intervention effect of aerobic exercise in dialysis on restless legs syndrome and psychological condition of maintenance hemodialysis patients [J]. Journal of Clinical Nephrology, 2020, 20(7): 586-90.

1325 Zhang LH, Zhou WP, Hong MQ, et al. Effects of resistance exercise on blood pressure and quality of life in maintenance hemodialysis patients [J]. Medical Equipment, 2020, 33(15): 136-8.

1326 Zhang, Xi-Mei. Meta-analysis of roxarestat in the treatment of non-dialysis anemic patients with chronic kidney disease [D]; Nanhua University, Clinical Medicine (Internal Medicine), 2020.

1327 Zhang Xiaohong. Effects of rehabilitation exercise nursing on psychological status of elderly hemodialysis patients [J]. Journal of Electrocardiography (Electronic Edition), 2020, 9(2): 174-5.

1328 Zhang Yungang, Han Jing, Zhang Silei, et al. Effects of urethane granules combined with leucovorin on renal function, oxidative stress and microinflammatory status in maintenance hemodialysis patients [J]. Hainan Medicine, 2020, 31(24): 3159-62.

1329 Zhang Zhi, Hu Song, Xiao Xuejun, et al. Clinical effect of thymidine injection combined with blood purification in treating patients with severe sepsis [J]. Chinese Journal of Physicians, 2020, 22(1): 142-5.

1330 Zhang ZN. Preparation of Curcumin Liposomes and Evaluation of Their Antitumor Effects in Vivo and Ex Vivo [D]; Anhui Medical University, Pharmacy, 2020.

1331 Zhang Fei. Meta-analysis of the efficacy of hemoperfusion combined with hemodialysis versus hemoperfusion alone in the treatment of acute paraquat poisoning [D]; Nanchang University School of Medicine, Emergency Medicine, 2020.

1332 ZHANG Yan, YUE Zhang, HU Meiyan, et al. Effect of exercise therapy in hemodialysis patients [J]. Nursing Practice and Research, 2020, 17(3): 90-2.

1333 Zhao P, Huang YL, He L, et al. Meta-analysis of the effects of bicycle exercise on exercise capacity and circulatory status in maintenance hemodialysis patients [J]. Chinese Family Medicine, 2020, 23(14): 1769-77.

1334 ZHAO Yanhong, LI Hongmei, SUN Yang, et al. Effects of high flux hemodialysis combined with Haikun Renxi on oxidative stress and microinflammatory status in diabetic nephropathy patients [J]. China Medical Journal, 2020, 17(4): 98-101,9.

1335 ZHENG FENG, HUANG XIONG-LIANG H. Effects of cardiac rehabilitation exercise on cardiac function indexes in chronic renal failure combined with cardiac insufficiency patients on maintenance hemodialysis [J]. Chinese and Western Medicine Nursing, 2020, 6(12): 45-8.

1336 Zhong Qing, Huang Xianli, Hao Yan, et al. Efficacy and prognosis of bimodal plasma exchange in antineutrophil cytoplasmic antibody-associated vasculitis requiring renal replacement therapy [J]. China Blood Purification, 2020, 19(9): 618-22.

1337 Zhou Shuyan, Zhang Juanjuan, Zhuang Ji, et al. Impact of IMB-guided nutritional assessment and care on nutritional status of maintenance hemodialysis patients [J]. China Modern Physician, 2020, 58(20): 162-5.

1338 Zhu Li-Yang, Lu Mei-Su, Wang Hong-Lin, et al. Effects of planned aerobic-resistance exercise on patients' nutritional status and dialysis hypotension during the dialysis interval [J]. Chinese Journal of Modern Nursing, 2020, 26(14): 1894-8.

1339 Zhu Zhichun, Liao Liangying, Tan Ping, et al. Meta-analysis of the Effect of Aerobic Exercise to Improve Sleep Quality in Patients with Sleep Disorder Comorbidity [J]. Massage and Rehabilitation Medicine, 2020, 11(21): 30-4.

1340 Bao Xiaoyan, Ding Jie, Li Jun, et al. Family social support intervention in elderly hemodialysis patients [J]. Evidence-Based Nursing, 2021, 7(9): 1202-5.

1341 Bian Longfeng. Safety and efficacy of extracorporeal hemoperfusion therapy on postoperative inflammatory response in patients with acute type A aortic coarctation [D]; Sichuan University, Surgery (Thoracic Surgery), 2021.

1342 CAI Yanju, CHEN Xiao, XIAO C. Effects of resistance exercise intervention on fatigue state and physical activity level in maintenance hemodialysis patients [J]. International Journal of Nursing, 2021, 40(20): 3734-8.

1343 Cen Jianhua. Effects of moxibustion therapy combined with low-intensity aerobic exercise on sleep quality and fatigue in maintenance hemodialysis patients [J]. World Journal of Sleep Medicine, 2021, 8(8): 1351-2.

1344 Chen H.Y., Lai F.M., Zeng L.M., et al. Efficacy analysis of hemoperfusion combined with hemodialysis and hemodialysis filtration in the treatment of chronic renal insufficiency in the elderly [J]. Journal of Clinical Nephrology, 2021, 21(7): 529-34.

1345 Chen JF. Effects of continuous blood purification therapy on oxidative stress and microinflammatory status in elderly critically ill patients with acute kidney injury [J]. Special Health, 2021, (21): 175-6.

1346 Chen Kangfu, Zhong Shaodong, Peng Shixiu, et al. Ultrasound-guided transversus abdominis plane (TAP) block combined with rectus abdominis sheath block in peritoneal dialysis placement in uremic patients [J]. Primary Medical Forum, 2021, 25(35): 5041-3.

1347 Chen Lars, Wang Tingting, Li Laimei, et al. Therapeutic effect and mechanism of compound thromboxane capsule on maintenance hemodialysis in elderly diabetic nephropathy [J]. Chinese Journal of Gerontology, 2021, 41(14): 3009-13.

1348 Chen Na. Role of exercise-music therapy on motor function, negative emotions and fatigue in maintenance hemodialysis patients [J]. Dialysis and Artificial Organs, 2021, 32(4): 69-70.

1349 Chen S, Cheng YY, Tao Zhaodi, et al. Effects of peer health education on psychological self-care ability and health knowledge of perimenopausal hemodialysis patients [J]. China Maternal and Child Health, 2021, 36(6): 1398-401.

1350 Chen Xiu Juan, Zhu Zhaoming, Su Bao Yin, et al. Effects of Senqi Qingtu Capsules on Nutritional Indicators, Oxidative Stress, Inflammatory Factors, and Residual Renal Function in Hemodialysis Patients [J]. Shaanxi Traditional Chinese Medicine, 2021, 42(3): 334-7.

1351 CHEN YANNA, ZHOU QISHENG, QI SHENG Z. Effects of PDCA cycle management on health knowledge acquisition and self-care ability of hemodialysis patients [J]. Heilongjiang Medicine, 2021, 34(1): 240-2.

1352 CHENG JING, TAO LING LING, LING LING T. Effectiveness of active limb exercise in intervening symptomatic hypotension in hemodialysis patients [J]. Practical Clinical Medicine, 2021, 22(1): 60-1,97.

1353 CHENG Xinjie, TANG Liqun, ZHANG Zhen, et al. Effects of recumbent bicycle exercise on dialysis adequacy in maintenance hemodialysis patients [J]. Minimally Invasive Medicine, 2021, 16(5): 724-7.

1354 Cui Chengji, Liu Chunyan, Zhang Hongbao, et al. Effects of Chinese medicine nursing intervention on dialysis quality of elderly hemodialysis patients [J]. Chinese Medicine Modern Distance Education, 2021, 19(15): 147-9.

1355 Cui Xueyan. Construction and application of nursing program for elderly patients with cerebral infarction based on 5E rehabilitation model [D]; Xinxiang Medical College, Nursing, 2021.

1356 DAI Shanshan, MA Yingchun, YING CHUN M. Effects of incremental resistance exercise in dialysis on nutritional status and body fat composition of maintenance hemodialysis patients [J]. Chinese Journal of Nephrology, 2021, 37(5): 434-7.

1357 Shan Li-Hong. Impact of continuity of care model on the nursing effect of continuous peritoneal dialysis patients [J]. Electronic Journal of Practical Clinical Nursing, 2021, 6(5): 82-4,1.

1358 Dong Chen Di. Investigation of sleep in patients with chronic kidney disease and analysis of influencing factors [D]; Guangzhou University of Traditional Chinese Medicine, Integrative Medicine Clinic, 2021.

1359 Dong Li-Na, Liu Guo-Ping, Liu Ai-Qin, et al. Effects of high flux hemodialysis combined with levocanidin on immune function, oxidative stress and microinflammatory status in elderly maintenance hemodialysis patients [J]. Modern Biomedical Progress, 2021, 21(22): 4335-9.

1360 Fan L. Effect of exercise on microinflammatory status of maintenance hemodialysis patients [J]. Oriental Medicinal Cuisine, 2021, (17): 37-8.

1361 Fang Kun. Testicular interstitial cytotoxicity of DEHP and its mechanism [D]; Sichuan University, Surgery, 2021.

1362 Feng Lei, Li Yunshu, Yang Jie, et al. Effects of virtual reality-based somatosensory game integrated into home dialysis exercise management on fall risk and quality of life of dialysis patients [J]. China Blood Purification, 2021, 20(5): 302-5,32.

1363 Feng Huan. A study on the application of multidisciplinary teamwork in volume management of maintenance hemodialysis patients based on the 5E rehabilitation model [D]; Anhui Medical University, Nursing, 2021.

1364 Feng Q, Buheliqi Maimeti, Zhang Lei, et al. Dumbbell weight-bearing arm-swinging exercise in the maturation of arteriovenous endovascular fistula in hemodialysis patients [J]. Xinjiang Medicine, 2021, 51(8): 866-8.

1365 Fu Jun-Xiang, Wang Ai-Min, Zhou Yun-Ping, et al. Effects of eight-duanjin on sleep quality and negative emotions of maintenance hemodialysis patients [J]. Journal of Nursing Management, 2021, 21(4): 285-90.

1366 Gao Zhan, Zhang Xiaohui, Hu Lingdi, et al. Observations on the Effectiveness of Collaborative Nursing Services in Hemodialysis Patients [J]. Medical Food Therapy and Health, 2021, 19(14): 111-2.

1367 Gu Su. Effects of empowering health education concepts on hemodialysis patients' self-management and blood biochemical indicators [J]. Contemporary Nurses (上旬刊), 2021, 28(10): 153-6.

1368 GUO Suping, SHI Bin, JI Xiaojing, et al. Impact of home walking exercise with the application of pedometer software+ weibo on the quality of life of maintenance hemodialysis patients [J]. Contemporary Nurses (上旬刊), 2021, 28(6): 135-8.

1369 He Chuanmei, Liu Jinxiu, Li Hui, et al. Effects of hyperbaric oxygen-assisted hemodialysis combined with perfusion therapy on renal function and immunoinflammatory factors in patients with end-stage diabetic nephropathy [J]. Chinese Journal of Nautical Medicine and Hyperbaric Medicine, 2021, 28(4): 460-3.

1370 He Xiangzhi. Clinical observation on the treatment of renal anemia on hemodialysis with Bailing capsule combined with recombinant human erythropoietin injection [J]. Chinese Folk Therapy, 2021, 29(3): 96-8.

1371 HOU Xuehui. Intervention value of implementing collaborative care on self-care ability and quality of life level of hemodialysis patients [J]. Healthy Women, 2021, (45): 177,60.

1372 Hu Cai-Ping. Clinical study on the effect of tonifying kidney and activating blood on bone metabolism level and oxidative stress in CKD--MBD [D]; Guangzhou University of Traditional Chinese Medicine, Integrative Medicine Clinic, 2021.

1373 Hu Chunyan, Liu Jianlin, Li Fangxiao, et al. Cardioprotective effect of shuxuning on dialysis rats with chronic renal failure and its effect on JAK2/STAT3 signaling pathway [J]. Journal of Integrative Cardiovascular and Cerebrovascular Diseases, 2021, 19(17): 2929-34.

1374 HU Chunyan, LIU Jianlin, JIAN Lin L. Effects of different treatment modalities on anemia status, calcium-phosphorus balance and oxidative stress indexes in patients with renal anemia uremia on maintenance hemodialysis [J]. Sichuan Journal of Physiological Sciences, 2021, 43(3): 438-40.

1375 Hua Qiong. Effects of eight-duanjin on microinflammatory status and exercise capacity of elderly peritoneal dialysis patients [J]. Chinese Convalescent Medicine, 2021, 30(7): 700-3.

1376 HUANG Bizhen, XIE Yulian, LI Xiaoting, et al. Effects of WeChat-based extended care on maintaining out-of-hospital self-health management behaviors and quality of life of hemodialysis patients [J]. World Digest of Recent Medical Information (Continuous Electronic Journal), 2021, 21(18): 71-3.

1377 Huang Hai-Ping, Nie Kai-Lan, He Na, et al. Effects of Beneficial Kidney Soup via Colonic Dialysis on Intestinal Bacterial Flora and Microinflammation in Chronic Kidney Disease Stage 3~5 Patients [J]. International Journal of Traditional Chinese Medicine, 2021, 43(12): 1199-203.

1378 Huang J. The role of structured educational management in improving the training effect of peritoneal dialysis patients [J]. Dialysis and Artificial Organs, 2021, 32(1): 51-3,6.

1379 Huang Mingmin. Analysis of the effect of comprehensive nursing intervention combined with exercise therapy in hemodialysis patients [J]. Frontiers of Medicine, 2021, 11(29): 125-6.

1380 Jiang Ren, Zhang Yizhen, Li Ping, et al. Ultrasound-guided abdominal wall nerve block combined with dexmedetomidine in peritoneal dialysis placement [J]. China Modern Physician, 2021, 59(25): 121-5.

1381 JIANG Xue, ZHAO Hui, LI Xuesong, et al. Effects of aerobic exercise on nutritional status of maintenance hemodialysis patients [J]. Pharmacy Weekly, 2021, 30(47): 187-8.

1382 Jiang J. Application of specialized integrated care in nutritional management of peritoneal dialysis patients [J]. Chinese and Foreign Medicine, 2021, 40(4): 149-51.

1383 Jiang Y, Cai WQ, Yan GL, et al. Aerobic exercise combined with progressive muscle relaxation training in peritoneal dialysis patients [J]. Chinese Journal of Modern Nursing, 2021, 27(34): 4721-6.

1384 JIANG Yunzhi, ZHU Dongyun, MA Jipei, et al. Effects of Baduanjin on the quality of life and psychological status of non-dialysis patients with chronic kidney disease stage 3～ 5 [J]. Journal of Chinese Medicine, 2021, 33(1): 145-8.

1385 Jiang, Zhangjie. Alterations in respiratory and delirium-like behaviors and their mechanisms after catheterization in mice [D]; Nanchang University School of Medicine, Internal Medicine (Respiratory), 2021.

1386 JIN Yong, CHEN Zhaoxia, CHAO XIA C. Clinical efficacy observation of ropinirole combined with exercise training in the treatment of uremic restless legs syndrome [J]. Contemporary Medicine, 2021, 27(13): 144-6.

1387 Kong Yurou, Liu Lin, Jiao Le, et al. Gait characteristics of maintenance hemodialysis patients and their influencing factors [J]. Chinese Journal of Rehabilitation Medicine, 2021, 36(7): 816-21.

1388 Li Chuyang, Lou Xiaoping, Nancy Cui, et al. Impact of nursing intervention based on behavioral change model on adherence, self-efficacy and quality of life of patients with chronic renal failure [J]. Qilu Nursing Journal, 2021, 27(1): 7-10.

1389 Li D. Effect of psychological intervention on the care of uremic patients during hemodialysis and its impact on quality of life [J]. Maternal and Child World, 2021, (30): 204.

1390 Li D. Studies on the function of the hemolysin gene of Aeromonas hydrophila and its mechanism of action [D]; University of Electronic Science and Technology, Biochemistry and Molecular Biology, 2021.

1391 Li Danyu. Effect of ustekin combined with hemodialysis in the treatment of diabetic ketoacidosis [J]. Zhongguo Nankang Med, 2021, 33(23): 13-5.

1392 Li D. Clinical efficacy analysis of continuous blood purification in the treatment of infectious shock with renal dysfunction [J]. World Digest of Current Medical Information, 2021, (63): 50-1.

1393 LI J, LI YYY, YANYAN L I. Effect of levocanidin combined with erythropoietin on clinical efficacy and psychological status of patients with renal anemia on maintenance hemodialysis [J]. International Journal of Psychiatry, 2021, 48(6): 1076-9,105.

1394 LI JING, ZHANG YU TING, YU TING Z. Effects of high flux hemodialysis on oxidative stress and microinflammatory status in patients with diabetic nephropathy [J]. Self-Care, 2021, (5): 282.

1395 Li Kana, Zheng Zhelan, Wang Jiangtao, et al. Peak strain dispersion and longitudinal myocardial strain for evaluation of left ventricular systolic function in uremic patients on different alternative therapies [J]. Chinese Journal of Medical Ultrasound (Electronic Edition), 2021, 18(3): 258-65.

1396 LI Lin, YANG Guowen, NG Haowen, et al. Effects of Bailing Tablet on intestinal flora of patients with chronic kidney disease stage 4-5 (non-dialysis) based on the theory of tonifying fire and warming earth [J]. World Digest of Current Medical Information, 2021, 21(95): 4-6,9.

1397 Li Shuxiang. Impact of quality nursing care on blood pressure control, medication adherence and self-management behavior of hypertensive patients on maintenance hemodialysis [J]. Cardiovascular Disease Prevention and Control Knowledge, 2021, 11(19): 57-9.

1398 Li Sufang, Yin Lianliang, Li Chengcheng, et al. Clinical application of coenzyme Q10 in the treatment of hypertension in hemodialysis patients [J]. Health Care Medicine Research and Practice, 2021, 18(4): 78-81.

1399 Li CH. Exploring the effects of hemodialysis on the levels of inflammation and oxidative stress in uremic patients [J]. Scientific Health Care, 2021, 24(6): 210.

1400 Li Yucui. The effect of exercise intervention in dialysis on somatic function in hemodialysis patients based on the Knowing, Believing and Doing model [D]; South China University, Nursing, 2021.

1401 Lian Minling, Yang Xiaoli, Wang Xiuduan, et al. Analysis of the application effect of "5A" nursing model in maintenance hemodialysis patients [J]. Chinese and Foreign Medicine, 2021, 40(27): 171-4.

1402 Liang Xiao, Yang Positioning, Wu Zixia, et al. Effects of febuxostat on cardiovascular and cerebrovascular events and serological indices in dialysis patients with asymptomatic hyperuricemia [J]. Journal of Tianjin Medical University, 2021, 27(6): 564-8.

1403 Liang Yanying, Cheng Guifeng, Yu Zhilin, et al. Clinical Observation of Aerobic Cycling on Relieving Constipation in Maintenance Hemodialysis Patients [J]. World Abstracts of Recent Medical Information (Continuous Electronic Journal), 2021, 21(18): 203-4.

1404 LIN Zhixiang, LI Zhe, TIAN Lu, et al. Effects of levocanidin combined with hemoperfusion and hemodialysis on renal function, oxidative stress, and calcium and phosphorus metabolism in patients with chronic renal failure [J]. China Health Engineering, 2021, 20(1): 136-8.

1405 LING Haiyan, DAI Yunxia, YUN XIA D. Effects of power orientation therapy on psychological stress and treatment adherence of hemodialysis patients with diabetic nephropathy [J]. Chinese Journal of Modern Nursing, 2021, 27(14): 1894-7.

1406 Ling Shuyi. Volume overload in diabetic hemodialysis patients and the clinical efficacy of kidney-enhancing and water-inducing formula [D]; Guangzhou University of Traditional Chinese Medicine, Chinese Medicine and Internal Medicine, 2021.

1407 Liu Honggui, Jin Zejun, Wang Jinbao, et al. Clinical effect of febuxostat in the treatment of hyperuricemia combined with maintenance hemodialysis in the elderly [J]. Chinese Journal of Gerontology, 2021, 41(21): 4758-60.

1408 Liu Min. Observation on the value of nursing intervention for maintenance hemodialysis patients [J]. Dietary Health Care, 2021, (2): 114.

1409 Liu Qixia. Effects of gynostemma saponin XLIX nanoparticles in the treatment of renal fibrosis and its mechanism [D]; Anhui Medical University, Pharmacy, 2021.

1410 LIU Sha, TANG Xueqin, XUEQIN T. Effects of cognitive-behavioral therapy combined with aerobic exercise on nutritional status and quality of life of hemodialysis patients [J]. Reflexology and Rehabilitation Medicine, 2021, 2(9): 164-6.

1411 Liu Shao-Fang, Li Jin-Hsiang, Chen Hai-Yan, et al. Effects of different dialysis modalities on uremic toxin clearance, inflammatory factors and oxidative stress status in MHD patients [J]. Journal of Molecular Diagnosis and Therapy, 2021, 13(10): 1656-9,64.

1412 Liu Sujiao. Impact of home care intervention on psychological health and medical compliance behavior of elderly dialysis patients with kidney disease [J]. Health| Health Must Read, 2021, (35): 89,91.

1413 Liu, Shuyun. Preparation of lignosulfonate nanoparticles and regulation mechanism [D]; Qilu University of Technology, Light Industry Technology and Engineering, 2021.

1414 LIU Zhengliang, ZHANG Sweet, LI Xiuyong, et al. Efficacy of Thromboxane capsule combined with aspirin on rethrombosis after arteriovenous endovascular fistula surgery for maintenance hemodialysis and its effect on serum PT, APTT and DD levels [J]. Chinese Journal of Traditional Chinese Medicine, 2021, 39(9): 189-92.

1415 Lu Hongmei. Clinical study on the treatment of non-dialysis patients with acute kidney injury by enema of Erhuang Tang and the mechanism of Erhuang Tang in treating acute kidney injury based on network pharmacology [D]; Chengdu University of Traditional Chinese Medicine, Clinics of Integrative Chinese and Western Medicine (Kidney Disease), 2021.

1416 Lv Yanhui, Chen Jianhua, Ge Jiali, et al. Effects of exercise therapy on microinflammatory state and hyperhomocysteinemia in maintenance hemodialysis patients [J]. China Blood Purification, 2021, 20(3): 166-70.

1417 Luo Yan, Wu Su-Min, Zhang Shao-Hua, et al. Effects of rehabilitation exercise intervention on sleep quality and quality of life of uremic hemodialysis patients [J]. World Journal of Sleep Medicine, 2021, 8(2): 196-8.

1418 Ma Tao, Li Xiuyong, Wang Li, et al. Effects of high-throughput hemodialysis combined with hematopoietin on renal anemia and oxidative stress in elderly patients on maintenance hemodialysis [J]. Chinese Journal of Clinical Physicians (Electronic Edition), 2021, 15(12): 1009-15.

1419 Miao Jiayi, Zhang Yiwen, Zhang Liyuan, et al. Effects of aerobic exercise combined with resistance exercise on lipid metabolism, osteoporosis and blood pressure in maintenance hemodialysis patients [J]. Chinese Journal of Nephrology, 2021, 22(10): 911-3.

1420 Pang P, Zhou XL, Xie ZF, et al. Meta-analysis of the efficacy and safety of hypoxia-inducible factor prolyl hydroxylase inhibitor in the treatment of renal anemia in dialysis patients [J]. China Blood Purification, 2021, 20(2): 99-106.

1421 PENG Gui-ying. Effect of intensive nursing care in preventing dialysis imbalance syndrome in long-term hemodialysis patients [J]. Chinese and Foreign Women's Health Research, 2021, (6): 117-8.

1422 Qiao Qicheng. Functional and mechanistic studies on the hypothalamic orexin-brachiocephalic caudal reticular nucleus and dorsolateral periaqueductal floor nucleus neural pathways regulating muscle tone [D]; Army Military Medical University, Physiology, 2021.

1423 QIN Panpan, CHEN Chaojuan, CHAOJUAN C. Effects of hemodialysis combined with hemoperfusion on oxidative stress and pancreatic islet function in diabetic patients with end-stage renal disease [J]. Hebei Medicine, 2021, 27(9): 1531-5.

1424 Rao Niu Ping. Effects of comfort care combined with exercise guidance on limb function, complications and psychological status of hemodialysis patients [J]. Healthy Women, 2021, (51): 214.

1425 Ren Guoyu. Effectiveness of maintenance hemodialysis in treating patients with diabetic nephropathy and its impact on their quality of life [J]. Diabetes New World, 2021, 24(2): 175-7.

1426 SHAO Ning, SUN Zhihua, HUANG Hao, et al. Clinical efficacy of α-lipoic acid combined with high flux hemodialysis on peripheral neuropathy in diabetic hemodialysis patients [J]. Journal of Clinical Nephrology, 2021, 21(4): 293-6.

1427 Toki I. Clinical characteristics and treatment analysis of patients with acute poisonous mushroom poisoning in emergency [J]. China Medical Engineering, 2021, 29(8): 26-9.

1428 Song Ya. Observations on the preventive effect of rational exercise care on hypotension in maintenance hemodialysis patients [J]. Pharmacy Weekly, 2021, 30(37): 113-4.

1429 Sun Dongni, Zhong Chunmei, Xiao Weijia, et al. Effect of comfort care for hemodialysis [J]. China Urban and Rural Enterprise Health, 2021, 36(4): 209-10.

1430 Sun W, Fan Dali, Jiang Z, et al. Effects of ABC emotional management combined with low phosphorus diet on psychological and nutritional status of hemodialysis patients with renal failure [J]. International Journal of Transplantation and Blood Purification, 2021, 19(4): 34-6.

1431 Tang Ou-feng, Chen Xiao-Feng, Zhou Hui, et al. Progress in the study of lung function in maintenance hemodialysis patients [J]. General Practice Nursing, 2021, 19(22): 3062-5.

1432 Tu H-T. PI3K/Akt-based study on the mechanism of anti-renal fibrosis of Qingxing and Turbid-lowering capsule and its monomer [D]; Guangzhou University of Traditional Chinese Medicine, Chinese Medicine and Internal Medicine, 2021.

1433 Wan D. Effectiveness of family participatory case management in maintenance hemodialysis patients [J]. Electronic Journal of Practical Clinical Nursing, 2021, 6(31): 88-91.

1434 WANG Haibo, LI Ye, WANG Guangwei, et al. Clinical study on the treatment of uremic hemodialysis-associated epilepsy by adding and subtracting the Definitive Epilepsy Formula combined with levetiracetam [J]. Hebei Traditional Chinese Medicine, 2021, 43(10): 1653-7.

1435 WANG Chenglong, LIU Yongmei, YONG MEI L. Effects of aerobic exercise on nutritional status, psychological condition and quality of life of maintenance hemodialysis patients [J]. World Digest of Recent Medical Information, 2021, 21(105): 274-6.

1436 Wang Chunrong, Chang Shengtao, Huang Miaochun, et al. Effects of self-efficacy education on weight management in hemodialysis patients [J]. China Urban and Rural Enterprise Health, 2021, 36(11): 106-8.

1437 Wang Chunrong, Liang Caihong, Chang Shengtao, et al. Effects of planned aerobic exercise-resistance exercise intervention on oxidative stress in maintenance hemodialysis patients [J]. Intelligent Health, 2021, 7(31): 157-9.

1438 Wang Fei, Gao Xiaoli, Wu Lulu, et al. Application of diabetic complication prevention and control exercises in hemodialysis patients with diabetic nephropathy [J]. Chinese Journal of Modern Nursing, 2021, 27(29): 3998-4003.

1439 Wang Juyan. Effect of comprehensive intervention on hemodialysis patients with sarcopenia [J]. Medical Food Therapy and Health, 2021, 19(7): 63-4.

1440 Wang Lei. Experimental study of ultrasound combined with microbubble-mediated targeted cell-mimicking methotrexate-loaded mesoporous silica nanoparticles for the treatment of arthritis in a rat model of CIA [D]; Sichuan University, Imaging Medicine and Nuclear Medicine, 2021.

1441 Wang Li, Zhou Meimei, Wang Jing, et al. Effects of dietary management combined with aerobic exercise on volume load and cardiac function in maintenance peritoneal dialysis patients [J]. Chinese Journal of Practical Nursing, 2021, 37(36): 2813-8.

1442 Wang Li-Fang. A study of the effects of FINGER-based multifactorial intervention on cognitive dysfunction in MHD patients [D]; Nanhua University, Nursing, 2021.

1443 Wang Shao-Hua. Clinical study of meridian acupuncture to improve motor function in hemodialysis patients [D]; China Academy of Traditional Chinese Medicine, Central and Western Medicine Clinic, 2021.

1444 Wang Shaohua, Li Xiaojuan, Luan Jie, et al. Clinical study on the improvement of motor function of hemodialysis patients by meridian acupuncture [J]. Chinese Journal of Nephrology, 2021, 22(3): 218-21.

1445 Wang Frost, Zhang Yi, Ying Shixin, et al. Comparison of the effects of ferrous fumarate docusate sodium capsule and polysaccharide iron complex capsule on oxidative stress in renal anemia patients with end-stage renal disease [J]. Journal of Clinical and Experimental Medicine, 2021, 20(4): 371-5.

1446 Wang Susu, Li Juanjuan, Hu Huagang, et al. Meta-analysis of the effect of exercise in dialysis on improving the quality of survival of maintenance hemodialysis patients [J]. Nursing Research, 2021, 35(6): 987-95.

1447 WANG Wenjuan, JIANG Xia, XIA J. Effects of different exercise modes on bone mineral density in maintenance hemodialysis patients [J]. Chinese Journal of Osteoporosis, 2021, 27(8): 1183-6,200.

1448 WANG Xiaona, LIU Nini, NI NI L. Analyzing the effect of using nursing process health education in the care of maintenance hemodialysis patients on patients' adherence to vascular access [J]. Healthy Women, 2021, (38): 156.

1449 Wang R. Effect of comfort care in hemodialysis [J]. Self-care, 2021, (11): 140-1.

1450 WU Menghan, WANG Yan, AI Shuanglan, et al. Improvement effects of exercise on cardiopulmonary function and psychological status of maintenance hemodialysis patients [J]. Chinese Journal of Frontiers of Medicine (Electronic Edition), 2021, 13(8): 67-70.

1451 XI Du Juan, TANG Jia Min, HUANG Wan Ying, et al. Application of Yoga Elastic Band in Rehabilitation Nursing Care of Regular Dialysis Patients [J]. International Journal of Nursing, 2021, 40(9): 1613-7.

1452 XIA Jinghua, SONG Dan, ZHU Wenbo, et al. Effectiveness of exercise intervention during dialysis to improve the quality of life and sleep quality of elderly maintenance hemodialysis patients under multidisciplinary collaborative team model [J]. Practical Geriatrics, 2021, 35(12): 1254-7.

1453 Xia Qing, Gao Xinlu, Li Cheng, et al. Effect of rat nerve growth factor combined with methylcobalamin on peripheral neuropathy in hemodialysis patients [J]. China Contemporary Medicine, 2021, 28(24): 48-51.

1454 Xiang Yuanxiang, Lin Shaorong, Zeng Shaoling, et al. Clinical value analysis of high flux hemodialysis for renal failure [J]. Dai Doctors, 2021, 6(7): 52-4.

1455 XIAO Yeqing, HE Liyu, HE L. Effects of hemoperfusion combined with hemodialysis on renal function-related serological indexes and complications in uremic patients [J]. Journal of Qiqihar Medical College, 2021, 42(22): 1942-6.

1456 Xie Ting, Bi Liming, Gao Jun, et al. Effects of Badaanjin Training Intervention on Fatigue Level, Sleep Quality and Psychological State of Maintenance Hemodialysis Patients [J]. Clinical Medicine Research and Practice, 2021, 6(29): 144-6,62.

1457 Xu Meixian, Liu Gang, Cao Lijing, et al. A non-randomized controlled trial of early blood purification techniques for the treatment of septic shock in children [J]. Chinese Journal of Evidence-Based Pediatrics, 2021, 16(3): 204-8.

1458 Xu Qinjuan, Hu Yanfei, Hu Huagang, et al. Net Meta-analysis of Different Exercise Modes on Improving Walking Ability of Maintenance Hemodialysis Patients [J]. PLA Nursing Journal, 2021, 38(7): 1-5.

1459 Xu W, Huang W, Ni Xiangrong, et al. Effects of hyperbaric oxygen combined with detoxification and kidney-fixing soup treatment on microinflammatory state and renal function in hemodialysis patients with end-stage renal disease [J]. Chinese Journal of Nautical Medicine and Hyperbaric Medicine, 2021, 28(2): 192-6.

1460 Xu Yanyan. Clinical effects of high flux dialysis on chronic renal failure patients and the impact of antioxidant function [J]. Heilongjiang Medicine, 2021, 45(4): 341-3.

1461 Hsu, J.-H. Evaluation of the effect of CRRT in the treatment of acute renal failure due to infectious shock [J]. China Practical Medicine, 2021, 16(27): 45-7.

1462 Xue, Pingfei. Study on the efficacy of in vitro cardiopulmonary resuscitation in rats and the mechanism of protection of neurons in the hippocampal CA1 region [D]; Qingdao University, Anesthesiology, 2021.

1463 YAN Xing, HUANG Xue-Fang, HE Min-Jing, et al. Effects of metabolic-equivalent-based moderate-intensity aerobic exercise on sleep quality and fatigue in maintenance hemodialysis patients [J]. General Practice Nursing, 2021, 19(29): 4130-3.

1464 Yang J, Feng L, Fu L, et al. Observations on the improvement of home exercise compliance and post-dialysis fatigue in dialysis patients with the "web-mediated+ exercise" program [J]. Journal of Clinical Nephrology, 2021, 21(7): 583-8.

1465 Yang Linlin. Comparison of clinical efficacy of different initial treatment programs for peritoneal dialysis-associated peritonitis [J]. China Rural Health, 2021, 13(22): 26-7.

1466 Yang Xiaoling. Effects of exercise therapy on nutritional status and quality of life of maintenance hemodialysis patients [J]. China Health Standard Management, 2021, 12(14): 141-4.

1467 Yang Y. Preparation of nano-emulsion eye drops of Hambutine and its pharmacokinetics in rabbit eyes [D]; Zhengzhou University, Ophthalmology, 2021.

1468 Yao Lan. Analysis of the preventive effect of rational exercise care on hypotension in maintenance hemodialysis patients [J]. Oriental Medicinal Cuisine, 2021, (15): 185.

1469 Yao L. Effects of exercise nursing intervention on cardiopulmonary function and psychological status of patients undergoing maintenance hemodialysis [J]. Chinese and Western Medicine Nursing (in English), 2021, 7(8): 151-3.

1470 YAO YAN, PENG YUAN, YUAN P. Impact of collaborative nursing interventions on adherence to sodium and water control and quality of life in elderly hemodialysis patients with chronic kidney disease [J]. Electronic Journal of Practical Clinical Nursing, 2021, 6(36): 100-3.

1471 Yi Min, Zhang Fenglian, Bai Qin, et al. Effect of renal failure capsule combined with urokinase on residual renal function and vascular endothelial function in uremic hemodialysis patients [J]. Journal of Naval Medicine, 2021, 42(1): 114-7.

1472 Yi Yanhong. Clinical effects of levocanidin in uremic hemodialysis patients and its role in improving patients' nutritional status [J]. Health Care Literature, 2021, 22(7): 86-7.

1473 Yu XT, Cao SM, Ji SJ, et al. Effects of exercise intervention in dialysis on maintenance hemodialysis patients [J]. Journal of Nursing, 2021, 36(17): 5-8.

1474 Yu XT, Cao SM, Ji SJ, et al. Meta-analysis of the effects of exercise on exercise capacity and dialysis efficiency in maintenance hemodialysis patients [J]. China Blood Purification, 2021, 20(8): 525-30.

1475 Yuan Xiazhi, Hua Ying, Fang Jinqiong, et al. Application of horizontal bicycle exercise in preventing hypotension in patients undergoing hemodialysis [J]. Nursing and Rehabilitation, 2021, 20(2): 1-4.

1476 Zhang Chengxiu. Effects of low-intensity aerobic rehabilitation exercise on sleep quality and fatigue in maintenance hemodialysis patients [J]. Reflexology and Rehabilitation Medicine, 2021, 2(21): 171-3.

1477 Zhang Chengxiu. Effects of rehabilitation exercise on sleep quality and fatigue level of hemodialysis patients with chronic renal failure [J]. Reflexology and Rehabilitation Medicine, 2021, 2(14): 172-4.

1478 Zhang JD. Effects of continuous renal replacement therapy on renal function and serum IL-6, TNF-α, and SAA levels in patients with severe acute renal failure [J]. Clinical Medicine, 2021, 41(9): 51-3.

1479 Zhang Juan. Effects of stage change theory-oriented health promotion on compliance behavior of hemodialysis patients with uremia with restless legs syndrome [J]. Heilongjiang Medicine, 2021, 45(14): 1481-2.

1480 Zhang LH, Zhou WP, Hong MQ, et al. Optimization of Resistance Exercise for Hemodialysis-Related Hypotension [J]. Cardiovascular Disease Prevention and Control Knowledge, 2021, 11(7): 51-3,6.

1481 ZHANG Linlin, FAN Yongzhao, GU Ruiting, et al. Wheel running pre-exercise improves cognitive function in rats with vascular dementia by inhibiting the loss of catecholamines in striatal brain regions [J]. Chinese Journal of Sports Medicine, 2021, 40(4): 287-93.

1482 Zhang Rui, Wang Zicheng, Jiang Rongli, et al. Efficacy of Fenghui Huangqi Tang Plus Flavor in the Treatment of Diabetic Nephropathy and Its Effect on Glycolipid Metabolism and Oxidative Stress in Patients [J]. Shaanxi Traditional Chinese Medicine, 2021, 42(8): 1049-52.

1483 Zhang Y. Study on the effect of aerobic exercise on maintenance hemodialysis patients [J]. Family medicine-medicine selection, 2021, (2): 322.

1484 ZHAO Bing, LIU Pingfu, WEI Xiaodong, et al. Evaluation of the efficacy of Chinese medicine treatment program for chronic renal failure [J]. World Abstracts of Recent Medical Information, 2021, 21(75): 408-9,11.

1485 ZHAO Jun, ZHAO Shuangshuang, SHUANG-SHUANG Z. Analysis of the efficacy of CRRT combined with levocanidin in the treatment of chronic renal failure combined with acute heart failure [J]. China Modern Medical Journal, 2021, 31(10): 15-9.

1486 Zhao ZJ. Preparation and functional study of buckwheat flavonoid nano-emulsion delivery carriers [D]; Shanxi University, Biochemistry and Molecular Biology, 2021.

1487 Zheng Miaomiao, Zhan Hongliang, Wu Teng, et al. Analysis of the effect of different blood purification methods in treating acute kidney injury after cerebral hemorrhage [J]. Medical Theory and Practice, 2021, 34(10): 1668-70.

1488 ZHENG Y, ZHANG Y, LI R, et al. Effectiveness of applying multidisciplinary cooperative exercise therapy in maintenance hemodialysis patients [J]. International Journal of Nursing, 2021, 40(14): 2610-4.

1489 Zheng Yanqi. Design and application study of the Home Exercise Instruction Manual for Maintenance Hemodialysis Patients [D]; HUZHOU NORMAL COLLEGE, EMERGENCY NURSING, 2021.

1490 ZHENG YUSHOU, WANG FUJUN, FUJUN W. Effects of exercise therapy on fatigue condition, psychological state and sleep quality of hemodialysis patients [J]. Reflexology and Rehabilitation Medicine, 2021, 2(13): 156-8.

1491 Zhong Henghe. Clinical efficacy analysis of leucovorin combined with hemodialysis in treating patients with chronic renal failure combined with heart failure [J]. Healthy Friends, 2021, (22): 285.

1492 Zhou Lijuan, Bian Yueqiu, Wang Xi, et al. Effects of eight-duanjin on fatigue symptoms and sleep quality of hemodialysis patients [J]. Tianjin Nursing, 2021, 29(2): 129-33.

1493 Zhou Liping. Effects of rehabilitation exercise training on sleep quality and fatigue level of uremic hemodialysis patients [J]. Oriental Medicinal Diet, 2021, (23): 120.

1494 ZHOU Yufei, WANG Xiangjuan, ZHENG Huafeng, et al. Effects of tea polyphenols on cardiac function in chronic renal failure patients on maintenance hemodialysis [J]. Contemporary Medicine, 2021, 19(9): 133-4.

1495 BU I, XU FI, FEI X. Positive effects of bicycle exercise in the life of hemodialysis patients [J]. Family Life Guide, 2022, 38(3): 86-8.

1496 CAI Guomei, HU Jingwen, WANG Quanrui, et al. Effects of exercise rehabilitation on nutritional status and fatigue syndrome in maintenance hemodialysis patients [J]. Chinese Contemporary Medicine, 2022, 29(16): 140-3,7.

1497 Cao Bing, Wang Fengmei, Mo Ying, et al. Comparison of the clinical effects of roxarestat and erythropoietin in the treatment of renal anemia in patients undergoing maintenance hemodialysis [J]. Journal of Clinical Rational Drug Use, 2022, 15(14): 117-20.

1498 Zeng Xiaojun, Xie Kewei, Li Ping, et al. Thiamine combined with folic acid for the treatment of cognitive impairment in maintenance hemodialysis patients [J]. Shanghai Medicine, 2022, 45(1): 3-9.

1499 Shen Peiyuan, Yang Suqin, Yang Suqin. The role of evidence-based nursing in the care of patients with cerebral infarction in the emergency care unit [J]. Journal of Modern Nursing Medicine, 2022, 1(5).

1500 Chen Guanjie, Zhang Hailin, Yin Lixia, et al. Construction and application of an exercise intervention program for patients with maintenance hemodialysis combined with sarcopenia [J]. Chinese Journal of Nursing, 2022, 57(7): 798-806.

1501 CHEN Guanjie, ZHANG Hailin, YIN Lixia, et al. Effects of exercise in dialysis on cognitively debilitated patients on maintenance hemodialysis [J]. Journal of Nursing, 2022, 37(20): 33-7.

1502 Chen H, Zhou WL, Zhang H, et al. Effects of incremental resistance exercise training in dialysis on dialysis quality and psychological status of middle-aged maintenance hemodialysis patients [J]. International Journal of Transplantation and Blood Purification, 2022, 20(2): 46-8.

1503 Chen J, Liu YX, Luo XJ, et al. Meta-analysis of the effects of exercise intervention on somatic function in maintenance hemodialysis patients [J]. China Blood Purification, 2022, 21(11): 850-7.

1504 Chen Juanjuan, Dong Chunxiu, Sun Yan, et al. Therapeutic efficacy of hemoperfusion combined with hemodialysis in acute organophosphorus poisoning [J]. Journal of Guangzhou Medical University, 2022, 50(6): 94-8.

1505 Chen S.H., Mai L.Y., Feng J.X., et al. Effects of aerobic exercise on psychological status, physiological function and quality of life of peritoneal dialysis patients [J]. Internal Medicine, 2022, 17(3): 348-50.

1506 Chen Yidan. Construction of a yeast microencapsulated drug delivery system for co-delivery of nucleic acids/statins and its oral targeting for the treatment of atherosclerosis* [D]; Army Medical University, Pharmacy, 2022.

1507 CHEN Yongwei, WEI Yanbin, DU Huanhuan, et al. Effects of aerobic exercise based on safe heart rate control on exercise tolerance, cardiorespiratory fitness and fatigue in uremic maintenance hemodialysis patients [J]. General Practice Nursing, 2022, 20(24): 3386-8.

1508 CHEN Yuqing, LUO Libing, LIN Zijuan, et al. Effects of eight-duanjin on volume load status and dietary compliance of peritoneal dialysis patients [J]. Fujian Traditional Chinese Medicine, 2022, 53(12): 63-5.

1509 Qiu Lin, Zhang Huanqiao, He Xiaoxue, et al. Efficacy of hemodialysis combined with reduced glutathione in the treatment of chronic renal failure [J]. Medical Clinical Research, 2022, 39(10): 1584-6.

1510 Fan Xiaobo, Jiang Yanping, Zhang Xiaoyan, et al. Application of upper limb rehabilitation exercises in maintenance hemodialysis patients [J]. Chinese Journal of Nursing, 2022, 57(21): 2572-8.

1511 FANG Yongbin, GAO Gaofeng, FENG G. Clinical efficacy of sodium thiosulfate in the treatment of calcification defense in dialysis patients with chronic kidney disease stage 5 [J]. Journal of Clinical Rational Drug Use, 2022, 15(34): 91-4.

1512 Feng Jing, Zhang Xingkai, Sun Yong, et al. Observation on the Effect of Oxygen Therapy Combined with Low Temperature Sodium Regulation Mode in Preventing Hypotension in Hemodialysis [J]. Journal of Clinical and Experimental Medicine, 2022, 21(21): 2292-6.

1513 Feng Ling, Liang Wei, Zhao Jing, et al. Effect of valsartan combined with leucovorin on cardiac and renal function in patients with chronic renal failure on hemodialysis with heart failure [J]. Hainan Medicine, 2022, 33(9): 1122-5.

1514 Feng Menghan. A study based on fMRI to explore the brain function characteristics of sleep disorders in hemodialysis patients and the mechanism of auricular stimulation [D]; Guangzhou University of Traditional Chinese Medicine, Clinical of Integrative Medicine and Western Medicine, 2022.

1515 Feng Sujuan, Zhang Peixuan, Sang Shengmei, et al. Effects of Focused Solution Short-Term Therapy Combined with Traditional Chinese Medicine Five Elements Music Therapy on Hemodialysis Patients [J]. Psychology Monthly, 2022, (23): 130-2,65.

1516 FENG YAN-LING, GONG PLAN, GONG J-H. Effects of WeChat-based platform continuity of care on dietary compliance and quality of life of hemodialysis patients with diabetic nephropathy [J]. Medical Food Therapy and Health, 2022, 20(14): 9-11,22.

1517 Fu Junxiang. Study on the application effect of Ba Duan Jin exercise in maintenance hemodialysis patients [D]; Qingdao University, Nursing, 2022.

1518 Fu Shezhu, Yang Fang, Sun Mengying, et al. Impact of mobile medical app home follow-up model on peritoneal dialysis patients' self-management ability and nutritional status [J]. International Journal of Nursing, 2022, 41(23): 4405-9.

1519 GINGGING YANG, LIU YERRONG, FAN DONGYING, et al. Effects of continuity of care on hemodialysis patients with diabetic nephropathy [J]. Gansu Medicine, 2022, 41(4): 359-61.

1520 Geng Huaiying, Zhu Aixia, Chen Xuexun, et al. Effects of low-flow oxygenation on cognitive function and quality of survival in maintenance hemodialysis patients [J]. Chinese Contemporary Medicine, 2022, 29(26): 51-4.

1521 Gu Xi. Serum metabolomic analysis of patients with chronic kidney disease [D]; Zhengzhou University, Internal Medicine (Nephrology), 2022.

1522 GU Min, ZHENG Yun, YUN Z. Intervention of aerobic exercise on fatigue status of maintenance hemodialysis patients [J]. Health Care Guide, 2022, (40): 93-6.

1523 He Guixiang. Protective effects of antler disc protein fractions of Meihua deer on LPS/D-GalN-induced acute liver injury in mice and their mechanisms [D]; Jilin Agricultural University, Wildlife Conservation and Utilization, 2022.

1524 He Lili. Effects of health education on self-management ability of non-knowledgeable elderly maintenance hemodialysis patients under the focused solution model [J]. Chinese Contemporary Medicine, 2022, 29(27): 141-4.

1525 He Qien, Ying Guanghui, Chen Zhaogui, et al. Meta-analysis of the interventional effects of exercise therapy on hemodialysis restless legs syndrome [J]. Chinese Journal of Integrative Nephrology, 2022, 23(10): 876-81,943.

1526 Hong Li-Mei. Effects of chronic care management on disease knowledge, treatment adherence and quality of life in patients with nephrotic syndrome [J]. International Journal of Nursing, 2022, 41(4): 705-8.

1527 Huang Chengguo, Meng Quanyu, Chestnut Ting, et al. Exploration of the value of urethraldehyde granules in assisting MHD in the treatment of uremia [J]. Chinese Journal of Nephrology, 2022, 23(3): 259-61.

1528 Huang Xiaoqin, Liu Qunying, Zhan Yun, et al. Clinical study on the treatment of diabetic nephropathy combined with itchy skin on maintenance hemodialysis by clearing heat and exuding dampness soup [J]. Sichuan Traditional Chinese Medicine, 2022, 40(11): 134-7.

1529 Huo Ai-Jing. Effectiveness of hemoperfusion in treating patients with secondary hyperparathyroidism on long-term blood purification [J]. Zhongguo Nongkang Med, 2022, 34(15): 50-3.

1530 JIANG FENG YI, HE XING LAI, XING LAI H. Analysis of the effects of diversified health education on self-efficacy and behavioral adherence of hemodialysis patients [J]. Electronic Journal of Clinical Medicine Literature, 2022, 9(11): 73-5.

1531 Jiang X. Effects of Lishukang capsule on inflammatory response and oxidative stress in maintenance hemodialysis patients [J]. New Chinese Medicine, 2022, 54(2): 78-82.

1532 Kong, Pingping. Effect of sacubitril valsartan on cardiac remodeling in hemodialysis patients with ejection fraction preserved heart failure [D]; Zhengzhou University, Internal Medicine (Nephrology), 2022.

1533 Lan Yang. Effect of hemodialysis combined with human erythropoietin in treating patients with chronic renal failure combined with anemia [J]. China Minkang Medicine, 2022, 34(13): 27-9,36.

1534 LAI Ning, CHEN Hualing, LI Maojun, et al. Effect of in vitro anticoagulation with citric acid in continuous renal replacement therapy for patients with severe burns [J]. Chinese Journal of Burns and Wound Repair, 2022, 38(1): 29-37.

1535 LI Bai, JIAO Tian J, TIAN JIE J. Efficacy of exercise therapy on hypotension and restless leg syndrome in uremic patients on dialysis and its effect on dialysis adequacy [J]. International Journal of Transplantation and Blood Purification, 2022, 20(4): 6-9.

1536 Li Changfeng, Xiao Beibei, Cheng Miao, et al. Effect of hemodialysis filtration therapy on renal fibrosis in patients with diabetic nephropathy [J]. Shenzhen Journal of Integrative Medicine, 2022, 32(12): 83-6.

1537 Li Chuyang, Huang Zheng, Wang Dandan, et al. Effects of health education based on the information motivation behavioral skills model on the self-management ability of chronic renal failure patients [J]. International Journal of Nursing, 2022, 41(7): 1263-7.

1538 Li J, Kuang JX, Fang F, et al. Effects of osteotriol on oxidative stress and microinflammatory status in patients with diabetic nephropathy [J]. Modern Practical Medicine, 2022, 34(11): 1488-90.

1539 Li Q, Yao B, Ge Lijuan, et al. Effects of high flux hemodialysis on metabolite clearance and nutritional status of diabetic nephropathy patients [J]. Health Medicine Research and Practice, 2022, 19(6): 10-3.

1540 Li Xisheng, Ma Shixing, Wang Qinchao, et al. Effectiveness of high-flux and low-flux hemodialysis in the treatment of diabetic nephropathy and the effects on patients' blood GSH-Px, MDA, and SOD levels [J]. Hainan Medicine, 2022, 33(19): 2467-70.

1541 Li Xiaoya. Study on the mechanism of phospholipase A2 toxicity in jellyfish jellyfish [D]; Chinese People's Liberation Army Naval Military Medical University; Naval Military Medical University, Specialty Medicine, 2022.

1542 Li Xuhua. Effect of auricular acupuncture point burying beans combined with umbilical dressing care on constipation in maintenance hemodialysis patients [J]. Guangming Traditional Chinese Medicine, 2022, 37(17): 3174-7.

1543 Li Xumei, Li Hao, Guo Lanying, et al. Effects of exercise therapy on exercise capacity and quality of life of maintenance hemodialysis patients [J]. Chinese Journal of Practical Nursing, 2022, 38(28): 2178-83.

1544 Li Yi-Nan, Ma Tao, Zhang Lei, et al. Aerobic-Resistance Exercise Combined with Stratified Intervention in Maintenance Hemodialysis Patients [J]. China Medical Journal, 2022, 19(12): 168-72.

1545 Li YB. Gentianoside modulates the TGF-β/Smads pathway and attenuates inflammation and oxidative stress to improve peritoneal fibrosis in rats [D]; Southwest Medical University, Integrative Nephrology, 2022.

1546 Li YB, Zhang J, Zhang ZY, et al. Inhibitory effect of gentianoside on peritoneal fibrosis in rats and its mechanism [J]. Chinese Journal of Pathophysiology, 2022, 38(6): 1075-82.

1547 LIANG Congdie, ZHANG Liming, LI Ming Z. Effects of hyperbaric oxygen therapy on cognitive function of uremic patients on maintenance hemodialysis [J]. Chinese Journal of Nautical Medicine and Hyperbaric Medicine, 2022, 29(2): 193-7.

1548 LIANG Yan, ZHANG Xiaoqin, XIAO Qin Z. Application effect of bicycle exercise in patients with maintenance hemodialysis uremia complicating restless leg syndrome [J]. China Clinical Nursing, 2022, 14(6): 331-3.

1549 Lin Wan, Yang Yu, Chen Lan, et al. Analysis of the effect of nursing intervention on hyperphosphatemia in young maintenance hemodialysis patients [J]. Chinese and Foreign Medicine, 2022, 41(4): 172-6.

1550 Lin Ying, Huang Bingbing, Wu Yaqin, et al. Effects of carvedilol and metoprolol on clinical efficacy and cardiac function in maintenance hemodialysis patients [J]. Journal of Central South University for Nationalities (Natural Science Edition), 2022, 41(4): 425-30.

1551 Ling Ling. Analysis of the effect of applying refined care model in hemodialysis care for end-stage diabetic nephropathy [J]. Intelligent Health, 2022, 8(21): 88-91.

1552 LIU Fang, WU Han, ZHANG Yingying, et al. Application of incremental resistance exercise training combined with WeChat health education platform in the nursing care of maintenance hemodialysis patients with chronic renal failure [J]. China Medical Journal, 2022, 19(3): 162-5,81.

1553 Liu Lanying. Effects of individualized exercise instruction on sleep quality and quality of life in hemodialysis patients [J]. World Journal of Sleep Medicine, 2022, 9(4): 754-6.

1554 Liu LJ. Nursing effect of moderate aerobic exercise in uremic maintenance hemodialysis patients [J]. Sports Illustrated, 2022, (8): 118-9,21.

1555 LIU YINYAN, XU FENGLING, FENGLING X U. Application effect of cycling exercise combined with respiratory training in elderly maintenance hemodialysis patients [J]. Clinical Medicine Research and Practice, 2022, 7(18): 176-8.

1556 LIU Zhen, COMFORT, ZHANG Xuekang, et al. Observation on myocardial protection efficacy of sodium creatine phosphate in patients undergoing peritoneal dialysis tubing placement [J]. Drug Evaluation, 2022, 19(2): 90-3.

1557 Liu Qianghui, Gao Bifeng, Li Xiaohui, et al. The efficacy of enema combined with prostaglandin in the treatment of chronic renal failure [J]. Shenzhen Journal of Integrative Medicine, 2022, 32(5): 37-9.

1558 Lu SQ. Salvianolic acid A inhibits GSK3β to ameliorate peritoneal fibrosis by regulating Nrf2 and NF--κB signaling pathways [D]; Zhengzhou University, Internal Medicine (Nephrology), 2022.

1559 Lu Manyu. A study of TCM behavioral intervention and health management on nutritional status of MHD patients [D]; Liaoning University of Traditional Chinese Medicine, TCM Internal Medicine, 2022.

1560 Luo Li, Zheng Jianqin, Zhang Xiaohong, et al. Clinical study on the renal protective effect of Renal Failure Health Formula on chronic renal failure patients under oxidative stress [J]. Chinese Medicine Clinical Research, 2022, 14(9): 61-4.

1561 Luo Q. Sorafenib nanolipid carrier eye drops for corneal neovascularization therapy: preparation, in vitro and in vivo studies [D]; Zhengzhou University, Pharmacology, 2022.

1562 Luo Xiju, Deng Siyan, Chen Jing, et al. Net Meta-analysis of the Effect of 9 Exercise Modalities on Improving Dialysis Adequacy in Hemodialysis Patients [J]. Chinese Journal of Nursing, 2022, 57(23): 2921-9.

1563 Ma Z H, Meng Y, Bit Y L, et al. Effects of resistant starch on inflammation, uremic toxins and renal function in patients with chronic kidney disease: a systematic evaluation and Meta-analysis [J]. China Food and Nutrition, 2022, 28(7): 46-52.

1564 MAO Yufeng, FAN Jiayi, CHANG Juan, et al. Clinical effect of roxarestat capsules combined with recombinant human erythropoietin injection in the treatment of renal anemia on peritoneal dialysis [J]. International Journal of Urology, 2022, 42(6): 1116-9.

1565 Jingwen Meng, Chen Xu, Caiju Li, et al. Literature analysis of exercise regimens in maintenance hemodialysis patients on dialysis [J]. International Journal of Transplantation and Blood Purification, 2022, 20(3): 36-42.

1566 Niu Fukun, Hu Fanling, Guo Shuxia, et al. Effects of hyperbaric oxygen therapy on the efficacy and quality of life of peritoneal dialysis patients with cognitive dysfunction [J]. Chinese Journal of Nautical Medicine and Hyperbaric Medicine, 2022, 29(5): 665-70.

1567 Niu Tieming, Luan Xunfei, Dong Qingze, et al. Effects of aerobic exercise combined with resistance training on motor function and factors related to cardiovascular events in peritoneal dialysis patients [J]. Chinese Journal of Physical Medicine and Rehabilitation, 2022, 44(6): 540-2.

1568 Ouyang Xumei, Zhou Qiaoyun, Li Jingshi, et al. Effects of health behavior management on maintaining somatic and psychological functioning of hemodialysis patients [J]. Jilin Medical Science, 2022, 43(12): 3356-8.

1569 Pan Qin, Hao Yan, Liu Ping, et al. Effects of multimodal exercise on symptom clusters of fatigue-negative mood-sleep disorder in maintenance hemodialysis patients [J]. Hebei Medicine, 2022, 44(13): 1970-3,7.

1570 Qiu Xingwen. Effects of increasing aerobic exercise on quality of life of hemodialysis patients [J]. Self-Care, 2022, 26(18): 65-7.

1571 SU Jian-Ting, ZHAO Yu-Chan, YU CHAN Z. Effect of safe heart rate-controlled aerobic exercise in the care of uremic maintenance hemodialysis patients [J]. Medical Theory and Practice, 2022, 35(1): 165-7.

1572 Su Tingting. Exercise management combined with paricalcitol on calcium and phosphorus metabolism and FGF23/Klotho axis in patients with MHD combined with SHPT [D]; Dalian Medical University, Internal Medicine, 2022.

1573 Su Xiaolian, Ai Lingyan, Duan Yinfeng, et al. Effects of aerobic exercise on resting energy metabolism, PEW status, and cardiopulmonary endurance in maintenance hemodialysis patients [J]. Dialysis and Artificial Organs, 2022, 33(2): 96-101.

1574 Sun W, Jia ZJ, Liu XH, et al. Effect of acupuncture on twelve meridian primary points in treating hemodialysis-associated hypotension and its effect on vascular endothelial function [J]. Modern Journal of Integrative Medicine, 2022, 31(18): 2555-8.

1575 QIN Xun, WANG Xiaoyu, HUANG Cheng, et al. Clinical application of Chinese herbal kidney failure formula combined with colonic dialysis in the treatment of chronic kidney disease [J]. Health Care Literature, 2022, 23(17): 236-40.

1576 Tan, Chao, Wu, Yun, He, Guizhen, et al. Clinical application of potassium-containing drugs by oxygen nebulization inhalation in correcting low potassium in peritoneal dialysis patients [J]. Primary Medical Forum, 2022, 26(18): 7-9.

1577 TANG Qi, ZHANG Liming, HU Xiaohua, et al. Effects of sodium thiosulfate combined with hemoperfusion on intractable pruritus and oxidative stress in maintenance hemodialysis patients [J]. China Blood Purification, 2022, 21(1): 33-7,47.

1578 TAO Chenghui, LIU Yang, RAN Jialu, et al. Application value of cardiac rehabilitation exercise in uremic maintenance hemodialysis patients [J]. Health| Health Essentials, 2022, (34): 27-8.

1579 TIAN Chaoyang, WEI Xuquan, KONG Lingxin, et al. Observations on the therapeutic effects of Kidney Protecting and Blood Activating Patch on the exercise endurance and Chinese medicine symptoms of maintenance hemodialysis patients with spleen and kidney yang deficiency and blood stasis [J]. Sichuan Traditional Chinese Medicine, 2022, 40(1): 128-31.

1580 Tong L, Li Yucui, Li Yang, et al. The effect of exercise education on hypotension in dialysis patients based on the Knowing, Believing and Doing model [J]. Hebei Medicine, 2022, 44(4): 532-5.

1581 Wan Niu, Tong Hui, Wang Lisheng, et al. Summary of the best evidence for exercise management programs for maintenance hemodialysis patients [J]. Evidence-Based Nursing, 2022, 8(13): 1719-24.

1582 Wang D. Development of fish skin collagen-chitosan-pectin scaffolds and their application in human umbilical cord mesenchymal stem cell culture and skin repair [D]; Huazhong Agricultural University, Bioengineering, 2022.

1583 Wang Fanchuan. Effectiveness of continuous hemodialysis in the treatment of acute renal failure [J]. Chinese and Foreign Medicine Research, 2022, 20(25): 148-51.

1584 Wang F, Yu K, Wei W, et al. A meta-analysis of the effects of long-chain n-3 polyunsaturated fatty acids on muscle weight and muscle strength in randomized controlled clinical studies [J]. Chinese Journal of Clinical Nutrition, 2022, 30(4): 214-26.

1585 Wang Li. Clinical effects of 5E rehabilitation model in improving sleep quality and nursing care satisfaction of uremic hemodialysis patients [J]. World Journal of Sleep Medicine, 2022, 9(7): 1305-8.

1586 Wang L. Biological effects of self-assembled responsive dual drug-carrying nanoparticles on periodontitis in diabetic rats [D]; Chongqing Medical University, Stomatology; Oral Clinical Medicine, 2022.

1587 Wang Minxia, Wang Xu, Xu Jiayun, et al. Analysis of the role of continuous blood purification in the treatment of acute renal failure combined with ketoacidosis in diabetic nephropathy [J]. Journal of Rare Diseases, 2022, 29(3): 63-5.

1588 Wang Rong. Impact of health education and quality nursing care on hemodialysis care for elderly patients with diabetic nephropathy [J]. Chinese and Foreign Women's Health Research, 2022, (17): 156-8.

1589 Wang Tingting, Chen Xiangjiao, Liang Yanjuan, et al. Effects of low-intensity aerobic rehabilitation exercise on psychological status and lower limb motor function of uremic hemodialysis patients [J]. Jilin Medical Science, 2022, 43(2): 565-6.

1590 Wang, V.. Adipose mesenchymal stem cell-derived exosomes for the treatment of postrenal acute kidney injury in cats [D]; Northwest A&F University, Veterinary Medicine, 2022.

1591 WANG Weiping, JIANG Lu, ZHANG Qiang, et al. Effects of exercise rehabilitation guided by cardiopulmonary exercise test on immune function and fatigue level of hemodialysis patients [J]. Modern Journal of Integrative Medicine, 2022, 31(10): 1337-41,427.

1592 WANG Xianfang, LIU Junhui, JUNHUI L I U. Effectiveness of Ba Duan Jin applied to patients on maintenance hemodialysis [J]. Chinese and Western Medicine Nursing (in Chinese and English), 2022, 8(12): 41-4.

1593 Wang X. Effect of hemoperfusion combined with hemodialysis in the treatment of patients with diabetic nephropathy and its effect on inflammatory factors, insulin resistance, and malnutrition status [J]. Diabetes New World, 2022, 25(14): 26-9.

1594 Wang Yanxia. Effects of Chinese medicine enema on adverse reactions such as constipation, loss of appetite and vomiting in uremic hemodialysis patients [J]. Medical Information, 2022, 35(17): 88-90,4.

1595 WU Jiaqi, LIU Lingfeng, MAO Linbo, et al. Application and effect of Tai Chi Softball Exercise in maintenance hemodialysis patients [J]. World Digest of Current Medical Information, 2022, 22(23): 29-34.

1596 WU Juntao, QIU Juanjuan, LIN Cuiyun, et al. The effect of tai chi exercise combined with acupressure on the fatigue status and sleep quality of hemodialysis patients [J]. Contemporary Nurses (Zhongdian), 2022, 29(8): 72-4.

1597 WU T, SUN Q, QI S. Observations on the Application Effect of Hierarchical Correspondence Management in Hemodialysis Patients [J]. Contemporary Nurses (Lower Decade), 2022, 29(1): 121-3.

1598 Wu Xiaohong. Application of full health education model in improving standardized test compliance care for hemodialysis patients [J]. Health Care World, 2022, (22): 268-9.

1599 Wu Yuchi. Exploring the mechanism of rhubarb enema in the treatment of chronic kidney disease based on the pathogenesis of damp-heat and toxicity and oxidized trimethylamine [D]; Guangzhou University of Traditional Chinese Medicine, TCM Internal Medicine, 2022.

1600 WU Yun, TAN Shao, HE Guizhen, et al. Effect of clinical application of potassium-containing drugs by oxygen nebulization inhalation in correcting low potassium in peritoneal dialysis patients [J]. China Medical Innovation, 2022, 19(17): 9-13.

1601 Wu Hao, Xu Fangfang, Tao Ling, et al. Effect of aerobic combined stretching exercise in patients with hemodialysis combined with restless legs syndrome [J]. China Medical Innovation, 2022, 19(27): 108-12.

1602 Xia Dan, Tong Hui, Wang Lisheng, et al. Summary of the best evidence on exercise management programs for peritoneal dialysis patients [J]. China Clinical Nursing, 2022, 14(11): 700-6.

1603 XIA Jinghua, ZHU Wenbo, SONG Dan, et al. Application effect of three-party empowerment combined with Zhixinxing health education model in exercise intervention for maintenance hemodialysis patients [J]. Guangxi Medicine, 2022, 44(17): 2065-8.

1604 XIA Min, CHEN Fu-Xing, FU-XING C. Application value of bicycle exercise therapy and gabapentin in the treatment of hemodialysis patients with restless legs syndrome [J]. Chinese Family Medicine, 2022, 20(5): 793-5,807.

1605 Xie, A. W.. Effects of hemodialysis and peritoneal dialysis on lipids, oxidative stress and inflammatory factors in patients with end-stage renal disease [J]. Chinese and Foreign Medicine, 2022, 41(29): 76-9,84.

1606 Xie Lijuan, Shi Suhua, You Gengji, et al. Effect of aerobic exercise care in maintenance hemodialysis patients [J]. Zhongguo Nankang Med, 2022, 34(13): 190-2.

1607 Xiong Jiuhong, Yang Qian, Li Na, et al. Observations on the effect of self-proposed solidifying and expelling turbid formula in the adjuvant treatment of hyperphosphatemia in patients undergoing maintenance hemodialysis [J]. Guangxi Medicine, 2022, 44(5): 521-5,38.

1608 Xiong Qianqian. Accumulation of the intestinal toxin oxidized trimethylamine in peritoneal dialysis patients and the regulatory role of prebiotics [D]; Huazhong University of Science and Technology, Nutrition and Food Hygiene, 2022.

1609 Xiong Changqing, Wang Weiping, Jia Xiaojun, et al. Effects of aerobic exercise on oxidative stress and quality of survival in maintenance hemodialysis patients [J]. Contemporary Medicine, 2022, 20(23): 36-8.

1610 Xu Xiaofang. Effects of power orientation therapy on psychological stress and treatment adherence in hemodialysis patients with diabetic nephropathy [J]. Systemic Medicine, 2022, 7(17): 106-9.

1611 Xu Lixuan, Liu Jianjing, Xu Yingyin, et al. Meta-analysis of the effectiveness of Baduanjin exercise in improving sleep quality of maintenance hemodialysis patients [J]. Primary Chinese Medicine, 2022, 1(1): 57-63.

1612 Xu M, Gong LF, Xu W, et al. Analysis of the application effect of bupivacaine lumbar anesthesia for very low level peritoneal dialysis placement in patients with chronic kidney disease stage 5 [J]. Clinical Medicine Literature Electronic Journal, 2022, 9(52): 34-7.

1613 Xue WJ, Wu Han, Liu F, et al. Application of rehabilitation nursing based on HACCP principles combined with exercise therapy in maintenance hemodialysis patients [J]. China Medical Journal, 2022, 19(33): 151-4.

1614 YAN Xing, ZHAO Qiaohong, PENG Yaoli, et al. Effects of progressive resistance exercise on exercise capacity, nutritional indicators and sleep quality of maintenance hemodialysis patients [J]. Evidence-Based Nursing, 2022, 8(9): 1215-9.

1615 Yang HL. Effect of early mechanical ventilation on vital signs and blood gas indexes in elderly patients with uremia combined with severe acute left heart failure [J]. Modern Medicine and Health Research (Electronic Edition), 2022, 6(2): 69-72.

1616 YANG Yajing, ZHENG Na, NA Z. A study on the application of health education of time-sensitive incentive theory in weight management of maintenance hemodialysis patients [J]. China Health Education, 2022, 38(4): 367-70.

1617 YE Dan, ZHENG Na, ZHAO Lingyu, et al. The effects of continuity of care with aerobic exercise on sleep quality and symptoms in maintenance hemodialysis patients [J]. Journal of Naval Medicine, 2022, 43(6): 607-10.

1618 Ye Xiaoshang, Jiang Luyue, Jun Chenxia, et al. Effects of aerobic-resistance exercise on physiological function and quality of life of elderly hemodialysis patients [J]. Chinese Journal of Gerontology, 2022, 42(6): 1399-402.

1619 ZHAN H B, SHI L D, LIDAN S H I. Effects of levocanidin on inflammatory factors, oxidative stress and cardioprotective effects in peritoneal dialysis rats with chronic renal failure [J]. Northern Pharmacology, 2022, 19(2): 15-7,20.

1620 Zhang B, Dai HN, Hu YY, et al. Effects of different hemofiltration modalities combined with hemoperfusion on myocardial enzyme profiles, antioxidant capacity, and renal function in patients with bee stings combined with multiple organ dysfunction syndrome [J]. International Journal of Transplantation and Blood Purification, 2022, 20(2): 31-4.

1621 Zhang Fan, Liao Jing, Zhang Weihong, et al. Evaluation of the application effect of eight-duanjin exercise in peritoneal dialysis patients [J]. Shanghai Nursing Management, 2022, 22(8): 34-7.

1622 Zhang Jiao Na, Wang At Han, Guo Bing Bing, et al. Effects of intensive psychological care on health knowledge cognition and negative emotions of hemodialysis patients [J]. China Primary Care Medicine, 2022, 29(8): 1255-8.

1623 Zhang Lijun, Wang Rong, Zhang Feng, et al. Effects of levocanidin injection on microinflammatory status, malnutrition and oxidative stress in maintenance hemodialysis patients [J]. Shaanxi Medical Journal, 2022, 51(8): 1004-7.

1624 Zhang Li-Na, Chen Jin-Yan, Zhang Jing-Li, et al. Effects of hemoperfusion tandem hemodialysis on liver, kidney and lung target organ damage in patients with acute paraquat poisoning [J]. International Journal of Transplantation and Blood Purification, 2022, 20(3): 29-32.

1625 Zhang Qian. The efficacy of HIF-PHI in treating renal anemia in patients on maintenance hemodialysis in Xining [D]; Qinghai University, Internal Medicine (Nephrology), 2022.

1626 Zhang W, Wang H, Huan Xuelai, et al. Study of aerobic exercise in dialysis on the occurrence of IDH in hemodialysis patients [J]. Practical Clinical Nursing Electronic Journal, 2022, 7(38): 60-2,59.

1627 Zhang Xiangfang. Effects of hemoperfusion combined with sequential dialysis for the treatment of end-stage diabetic nephropathy in the elderly on patients' serum sTNFER Ⅰ, sTNFER Ⅱ levels and insulin resistance [J]. Pharmaceutical Biotechnology, 2022, 29(5): 489-93.

1628 Zhang Yayi, Li Chunxi, Huang Xiaoling, et al. Effectiveness of Exercise Intervention Applied to Prevent Symptomatic Hypotension During Hemodialysis [J]. Primary Medical Forum, 2022, 26(21): 1-3.

1629 Zhang Yuanli, Liu Yinglian, Lin Zicheng, et al. Effects of exercise management guided by traditional Chinese medicine exercise theory on dialysis adequacy and microinflammatory status in MHD patients [J]. Chinese Journal of Gerontology, 2022, 42(17): 4210-4.

1630 ZHANG Zhi-Qin, YANG Xue-Hua, XUE HUA Y. Effects of different iron supplementation modalities on anemia-related and microinflammation indexes in maintenance hemodialysis patients based on pharmacological supervision [J]. International Journal of Transplantation and Blood Purification, 2022, 20(2): 22-5.

1631 ZHAO Chen, YAN Li, LI Y A N. Analysis of the application value of exercise intervention therapy in hemodialysis patients based on multidisciplinary collaboration [J]. Xinjiang Medicine, 2022, 53(6): 711-4.

1632 Zhao Jiale. Role of CD38 and its mechanism in diabetes-induced kidney injury [D]; Nanchang University of Medicine, Translational Medicine, 2022.

1633 Zheng, Danping. T-BTO@PEG@Ce6 nanomaterials combined with piezoelectric acoustic kinetic therapy for laryngeal cancer [D]; Jiangsu University, Otolaryngology, 2022.

1634 Zheng Weifang, Zhang Yuezhen, Zheng Shufang, et al. The effect of food intake time on patients' complications during hemodialysis [J]. Chinese Rural Medicine, 2022, 29(4): 67-8.

1635 Zhong Liang, Zeng Yuchun, Tang Xueyu, et al. Effects of Green's model intervention for maintenance hemodialysis on patients' compliance behavior, health perception and complications [J]. Chinese Journal of Health Care Medicine, 2022, 24(2): 128-31.

1636 Zhou Piao Piao. Effectiveness of continuous veno-venous hemofiltration dialysis in treating acute renal failure in children [J]. China Maternal and Child Health Care, 2022, 37(14): 2578-81.

1637 Zhou Q. Clinical and mechanistic study of the effect of molecular hydrogen on ultrafiltration function in peritoneal dialysis patients [D]; Chinese People's Liberation Army Naval Military Medical University; Naval Military Medical University, Internal Medicine (Nephrology), 2022.

1638 ZHU Dan, LI Lianjiang, LIANJIANG L I. Effect of functional exercise training in the treatment of patients with chronic kidney disease [J]. Chinese Medical Science, 2022, 12(17): 9-13.

1639 Zhu Li-Fang. Effect of incremental resistance exercise in hemodialysis applied to maintenance hemodialysis patients [J]. Dialysis and Artificial Organs, 2022, 33(3): 85-8.

1640 Zuo Chuanlong, Jia Xinlei, Xing Runlei, et al. Net Meta-analysis of the Effect of Non-pharmacological Interventions on Depression in Renal Dialysis Patients [J]. China Nursing Management, 2022, 22(1): 88-95.

1641 Zuo Liyan. Effectiveness of collaborative exercise therapy management based on multidisciplinary diagnosis and treatment applied to maintenance hemodialysis patients [J]. Dialysis and Artificial Organs, 2022, 33(3): 81-4.

1642 Ao Yunong. Preparation and functional evaluation of articular cartilage bionic matrix hydrogel [D]; Army Medical University, Surgery (Orthopedics), 2023.

1643 Bi Xueqing, Wang Lihua, Jia Lan, et al. Efficacy of hypoxia-inducible factor prolyl hydroxylase inhibitor on anemia in erythropoietin-resistant maintenance hemodialysis patients [J]. Chinese Journal of Practical Internal Medicine, 2023, 43(1): 58-62.

1644 Cao Peiye, Zhao Huiping, Wu Bei, et al. Summary of the best evidence for the management of adult peritoneal dialysis patients [J]. China Blood Purification, 2023, 22(7): 551-6.

1645 ZENG Fei, WANG Xiaosheng, XIAO SHENG W. Impact of self-management based on WeChat punch card supervision on the effect of exercise intervention in maintenance hemodialysis patients [J]. International Journal of Nursing, 2023, 42(24): 4596-600.

1646 ZENG Jian-Fen, DING Xiao-Ying, XIAO YING D. Nursing Strategies and Patient Response Assessment in ARNI-Treated Dialysis Patients with Combined HFpEF [J]. Journal of Chinese and Foreign Medical Research, 2023, 2(12): 13-5.

1647 Zeng Shuqiong, Cha Wenhao, Zhang Yu, et al. Bilateral rectus abdominis muscle sheath block and transversus abdominis muscle plane block combined with remazolam benzenesulfonate for peritoneal dialysis catheterization with cardiovascular disease [J]. Clinical Medicine Practice, 2023, 32(2): 90-2.

1648 ZENG Yanhua, DING Xiaoying, XIAO YING D. Evaluation of the effect of ARNI treatment on wound healing and nursing strategies in dialysis patients with combined HFpEF [J]. Medical Forum, 2023, 5(19): 7-9.

1649 Chang YJ. The effect of tele-health education combined with traditional Chinese medicine nursing on hemodialysis patients [J]. China Urban and Rural Enterprise Health, 2023, 38(7): 173-5.

1650 Chen Feifei. Effects of CPET-based rehabilitation exercise on immune function and fatigue in MHD patients [J]. Guangzhou Medicine, 2023, 54(10): 101-5.

1651 Chen Huiping, Kong Xiaoli, Chen Ning, et al. Effects of eight-duanjin exercise combined with traditional Chinese medicine dietary therapy intervention on volume load, nutrition and fatigue status of maintenance peritoneal dialysis patients [J]. Qilu Nursing Journal, 2023, 29(17): 57-60.

1652 Chen Jinrong, Chen Yayan, He Liping, et al. Analysis of the effects of individualized exercise therapy combined with nursing intervention on sleep quality and quality of life of hemodialysis patients [J]. World Journal of Sleep Medicine, 2023, 10(4): 923-5,9.

1653 Chen Liyan, Wang Menghuan, Li Wenwen, et al. Clinical observation of transcranial direct current stimulation to improve attention and executive ability of maintenance hemodialysis patients [J]. Chinese Journal of Rehabilitation Medicine, 2023, 38(9): 1221-6.

1654 Chen, Qiuqi anonymous. An applied study of pre-dialysis cardiovascular color ultrasound assessment in patients with end-stage renal failure [D]; University of Electronic Science and Technology, Ultrasound Medicine, 2023.

1655 Chen Shudian, Chen Wancong, Liu Shaoqin, et al. Observations on the effects of TTM-based oriented interventions on self-management behaviors and hyperkalemia in maintenance hemodialysis patients [J]. Life Science Instrumentation, 2023, 21(z1): 36.

1656 Chen Shuangru, Cui Wenfang, Lv Huali, et al. Effects of individualized exercise therapy care on negative emotions and sleep quality in maintenance hemodialysis patients [J]. World Journal of Sleep Medicine, 2023, 10(10): 2482-4.

1657 CHENG Lan, ZHANG Yujie, XING Binnan, et al. Clinical Study on the Treatment of Renal Anemia in Non-Dialysis Patients with Chronic Kidney Disease by Combination of Tranquilizing the Spleen and Promoting Blood Tablet and Roxarestat [J]. Modern Drugs and Clinics, 2023, 38(8): 2031-5.

1658 CHENG Xiu Jin, LAN Jing, ZHANG Ming Ming, et al. Effects of valsartan combined with levocanidin on cardiac and renal function in patients with chronic renal failure accompanied by heart failure and receiving hemodialysis [J]. Da Dao Doctors, 2023, 8(22): 16-8.

1659 Chu Mingyue, Deng Beibei, Meng Lingling, et al. Clinical efficacy of Angelica sinensis blood replenishing soup in the adjuvant treatment of patients with chronic renal failure and its effect on renal function and oxidative stress level [J]. World Journal of Integrative Medicine, 2023, 18(12): 2473-7.

1660 Zhai Wanchun, Chen Yu, Chen Li, et al. A clinical study of aerobic exercise combined with resistance exercise training to improve the debilitating condition of elderly hemodialysis patients [J]. Healthy Women, 2023, (41): 29-30.

1661 Zhai Wanchun, Wu Shumin, Mo Yangping, et al. Effects of nutritional support combined with exercise training on the debilitating condition of hemodialysis patients [J]. China Health Standard Management, 2023, 14(15): 187-90.

1662 Ding Lei. Preparation of reduction-responsive lenvatinib nanomedicine and its application in the treatment of hepatocellular carcinoma [D]; Jilin University, Surgery, 2023.

1663 Dong Bo, Kong Min, Song Ying, et al. Effects of tianphenin on the 5-hydroxytryptamine system and related mechanisms in rats modeled with hyperactive tic disorder in children [J]. Shandong Medicine, 2023, 63(16): 5-8.

1664 Dong Hanzhi. Effects of Kidney Impotence Formula on Nutritional Status of Non-Dialysis Patients with Chronic Kidney Disease [D]; Chengdu Medical College, Internal Medicine, 2023.

1665 Dong Yangcui, Li Suhua, Ma Qin, et al. Application of specialized contracted follow-up management in patients with diabetic nephropathy [J]. Zhong Chinese Journal of Modern Nursing, 2023, 29(25): 3476-81.

1666 Fan Huanxin. Impact of nutritional management combined with aerobic exercise on the nursing outcomes of elderly patients on maintenance hemodialysis [J]. Evidence-Based Nursing, 2023, 9(9): 1701-4.

1667 Fan Meirong. Design and application of rehabilitation exercises for young and middle-aged peritoneal dialysis patients [D]; Jiangsu University, Nursing, 2023.

1668 FANG Meng, DAI Min, YANG Wenjuan, et al. Effects of aerobic combined with resistance exercise on elderly maintenance hemodialysis patients with obese sarcopenia [J]. Journal of Nursing, 2023, 38(5): 95-100.

1669 Gao, L., Cai, H. Y., Liu, T. W., et al. Effect of selenium on peritoneal dialysis-associated peritoneal fibrosis induced by high glucose in rats [J]. Journal of China Medical University, 2023, 52(1): 46-50.

1670 Gao YM. Effect of high-throughput hemodialysis combined with EPO on renal anemia and microinflammatory status in MHD patients [J]. Modern Diagnosis and Treatment, 2023, 34(19): 2856-8,87.

1671 GE Shaoqin, YANG Bo, BO Y. Construction of a whole management program based on the lean quality management model and its application in hemodialysis patients with diabetic nephropathy [J]. Medical Theory and Practice, 2023, 36(18): 3202-5.

1672 Ge Xing. Effects of aerobic exercise on cognitive function, daytime somnolence and non-high-density lipoprotein cholesterol in maintenance hemodialysis patients [D]; Anhui Medical University, Internal Medicine (Nephrology), 2023.

1673 GE X, LIU YM, YONG MEI L. Effects of aerobic exercise on cognitive function, daytime somnolence, and non-high-density lipoprotein cholesterol in maintenance hemodialysis patients [J]. World Digest of Recent Medical Information, 2023, 23(37): 162-6.

1674 Ge Yuan, Yang Slowly, Jin Ru, et al. Effects of low-intensity aerobic rehabilitation exercise on psychological elasticity, lower limb motor function and bone metabolism level in maintenance hemodialysis patients with diabetic nephropathy [J]. International Journal of Transplantation and Blood Purification, 2023, 21(1): 34-6.

1675 GU Tong, ZHANG Jianmei, LI Yaguang, et al. Observation of the efficacy of different intravenous iron dosages on maintenance hemodialysis patients and survival analysis [J]. Journal of Clinical Nephrology, 2023, 23(7): 563-8.

1676 GUO Chuanqi, AI Jian, JIAN A I. Study on the therapeutic effect of 73 cases of chronic renal failure treated with Wen Kidney Drainage Turbid Soup [J]. Shaanxi Traditional Chinese Medicine, 2023, 44(9): 1237-40.

1677 GUO Erwei, LI Yanbin, ZHANG Tongjing, et al. Efficacy of Angelica sinensis blood tonifying soup plus flavor combined with western medicines in treating renal anemia on maintenance hemodialysis and its effect on iron metabolism indexes and microinflammatory response [J]. Chinese Medicine Research, 2023, 36(6): 34-8.

1678 GUO JILEI, LI FENG RONG, FENG RONG L. Study on the effect of leucovorin on vascular sclerosis and vascular endothelial function in chronic renal failure patients on maintenance hemodialysis [J]. Chinese Journal of Continuing Medical Education, 2023, 46(10): 909-13.

1679 Guo Liping. Efficacy analysis of the effect of motivational interviewing on cognition and adherence to exercise rehabilitation in MHD patients [J]. Clinical Nursing Research, 2023, 32(4): 138-40.

1680 Guo Yueyue, Bo Xiangmin, Liu Shengfeng, et al. Application of Fitness Qigong Five Animal Play in Exercise Rehabilitation of Dialysis Patients with Myasthenia Gravis [J]. Journal of Nurse Advancement, 2023, 38(20): 1865-9.

1681 Han Huanmei. Effect of aerobic exercise on maintenance hemodialysis patients [J]. Maternal and Child Nursing, 2023, 3(14): 3423-4.

1682 HAN Mei, HUANG Jihong, XU Bo, et al. Effects of cyclic resistance training combined with rational diet on the quality of survival of hemodialysis patients with sarcopenia [J]. Journal of Naval Medicine, 2023, 44(7): 706-10.

1683 HAN Meixiang, HUANG Yiqi, SHEN Weigang, et al. Clinical study on the treatment of erectile dysfunction in peritoneal dialysis patients by combining Shao Abdominal Blood Stasis Expelling Tang with low-dose tadalafil [J]. New Chinese Medicine, 2023, 55(17): 67-71.

1684 Han WY. Effects of levocanidin combined with roxarestat on iron metabolism and oxidative stress levels in patients with renal anemia caused by MHD [J]. Medical Theory and Practice, 2023, 36(3): 437-9.

1685 He L. Evaluation of the effect of continuous renal replacement therapy in the treatment of acute renal failure caused by infectious shock [J]. Shanghai Medicine, 2023, 44(22): 25-9.

1686 He Q. Microfluidic chip method for the preparation of josunin micelles and anti-hyperuricemia study [D]; Jiangsu University, Pharmacy, 2023.

1687 He Xiuli, Han Qinghui, Song Na, et al. Impact of an intervention program based on ITHBC theory on weight management in maintenance hemodialysis patients [J]. Evidence-Based Nursing, 2023, 9(19): 3570-3.

1688 He Yanping. Impact of intermittent exercise care in dialysis on the incidence of hypotension and quality of life of maintenance hemodialysis patients. [J]. Scientific Counseling, 2023, (7): 142-4.

1689 HONG LIMEI, CHEN YA Xiu, YAXIU C. Effects of 6S-based management stage nursing application on nursing compliance and complications in hemodialysis critically ill patients [J]. China Medical Science, 2023, 13(21): 151-4,66.

1690 HOU Chunhua, CHI Jing, JING C. Effects of psychological intervention of gratitude on compliance behavior of maintenance hemodialysis patients [J]. Great Health, 2023, (13): 191-3.

1691 Hu Yachan. Effects of leucovorin combined with resistance exercise training on hypotension in hemodialysis patients [J]. Medical Theory and Practice, 2023, 36(24): 4308-9,18.

1692 Hu Yanshuo, Gao Yanjun, Chen Fang, et al. Comprehensive effect observation of home exercise management program on hemodialysis patients [J]. Chinese Journal of Nephrology, 2023, 24(6): 529-32.

1693 Huang Jingjing, Chen Jiarong, Zhang Fu, et al. Comparison of the effects of deep hypothermic arrested circulation and cisplaced cerebral perfusion in neonatal aortic constriction correction [J]. Guangxi Medicine, 2023, 45(23): 2825-30,41.

1694 Huang, S. M.. Comparative efficacy of roxarestat and recombinant human erythropoietin combined with intravenous iron in the treatment of renal anemia in hemodialysis patients with iron compliance [D]; Chongqing Medical University, Clinical Medicine; Internal Medicine, 2023.

1695 JIANG Xiaolan, LIU Bin, BIN L I U. Study on the effects of high flux hemodialysis on serum ferredoxin and hypoxia-inducible factor in maintenance dialysis patients [J]. China Blood Purification, 2023, 22(2): 81-5.

1696 Jiao Lijuan, Wang Ting, Li Yuanyuan, et al. Hemodialysis combined with hemoperfusion for the treatment of end-stage patients with chronic kidney disease and the effect on renal function in response to microinflammation [J]. Hebei Medicine, 2023, 29(11): 1832-7.

1697 Kuai Wenhao. Study on the protective effect of salvinorin B and triclopyr on skin injury by sand jellyfish toxin [D]; Chinese People's Liberation Army Naval Military Medical University; Naval Military Medical University, Dermatology and Venereology, 2023.

1698 Lan Guanhua. Preparation of muscimol-encapsulated tissue-adhesive hydrogel and its promotion of diabetic wound repair [D]; Nanchang University School of Medicine, Surgery, 2023.

1699 LAI Weichong, FANG Sheng, SHENG F. Observation on the clinical effect of hemoperfusion combined with continuous hemodialysis filtration in the treatment of patients with acute severe organophosphorus poisoning [J]. Modern Medicine and Health Research (Electronic Version), 2023, 7(12): 76-8.

1700 Li Guangyao. Effects of recombinant human erythropoietin combined with reduced glutathione on anemia indexes and oxidative stress in patients with uremic anemia [J]. Medical Information, 2023, 36(19): 156-9.

1701 Li Hongli, Wang Yan, Niu Xiuru, et al. Intervention Study of Individualized Exercise on Survival Quality of Elderly Maintenance Hemodialysis Patients [J]. Primary Chinese Medicine, 2023, 2(8): 58-63.

1702 Li JQ, Yang Q, Yuan DL, et al. Meta-analysis of the efficacy and safety of roxarestat in the treatment of renal anemia in hemodialysis patients [J]. Chinese Family Medicine, 2023, 26(6): 704-10.

1703 Li JL, Liu SH, Xu Y, et al. The value of echocardiography based on Lasso-Cox analysis in the risk evaluation of cardiovascular adverse events in end-stage renal disease [J]. Chinese Journal of Biomedical Engineering, 2023, 29(4): 395-403.

1704 Li Jing, Fan Xing, Wang Qian, et al. Efficacy and safety of roxarestat in the treatment of diabetic nephropathy complicated by renal anemia [J]. Chinese Journal of New Drugs and Clinics, 2023, 42(5): 305-10.

1705 Li Jing, Liao Jian-so, Liu Xiu-ying, et al. Effects of personalized exercise combined with targeted nursing intervention on uremic hemodialysis patients [J]. Chinese Community Physician, 2023, 39(6): 126-8,31.

1706 Li Juanjuan, Zhang Rui, Shi Juan, et al. Effect of roxarestat combined with polysaccharide iron complex capsule in the treatment of renal anemia in hemodialysis patients and the effect on oxidative stress [J]. Clinical Medicine Research and Practice, 2023, 8(29): 33-6.

1707 LI JUAN YING, LI JING, JING L I. Effects of risk-graded care on the function of internal fistula in maintenance hemodialysis patients [J]. Clinical Medicine Research and Practice, 2023, 8(9): 143-5.

1708 Li Jun, Lin Li, Wang Jianmei, et al. Protective effect of Astragalus Angelicae Paste on autologous arteriovenous endovascular fistulae in maintenance hemodialysis patients [J]. Chinese Electronic Journal of Nephrology, 2023, 12(2): 87-92.

1709 LI PINGPING, LIU YUNQI, YUN QI L. A study on the effect of aerobic exercise on anxiety and depression in patients with end-stage renal disease on maintenance hemodialysis [J]. Frontiers of Medicine, 2023, 13(32): 15-7.

1710 Li Wanlin, Yun Jie, He Ling, et al. Baye s net meta-analysis of the effect of different exercise therapies on fatigue intervention in maintenance hemodialysis patients [J]. Modern Medicine and Health, 2023, 39(24): 4215-24,31.

1711 Li Ye, Wei Huajuan, Wang Haibo, et al. Clinical study on the treatment of hemodialysis-associated epilepsy with oxcarbazepine combined with the addition and subtraction of Definitive Epilepsy Formula [J]. World Journal of Integrative Medicine, 2023, 18(9): 1836-41.

1712 Li Y. Analysis of factors affecting cardiovascular prognosis of hemodialysis patients [D]; Southern Medical University, Internal Medicine (Nephrology), 2023.

1713 Chestnut L. Effects of different peritoneal dialysis modalities on renal function and inflammatory response in patients with end-stage renal disease [J]. Practical Chinese and Western Medicine Clinic, 2023, 23(9): 18-20,7.

1714 Liang Chen. Effects of home-based Otago exercise on fall efficacy in elderly maintenance hemodialysis patients [D]; Jiangsu University, Nursing, 2023.

1715 Liang Li. Analysis of the impact of rehabilitation exercise nursing on the quality of life of elderly hemodialysis patients [J]. Self-Care, 2023, 27(7): 219-21.

1716 LEONG Y M, SHEN Y, YAN S. Effect of continuity of care of exercise in dialysis on MHD patients [J]. Corps Medicine, 2023, 21(2): 75-7.

1717 LIN Haixue, SHENG Qilin, ZHU Beixia, et al. Summary of the best evidence on home exercise rehabilitation for peritoneal dialysis patients [J]. Chinese Journal of Modern Nursing, 2023, 29(9): 1176-81.

1718 Lin Y. Effects of a moxibustion device combined with pedicab exercise in dialysis in patients with uremia complicated by restless leg syndrome [J]. Health| Health Must Read, 2023, (2): 193-4.

1719 LIU Bi-Qiong, CHEN Guo-Wei, LIN Zhi-Dan, et al. Effects of hemodialysis combined with hemoperfusion on serum calcium and phosphorus levels and renal function in patients with end-stage renal disease [J]. Chinese and Foreign Medicine, 2023, 42(28): 26-30.

1720 Liu J, Zhu XL, Chen XX, et al. Effects of exercise rehabilitation on peripheral nerve function and fatigue status in maintenance hemodialysis patients [J]. Journal of Modern Electrophysiology, 2023, 30(2): 81-4.

1721 Liu Jun. Application effect of digital five-step management method in maintenance hemodialysis patients [J]. Chinese Contemporary Medicine, 2023, 30(34): 192-6.

1722 Liu Lei, Lei Jiangshu, Chen Ying, et al. Effects of Tai Chi exercise on life treatment of uremic renal bone disease [J]. Special Health, 2023, (17): 13-4.

1723 Liu Ling. Effects of individualized exercise therapy care on sleep and quality of life of maintenance hemodialysis patients [J]. Rehabilitation, 2023, (9): 90-1,4.

1724 LIU SHENG FENG, ZOU XUE QIN, XUE QIN Z. Intensive health education for maintenance hemodialysis patients' nursing care on the reduction of complication rate [J]. Medical Therapy and Health, 2023, 21(18): 64-7.

1725 LIU Weifen, SHI Suhua, LIN Min, et al. Study on the effect of anticipatory care on reducing hypoglycemia in hemodialysis patients with diabetic nephropathy [J]. Diabetes New World, 2023, 26(4): 132-5.

1726 LIU SATELLITE, ZHANG SHU, SHU Z. Clinical effect of polysaccharide iron complex combined with leucovorin in the treatment of patients with renal anemia on maintenance hemodialysis [J]. Clinical Medicine Research and Practice, 2023, 8(17): 41-4,81.

1727 Liu Xiaohong. Exploring the effects of continuity of care with aerobic exercise on sleep quality and symptoms in maintenance hemodialysis patients [J]. International Nursing Research, 2023, 5(4).

1728 Liu X. The role and mechanism of Zhenwu Tang and its active ingredient paeoniflorin in modulating Nrf2 to ameliorate urotoxin-mediated endothelial injury [D]; Guangzhou University of Traditional Chinese Medicine, TCM Internal Medicine, 2023.

1729 Liu Yibing, Ding Meimei, Wang Xiaoyu, et al. Exercise intervention based on ADOPT model in hemodialysis patients [J]. Journal of Nursing, 2023, 38(13): 78-82,8.

1730 Lu Jianghong. Construction of a prediction model for early mortality risk in critically ill non-operative patients with cervical spine injury [D]; Shantou University, Orthopaedics, 2023.

1731 Lu Xianying, Hou Chaoming, Gao Jing, et al. Net Meta-analysis of the Effects of Non-pharmacological Interventions on Fatigue in Maintenance Hemodialysis Patients [J]. Chinese Nursing Education, 2023, 20(4): 467-74.

1732 Lu Y, Shen HY, Gao LY, et al. Phenethyl caffeate improves peritoneal dialysis-associated peritoneal fibrosis by alleviating oxidative stress injury through activation of the nuclear factor E2-associated factor 2/heme oxygenase 1 pathway [J]. Chinese Journal of Nephrology, 2023, 39(6): 446-55.

1733 Lu Shan, Wen Wen, Wu Xianglan, et al. Effects of exercise therapy on dialysis adequacy and psychological status of maintenance hemodialysis patients [J]. International Journal of Transplantation and Blood Purification, 2023, 21(1): 18-21.

1734 LU Minghua, TIAN Qisheng, SUN Yanmei, et al. Clinical Study on Renal Failure Ning Tablets Combined with Bailing Capsules in the Treatment of Chronic Renal Failure Hemodialysis [J]. New Chinese Medicine, 2023, 55(17): 122-7.

1735 Luo Xiju, Liu Yixiu, Deng Siyan, et al. Meta-analysis of the effect of exercise on improving dialysis adequacy and solute clearance in hemodialysis patients [J]. Journal of Nurse Advancement, 2023, 38(20): 1890-6.

1736 Ni Xue. Vitamin E treatment improves antioxidant capacity in dialysis patients: a Meta-analysis [D]; Jilin University, Clinical Medicine, 2023.

1737 PAN Guowei, LIU Guohui, GUO HUI L. Effects of different hemodialysis modalities on the expression levels of serum hypoxia-inducible factor 1α and 2α in maintenance hemodialysis patients [J]. Longevity, 2023, (12): 3608-9.

1738 Peng Dandan. Effectiveness of progressive resistance exercise in patients with MHD combined with sarcopenia [J]. Medical Theory and Practice, 2023, 36(17): 3040-2.

1739 PENG Li-Min, LIU Key, LIU J. Observation on clinical efficacy of Bailing capsule combined with high flux hemodialysis in the treatment of uremic patients [J]. Capital Food and Medicine, 2023, 30(22): 63-5.

1740 QIQIG, ZHAO Jian-Rong, JIAN-RONG Z. Effects of aerobic combined impedance exercise on dialysis adequacy and quality of life of hemodialysis patients [J]. China Blood Purification, 2023, 22(7): 498-502.

1741 QIAO YALI, XIAO LING, LING X. Analysis of the application value of anticipatory care in the care of maintenance hemodialysis patients [J]. Longevity, 2023, (9): 2641-2.

1742 Shi Donghai. Effects of extended care with aerobic exercise on sleep quality and symptoms in maintenance hemodialysis patients [J]. Journal of Modern Nursing Medicine, 2023, 2(3).

1743 Shi, L. R.. A study on the effect of online exercise nursing instruction on fatigue and quality of life of hemodialysis patients [J]. Healthy Women, 2023, (38): 159-60.

1744 Shi Juanjuan, Xu Sa, Wang Huiping, et al. Effects of menu-based care on nutritional status, health behaviors and social support of peritoneal dialysis patients [J]. Hainan Medical Science, 2023, 34(24): 3625-30.

1745 Shu Hailin, Shi Yundi, Lu Tongan, et al. Effect of insulin pump combined with hemodialysis in the treatment of diabetic ketoacidosis [J]. Chinese and Foreign Medicine Research, 2023, 21(23): 29-32.

1746 Sun Shu, Sun Jiali, Sun Yonghui, et al. Observation on the efficacy of leucovorin combined with erythropoietin in patients with renal anemia on maintenance hemodialysis [J]. China Journal of Practical Medicine, 2023, 50(4): 94-6.

1747 QIN Jinfang, LIN Dongni, FAN Guijuan, et al. Study on the intervention of far-infrared ray therapy on early autologous arteriovenous endovascular fistula in hemodialysis patients [J]. World Abstracts of Current Medical Information (Continuous Electronic Journal), 2023, 23(59): 154-8.

1748 Tang H. Z. Finger-fist-arm movement exercise combined with video feedback education in the maturation of arteriovenous endovascular fistula in hemodialysis patients [J]. China Clinical Nursing, 2023, 15(9): 557-9.

1749 Tang Lulu. Efficacy of different starting doses of roxarestat in the treatment of renal anemia in patients undergoing maintenance hemodialysis [D]; Henan University, Internal Medicine, 2023.

1750 Tian Qiuyue, Zhang Yan, Yuan Liujie, et al. Changes of serum oxidative stress indicators in hemodialysis patients with glomerulonephritis after different nursing interventions [J]. Chinese Journal of Hemorheology, 2023, 33(2): 304-8.

1751 Tian W. Effect of individualized exercise therapy care for maintenance hemodialysis patients [J]. Chinese and Foreign Medicine Research, 2023, 2(14): 112-4.

1752 Tian Zhijuan, Xu Jun, Dai Huanhuan, et al. Effects of progressive rehabilitation exercise on respiratory function, motor function and iron metabolism in elderly hemodialysis patients [J]. Qilu Nursing Journal, 2023, 29(21): 13-6.

1753 Wang Chunying, Zhang Qian, Liao Yingchun, et al. The efficacy of total glycoside capsule of Dixanthus leaves combined with leucovorin in hemodialysis patients [J]. International Journal of Medicine and Health, 2023, 29(21): 3076-80.

1754 WANG Guirong, ZHANG Yun, YUN Z. Effects of intestinal microecological agents on intestinal barrier function in hemodialysis patients [J]. Chinese and Foreign Medicine Research, 2023, 21(15): 64-7.

1755 Wang Haiyan, Zhang Yuanyuan, Yu Man, et al. Effects of a nursing intervention targeting parents on postperitoneal dialysis complications in infants and children with prevalent heart disease and their parents' nursing knowledge [J]. Clinical Medical Engineering, 2023, 30(1): 91-2.

1756 WANG Hongcui, WANG Yuanyuan, YUANYUAN W. The value of health literacy intervention based on the Knowing, Believing and Doing model in hemodialysis patients [J]. China Medicine Herald, 2023, 20(25): 194-7.

1757 Wang Hongxia. Impact of PDCA cycle management on the quality of nursing care in blood purification unit [J]. Health Friends, 2023, (19): 233-5.

1758 Wang HZ. Study on the Improvement Role of Holistic Nursing on Negative Psychology and Quality of Life of Hemodialysis Patients [J]. Wisdom Health, 2023, 9(32): 96-9.

1759 WANG Li-Na, LIU Ke, KE L I U. Intervention effect of virtual reality-based exercise model on physical activity in peritoneal dialysis patients [J]. Nursing Research, 2023, 37(20): 3727-31.

1760 WANG Manli, WENG Yingying, ZHANG Ping, et al. Analysis of the effect of target-intensity guided aerobic exercise workout method on nutritional status of abdominal dialysis patients with chronic renal failure [J]. Chinese Contemporary Medicine, 2023, 30(16): 96-9,103.

1761 Wang Na. Mechanisms of vascular endothelial cell injury by protein-bound uremic toxins [D]; Qingdao University, Internal Medicine (Nephrology), 2023.

1762 WANG Tingting, TIAN Xing, LIU Lu, et al. Effects of progressive resistance exercise on patients with maintenance hemodialysis combined with sarcopenia [J]. International Journal of Transplantation and Blood Purification, 2023, 21(6): 41-4.

1763 WANG W, MADA, DA M. Research on the application value of high flux hemodialysis in the clinical treatment of patients with chronic renal failure in uremic stage [J]. Modern Medicine and Health Research (Electronic Edition), 2023, 7(13): 59-61.

1764 Wang Yalin. Effect of psychological care and health education in hemodialysis patients with end-stage renal disease [J]. Chinese Community Physician, 2023, 39(24): 143-5.

1765 Wang Diem, Luo Jing, Yang Jin, et al. Effects of interactive combined revisited education model on health knowledge acquisition of hemodialysis patients [J]. Clinical Medical Engineering, 2023, 30(1): 131-2.

1766 WANG Ying, FU Dongmei, LI Sa, et al. Changes of blood rheology indexes in renal failure patients after different nursing interventions [J]. Chinese Journal of Blood Rheology, 2023, 33(2): 299-303.

1767 Wei Yani, Lan Xinrong, Fan Guorong, et al. Exploring the protective effect of renal failure granules on the intestinal barrier of uremic peritoneal dialysis rats based on the intervention of aromatic hydrocarbon receptor in MLCK/pMLC signaling pathway [J]. Chinese Journal of Integrative Nephrology, 2023, 24(10): 858-61,Posterior to 1.

1768 Weng Yaping, Fei Jing, Zhu Yafei, et al. Effects of nutritional rationing combined with individualized exercise program on postmenopausal women on hemodialysis [J]. China Maternal and Child Health, 2023, 38(22): 4340-3.

1769 Wu QF, Li Z, Chang LY, et al. Meta-analysis of the effects of exercise therapy and nutritional intervention on hemodialysis patients with sarcopenia [J]. Nursing and Rehabilitation, 2023, 22(5): 24-30.

1770 Wu, Xiao. Investigation of the mechanism of CKD treatment with spleen- and kidney-enhancing formula based on fatty acid metabolic imbalance [D]; Guangzhou University of Traditional Chinese Medicine, TCM Internal Medicine, 2023.

1771 Wu Yuxuan, Li Chenghua, Fang Zhiqiu, et al. Effects of moxibustion combined with Baduanjin on sleep quality and lower limb motor function in hemodialysis patients [J]. World Abstracts of Recent Medical Information (Continuous Electronic Journal), 2023, 23(50): 204-7.

1772 WU Zengfu, HUANG Zuyi, HUANG Jingrong, et al. Effects of roxarestat combined with leucovorin on anemia indexes and oxidative stress in patients with renal anemia on maintenance hemodialysis [J]. Modern Diagnosis and Therapy, 2023, 34(14): 2102-4.

1773 Wu, Zongbi. A study on the application of needs-oriented nursing intervention in self-management of young and middle-aged hemodialysis patients [D]; Nanhua University, Nursing, 2023.

1774 WU Yunzhou, LIN Xiaomeng, WEN Xiaoyou, et al. Effects of retention enema with Drain Turbidity and Passing Vessel Formula on IS, PCS, and TMAO in maintenance hemodialysis patients [J]. Zhejiang Clinical Medicine, 2023, 25(1): 36-8.

1775 Xiao Hairong. Experimental study on the construction of a new type of nano-micellar eye drops of engegliflozin and its effectiveness in the treatment of corneal chemical injury [D]; Qingdao University of Science and Technology, Pharmacy, 2023.

1776 Xiao, Luwei. A study on the construction and application of a music-movement training program for maintenance hemodialysis debilitated patients based on a trans-theoretical model [D]; Nanhua University, Nursing, 2023.

1777 Xie Caiyun, Chen Qiongmei, Zou De'e, et al. Effects of rehabilitation exercises on quality of life and sleep quality of maintenance hemodialysis patients [J]. Chinese Geriatric Medicine, 2023, 21(6): 148-51.

1778 XU Jing, WU Xue-Rong, XUE-RONG W U. Improvement effects of home-based low-intensity physical activity on psychological status and cognition in maintenance hemodialysis patients [J]. Dialysis and Artificial Organs, 2023, 34(3): 76-9,114.

1779 Xu, S. J.. Analysis of the effects of high flux hemodialysis on oxidative stress and microinflammatory status in maintenance hemodialysis patients with diabetic nephropathy [J]. Diabetes New World, 2023, 26(4): 176-9.

1780 Xue Pei, Li Jia, Hong Meilin, et al. Clinical observation on the combination of continuous hemodialysis filtration and hemoperfusion for acute severe organophosphorus pesticide poisoning [J]. Chinese Journal of Industrial Medicine, 2023, 36(3): 218-21.

1781 YANG Nian, TAN Huiyi, WANG Zhenzhen, et al. Net Meta-analysis of the Effectiveness of Different Interventions to Improve Fatigue in Maintenance Hemodialysis Patients [J]. Modern Clinical Nursing, 2023, 22(1): 59-67.

1782 Yang Q. Effects of aerobic exercise continuum of care on sleep quality and symptoms of maintenance hemodialysis patients [J]. Journal of Modern Nursing Medicine, 2023, 2(1).

1783 Yang Xuemei, Zhang Wu, Li Wenli, et al. Effects of detoxification and kidney-stabilizing soup on renal function and serum LPO and PAI-1 in hemodialysis patients [J]. Hainan Medicine, 2023, 34(14): 1982-5.

1784 Ying XT, Xiao BB, Jia JW, et al. Effect of high flux dialysis combined with sodium thiosulfate in the treatment of uremia [J]. Huaxia Medicine, 2023, 36(6): 111-5.

1785 Yu, Mengyuan. Comparison of Lipid Peroxidation Status and Benefit of Antioxidant Therapy in Dialysis Patients-A Meta-Analysis [D]; Jilin University, Clinical Medicine, 2023.

1786 Zhang, Chi. Natural ice tablet ameliorates behavioral deficits in MPTP Parkinson's disease model mice by modulating glutamate and its transporters [D]; Anhui Medical University, Traditional Chinese Medicine, 2023.

1787 Zhang Fan, Dong Yan, Zeng Zhen, et al. Effects of prone bicycle exercise in dialysis on exercise capacity of hemodialysis patients [J]. Evidence-Based Nursing, 2023, 9(11): 2037-42.

1788 Zhang J. Effect of exercise guidance combined with intensive skin management on maintenance hemodialysis patients [J]. Qingdao Medicine and Health, 2023, 55(6): 440-3.

1789 ZHANG Jiajia, XU Yang, QIN Lulu, et al. Effects of Renal Failure Capsules on Intestinal Mucosal Barrier Function and Microinflammatory State of Maintenance Hemodialysis Patients [J]. New Chinese Medicine, 2023, 55(12): 76-80.

1790 Zhang Jingjing, Xu Yaguang, Wang Tianshu, et al. Evaluation of levocanidin with iron sucrose injection for the treatment of renal anemia complicated during maintenance hemodialysis [J]. Chinese and Foreign Medicine, 2023, 42(3): 15-8,22.

1791 Zhang M. Development and preliminary clinical application of a predictive model for chronic disease management in Chinese medicine for CKD based on decision tree technology [D]; Guangzhou University of Traditional Chinese Medicine, TCM Internal Medicine, 2023.

1792 Zhang Qiaoping, Jin Minyan, Lei Xiufen, et al. Effects of online supervision model on self-management behavior of dry weight control in MHD patients [J]. Zhejiang Clinical Medicine, 2023, 25(10): 1543-6.

1793 Zhang Xiaoyan, Lu Hao, Liu Xiaoju, et al. Effects of Fu Zheng Hua Turbid Blood Activating Tang on intestinal microecology of hemodialysis patients with chronic renal failure [J]. Liaoning Journal of Chinese Medicine, 2023, 50(5): 152-5.

1794 Zhang Xuan, Guo Yueyue, Zhou Kouxiang, et al. Evidence-based practice of exercise rehabilitation in maintenance hemodialysis patients [J]. China Blood Purification, 2023, 22(6): 472-6.

1795 ZHANG YANLIAN, ZHANG LIFANG, LI FANG Z. Nursing countermeasures to alleviate fatigue of maintenance hemodialysis patients by combining relaxation music with horizontal bicycle [J]. Dietary Health Care, 2023, (2): 81-4.

1796 ZHANG YE JUN, ZHANG YAN LIN, YAN LIN Z. Effect of Colimet on blood potassium in patients on Nosinto maintenance dialysis [J]. North Pharmacology, 2023, 20(4): 90-2.

1797 Zhang Ying. Effect of dexmedetomidine and remifentanil combined with rectus abdominis sheath nerve block in peritoneal dialysis tube implantation [J]. China Pharmacoeconomics, 2023, 18(12): 78-82.

1798 ZHANG Zhiqiu, FAN Jihui, JIHUI F A N. Efficacy of recombinant human erythropoietin combined with leucovorin in the treatment of renal anemia in patients undergoing maintenance hemodialysis and its effect on the levels of inflammatory factors and iron metabolism [J]. Hebei Medicine, 2023, 29(3): 486-91.

1799 Palm Xiaonan. Effects of hypoxia-inducible factor-prolinyl hydroxylase inhibitor treatment of renal anemia on iron metabolism in non-dialysis chronic kidney disease stage 4-5 [D]; Hebei Medical University, Internal Medicine, 2023.

1800 ZHAO Wenyang, HE Lin, LIN H. Clinical value of ginseng qi yi xin tang combined with cardiopulmonary exercise rehabilitation for chronic heart failure [J]. Rehabilitation, 2023, (7): 111-3.

1801 Zhao XY. Clinical efficacy of sacubitril valsartan in the treatment of patients with heart failure with preserved ejection fraction combined with maintenance hemodialysis [D]; Bengbu Medical College, Internal Medicine, 2023.

1802 ZHAO Xinyu, ZHANG Lizhi, LIU Li, et al. Clinical efficacy analysis of sacubitril valsartan in the treatment of patients with heart failure with preserved ejection fraction combined with maintenance hemodialysis [J]. Journal of Medical Research, 2023, 52(11): 154-8,69.

1803 Zheng L. Screening of major target genes and mechanism of action of roxarestat in the treatment of renal anemia with core prescription of traditional Chinese medicine [D]; Lanzhou University, Basic Medicine, 2023.

1804 Zhou Shengzhen, Zhang Ling, Pan Yanzhen, et al. Analysis of the treatment effect of CRRT for acute left heart failure with renal insufficiency [J]. Modern Diagnosis and Therapy, 2023, 34(14): 2185-7.

1805 ZHOU Xiaorui, LENG Yingjie, FU Xiaorong, et al. Effects of appropriate exercise training during dialysis on dialysis adequacy and fatigue in hemodialysis patients [J]. China Medical Innovation, 2023, 20(20): 102-6.

1806 Zhou YY. Analysis of the effect of exercise rehabilitation exercise on sleep quality and fatigue level of uremic hemodialysis patients [J]. Health Home, 2023, (4): 96-8.

1807 ZHOU Zhe, PING Liying, ZHENG Yang, et al. Analysis of the efficacy of the formula for clearing heat and eliminating blood stasis combined with blood purification in the treatment of acute pancreatitis [J]. Chinese Journal of Integrative Medicine and Digestion, 2023, 31(7): 567-72.

1808 Zhu Chan. Effects of Focused Empowerment Model Exercise Combined with Self-Efficacy Training on Exercise Self-Efficacy and Motor Function in Peritoneal Dialysis Patients [J]. Reflexology and Rehabilitation Medicine, 2023, 4(13): 83-6.

1809 Zhu Guo-Ying. Application of exercise program in hemodialysis care for patients with end-stage renal disease [J]. Chinese Rural Medicine, 2023, 30(24): 65-6.

1810 Zhuang Peixia. Effects of extended health guidance on blood pressure and quality of life in patients with hypertension combined with maintenance hemodialysis [J]. Cardiovascular Disease Prevention and Control Knowledge, 2023, 13(19): 35-7.

1811 Zou, Chunbo, Su, Tingting, Zhang, Shuyan, et al. Exercise management combined with paricalcitol upregulates serum Klotho protein and improves secondary hyperparathyroidism [J]. China Blood Purification, 2023, 22(2): 114-8.

1812 BIAN Baohua, LIU Hong, HONG L I U. Clinical study of renal failure granules combined with reduced glutathione in the treatment of chronic renal failure on maintenance hemodialysis [J]. Modern Drugs and Clinics, 2024, 39(3): 715-9.

1813 Cai Yi, Zhang Qiankun, Ding Jingxiang, et al. Effect of health education based on symptom management strategy in high flux hemodialysis patients [J]. China Medical Journal, 2024, 21(15): 152-5.

1814 Chai Zai Kun, Feng Dan, Aruna, et al. Exploring the central mechanism of electroacupuncture in treating depressed rats based on purinergic signaling pathway [J]. Journal of Liaoning University of Traditional Chinese Medicine, 2024, 26(4): 77-83.

1815 Shen Xinxiu, Sheng Lingfeng, Shi Yanyan, et al. Effect of hemoperfusion combined with hemodialysis in treating patients with diquat poisoning [J]. Chinese and Foreign Medicine Research, 2024, 22(9): 140-3.

1816 CHEN Caihe, MA Shuyan, HUANG Wenhui, et al. Prognostic risk factors of combined catheter-associated bloodstream infections in patients on maintenance hemodialysis [J]. Chinese Emergency Medicine for Critical Illness, 2024, 36(2): 183-8.

1817 Chen X. Analysis of the effect of leucovorin combined with intermittent exercise therapy on dialysis with hypotension in uremic patients [J]. Medical Theory and Practice, 2024, 37(11): 1964-5,70.

1818 CHENG Rui, ZHU Changcai, ZHANG CAI Z. Regulation and mechanism of peritoneal mesothelial cell nucleotide-binding oligomerization structural domain-like receptor protein 3 inflammatory vesicles by thioredoxin 1/thioredoxin interacting protein [J]. Chinese Journal of Physicians' Advancement, 2024, 47(6): 513-7.

1819 Du Wenwen. Efficacy of febuxostat combined with hemodialysis in the treatment of chronic kidney disease and its effect on inflammatory factor levels and renal function in patients. [J]. Medical Clinical Research, 2024, 41(1): 70-3,7.

1820 Fu Shuige, Yuan Xiaoqiang, Xia Xuan, et al. Analysis of the efficacy of peritoneal dialysis and hemodialysis in the treatment of end-stage renal disease in the elderly [J]. Clinical Research, 2024, 32(5): 45-8.

1821 Fu Y, Ma W, Li X, et al. Exploring the efficacy mechanism of detoxification and kidney-preserving pills in the treatment of chronic kidney disease based on intestinal-derived toxins [J]. Chinese Medicine Pharmacology and Clinics, 2024, 40(3): 98-102.

1822 Gao Gao F, Zhao Jin L, Yang Ya Qin, et al. Efficacy of roxarestat in the treatment of renal anemia with microinflammatory state on maintenance hemodialysis and its effect on serum Hepcidin, inflammatory markers and oxidative stress levels [J]. Chinese Medical Journal, 2024, 26(4): 577-81.

1823 HAN Miaomiao, TIAN Mingjie, ZHAO Haihong, et al. Analysis of the effect of incremental resistance exercise combined with nutritional therapy in patients with end-stage renal disease receiving maintenance hemodialysis [J]. Journal of Clinical and Experimental Medicine, 2024, 23(3): 265-70.

1824 He Chuan'e, Rao Yifeng, Song Zhixia, et al. Analysis of the efficacy of Bailing capsule in the treatment of early peritoneal dialysis patients [J]. Ba Chu Medicine, 2024, 7(2): 36-41.

1825 HOU Mingran, LI Zhongxin, ZHONG-XIN L I. Effects of hypoxia-inducible factor prolyl hydroxylase inhibitor on cardiovascular indices and cardiovascular and cerebrovascular complications in patients with uremic dialysis [J]. Chinese Journal of Practical Internal Medicine, 2024, 44(2): 159-63.

1826 Huang QF. Effect of exercise therapy combined with paricalcitol in the treatment of MHD patients with secondary hyperparathyroidism and its effect on their FGF23 and Klotho protein levels [J]. Reflexology and Rehabilitation Medicine, 2024, 5(3): 61-4.

1827 Jiang, Guanghui, Xu, Mingyue, Dai, Wenli, et al. Power orientation intervention combined with quantitative exercise guidance in elderly uremic maintenance hemodialysis patients [J]. Qilu Nursing Journal, 2024, 30(5): 30-4.

1828 JIANG Z, CHEN YUYU, PEI PEI C. Effect of humanistic care in hemodialysis patients with diabetic nephropathy [J]. Chinese and Foreign Medicine Research, 2024, 3(5): 138-40.

1829 LAI YAN YANG, QUAN MIN YING, MIN YING Q. Effects of bicycle exercise combined with nutritional intervention on complications in maintenance hemodialysis patients [J]. Heilongjiang Medicine, 2024, 37(3): 682-4.

1830 LI A-MIN, ZHOU Li-Heng, LIHENG Z. Exercise-cognitive dual-task exercise in hemodialysis patients with combined cognitive decline [J]. Nursing Research, 2024, 38(6): 1064-7.

1831 LI CHUN YAN, CAO TING TING, TING TING C. Effects of multimodal exercise therapy on fatigue status and exercise capacity of maintenance hemodialysis patients [J]. Contemporary Nurse, 2024, 31(10): 21-4.

1832 Li H.Y., Du Juan, Sun Xiaohui, et al. Effectiveness of exercise program combined with self-management in dialysis applied to hemodialysis patients [J]. Xinjiang Medicine, 2024, 54(4): 485-8.

1833 Li Pengcheng. Observation on the efficacy of scopolamine combined with high flux hemodialysis in the treatment of patients with acute organophosphorus poisoning [J]. Medical Theory and Practice, 2024, 37(3): 424-6.

1834 Li Shuyue, Sun Wanting, Gong Xiang, et al. Effects of cycling training and respiratory training on exercise endurance, lung function and quality of survival of maintenance hemodialysis patients [J]. Chinese Journal of Rehabilitation Medicine, 2024, 39(5): 687-92.

1835 Li Xiaoqin. Effects of hypoxia-inducible factor stabilizer on hematopoietic function and iron death in renal anemia patients on hemodialysis [J]. International Journal of Urology, 2024, 44(2): 310-4.

1836 Li Yucui. Effects of an empowerment model-based exercise intervention on frailty in elderly hemodialysis patients [J]. Evidence-Based Nursing, 2024, 10(10): 1819-24.

1837 Li Yucui, Tong Li, Li Yang, et al. The effect of physical exercise on patients' negative emotions in dialysis based on the Knowing, Believing and Doing model [J]. Journal of Nurse Advancement, 2024, 39(1): 71-6.

1838 LIAO Xianyong, ZHONG Yanghong, ZHOU Yishan, et al. Clinical efficacy and effect on oxidative stress and inflammatory response in patients with chronic renal failure treated by adding ginseng qiqi and resolving turbidities in combination with high flux hemodialysis [J]. World Journal of Integrative Medicine, 2024, 19(4): 789-93,99.

1839 Liu Cong, Yang Jiju, Li Hongdian, et al. Net Meta-analysis of Different Exercise Modalities for the Treatment of Sleep Disorders in Hemodialysis Patients [J]. Tianjin Traditional Chinese Medicine, 2024, 41(5): 600-11.

1840 Liu Feng, Qiu Mingtao, Liao Wen, et al. Gabapentin combined with exercise training in patients with maintenance hemodialysis combined with restless legs syndrome syndrome [J]. China Medical Innovation, 2024, 21(10): 150-4.

1841 Liu Tingting, Luo Yu, Fan Jianning, et al. Vitamin D supplementation to improve oxidative stress in patients with end-stage renal disease [J]. China Hospital Drug Evaluation and Analysis, 2024, 24(4): 422-5,30.

1842 LIU Yanyan, DONG Yongqing, ZHANG Song, et al. Impact of Total Nursing Intervention on Hemodialysis Patients with Renal Failure [J]. Journal of Community Medicine, 2024, 22(9): 306-11.

1843 LUO Lianhua, CHENG Lin, LIN C. Effects of group acceptance and commitment therapy combined with aerobic exercise on hemodialysis patients [J]. Chinese and Foreign Medical Research, 2024, 22(10): 144-7.

1844 QIN Jiaqi, JIANG Changying, LIU Zhiyan, et al. Summary of the best evidence on sleep disorders in maintenance hemodialysis patients [J]. World Abstracts of Current Medical Information (Continuous Electronic Journal), 2024, 24(11): 102-5,14.

1845 Shi Shuai, Li Xiowen, Zhao Yiting, et al. Efficacy of leucovorin-assisted continuous low-efficacy slow hemodialysis in the treatment of end-stage diabetic nephropathy and its effects on patients' quality of life and oxidative stress [J]. Modern Biomedical Progress, 2024, 24(4): 713-7.

1846 Shi Haiyan, Zang Xiujuan, Chen Peng, et al. The effect of quantitative aerobic exercise under the guidance of cardiopulmonary exercise test on cardiopulmonary function and quality of life of hemodialysis patients [J]. Chinese Journal of Nephrology, 2024, 25(2): 142-5.

1847 Song Q. Effects of cognitive-behavioral interventions on compliance behavior, exercise capacity, and risk events in hospitalized peritoneal dialysis patients [J]. Journal of Qiannan National Medical College, 2024, 37(1): 107-9.
[truncated: 464,894 more chars]
