# Supplementary material for: Meta-analysis of the effects of exercise interventions on dialysis patients with cardiac function disorders
Source: Front Med (Lausanne). 2025 May 13;12:1573498. doi: 10.3389/fmed.2025.1573498 (PMC12106465; doi:10.3389/fmed.2025.1573498)
Supplement: Supplementary file 5 [file Table_5.docx]

Updated protocol

Table Protocol deviations

| Section | Previous protocol | Update |
| --- | --- | --- |
| Title | the effect of exercise intervention on cardiovascular function in dialysis patients:a systematic review and meta-analysis | Meta-analysis of the Effects of Exercise Interventions on Dialysis Patients with Cardiac Function Disorders |
| Authors | liu huizhen, huang tingrong, chen minqi, chen liusi | Huizhen Liu, Ming Zhang, Minqi Chen, Liusi Chen, Tingrong Huang, Zhao Ye |
| Search strategy | From January 2010 to July 2024, two researchers searched Chinese and English databases using terms such as "dialysis", "hemodialysis", "peritoneal dialysis", "exercise", and "randomization"： English databases(Pubmede, Web of Science, Cochrane Library) and Chinese databases (CNKI, CBM, Wanfang, VIP) | From January 2010 to July 2024, two researchers searched Chinese and English databases using terms such as " Renal Dialysis ", "Aerobic Exercise", "Resistance Exercise", "Physical Activity ", "Stretching", "Cycling", "physical fitness", "exercise training ", and "Randomized Controlled Trial ", combined with free-text keywords for comprehensive searches. English databases(Pubmede, Web of Science, Cochrane Library) and Chinese databases (CNKI, CBM, Wanfang, VIP) |
| Study quality assessment | Two independent reviewers will evaluate the risk of bias in all included studies with the Cochrane  Collaboration's tool assessment method and complete the Standards for Reporting Interventions in Clinical。 | In this study, two independent researchers (H.Z.L. and M.Q.C.) utilized the risk of bias tool developed by the Cochrane Collaboration to conduct a quality assessment. The assessment criteria included random allocation methods, allocation concealment, blinding of assessors, integrity of outcome data, selective reporting, and other potential biases. |
| Data synthesis | No meta regression analysis. | No meta regression analysis. |
|  |  |  |
